# Supplementary material for: The Adeno-Associated Virus Replication Protein Rep78 Contains a Strictly C-Terminal Sequence Motif Conserved Across Dependoparvoviruses
Source: Viruses. 2024 Nov 12;16(11):1760. doi: 10.3390/v16111760 (PMC11598897; doi:10.3390/v16111760)
Supplement: Supplementary file 1 [file viruses-16-01760-s001.zip › Suppl File S2_Multiple sequence alignment of Dependoparvovirus Rep78 proteins.pdf]

Suppl File S2: **Multiple sequence alignment of *Dependoparvovirus* Rep78 proteins**

Note: the Rep78 protein of the bank vole dependoparvovirus MgAAV2 (Genbank accession QHD57625) most probably contains a sequencing error in its C-terminus, resulting in an artefactual frameshift. Indeed:

1) its C-terminal sequence is highly dissimilar its sequence to that of all other dependoparvovirus Rep78;

2) instead, the frameshifted nucleotide sequence is highly similar to that other dependoparvovirus Rep78 and contains a DDx3EQ C-terminal motif.

The alignment below thus includes the last 50aa of Mg AAV2 Rep78 with the artefactual frameshift approximately corrected.

```
>AAV2|NC_001401.2_cds_YP_680423.1_2/1-621
--------------M--------------------------------------------PGFYEIVIKVPSD
LDEHLPGISDSFVNWVAEKE--WELPP----DSDMDLNLI-----------EQAPLTVAEKLQRDFLTEWRR
VSKA-------PEALFFVQFEKG-ESYFHMHVLVETTGVKSMVLGRFLSQIREKL-IQRIYRGIEPT-LPNW
FAVTKTRNGA-GGGNKVVD---ECYIPNYLLPK-------TQPE---------LQWAWTNMEQY---LSACL
NLTERKRLVAQHL-THVSQTQE--------------QNKENQ-----NPNSDAPVI-RSKTSARYM------
ELVGWLVDKGITSEKQWIQEDQASYISFNAASNSRSQIKAALDNAGKIMSLTKTAPDYLVGQQPVED--IS-
SNRIYKILELNGYDPQYAASVFLGWATKKFGKRNTIW-LFGPATTGKTNIAEAIAHTV-PFYGCVNWTNENF
PFNDCVDKMVIWWEEGKMTAKVVESAKAILGGSKVRVDQKCKSSAQIDPTPVIVTSNTNMCAVIDGNSTTFE
HQQPLQDRMFKFELTRRLDHDFGKVTKQEVKDFFRWAK-DHVVEVEHEFYVK----------K-G-------
------------GAK--KRP-----------------------------------APSD-------------
--------------ADISE-PKR-VRE-SVAQPSTSDA--------------------------E-ASINYA
DRYQNKCSRHVG-MNLMLFPCRQC--ER-------------MNQNS---------NICFTHGQ---------
--------KDCLECFP---VSE----------------------SQ-----PVSVV----------------
-----------------------KKA------YQ-KLCYIHHIMGKVPD------------ACTACDLVNV-
-------------------------------D-LDDCIFEQ-------
>AAV4|NC_001829.1_cds_NP_044926.1_1/1-623
--------------M--------------------------------------------PGFYEIVLKVPSD
LDEHLPGISDSFVSWVAEKE--WELPP----DSDMDLNLI-----------EQAPLTVAEKLQREFLVEWRR
VSKA-------PEALFFVQFEKG-DSYFHLHILVETVGVKSMVVGRYVSQIKEKL-VTRIYRGVEPQ-LPNW
FAVTKTRNGA-GGGNKVVD---DCYIPNYLLPK-------TQPE---------LQWAWTNMDQY---ISACL
NLAERKRLVAQHL-THVSQTQE--------------QNKENQ-----NPNSDAPVI-RSKTSARYM------
ELVGWLVDRGITSEKQWIQEDQASYISFNAASNSRSQIKAALDNASKIMSLTKTAPDYLVGQNPPED--IS-
SNRIYRILEMNGYDPQYAASVFLGWAQKKFGKRNTIW-LFGPATTGKTNIAEAIAHAV-PFYGCVNWTNENF
PFNDCVDKMVIWWEEGKMTAKVVESAKAILGGSKVRVDQKCKSSAQIDPTPVIVTSNTNMCAVIDGNSTTFE
HQQPLQDRMFKFELTKRLEHDFGKVTKQEVKDFFRWAS-DHVTEVTHEFYVR----------K-G-------
------------GAR--KRP-----------------------------------APND-------------
--------------ADISE-PKR-ACP-SVAQPSTSDA--------------------------E-APVDYA
DRYQNKCSRHVG-MNLMLFPCRQC--ER-------------MNQNV---------DICFTHGV---------
--------MDCAECFP---VSE----------------------SQ-----PVSVV----------------
----------------------RKRT------YQ-KLCPIHHIMGRAPEV-----------ACSACELANV-
-------------------------------D-LDDCDMEQ-------
>AAV3|NC_001729_1_cds_NP_043940/1-624
--------------M--------------------------------------------PGFYEIVLKVPSD
LDERLPGISNSFVNWVAEKE--WDVPP----DSDMDPNLI-----------EQAPLTVAEKLQREFLVEWRR
VSKA-------PEALFFVQFEKG-ETYFHLHVLIETIGVKSMVVGRYVSQIKEKL-VTRIYRGVEPQ-LPNW
FAVTKTRNGA-GGGNKVVD---DCYIPNYLLPK-------TQPE---------LQWAWTNMDQY---LSACL
NLAERKRLVAQHL-THVSQTQE--------------QNKENQ-----NPNSDAPVI-RSKTSARYM------
ELVGWLVDRGITSEKQWIQEDQASYISFNAASNSRSQIKAALDNASKIMSLTKTAPDYLVGSNPPED--IT-
KNRIYQILELNGYDPQYAASVFLGWAQKKFGKRNTIW-LFGPATTGKTNIAEAIAHAV-PFYGCVNWTNENF
PFNDCVDKMVIWWEEGKMTAKVVESAKAILGGSKVRVDQKCKSSAQIEPTPVIVTSNTNMCAVIDGNSTTFE
HQQPLQDRMFEFELTRRLDHDFGKVTKQEVKDFFRWAS-DHVTDVAHEFYVR----------K-G-------
------------GAK--KRP-----------------------------------ASND-------------
--------------ADVSE-PKR-ECT-SLAQPTTSDA--------------------------E-APADYA
DRYQNKCSRHVG-MNLMLFPCKTC--ER-------------MNQIS---------NVCFTHGQ---------
```

--------RDCGECFPG--MSE----------------------SQ-----PVSVV----------------
----------------------KKKT------YQ-KLCPIHHILGRAPEI-----------ACSACDLANV-
-------------------------------D-LDDCVSEQ-------
>lcl|AY631966.1_cds_AAT46338.1_1/1-622
--------------M--PGFYEIVIKVPSD
LDEHLPGISDSFVNWVAEKE--WELPP----DSDMDRNLI-----------EQAPLTVAEKLQRDFLVHWRR
VSKA-------PEALFFVQFEKG-ESYFHLHVLVETTGVKSMVLGRFLSQIRDRL-VQTIYRGVEPT-LPNW
FAVTKTRNGA-GGGNKVVD---ECYIPNYLLPK-------TQPE---------LQWAWTNMEEY---ISACL
NLAERKRLVAQHL-THVSQTQE--------------QNKENL-----NPNSDAPVI-RSKTSARYM------
ELVGWLVDRGITSEKQWIQEDQASYISFNAASNSRSQIKAALDNAGKIMALTKSAPDYLVGPSLPAD--IK-
ANRIYRILELNGYDPAYAGSVFLGWAQKKFGKRNTIW-LFGPATTGKTNIAEAIAHAV-PFYGCVNWTNENF
PFNDCVDKMVIWWEEGKMTAKVVESAKAILGGSKVRVDQKCKSSAQIDPTPVIVTSNTNMCAVIDGNSTTFE
HQQPLQDRMFKFELTRRLEHDFGKVTKQEVKEFFRWAQ-DHVTEVAHEFYVR----------K-G-------
------------GAT--KRP-----------------------------------APSD-------------
--------------ADISE-PKR-ACP-SVPEPSTSDA--------------------------E-APVDFA
DRYQNKCSRHAG-MLQMLFPCKTC--ER-------------MNQNF---------NVCFTHGV---------
--------RDCSECFPG--ASE----------------------SQ-----P--VV----------------
----------------------RKKT------YQ-KLCAIHHLLGRAPEI-----------ACSACDLVNV-
-------------------------------D-LDDCVSEQ-------
>AAV1|NC_002077.1_cds_NP_049541.1_1/1-623
--------------M--PGFYEIVIKVPSD
LDEHLPGISDSFVSWVAEKE--WELPP----DSDMDLNLI-----------EQAPLTVAEKLQRDFLVQWRR
VSKA-------PEALFFVQFEKG-ESYFHLHILVETTGVKSMVLGRFLSQIRDKL-VQTIYRGIEPT-LPNW
FAVTKTRNGA-GGGNKVVD---ECYIPNYLLPK-------TQPE---------LQWAWTNMEEY---ISACL
NLAERKRLVAQHL-THVSQTQE--------------QNKENL-----NPNSDAPVI-RSKTSARYM------
ELVGWLVDRGITSEKQWIQEDQASYISFNAASNSRSQIKAALDNAGKIMALTKSAPDYLVGPAPPAD--IK-
TNRIYRILELNGYEPAYAGSVFLGWAQKRFGKRNTIW-LFGPATTGKTNIAEAIAHAV-PFYGCVNWTNENF
PFNDCVDKMVIWWEEGKMTAKVVESAKAILGGSKVRVDQKCKSSAQIDPTPVIVTSNTNMCAVIDGNSTTFE
HQQPLQDRMFKFELTRRLEHDFGKVTKQEVKEFFRWAQ-DHVTEVAHEFYVR----------K-G-------
------------GAN--KRP-----------------------------------APDD-------------
--------------ADKSE-PKR-ACP-SVADPSTSDA--------------------------EGAPVDFA
DRYQNKCSRHAG-MLQMLFPCKTC--ER-------------MNQNF---------NICFTHGT---------
--------RDCSECFPG--VSE----------------------SQ-----P--VV----------------
----------------------RKRT------YR-KLCAIHHLLGRAPEI-----------ACSACDLVNV-
-------------------------------D-LDDCVSEQ-------
>AAV8|NC_006261.1_cds_YP_077179.1_1/1-625
--------------M--PGFYEIVIKVPSD
LDEHLPGISDSFVNWVAEKE--WELPP----DSDMDRNLI-----------EQAPLTVAEKLQRDFLVQWRR
VSKA-------PEALFFVQFEKG-ESYFHLHVLVETTGVKSMVLGRFLSQIREKLGPDHLPAGSSPT-LPNW
FAVTKDAVMAPAGGNKVVD---ECYIPNYLLPK-------TQPE---------LQWAWTNMEEY---ISACL
NLAERKRLVAQHL-THVSQTQE--------------QNKENL-----NPNSDAPVI-RSKTSARYM------
ELVGWLVDRGITSEKQWIQEDQASYISFNAASNSRSQIKAALDNAGKIMALTKSAPDYLVGPSLPAD--IT-
QNRIYRILALNGYDPAYAGSVFLGWAQKKFGKRNTIW-LFGPATTGKTNIAEAIAHAV-PFYGCVNWTNENF
PFNDCVDKMVIWWEEGKMTAKVVESAKAILGGSKVRVDQKCKSSAQIDPTPVIVTSNTNMCAVIDGNSTTFE
HQQPLQDRMFKFELTRRLEHDFGKVTKQEVKEFFRWAS-DHVTEVAHEFYVR----------K-G-------
------------GAS--KRP-----------------------------------APDD-------------
--------------ADKSE-PKR-ACP-SVADPSTSDA--------------------------EGAPVDFA
DRYQNKCSRHAG-MLQMLFPCKTC--ER-------------MNQNF---------NICFTHGV---------
--------RDCSECFPG--VSE----------------------SQ-----P--VV----------------
----------------------RKRT------YR-KLCAIHHLLGRAPEI-----------ACSACDLVNV-
-------------------------------D-LDDCVSEQ-------
>AAV12|DQ813647/1-621
--------------M--PGFYEVVIKVPSD
LDEHLPGISDSFVNWVAEKE--WELPP----DSDMDQNLI-----------EQAPLTVAEKLQREFLVEWRR
VSKF-------LEAKFFVQFEKG-DSYFHLHILIEITGVKSMVVGRYVSQIRDKL-IQRIYRGVEPQ-LPNW
FAVTKTRNGA-GGGNKVVD---ECYIPNYLLPK-------VQPE---------LQWAWTNMEEY---ISACL
NLAERKRLVAQHL-THVSQTQE--------------GDKENL-----NPNSDAPVI-RSKTSARYM------

ELVGWLVDKGITSEKQWIQEDQASYISFNAASNSRSQIKAALDNASKIMSLTKTAPDYLIGQQPVGD--IT-
TNRIYKILELNGYDPQYAASVFLGWAQKKFGKRNTIW-LFGPATTGKTNIAEAIAHAV-PFYGCVNWTNENF
PFNDCVDKMVIWWEEGKMTAKVVESAKAILGGSKVRVDQKCKASAQIDPTPVIVTSNTNMCAVIDGNSTTFE
HQQPLQDRMFKFELTRRLDHDFGKVTKQEVKDFFRWAA-DHVTDVAHEFYVT----------K-G-------
------------GAK--KRP-----------------------------------APSD-------------
--------------EDISE-PKR-PRV-SFAQPETSDA--------------------------E-APGDFA
DRYQNKCSRHAG-MLQMLFPCKTC--ER-------------MNQNS---------NVCFTHGQ---------
--------KDCGECFP---GSE----------------------SQ-----PVSVV----------------
-----------------------RKT------YQ-KLCILHQLRG-APEI-----------ACSACDQLNP-
-------------------------------D-LDDCQFEQ-------
>RhinolophusPusillusBtAAV-CXC1|MK391482.1_cds_QDX47269.1_1/1-636
---MSFYEVIIRVPHD
IEEHLPGISDHFVDWIANKE--WELPD----WADLDITLI-----------DMPQLTLAEKIQREFLTEWSK
ITKE-------KELVYFIQFEKG-EKFYHLHTLMSTEGMKSMVLGRYLNQIRQKL-VSQSYRGIEPQ-IPDW
LAVTKTKK---EGANRLRD---RDYIPAYLLPK-------VQSE---------LQWAWTSMDEY---KLATL
NLEERKRLVAAFQ-AELALKKS--------------AAEESQI---EKNGVGNPRW-RGKTTAKYM------
ELVNWLVERGITSEREWIKEDQESYLSFNSTGTARGQIKAALDNASRIMSTTKEAADYLIGQDVPED--IT-
SNRVYRIFEMNGYDPKYAGSILVGWCQKRFGKRNTVW-LFGPATTGKTNIAEAIAHAV-PFYGCVNWTNENF
PFNDCVDKMIIWWEEGKMTAKVVESAKAILGGSMVRVDQKCKSSSQIDQTPVIVTSNTNMCAVIDGNSTTFE
HQQPLEDRMFKFELTRRLDSDFGKITKAEIKDFMAWAR-ANEVPVTHEFRVK----------R-H-------
------------APIFSKRP-----------------------------------APEG-------------
----------EEP-EGIKD-CKR-FRS-SSFEEEQSSR--------------------------E-EPDNFA
LRYVNKCSRHLN-FVQMMFPCQIC--DR-------------MNQSV---------DVCFTHGA---------
--------KSCEICFP-----R----------------------GE-----PDG------------------
-------------IET-EVQEAQKEEQNFLAAYP-NRCNICKMRG-IYNP-----------ACGLCAEII--
-------------------------------DCIDDVNKEQ-------
>RhinolophusSinicusRs-BtAAV1|MF682927.1_cds_ATV81502.1_1/1-654
--------------MAGYLSGSSQP----------------ASHSTSACR------GVKMSFYEVIIRVPHD
VEEHLPGISDHFVDWIANKE--WELPE----WSDMDITLI-----------DMPQLTLGEKLKREFLLEWHK
ITKL-------SNLDYFIQLEKG-EKFYHLHVLISVEGMKSMVLGRYLNQIRQKL-VSQIYRGIEPT-IPDW
LAVTKTKK---EGANRIRD---TGYIPAYLLPK-------VQSE---------LQWAWTSLEDY---KLATL
NLQERKRLADEFQ-AELALSRP--------------VIEETE--------AGNPRW-RGKSTAKYM------
ELVKWLVEKGITSEKEWIKEDQESYLTFNSTGTARGQIKAALDNAGRIMSTTKQAADYLIGPDVPPD--IK-
SNRVYRIFEMNGYDPRYAGSVLVGWCQKKFGKRNTVW-LFGPATTGKTNIAEAIAHAV-PFYGCVNWTNENF
PFNDCVDKMIIWWEEGKMTAKVVESAKAILGGSMVRVDQKCKSSSQIEQTPVIVTSNTNMCAVIDGNSTTFE
HQQPLEDRMFKFELTRRLDSDFGKISKTEIREFMAWAA-ANEVPVRHEFRVQ----------R-S-------
------------ITSLGKRP-----------------------------------APEG-------------
----------EEPGEGINA-AKR-PR--SSFDEPQPEG--------------------------E-DVENFA
LRYVNKCSRHLN-FVHMMFPCQIC--DR-------------MNQSV---------DVCFTHGV---------
--------KSCEICFP-----R----------------------GE-----PDG------------------
-------------VEE-QAKEAEKEEQNFIDAYP-NRCNICKMRG-HFNP-----------ACGLCAEIA--
-------------------------------DCIDDLNKEQ-------
>AAVbatCroatiaCull_12|QHY93489.1/1-635 replicase [Adeno-associated virus Croatia cul1_12]
---MSFYEVIIRVPHD
VEDQLPGVSDSFVDWIVRKE--WELPD----WSDMDITLI-----------DQPQLTLAEKIQREFLSEWQK
ITKE-------KETPHFVQLEKG-EKFYHLHTLVSTEGMKSMVLGRYLNQIRQKL-VSQIYRGVEPQ-IPDW
MAVTKVKK---EGANRIRD---TQYIPAYLLPK-------TQSE---------LQWAWTNLEEY---KLATL
NLEERKRLSEQYQ-TEFRRNQP--------------A-SDSQI---SETDSGNPRW-RGKSTARYM------
ELVKWLVERGITSEKQWIQEDQESYLSFNSTGTARGQIKAALDNAGRIMSTTKSASDYLIGPHPPED--VK-
NNRVYRIFELNGYDPKYAGSILVGWCRKEFGKRNTVW-LFGPATTGKTNIAEAIAHAV-PFYGCVNWTNENF
PFNDCVDKMIIWWEEGKMTAKVVESAKAILGGSMVRVDQKCKSSQQIEQTPVIVTSNTNMCAVIDGNSTTFE
HQQPLEDRMFKFELTRRLEPDFGKISKREIREFMNWAE-SNKVPVTHEFRVK----------K-F-------
------------APIF-KRH-----------------------------------APEG-------------
----------EES-EGINN-RKR-FRS-SSFEEESSER--------------------------A-DVENFA
LRYVNKCSRHLN-FVTMMFPCQIC--DR-------------MNQSV---------DVCFTHGA---------
--------KSCEICFP-----R----------------------SD-----PDGQVD---------------

```
------------CIEMGESEKAEKEE------MPENRCNTCKMRN-IFRP-----------NCGLCAELAL-
-------------------------------ECIDDVNKEQ-------
>AAV5|NC_006152.1_cds_YP_068408.1_1/1-610
--------------M--------------------------------------------ATFYEVIVRVPFD
VEEHLPGISDSFVDWVTGQI--WELPP----ESDLNLTLV-----------EQPQLTVADRIRRVFLYEWNK
FSK--------QESKFFVQFEKG-SEYFHLHTLVETSGISSMVLGRYVSQIRAQL-VKVVFQGIEPQ-INDW
VAITKVKK---GGANKVVD---SGYIPAYLLPK-------VQPE---------LQWAWTNLDEY---KLAAL
NLEERKRLVAQFLAESSQRSQE--------------AASQRE-----FSA--DPVI-KSKTSQKYM------
ALVNWLVEHGITSEKQWIQENQESYLSFNSTGNSRSQIKAALDNATKIMSLTKSAVDYLVGSSVPED--IS-
KNRIWQIFEMNGYDPAYAGSILYGWCQRSFNKRNTVW-LYGPATTGKTNIAEAIAHTV-PFYGCVNWTNENF
PFNDCVDKMLIWWEEGKMTNKVVESAKAILGGSKVRVDQKCKSSVQIDSTPVIVTSNTNMCVVVDGNSTTFE
HQQPLEDRMFKFELTKRLPPDFGKITKQEVKDFFAWAK-VNQVPVTHEFKVPRELAG----TK-G-------
------------AEKSLKRP-----------------------------------LGDV-------------
----------TNT-SYKSL-EKR-ARL--SFVPETPRSS--DVTVDP----------------APLRPLNWN
SRYDCKCDYHAQ-FDNISNKCDEC--EY-------------LNRGK---------NGCICHNV---------
--------THCQICHG----------------------------IP-----PWE------------------
----------------------KENLSDF-------------------------------------------
-------------------------------GDFDDANKEQ-------
>BovineAAV|NC_005889.1:368-2200/1-610
--------------M--------------------------------------------ATFYEVIVRVPFD
VEEHLPGISDNFVDWVTGQI--WELPP----ESDLNLTLI-----------EQPQLTVADRIRRVFLYEWNK
FSK--------QESKFFVQFEKG-SEYFHLHTLVETSGISSMVLGRYVSQIRAQL-VKVVFQNIEPR-INDW
VAITKVKK---GGANKVVD---SGYIPAYLLPK-------VQPE---------LQWAWTNLEEY---KLAAL
NLEERKRLVAQFQLESSQRSQE--------------ASSQRD-----VSA--DPVI-KSKTSQKYM------
ALVSWLVEHGITSEKQWIQENQESYLSFNSTGNSRSQIKAALDNASKIMSLTKSASDYLVGQTVPED--IS-
ENRIWQIFDLNGYDPAYAGSVLYGWCTRAFGKRNTVW-LYGPATTGKTNIAEAISHTV-PFYGCVNWTNENF
PFNDCVEKMLIWWEEGKMTSKVVEPAKAILGGSRVRVDQKCKSSVQVDSTPVIITSNTNMCVVVDGNSTTFE
HQQPLEDRMFRFELMRRLPPDFGKITKQEVKDFFAWAK-VNQVPVTHEFMVPKKVAG----TE-R-------
------------AETSRKRP-----------------------------------LDDV-------------
----------TNT-NYKSP-EKR-ARL--SVVPETPRSS--DVPVEP----------------APLRPLNWS
SRYECRCDYHAK-FDSVTGECDEC--EY-------------LNRGK---------NGCIFHNA---------
--------THCQICHA----------------------------VP-----PWE------------------
----------------------KENVSDF-------------------------------------------
-------------------------------NDFDDCNKEQ-------
>ParvoviridaeDogfe340C4|WDW25818.1/1-610 MAG: replication-associated protein
[Canine parvovirus]
--------------M--------------------------------------------ATFYEVIVRVPFD
VEDHLPGISDSFVDWVTGQT--WELPP----ESDLNLTLI-----------EQPQLTVADRIRRVFLYEWNK
FSK--------QESKFFVQFEKG-SEYFHLHTLVETSGISSMVLGRYVSQIRAQL-VKVVFQNTEPR-INDW
VAITKVKK---GGANKVVD---SGYIPAYLLPK-------VQSE---------LQWAWTNIDEY---KLATL
NLEERKRLVAQFQLESLQHSQE--------------ASSQRD-----FSA--DPVI-KSKTSQKYM------
ALVSWLVEHGITSEKQWIQENQESYLSFNSTGNSRSQIKAALDNASKIMSLTKSASDYLVGQTVPED--IS-
ENRIWQIFDLNGYDPAYAGSVLYGWCTRAFGKRNTVW-LYGPATTGKTNIAEAISHTV-PFYGCVNWTNENF
PFNDCVDKMLIWWEEGKMTSKVVEPAKAILGGSRVRVDQKCKSSVQVDSTPVIITSNTNMCVVVDGNSTTFE
HQQPLEDRMFRFELMRRLPPDFGKITKQEVKDFFAWAM-ANQVPVTHEFMVPRKVAG----NK-K-------
------------AETSKKRQ-----------------------------------LVDV-------------
----------TST-SYKSP-EKR-ARL--SVAPETPRSS--DVPVEP----------------APLRPINWC
SRYECRCDYHAQ-FESVTGECADC--EY-------------LNRGK---------NGCMFHNA---------
--------THCQICHA----------------------------VP-----PWE------------------
----------------------KENVPDF-------------------------------------------
-------------------------------NDFDDCNKEQ-------
>CaliforniaSeaLion|NC_038539.1:291-2093/1-600
--------------M--------------------------------------------ASFYEVVAKIPSD
LEEHLPGICDDFVSVIMSKE--WRLPE----SSDLVLDQI-----------DQPKLTIADAVRKVFYFEWLK
YCRD------VEEPLFFFQFEKG-KENFHVHMVIETSNVSSMVLGRYIGTIKKKL-VRKVFREVEPQ-MPDW
LAVTKTKQS--GGVNKTYD---KGYIPAYLLPK-------TQPE---------LQWAWTNIEEY---KSASL
NLAERKRLVDEFLA-SLRRDGP--------------SQSEPD-----DQQPHGPVI-RNRTSQKYM------
```

```
ALVSWLVENGITSEKQWIQEDQESYLSFNAAGSSRSQIKSALDNASRIMSLTKKASDYLVGQSVPED--IT-
ENKIYQLFKMNGYDPAYLGSILLGWCQGRFGKRNTVW-LYGPATTGKTNLAEAIAHSV-PFYGCVNWTNENF
PFNDCVDKMLIWWEEGKMTSKVVESAKAILGGSKVRVDQKCKSSVQIDSTPVIITSNTDMCCVIDGNSTTFE
HRQPLEDRMFRINLEQRLSHDFGKITKREVREFLAWAQ-EYEVDVEHTFEVT-KLA------K-P-------
------------KVTKRSAP-----------------------------------LSD--------------
--------------DYKSP-AKR-ARLIPDLVAEEATSS----ALAE----------------AEEWDLNWD
RRYDCRCEAHSM-SVRVEGLCRDC--EY-------------LNRGK---------NMCLVHGD---------
--------TGCHVCHA----------------------------VP-----PW-------------------
----------------------------V-------------------------------------------
-------------------------------SDPDDCTDEQ-------
>WXG22705.1/1-613 MAG: rep protein [Bat adeno-associated virus 2]
-----------------------------------------------------------MSFYELVVKIPHD
LEENVPGAADSFVNWLLNKR--WELPE----GSDLDLDKI-----------DQPQLTLSDKIQRTILTDWRR
ITRS-------PGIKYFVQFEKG-SCYYHMHVLLEVGDVKSLVVGRYVSQMRKTI-VKVVYRDIEPR-LPDW
MALAKTKT---NGANRMRD---EGYIPAYLLPK-------RQPE---------LQWAWTNIQKF---ERAAL
NVSERRRVCEEEY-ADLALSQQ--------------SDASQN-----NYSESAPRR-VGRAAFDYM------
ALVKWLVEKGITSERAWMREDSDSYLSYNATGASRAQIKSSLDNACRIMANTKTAADYLIGDEVPED--IE-
DNRMFRIFKLNGYDPAYAASVLLGWCQRGFGKRNTVW-LFGPATTGKTNIAEAIAHSV-PFFGCVNWTNENF
PFNDCVDKMVIWWEEGKMTAKVVESAKAILGGSIVRVDQKCKSSAQIEQTPVIITSNTNMCEVIDGNSTSFE
HRQPLEDRMFKFELLHRLPPDFGKITKAEVRGFFAWAK-SVRVEVEPAFSVK----------K-I-------
------------PALFLKRP-----------------------------------RPEG-------------
----------AEPSEAINP-GKK-ARH-ESFAP----------ETLP-----------------EEEPANFA
LRYVHKCSRHLA-MNQMLFPCRNC--ER-------------MNLNC---------NICVPHGV---------
--------SDCTICFP-----RLEP------------------DGA-----PEPSV----------------
---------------VHDNHDLPVDNWDF---YV--------------------------------------
--------------------------------NLDDKNKEQ-------
>BatAAV_YNM|NC_014468/1-613
-----------------------------------------------------------MEFYSIVLRLPGD
FDSEVPGLQDSFYKWISGPR--RELPE----WSDLDPGQI-----------ESAYQILADKLVREFAQKWAA
FSED-------PRAPYFAQLEKG-RENFHVHVLASSKKVGSFVVGRYVRKMRQHL-VDVVFRKCEPV-DADW
LQVQKSGN---HKSNEIKD---EGFIPAYLLPK-------RQSE---------LQWAWTNIEKY---ERATL
SVAERARLVEEWK--RSLAAEE--------------SDPAE--------PERRPRK-STKSASEYM------
ALVRWLVDNGIATEREWMREDSDGYLSYNATGATRAQIKAALDNAARIMVNTKTAADYLVGRNPPLD--VE-
DNRIYRLFRMNGYDPAYAGSVLLGWCRTGFGKRNTVW-LFGPATTGKTNLAEAISHSV-PFYGCVNWTNENF
PFNDCVDKMIIWWEEGKMTSKVVESAKAILGGSKVRVDQKCKNSQQIEPTPVIITSNTNMCEVVDGNSTTFE
HRQPLEDRMFKFELTVRLQPTFGKITKQEVREFFKWAE-LNAVDVEYDFLVR----------K-I-------
------------NQSDTGGG-----------------------------------VKRG-------------
----------AEPTKDEPP-AKR-VFF-YGATSEGEDVR----EGAP----------------GESDSVNFA
ERYVSKCSKHLS-WSNMRYPCRAC--ER-------------MNADV---------NVCTPHGC---------
--------RDCPECFP-----R----------------------PA-----PVPIA----------------
---------------EHDLCLAPIEDSDF---YV--------------------------------------
-------------------------------GCIDDVNKEQ-------
>BatAAV2|WXG22610.1/1-611 MAG: rep protein [Bat adeno-associated virus 2]
-----------------------------------------------------------MEFYSVVIRLPND
FSDEVPGLQESFFAWIMSDR--NPLPE----WSDLDPGQI-----------DKPYQIVADKMVRECVAKWDG
LSRT-------ENQPYFAQLEKR-PENVHIHLLLSAKVIASFVAGRFFKHVVKHL-VDVVYRSCAPT-ADGW
FEVRKSST---RGANLIQT---EGFIPAYLLPK-------RQSE---------LQWAWTNIEKY---ARATL
NVAERARLVEEWKLERPSPKAE--------------SVSA------------VPVK-NAKSVTEYM------
ALVQWLVDNGIATERDWMRESTESYLSYNATGGSRAQIKAALDNAARIMINTKTASDYLIGASPPAN--VA-
DNRIYRLFALNGYDPAYAGSVLLGWCRNGFGKRNTVW-LFGPATTGKTNLAEAISHSV-PFYGCVNWTNENF
PFNDCVDKMIIWWEEGKMTSKVVESAKAILGGSKVRVDQKCKNSQQIEPTPVIITSNTNMCEVVDGNSTTFE
HRQPLEDRMFKFELAVRLQPSFGKITKQEVREFFKWAE-LNAVDVEYDFLVR----------K-I-------
------------GQSDTGGG-----------------------------------AKRG-------------
----------AEPSKDEPP-AKR-VFF-YGAPSEGEDVR----EGAP----------------GESDSVNFA
ERYVSKCSRHLS-WSNMRFPCRAC--ER-------------MNADA---------NVCTPHGC---------
--------GDCPDCFP-----R----------------------PA-----PVPIA----------------
---------------EHDLCLAPIEDSDF---YV--------------------------------------
```

```
-------------------------------GCVDDVNKEQ-------
>RhinolophusPusillus|MF682926__Rp-BtAAV1_34C_MJ_YN_2012/1-666
-----------------------------------------------------------MSYYGIVVRIPWD
VIYQLPQVADSVLIQHETAQ--IFTPD----DSDLDIYEI-----------NNVEWSMAQEMHEHFINKWIE
ITKH-------EAVKYFAQLEKG-KEFFHIHYVIEMTGMKSMCLGRYLNVIEGHV-IRETFGIYHHK-IDNF
IQIDKTRT---NGPNRQYD---DDFILAYLIPK-------RQPE---------LQWAWTTIDKF---ARAAL
NIAERARLAEEFQLGLAARREE--------------ARLERI-----SNNNGNPKI-CGKSSRRYM------
ELVNWLVEKGVTSERDWIKEDQESYLSYNATGSSRGQIKAALENACRIMSTVKKASDYLVGTEFPED--IE-
KNRVYRIFQMNGYDPAYAGSIFLGWCRRQFGKRNTIW-LFGPATTGKTNIAEAVAHAV-PFYGCVNWTNENF
PFNDCVDKMIIWWEEGKMTAKIVESAKAILGGSTVRVDQKCKSSCQIEQTPVIVTSNTNMCAVIDGNSTTFE
HQQPLEDRMFKFELTTRLQSDFGKISKAEIKEFLAWAD-VNRVEVKHEFRVQ----------RVY-------
------------THVMMKRP-----------------------------------APAW-------------
----------EEPTEGINT-AKK-LKT-ESVAAVPSEG-------------------------EE----DFG
QRYVNRCTNHMN-LTVMRFPCKFC--AK-------------MNQTE---------DICFTHGA---------
--------KSCEICFPTNNVVKLSPVKIQYEEKKEQI------VEA-----PKEQILLC-------------
---------EKMADVEEEIKKKIQEERDFVEQYG-DRCNICKMRG-WFNP-----------KCGLCAEIK--
-------------------------------GLMDDLNKEQ-------
>ParvoviridaeDogfe340C1|WDW25820.1/1-602 MAG: Rep [Canine parvovirus]
--------------M--------------------------------------------ASFYQFVVKLPED
FASDLPNLSPALEESLTNFQ--VQIPD----WSDLNVNKI-----------DEIHVGIGERIQRCFNQEWRR
KSNN-------EEFKYFYQLEKA-SDKHHIHMMVETTGVKSMVLGRYVNQIKKTL-VDWVYGGAEPQFEEDW
FHVCKTSPV--GGTNRVYS---QSFIPTYLLPK-------KQEE---------LQWAWTNIKKY---IKAAL
SERVRDEIVEEHK-VELAAR-------------------EEA-----GEFQSTPKV-RNKTAETYM------
KLVQWLVENGITSERQWIQEHQESYLSYNASSSNRSQIKAAMDNASKIMSLTKKASDYLIGKCVPEN--IC-
NNRIYKILKLNNYHVEYVASILLGWSRGEFGKRNTIW-FFGPATTGKTNIAEAIAHAV-PFYGCVNWTNENF
PFNDCVDKMVIWWEEGKMTSKVVESAKAILGGSRVRVDQKCKSSVQIDPTPVVITSNTDMTVVVDGNSTTFE
HRQPLEDRIFKFFLEERLPDDFGKVTKEEVQQFFAWAE-QNRVKVEPCFTVP----------KSM-------
------------SGHALKRP-----------------------------------ASSC-------------
----------EG--EDIKR-AKS-AQP-SVADSLLIQADA-GARGSP---------------PTDWRPERWH
SRYFERCDFHGQREAIIEQFCKYC--EY-------------LNRGR---------AYCFPHEN---------
--------VRCQICHA----------------------------RA-----PWD------------------
---------------------------------------------------------------------NN-
-------------------------------VELEDVNKEQ-------
>UAJ21440.1/1-646 nonstructural protein 1 [Duck adeno-associated virus]
--------------MCA------------------------------------------ATYYEVIVELPSD
LETQLPLVSDSFVNWVTSKE--WEPPL----DSKWDMDQV-----------DQVQLTLGDKIQREILKHWRT
ITGN-------PEAKYFVQLEQG-ESYFHLHTLLECCKVKPLVLGRYINQIEKKL-VSTVYGGHNPQ-IDNW
MRITKTKSV--GGSNKIRA---ESYIPAYLIPK-------QQPE---------VQWAWTNIEEY---IKACL
NTDLRGSIAAEHF-EKQGLD----------------GPKKQSL---QFSSDGAPVI-ESKSSKRYM------
GLVGWLVEKGITTEKQWILENQESYLSFQASGNSARQIKTALDNAAKLMLLTKTAPDYLVGKDMVED--IT-
ENRIYKILEMNNYDPAYVGNIFLGWSKQQFGKRNTIW-LFGPATTGKTNIAEAIAHAV-PFYGCVNWTNENF
PFNDCTDKMIIWWEEGKMTAKVVETAKAILGGSKVRVDQKCKSSIQLDPTPVIITSNTNMCYVVDGNTTTFE
HAQPLQDRMFKLELTRRLPDNFGKVTKQEVRDFFKWAE-QNPVEIVPEFHVR----------K---------
-------------AESRKRP-----------------------------------APE--------------
-----------EREDKSLA-AKA-PRT---------------DEPLP---------------SPGEEGTSLS
RRYVFKCAKHLG-MVTMMWPCKDC--ER-------------ANSAV---------NQCILHKS---------
--------LACKECYP------------DY-D-----------ENP-----PLSDTQLSETDS-------FK
TSLLEAKSQDPPLV----------NDKKW------TACSYHQLTGVAKP------------TCSMCQLRNV-
--------------------------------DLDDCNSEQ-------
>QKN88764.1/1-636 MAG: replication protein [Dependoparvovirus sp.]
--------------MPS------------------------------------------PTFYEVIVELPSD
LDTQLPLVSDSFVNWITSKT--WEPPL----DSDWDMEQV-----------DQVQLTLGDKIQREILKQWRV
ITGD-------PDPKYYVQLEQG-ESYFHLHTLLECCNIKPLVLGRYIKQIEKKL-VSTVYGGHTPL-IDNW
MRITKTKAI--GGSNKIRA---QSYIPAYLIPK-------KQPE---------VQWAWTNIEEY---IKAVL
NASLRHSIGEEHV-QQQGLV----------------SRDSDNL---ARNSEGAPII-ASKCSKKYM------
ELVDWLVEKGITTEKQWLLENKESYRSFQASSNSARQIKAALQGAVQEMLLTKTASDYLVGKDPIGD--IT-
ENRIYQIMEMNGYDPLYVANLLVGWCQMKYGKRNTIW-LFGPATTGKTNIAEAIAHAV-PFYGCVNWTNENF
```

PFNDCLEKMIIWWEEGKMTAKIVETAKAILGGSKVRVDQKCKSSMQLEPTPVIITSNTNMCYVVDGNTTTFE
HAQPLQDRMFKLELLKRLPDDFGKVTKKEVKDFFAWGA-KNTLEVKSEFLVR----------K---------
-------------AESRKRH-----------------------------------APE--------------
----------VGSEDKSPP-AKA-PRA---------------DERQP---------------SSGEEGTSIS
SRYVLKCAKHLG-MVTMMWPCRDC--EK-------------ANCNI---------NQCILHKT---------
--------LSCKECFP------------DY-DVDS---------SP--------------------------
---------QPPLVKSEDELSKDKDSKIW------TPCSYHHLTGVANH------------KCSMCKLRNV-
--------------------------------DLDDCDDEQ-------
>QLF86431.1/1-650 MAG: replication protein [Dependoparvovirus sp.]
--------------MPS--PTFYEVIVELPSD
LDTQLPLVSDSFVNWITSKT--WEPPL----DSDWDMEQV-----------DQVQLTLGDKIQREILKQWRV
ITGD-------PDPKYYVQLEQG-ESYFHLHTLLECCNIKPLVLGRYIKQIEKKL-VSTVYGGHTPL-IDNW
MRITKTKAI--GGSNKIRA---QSYIPAYLIPK-------KQPE---------VQWAWTNIEEY---IKAVL
NASLRHSIGEEHV-QQQGLV----------------SRDSDNL---VRNSEGAPII-ASKCSKKYM------
ELVDWLVEKGITTEKQWLLENKESYRSFQASSNSARQIKAALQGAVQEMLLTKTASDYLVGKDPIGD--IT-
ENRIYQIMEMNGYDPLYVANLLVGWCQMKYGKRNTIW-LFGPATTGKTNIAEAIAHAV-PFYGCVNWTNENF
PFNDCLEKMIIWWEEGKMTAKIVETAKAILGGSKVRVDQKCKSSMQLEPTPVIITSNTNMCYVVDGNTTTFE
HAQPLQDRMFKLELLKRLPDDFGKVTKKEVKDFFAWGA-KNTLEVKSEFLVR----------K---------
-------------AESRKRH-----------------------------------APE--------------
----------VGSEDKSPP-AKA-PRA---------------DERQP---------------SSGEEGTSVS
ARYVLKCAKHLG-MVTMMWPCRDC--EK-------------ANCNI---------NQCILHKT---------
--------LSCKECFP------------DY-EQESV-------TTP-----PLS------------------
ESDEEVTSHQPPLVNLRDELLRSEKQKTW------TPCSYHHLTGVANH------------KCSMCKLRNV-
--------------------------------DLDDCDDEQ-------
>DependoparvoBird|QKN88762.1/1-647 MAG: replication protein [Dependoparvovirus sp.] Isolate hftbif18par1
--------------MPS--PTFYEVIVELPSD
LDTQLPLVSDSFVNWITSKT--WEPPL----DSDWDMEQV-----------DQVQLTLGDKIQREILKQWRV
ITGD-------PDPKYYVQLEQG-ENYFHLHTLLECCNIKPLVLGRYIKQIEKKL-VSTVYGGHTPL-IENW
MRITKTKAI--GGSNKIRA---QSYIPAYLIPK-------KQPE---------VQWAWTNIEEY---IKAVL
NAALRHSIGEEHF-QQQGLA----------------LRDSTNL---ARNSEGAPII-ASKCSKKYM------
ELVDWLVEKGITTEKQWLLENKESYRSFQASSNSARQIKAALQGAVQEMLLTKTASDYLVGKDPIGD--IT-
DNRIYKILEMNGYDPLYVANLFVGWCQMKFGKRNTIW-LFGPATTGKTNIAEAIAHAV-PFYGCVNWTNENF
PFNDCTEKMIIWWEEGKMTAKIVETAKAILGGSKVRVDQKCKSSIQLEPTPVIITSNTNMCYVVDGNTTTFE
HAQPLQDRMFKLELLKRLPDDFGKVTKKEVKDFFAWGA-KHTLKVNSEFLVQ----------K---------
-------------AESRKRH-----------------------------------APE--------------
----------VGSEDKSPP-AKA-ARA---------------DELQP---------------SSGEEGTSVS
VRYVLKCAKHLG-MVTMMWPCRDC--EK-------------ANCNI---------NQCILHKT---------
--------LSCKECFP------------DY-D-DT---------NP-----PSS------------------
SSDEDIPSHQPPLVSIRDEPSRGEKSNIW------VPCSYHHLTGVANH------------KCSMCKLRNV-
--------------------------------DLDDCDNEQ-------
>QKN88760.1/1-658 MAG: replication protein [Dependoparvovirus sp.]
--------------MPS--PTFYEVIVELPSD
LDTQLPLVSDSFVNWITSKT--WEPPL----DSDWDMEQV-----------DQVQLTLGDKIQREILKQWRV
ITGA-------PDPKYYVQLEQG-ESYFHLHTLLECCNIKPLVLGRYIKQIEKKL-VSTVYGGHTPL-IENW
MRITKTKAT--GGSNKIRA---QSYIPAYLIPK-------KQPE---------VQWAWTNIEEY---IKAVL
NASLRHSIGEEHV-QQQGLVSRDSQEDVQRQS--LFSRDSDNL---ARNSEGAPII-ASKCSKKYM------
ELVDWLVEKGITTEKQWLLENKESYRSFQASSNSARQIKAALQGATQEMLLTKTASDYLIGKDTVGD--IT-
ENRIYQIMEMNGYDPLYVANLLVGWCQMKYGKRNTIW-LFGPATTGKTNIAEAIAHAV-PFYGCVNWTNENF
PFNDCLEKMIIWWEEGKMTAKIVETAKAILGGSKVRVDQKCKSSMQLEPTPVIITSNTNMCYVVDGNTTTFE
HAQPLQDRMFKLELLKRLPDDFGKVTKKEVKDFFAWGA-KNTLEVKSEFLVR----------K---------
-------------AESRKRH-----------------------------------APE--------------
----------VGSEDKSPP-AKA-PRA---------------DERQP---------------SSGEEGTSVS
SRYVLKCAKHLG-MVTMMWPCRDC--EK-------------ANCNI---------NQCILHKT---------
--------LSCKECFP------------DY-DLDF---------SPR-EGSPL-------------------
-------KSQPPLVKSEDELSKDKDSKIW------TPCSYHHLTGVANH------------KCSMCKLRNV-
--------------------------------DLDDCDNEQ-------

```
>AAV_MHH-05-2015|NC_040671.1:431-2377/1-648
--------------MTT------------------------------------------PTYYELIVELPSD
IDTQLPLVSDSFVRWVTSKT--WEPPL----DSKWDMDQV-----------DQVQLTLGDKIQREILKQWRT
ITGD-------PDPKYYVQLEQG-ETYFHLHTLLQCCNIKPLVLGRYVKQIEKKL-VSTVYGGHNPL-IDNW
LRITKTKSI--GGSNKIRA---QSYIPAYLIPK-------KQPE---------VQWAWTNIEEY---IKAVL
NSELRHQIGEAHF-QEQGLA----------------LRDSTNL---SRNSEGAPII-VSKCTKKYM------
ELVEWLVEKGITTEKQWLLENKESFRSFQASSNSARQIKAALQGATQEMLLTKTASDYLIGKDPIGD--MT-
DNRIYKILEMNGYDPLYVANLFVGWCQMKFGKRNTIW-LFGPATTGKTNIAEAIAHAV-PFYGCVNWTNENF
PFNDCLEKMIIWWEEGKMTAKIVETAKAILGGSKVRVDQKCKSSMQLEPTPVIITSNTNMCYVVDGNTTTFE
HAQPLQDRMFKLELLKRLPDDFGKVTKKEVRDFFAWGA-KHTVEVDSCFLVR----------K---------
-------------AESRKRH-----------------------------------APE--------------
----------VASEDKSPP-AKA-ART---------------DELQH---------------LSGEEGTSVS
ARYVLKCAKHLG-MVTMMWPCRDC--EK-------------ANCNI---------NQCILHKS---------
--------LSCKECFP------------DY-DSDVSVQEGEPSGNP-----PLS------------------
SSDEDIPSHQPPLV---------KDCKPW------TPCSYHHLTGVANR------------NCSMCKLRNV-
--------------------------------DLDDCDSEQ-------
>AvianAAV|NC_006263.1:244-2235/1-663
----------------M------------------------------------------RSYYEVIVQLPND
VESQVPGISDSFVNWITSRE--WTLPE----DADWDLDQV-----------DQVQLTLGDKIQREIRNHWGT
MAKE-------PDFHYFIQLEQG-EVFFHLHVLLETCSVKPMVLGRYIRHIQQKI-VSKVYCGHEPA-MEGW
MRVTKTKNF--GGANKVRA---ESYIPAYLIPK-------QQPE---------VQWAWTNVPEY---IKACL
HRELRASLARLHF-EEAGLS-----------------QSKENL---ARTADGAPVI-ATRVSKRYM------
ELVDWLVEKGITTEKEWLLENRESFRSFQASSNSARQIKTALQGAIQEMLLTKTAEDYLVGKEPVSDDEIR-
QNRIYKILELNHYDPAYVGSILVGWCQKKWGKRNTLW-LFGPATTGKTNIAEAIAHAV-PFYGCVNWTNENF
PFNDCVEKMIIWWEEGKMTAKVVETAKAILGGSRVRVDQKCKASVPIEPTPVIITSNTNMCYVIDGNTTTFE
HKQPLEDRMFKLELLTRLPDDFGKVTKQEVRQFFRWSQ-DHLTPVIPEFLVR----------K---------
-------------AESRKRP-----------------------------------AP---------------
-----------SGEGYISP-TKR-PAL---------------AEQQQ---------------AS-ESAEPVP
TRYRIKCSKHCG-MDKMLFPCQIC--ES-------------MNRNI---------NICAIHKT---------
--------TECKECFP------------EYGDKDTV-----P-ELP-----PCTEHNVSRCYQCHSGELYRV
TSD----SDEKPAP-ESDE----GTEPSY------APCTIHHLMGKSRGL----------VSCAACRLKNST
L-----------------------------HDDLDDGDLEQ-------
>WBY51256.1/1-654 replication protein [Psittacidae dependoparvovirus]
-----------MAAGRD------------------------------------------TQFFELIVQLPSD
IETQLPNISESFVNWITGKQ--WRLPE----GSDWNLDKV-----------DQVQLTLGDKIQREIYRYWVS
ITHN-------ENAEFFVQLEES-PNYFHLHVLLECCGIKPLVLGRYVKHLQQKL-ISTVYSGHAPQ-IENW
LRITKTKAV--GGSNKMRG---KSYIPAYLIPK-------VQPE---------VHWAWTNIPEY---APACL
NARLRAEIAETHF-LEAGYTQR--------------TPPEGGL---AKNGDGAPVI-LSKASRRYM------
ELVDWLVDKGITTEKQWLTENKESYRSFQASGNSARQIKVALQNAIQEMLLTKTAPDYLIGRDPPDEEQIRT
SNRIYDIFQRNGYDPAYAANVLVGWCKGAFNKRNTVW-LFGPATTGKTNIAEAIAHAV-PFYGCVNWTNENF
PFNDCLNKMIIWWEEGKMTAKIVESAKAILGGSRVRVDQKCKSSEEIQPTPVIITSNTNMCHVIDGNSTTFE
HQKPLEDRMFLFELVKPLPPNYGKVTKQEVLDFFRWGL-DHPIEVTHEFRVP----------K---------
-------------AAASKRKRTNEN--------------------------EFSSTPEAIKEAFGTA-----
-------SPPIRREDVFAV-TA------------------------------------------SGDSSADL
DRYERKCTKHLH-LDKVRFYCSAC--EG-------------INRRL---------DVCFSHGT---------
--------LNCEECFP-------------------------------------Q------------------
-----------------------ENKAVF------EACASCHLMGKKPDP----------VKCPACKLKNVA
LLMGEDSSDE---------SSLVRP-----EQEWDDTLNEQ-------
>Psittacidae|QTE03943.1/1-676 MAG replication-associated protein [Psittacidae
dependoparvovirus]
--------------MDS------------------------------------------NKFYEIIVQLPND
IEAELPNISDSFVNWISNRE--WTLPE----GSDWDLDQV-----------DQVQLTLGDKIQREMYRHWVG
ITHD-------ENAYFFTQLEQS-PNYFHLHVLFQCCGIKPMVLGRYVKQIQQRV-VSTVYSGHNPQ-IENW
MRITKTKNV--GGCNKIRD---KSYIPAYLIPK-------QQPE---------VHWAWTNIQEY---VNACL
NARLRQEIAEAHL-QEAGF-EN--------------LSQEGGL---ARNSDGAPVI-PGKASRRYM------
ELVDWLVDRGITTEKQWLTENKESYRSFQASSNSARQIKAALQNAIQEMLLTKSAPDYLIGKTIPSEEDIR-
KNKIYDIFQRNGYDPAYAANVLVGWCKGAFNKRNTIW-LFGPATTGKTNIAEAIAHAV-PFYGCVNWTNENF
```

```
PFNDCLNKMIIWWEEGKMTAKVVESAKAILGGSRVRVDQKCKSSEEIQPTPVIITSNTNMCHVIDGNSTTFE
HQKPLEDRMFLFELVKVLPPDFGKVTKQEVRDFFRWGI-ENPIEVTHEFRVP----------K---------
-------------AAASKRKSTNQN-----------------------PDDEFQFTPETIKRATTSA-----
-------FQSVSRENTFDS-TSR-FEE---------------TRTQK---------------ETSGSSTPEL
DRYERKCAKHLH-LDKVRFYCSGC--ET-------------INRRL---------DVCFSHGV---------
--------LNCEECFP------------S--D-------------------PVQ------------------
-----------------------KIKLCY------QPCAICHLLGKSPDV----------KKCAACRLKNVT
MQIIEESSSESEGRASNEASSLIRP-----EQEWDDTLNEQ-------
>Dependo_wpk049par01|QKE60686.1/1-675 MAG: replication protein
[Dependoparvovirus sp.]
--------------MAN------------------------------------------PSYFEFVIRLPAD
VEEDLPGIPDSFIDWLCRES--PELPE----GSDLDPEQI-----------EMPMVVLGQRIIKIILNAWRS
ITKQ-------QDVKYYVQLEKV-DSAFHLHVLVETCKVPSFTLGRHVNRIKEDI-VRQVYQKVAPR-IDDW
FSIAKTRGG--HKSNKIHS---ESYIDAYLLGK-------VQPE---------LQWAWTNIPAY---VSACL
SLAERQELVKNHR-IELGHRFS--R-----------SASISS-----STGSTAPVI-QTKSADRYM------
ALVDWLVENGITTAKQWLQEDKDSYLSFHATGSSSRQVKSALENACEVMSLTKTAADYLIGKQVPED--IT-
QNRIYKLFELNGYDPHYAGKILLGWCKREHGKRNTVW-LFGPATTGKTILAEAIAHAV-PFFGCVNWTNENF
PFNDCVNKMLIWWEEGKMTTKIVESAKAILGGSQVRVDQKCKSSSPIEQTPVIITSNTPMYRVYDGNSTTFE
HEKPLKDRMFCFEFLRPLDKTFGKVTKQEVKEFFAWAQAQDDVIVEDSFHVL----------K---------
-------------SRGVKRSHDCDP---------------------------LPICPEG-------------
----------AEPKEATPT-TEV-INISAAAPPSSVDDGT-ASSADP--------------PPPPPPDSAEV
SLYKNKCSRHLA-LSIVKYVCRDC--EL-------------LNKKG--------GDVCIMHNQ---------
--------EGCSQCFP------------GYEES----------IEP-----PVHKKSK--------------
-----------------------------------TVCGQCHLLGNNFCTHYEPMVIGKLMRCTSCVIKYR-
-------------------NGVTLPPWGDCQMCMDLDELEQ-------
>BirdAAV|QKE54950.1/1-601 replication protein [Parvoviridae sp.] METAGENOME
-------MCDLANALQSALQ-------------------------------------EGTKFYELIIRLPGD
VDSHLPGISETFIRWLTTTT--PTLPN----DHEWNLTLC-----------NQPLVVLATAVTNSITQKWRS
FQQ--------RTLRTFYQLEEG-TEYFHLHCLLETGDLRSFVLGRYVRQISLHL-TTTIFENTTPQ-LENW
ITITKSRR---GGQNKQQD---LQYILGYLIPK-------KQPE---------CHWAWSNIPLL---KKACL
NTTERDRLHALYA----------------DL----LGDKENQDPD-P-TINRAPII-SNRAARKYA------
EVVDWLVQQGITTEKQWLIEDKDSYRSFQSTANSSRQVRAALENARAEMLLTKSAQDYLIGRSASPD--MD-
TNRIYQIFLKNGYDPLLCSNILVRWATRAFNKRNTIW-LFGPATTGKTNIAEAIAHTV-PFYGCVNWTNENF
PFNDCVDKMIIWWEEGKMTNKVVEAAKAILGGSRVRVDQKCKGSVQIEPTPVIVTSNTDMTIVADGNSYTME
HKDPLEERMFKFLLTHKLPPSFGKITKQEVRAFLKWGQ-DNPVNVEPTFLVP----------K---------
-----------------------------------------------------AQEP---------------
------------------P-PT------DNIDSATNE------QPGP------------S----APRKRPAD
ELDAQPCSDHQDQNTLAVLACTKCHPETAG--KSPRFSNKYFRETF----------QCMFHKT---------
--------FNCLECYP---------------------------DTP--------------------------
-----------------------------------DSTDIE-------------------------------
----------------------------------DDVFDEQ-------
>Bird_cfw059par1QKN88755.1/1-599 capsid protein [Dependoparvovirus sp.]
-----------MNSVEELLH-------------------------------------HNKQYFELIIRLPGD
VDNHLPGISDHFVQWLTTTI--PDLPD----DHDWDVAKV-----------NQPLVVLAGAITHTISSKWRS
FQQ--------QTLKTFYQLEEG-TEYFHLHCLLEMGSIKSFVLGRYIRQIAQTL-TTNLFENRTPN-LENW
FSITKSRR---GGQNKIQD---HHYLLAYLIPK-------LQPE---------CQWAWTNIPLL---QKATL
NASERARLHNLYL----------------CN----ISEKENHTPT-PDSLTKAPII-SNRTARKYC------
EVVDWLVAQGITTEKQWLIEDKDSYRSFQSTANSSRQVRAALDNARAEMLLTKSAPDYLLGPSTSTD--MD-
TNRIYQIFLKNGYDPLLCANILVRWASRDFNKRNTIW-LYGPATTGKTNIAEAIAHAV-PFYGCVNWTNENF
PFNDCVDKMIIWWEEGKMTNKVVEAAKAILGGSKVRVDQKCKGSVQIEPTPVIITSNTDMTVVADGNSITME
HKEPLEERMFKFQLNIKLPIDFGKITKQEVRNFFRWGA-DNKVNIVPQFNVP----------Q-N-------
----------------------------------------------------INNAT---------------
------------------P-PD------SSLPCA---------QPGP------------ST-STTPTKRKAL
EPLATRCEEHADCNNLSVLCCSAC---TSGKPKSPKFSNKYFRENF----------HCMIHGT---------
--------FNCLECYP---------------------------LSS--------------------------
-----------------------------------DDTDID-------------------------------
----------------------------------DDVFAEQ-------
```

```
>MuscovyDuck|NC_006147.2:548-2431/1-627
------------MAFSRPLQI------------------------------------SSDKFYEVIIRLPSD
IDQDVPGLSLNFVEWLSTGVWEPT--------GIWNMEHV-----------NLPMVTLADKIKNIFIQRWNQ
FNQ--------DETDFFFQLEEG-SEYIHLHCCIAQGNVRSFVLGRYMSQIKDSI-LRDVYEGKQVK-IPDW
FSITKTKR---GGQNKTVT---AAYILHYLIPK-------KQPE---------LQWAFTNMPLF---TAAAL
CLQKRQELLDAFQ----------------E------SEMNAVVQE-DQASTAAPLI-SNRAAKNYS------
NLVDWLIEMGITSEKQWLTENKESYRSFQATSSNNRQVKAALENARAEMLLTKTATDYLIGKDPVLD--IT-
KNRIYQILKLNNYNPQYVGSVLCGWVKREFNKRNAIW-LYGPATTGKTNIAEAIAHAV-PFYGCVNWTNENF
PFNDCVDKMLIWWEEGKMTNKVVESAKAILGGSAVRVDQKCKGSVCIEPTPVIITSNTDMCMIVDGNSTTME
HRIPLEERMFQIVLSHKLEGNFGKISKKEVKEFFKWAN-DNLVPVVSEFKVP----------T-N-------
------------EQTKLTEP-----------------------------VPERANEPSE-------------
------------PPKIWAP-PTR-EEL-EEILRASPELFA-SVAPLP------------SSPDTSPKRKKTR
GEYQVRCAMHSLDNSMNVFECLEC--ERA---NFPEFQS--LGENF-----------CNQHGW---------
--------YDCAFCNE---------------------------LKD--------------------------
-----------------------------------DMNEIEHV-----------------------------
-------------------------------FAIDDMENEQ-------
>GooseParvovirus|NC_001701.1_cds_NP_043514.1_1/1-627
------------MALSRPLQI------------------------------------SSDKFYEVIIRLSSD
IDQDVPGLSLNFVEWLSTGVWEPT--------GIWNMEHV-----------NLPMVTLAEKIKNIFIQRWNQ
FNQ--------DETDFFFQLEEG-SEYIHLHCCIAQGNVRSFVLGRYMSQIKDSI-IRDVYEGKQIK-IPDW
FAITKTKR---GGQNKTVT---AAYILHYLIPK-------KQPE---------LQWAFTNMPLF---TAAAL
CLQKRQELLDAFQ----------------E------SDLAAPLPD-PQASTVAPLI-SNRAAKNYS------
NLVDWLIEMGITSEKQWLTENRESYRSFQATSSNNRQVKAALENARAEMLLTKTATDYLIGKDPVLD--IT-
KNRVYQILKMNNYNPQYIGSILCGWVKREFNKRNAIW-LYGPATTGKTNIAEAIAHAV-PFYGCVNWTNENF
PFNDCVDKMLIWWEEGKMTNKVVESAKAILGGSAVRVDQKCKGSVCIEPTPVIITSNTDMCMIVDGNSTTME
HRIPLEERMFQIVLSHKLEPSFGKISKKEVREFFKWAN-DNLVPVVSEFKVR----------T-N-------
------------EQTNLPEP-----------------------------VPERANEPEE-------------
------------PPKIWAP-PTR-EEL-EELLRASPELFS-SVAPIP------------VTPQNSPEPKRSR
NNYQVRCALHTYDNSMDVFECMEC--EKA---NFPEFQP--LGENY-----------CDEHGW---------
--------YDCAICKE---------------------------LKN--------------------------
-----------------------------------ELAEIEHV-----------------------------
-------------------------------FELDDAENEQ-------
>WGJ63685.1/1-628 REP [Goose parvovirus]
------------MALSRPLQI------------------------------------SSDKFYEVIIRLSSD
IDHDVPGLSLNFVEWLSTGVWEPT--------GIWNMEHV-----------NLPMVTLAEKIKNIFIQRWNQ
FNQ--------DETDFFFQLEEG-SEYIHLHCCIAQGNVRSFVLGRYMSQIKDSI-IRDVYEGKQIK-IPDW
FAITKTKR---GGQNKTVT---AAYILHYLIPK-------KQPE---------LQWAFTNMPLF---TAAAL
CLQKRQELLDAFQ----------------E------SDLAAPLPD-PQASTVAPLI-SNSATKNYS------
NLVDWLIEMGITSEKQWLTENRESYRSFQATSSNNRQVKAALENARAEMLLTKTATDYLIGKDPVLD--IT-
KNRVYQILKMNNYNPQYIGSILCGWVKREFNKRNAIMALTGPATTGKTNIAEAIAHAV-PFYGCVNWTNENF
PFNDCVDKMLIWWEEGKMTNKVVESAKAILGGSAVRVDQKCKGSVCIEPTPVIITSNTDMCMIVDGNSTTME
HRIPLEERMFQIVLSHKLEPSFGKISKKEVREFFKWAN-DNLVPVVSEFKVR----------T-N-------
------------EQTNLPEP-----------------------------VPERANEPEE-------------
------------PPKIWAP-PTR-EEL-EELLRASPELFS-SVAPIP------------VTPQNSPEPKRSR
NNYQVRCALHTYDNSMDVFECMEC--EKA---NFPEFQP--LGENY-----------CDEHGW---------
--------YDCAICKE---------------------------LKN--------------------------
-----------------------------------ELAEIEHV-----------------------------
-------------------------------FELDDAENEQ-------
>QKN88756.1/1-570 MAG: replication protein [Dependoparvovirus sp.]
----------------------------------------------------------MTSFFEIVVRLPND
FYAELPGISDAWVDNICEEN--LHPPD----TCDFNLDLV-----------EPPYVALAERIRQEINLEWSY
RAG---------PHKFFIQLEKG-EENYHIHVLLEPVEVKSFVFGRYLPGFKERI-KDRVYAGIEPQ-TADW
FEAAKVKK---GGANALRH---VSYITNYLLPK-------KQPE---------LQWAWTNLEEF---RLAAL
NLHERARLVEEQRL-ALEERHK-------EA-----TAPTNV----------TPRV-GGKSAERYM------
ALVNWLVTNGITSEKEWIQADQESYLTHNANSNSRAQIKTALDNASRIMQLTKTACDYLIGPEPPAD--VT-
TNRVYRIFEMNGYDPQLAGSILLGWANRRFGKRNAIW-LFGPATTGKTNIAEAIAHAV-PFYGCVNWTNENF
PFNDCVNKMLIWWEEGKMTAKIVESAKSILGGSRVRVDQKCKSSQQIDSTPVIITSNTDMTMVVDGNTTTFE
```

```
HREALEDRMFQFYLGKRLDNDFGKITKTEVREFFKWAE-LNPTTPPHVFRVP----------RVN-------
------------DAG-YKRPDSPAA------------------------SSYATERPS--------------
------------GEE---PAAKR-PRY-E-HKPVDNGRV--EKSTLDLWLE----------GNPTPAALVVH
DLMEAS----------------GS--V--------------FDMTV---------R----------------
---------EC----------------------------------------P--------------------
------------------------------------------------------------------------
-----------------------------------DSNKEQ-------
>MurineAAV1|MF416383.1_cds_AWB14637.1_1/1-572
----------------------------------------------------------MTSFYEVVVKLPSD
FYDDLPGIDHVWIDNLCEEN--LHPPD----TCDFDLSLV-----------EPAYVNLAERIKQEIILEWSY
RAG---------KHRYFIQLEKG-EQFFHLHVLLDTVDVKGFVFARYASGFKQRI-RERVYCGIEPL-IPDW
FEITKTKKV--GGANAVRG---EAYIYNYLLPK-------KQSE---------LQWAWTNIPTF---ELAAL
NLNERQRLLEERRAEELAAREE-------RQ-----QSDSNS----------QNRV-YGKAAERYM------
ALVNWLVSNGITSEKEWIQADQVSYLTQNATSNGRAQIKTALDNASRIMQLTKTATDYLIGPSPPAD--VT-
TNRVYRIFEMNGYDPQLAGSILLGWANRRFGKRNAIW-LYGPATTGKTNIAEAIAHAV-PFYGCVNWTNENF
PFNDCVNKMLIWWEEGKMTAKIVESAKSILGGSRVRVDQKCKASQQIDSTPVIITSNTDMTMVVDGNTTTFE
HREALEDRMFQFYLGKRLDNDFGKIDKKEVREFFKWAE-LNQVPPPHVFRVP----------RVH-------
------------DAG-YKRPDSPAA------------------------SSYATERPS--------------
------------GEE---PAAKR-PRY-E-HKPVDNGRE--EKSTLDLWFH----------GNPTPAPMVVH
DLMDSS----------------GS--V--------------FNMTI---------R----------------
---------EC----------------------------------------P--------------------
------------------------------------------------------------------------
-----------------------------------DSNKEQ-------
>QKN88758.1/1-565 MAG: replication protein [Dependoparvovirus sp.]
----------------------------------------------------------MALFFEIVVKLPND
FYADLPGIADAWVEAICEEE--LHPPD----TCDFDLSLV-----------EAPYVTLAERVRQEINLEWSY
RAG---------THKYFIQLEKG-EQFFHLHVLIDRVDVKSFIFGRYVPGFKERI-RDRVYGGIEPQ-LPDW
FTVSKTKK---GGSNAQRD---EGYIYAYLLPK-------KQSE---------LQWAWTNIPKF---ELAAL
NLHERQRLLDEKRAEDLAAEQE-------NP-----RTEHN------------PRS-FGKAAERYM------
ALVNWLVENGITSEKEWIRADQVSYLTQNATSNGRAQIRSALDNASRIMQLTKTAIDYLIGPTPPED--VT-
TNRVYKIFSLNGYDPRLAGSILLGWAARRFGKRNTVW-LYGPATTGKTIIARAIAHAV-PFYGNVNWNNENF
PFNDSVNKMLIWWEEGKITAKTVEAAKAILGGSDVRVDQKCKASQQIDTTPVIITSNTDMTLVVDGNTTTFD
HKEALEDRMFQFYLFKKLDHDFGRVTKEEIRGFFKWAE-LNPVDVPHVFRVP----------RTL-------
------------DIS----SSSPSA------------------------STFAASTSE--------------
------------SEE---PVAKR-PRY-E--QPVDKGRV--EKSTLDFWLE----------GRPTPKPTFVA
DLSDST----------------ES--V--------------FDMKV---------R----------------
---------EC----------------------------------------P--------------------
------------------------------------------------------------------------
-----------------------------------DSNKEQ-------
>MurineAAV2|MF416384.1_cds_AWB14639.1_1/1-565
----------------------------------------------------------MASFFEVVVKLPND
FFADLPGISDLWVETICNEE--LHPPD----TCDFDLSLV-----------ESPYVALAERVKQEIILEWSY
RAG---------THKYFIQLEKG-EHFFHLHVLLDCVNVKSFIFGRYVAGFRERI-RDRVYSGIEPQ-LPDW
LTSSKTKK---GGSNAQRD---EGYIYAYLLPK-------KQSE---------LQWAWTNIPKF---ELAAL
NLNERQRLLDEKRAEDLAAEAE-------NP-----KPEHN------------PRS-FGKAAERYM------
ALVNWLVENGITSEKEWIRADQVSYLTQNATSNGRAQIKSALDNASRIMQLTKTAIDYLIGPTPPED--VT-
TNRVYKIFSLNGYDPRLAGSILLGWAARRFGKRNTVW-LYGPATTGKTIIARAIAHAV-PFYGNVNWNNENF
PFNDSVNKMLIWWEEGKITAKTVEAAKAILGGSDVRVDQKCKASQQIDTTPVIITSNTDMTLVVDGNTTTFD
HKEALEDRMFQFYLFRKLDHTFGRVTKEEIRGFFKWAE-LNQVEVPHVFRVP----------RTL-------
------------DIS----SSSPSA------------------------STVAASTSE--------------
------------SEE---PADKR-PRY-E--QPVDNGRE--EKSTLDLWLE----------GRPTPKPMFVA
DLSAAK----------------SS--V--------------FDMKV---------R----------------
---------DC----------------------------------------P--------------------
------------------------------------------------------------------------
-----------------------------------DSNKEQ-------
>QTE04020.1/1-574 MAG: replication protein [Anser anser dependoparvovirus]
-----------------------------------------------------------MYFYEIIVKLPND
```

```
FDTELPGICDAWIDALCAEN--LTLPD----TSDLDASQI-----------EEAYVALGQRIVNEILLEWKY
RTG--------VRYEYFVQLEKG-EHGYHLHVLLEQSNVKSFVFGRYAPGIKRRI-VDKVYSGVEPV-IDLW
FKLSKTREQ--GGANALRH---ESYLYNYLLPK-------TQPE---------LQYAWTNIDKF---LLAAV
NLNERKRLVDEYRA-QFQKPSE-------DE-----ESAHSDG--------PVPKV-RNKTSERYM------
ALVKWLVENGITSEQEWVKTDMESFLTHNASSSSRAQIKTALDNASRIMQLTKTASDYLIGQKPPED--VS-
TNRVYRIFEMNGYDPRLAGSILYGWARRQFGKRNTIW-LYGPATTGKTIIAEAIAHAV-PFYGCVNWNNENF
PFNDSVNKMLIWWEEGKMTAKVVEAAKCILGGSRVRVDQKCKSSQQIESTPVIITSNTDLTVVVDGNTTTME
HRQPLEDRMFQFNLVHRLTPDFGKVTKQEIREFFKWAE-LNKTEVPDAFRVP----------RVQ-------
------------EDGEYISESSSAQ------------------------PSDETATSS--------------
------------GEE-HRP-SKR-PRY-E--DSVDDGGR--VKSTLEKWLL----------DNPMPTPASVS
DNSDSE----------------NP--V--------------FDMRV---------R----------------
---------EC----------------------------------------P--------------------
------------------------------------------------------------------------
-----------------------------------DSNKEQ-------
>WDW25764.1/1-605 MAG: replication-associated protein [Canine parvovirus]
--------------MCEQASSSFHF----------------RTPSSTH---------LAMEFFSLTAKLPTD
LDNDVPGISQSMRDNYYAAD--VQLPH----TSDMNPADI-----------DGPSVLVGGMVVDSMVRFWRE
KMM--------DDFQYFAQLELS-EHSFHVHCLLETKGTKGFVLGRYMPQLKERI-ESDVFGGNEIQ-LPYW
LKARKTKQQ--GGANVTVN---EGYILNYLLPK-------RQTE---------LQWAWSNIERY---ERALL
NIEERKRIEDEFKAEAQRRKAE-------GL-----EPEF-P-----------QRS-SSSASDRYM------
ALVNWLVEHGITSEKEWIQEDQESYIRHHTHSNGRAQIKAALDNAAKIMSLTKSAKDYLIGPSPPSS--PE-
ENRIYRIFETNGYDPKLAGSILLGWARRGFGKRNAIW-LYGPATTGKTNIAEAIAHAV-PFYGCVNWTNENF
PFNDCVDKMLIWWEEGKMTAKIVESAKAILGGSKVRVDQKCKSSQQIDSTPVIITSNTDMTVVVDGNITTME
HRQPLEDRMFMFYLGKRLPDDFGKISKREVREFFKWAE-LNKMEVVPEFNVP----------SE--------
--------------SDYKRKRETGI------------------------EPFVTRTPDSADA----------
-----------SSEA-PAP-KKP--RY-----------E--TKSKEQILYEHLLAENLGEISNET-----VN
DQHDVSCKHGKH------LFCAEC--D--------------FNKNV---------KT--YNKY---------
--------LEC----------------------------------------P--------------------
------------------------------------------------------------------------
-----------------------------------DSNKEQ-------
>BankVoleMgAAV1|QHD57622.1/1-593 replication protein [Adeno-associated virus]
-----------------------------------------------------------MEFFSVVIKLPTD
IDSDVPGVSENLRDEFYSAD--VPPPA----SSDMNPAEV-----------DSPSVEMASRIVRSVRNFWRE
KVM--------EDFCDFFQLELS-DDKFHIHCLFETKGTKGFVLGRYVPQFKERI-ESDVFGGNEIS-IPNW
FKPRKTKQQ--GGANQTVN---EGFILNYLLPK-------RQSE---------LQWAWSNIERY---ERALL
NLEERKRIEDEFKAENQRRKEQ-------GL-----EPEFKP-----------PRT-GGSASDRYM------
ELVNWLVEHGITNEKEWIQEDQESYIRHHTNSNGRAQIKSALDNAAKIMSLTKSAKDYLIGPCPPSS--PE-
ENRIYKIFQKNGYDPALAGSILVGWAKRQFGKRNAIW-LYGPATTGKTNIAEAIAHAV-PFYGCVNWTNENF
PFNDCVDKMLIWWEEGKMTAKIVESAKAILGGSKVRVDQKCKSSQQIDSTPVIITSNTDMRLVVDGNQTTYE
HKQPLEDRIFRFYLGKRLPDDFGKISKREVREFFKWAE-TNKVEVTPTFMVP----------VL--------
--------------GDHKRKRETDC------------------------EPFVARSSNETDALPDS------
----------LTEEE-SAP-KKT--RY-E---------E--QKTPAQIAYEQILK----AIADDTPKPDLVH
DYHDVTCKHGKH------LFCADC--D--------------FNKNV---------KS--YNKY---------
--------LEC----------------------------------------P--------------------
------------------------------------------------------------------------
-----------------------------------DSNKEQ-------
>WDW25800.1/1-602 MAG: replication-associated protein [Canine parvovirus]
--------------MEDQ----------------------------------------KTTFFSITVDLPGD
FTTDVDNLPEAWLDNLFSAQ--LNLPP----ESDLNPEQI-----------EAVYVRLAEQIIEQIRLEWAF
RVG--------KSYADFYQLELA-DRGYHIHVLLETKDVKGFVFARFLPSFKQRI-RDRVYAGREIL-LSDW
FKTRKTKNV--GGANQTVN---EGYILNYLLPK-------RQSE---------LQYAWSNIEKY---VPALL
NISERERLVSEHLELVKQRQAE-------RA-----ANGEAN-----------PRV-SGKTSERYM------
ALVNWLVEHGITSEKEWIREDQESYLTHNASSSSRSQIKAALDNASKIMMLTKSAEDYLIGQSPPIC--PE-
ENRVYRIFEMNGYDPVVAGSILLGWAKRRFGKRNAIW-LYGPATTGKTNIAEAIAHAV-PFYGCVNWTNENF
PFNDCVDKMLIWWEEGKMSAKIVESAKAILGGSKVRVDQKCKASQQIDSTPVIITSNTDMTIVVDGNQTTRE
HRQPLEDRMFQFYLGRRLPDDFGKISKKEIREFFKWAE-LNRVDVISQFEVP----------AYR-------
--------------GDYKRKREPEP------------------------DSFPAQTLERTD-----------
```

```
------------ARE-SSP-AKT-PRY-ETVAETETKTE--TKSQAQILYEQILVENCGGITG----NVTIS
DHHDVECKHGKS------LFCTDC--E--------------FNKNV---------KT--HDKY---------
--------LEC----------------------------------------P--------------------
------------------------------------------------------------------------
-----------------------------------DSNKEQ-------
>ParvoviridaeDogfe352C3|WDW25804.1/1-601 MAG: replication-associated protein
[Canine parvovirus]
--------------MQN-----------------------------------------NTSFFEIRVKLPAD
FTCDVKSLPDAWMDELFGSS--IQLPD----TSDMNADQI-----------EPTYVNLSQRIVEQIRLEWNY
RVG--------KSYADFYQLEQT-ENGFHIHVLIETKDVKGFVLARFIPSFKQRI-TERVYAGCEIL-LDDW
FSPRKTKNV--GGANRTVD---KGYILTYLMPK-------KQSE---------LQWAWSNIDEY---SLALL
NLRERERLHTEYLAECAELRAQ-------RT-----ANGEAD-----------PEI-TGKTSEKYM------
SLVNWLVEHGITSEKEWIRENQKSYLTYNASSTNRAQIKSALDNAGKIMMLTKSAEDYLIGQSPPSY--VE-
GNRIYKIFKMNGYDPLLAGSILLGWAQRRFGKRNAVW-LYGPATTGKTNIAEAIAHAV-PFYGCVNWTNENF
PFNDCVDKMLIWWEEGKMTAKIVESAKAILGGSKVRVDQKCKSSQQIDSTPVIITSNIDMTIVVDGNQTTME
HRQPLEDRIFQFYLGRRLPDNFGKISKKEVREFFKWAE-LNRIDVTPCFHVP----------TV--------
--------------CQYKRKRESAP------------------------ESVRENTSEKTDVLPDA------
----------LPTEE-SSP-KKA--RY-E-----DKKTD--AKTPEQILYEQMLAENLGETKE----QPIVH
ENTDVACKHGKH------LFCSDC--D--------------FNKNV---------NT--YNKY---------
--------LEC----------------------------------------P--------------------
------------------------------------------------------------------------
-----------------------------------DSNKEQ-------
>BIRD2|QKE54991.1/1-612 replication protein [Parvoviridae sp.]
--------------MVP-----------------------------------------ETFFFEIRVLLPND
FNEDIERFPEDCIDEIFSAP--VYLPD----SSDMNPTLI-----------DNPYVTVAGRIVTAIRLEWAY
RVG--------KDFLDFYQLERT-ENGHHLHVLLETKDVKGFVLGRFLPSFRERI-QNRVYSGCHVL-IPDW
FKPRKNKNV--GGANRTVN---RGYIYNYLLPK-------RQSE---------LQWAWSNIEAL---QSALL
NISERERLVKEYLAQLKPVLDE-------RE-----ASGENH-----------PRI-GGKNSERYM------
ALVQWLVEQGITSEKQWIQENQDSYVSYNTSSTSRSQIKAALDNASKIMMLTKSAKDYLIGPSPPTC--LE-
ENRLYKIFKMNGYDPALAGSILIGWFQKKFGKRNAVW-LYGPATTGKTNIAEAIAHAV-PYYGCVNWTNENF
PFNDCVDKMLIWWEEGKMTAKIVESAKAILGGSKVRVDQKCKASQQIDSTPVIITSNTDMTVVVDGNITTME
HREPLEDRMFQFYLGRRLPDDFGKITKQEVREFFKWAE-LNQVEVTPCFRVP----------VR--------
---------------DLKRKRQPEV------------------------ESVAAETPEETD-APHAAAGTVS
DQEPDRGTVFLPAENYEEP-AKK-VRA-E---PRETHGG--PKQSLMEAYLQTLEANTPPSVG----PIEVH
DHSDVRCGHGKA------LFCDEC--D--------------FIKKV--------------------------
---------EC----------------------------------------P--------------------
------------------------------------------------------------------------
-----------------------------------DSNKEQ-------
>WRQ19904.1/1-508 MAG: rep protein [Adeno-associated virus]
------------------------------------------------------------------------
------------------------------------------------------------------------
-----------------------------MHLLFDTKGVKSIVLGRYVAQLLDRI-VNYVFGGAKPT-NPDW
LVICKTKPV--GGSNKIFS---ESYIPAYFLPK-------VQSE---------LQWAWTNIKKY---EPAAL
NLAERARIIEEHK-ASIAHKLV--------------FSDEEP--------EGTPVI-KNRASAKYM------
SLVNWLVENGITTEKQWIMENQESYISFNATGNSRSQIKSALDNAIKVMSLTKTAEDYLIGKDCPED--IS-
QNRIYKILRLNGYDPAYVGSIFLGWCRRSFGKRNTIW-LYGPATTGKTNIAEAISHTV-PFYGCVNWTNENF
PFNDCIDKMVVWWEEGKMTNKVVESAKAILGGSKVRVDQKCKASQQIDPTPVIITSNTDMCVVVDGNMNTYE
HKQPLEDRMFKIVLEERLDPEFGKITKQEVKDFFKWAE-DNKMKVEFDFLVK----------KAG-------
------------SVPA-KRE-----------------------------------APP--------------
--------------SDRVP-AKK-PRI-SVAD-FLIQ----NVTQND---------------DESYKKVDWN
SRYECRCDDHSN-DVIVKSVCLNC--EY-------------LNRNK---------NCCMKHNL---------
--------NKCEMCFR----------------------------YP-----PWQ------------------
-----------------------------------------------------------------CDVIDN-
-------------------------------VDLDDINKEQ-------
>Dependoparvovirus_zftwig05par3|QKN88780.1/1-613 MAG: replication protein
[Dependoparvovirus sp.]
-----------------------------------------------------------MEFYELIIQLPND
```

```
YSA----LSDGFIDSFSAVE----VPSV---SGDWNCSLV-----------DPLSWAVADSFVEAIR-NWSA
EISK------STAPSYFIQQEQG-EDYIHLHCCVETCIANSNVLGRYVNRLKGVL--AGLLEGHVHK--EDW
ISITKTGGS--SGKNKVCQ---ESYIIYYLLGK-------TQPD---------AIWAWSDIEKF---QDALL
DNTLRRELLEKYTAENPPKPKE----------------------------LKNPVV-ETLTAQKYM------
DLIDWLVEKGITTEKQWLLEDRTSFRSHQAGQGTARHIKEALKAAAQEILLTKCAKDYLVTKENDFEN-IK-
QNRIYKIMELNGYDPHLVACIFAKWSNREFGKRNTIW-LHGPATTGKTNIAEAIAHSV-PFYGCVNWTNDTF
PFNDCVNKLIIWWEEGKMTAKTVETAKAILGGSKVRVDQKCRGSEELEPTPVIITSNTNMCWVIDGNTTTYE
HKTPLQERMFKLELTVQLKPDFGKVTKQEVKQFFTWGA-TYPGIPPSVFAVE----------KKGGAATS--
----TAHAPLPTAPTGEKRKAEEEV-KSPTKKLLQTDIRSLTQ-----KKHWTDSIQEDVH-----------
----------LQTYKKWKS-ERE-TEMTENSVKKTIENF--N--------------------QGDGEG---A
YYYKMKCSKHLH-LDVLKYPCVEC--GR-------------ANWEI---------NCCRPHQL---------
--------KDCKECFP---------------------------IN---------------------------
------------------------------------------------------------------------
------------------------------------------------
>AvianAAV_BR_DF12|YP_010802670.1/1-600 Rep78 [Avian adeno-associated virus]
-----------------------------------------------------------MEFYEIVVQLPND
YSV----LSDGFIDAFTSTE----VPPV---AGHWDTTVI-----------DRLTWKVGDVFAEQIV-AWAT
ATAK------GKTPAFFIQEEQG-EEYVHLHCCIETVVSASHVLGRYVNKLKNVL--SGVLEGYVHK--DDW
ITINKSGGA--YTKNKVCD---LNYITFYLVGK-------TQPD---------VLWAWSNIDRY---QAALL
DNEERKRLLEEYIRENPPKKKE----------------------------LKTPVV-DTANAQKYM------
DLIDWLVEKGITTEKQWLLEDRTSFRSHQAGQGTARHIKEALKAAAQEILLTKCAKDYLVVKDQTYDD-IE-
DNRIYRIMKMNGYDPHVVAAIFSRWCNREYGKRNTVW-LHGPATTGKTNIAEAIAHAV-PFYGCVNWTNETF
PFNDCVNKLIIWWEEGKMTAKTVETAKAILGGSKVRVDQKCRGSEELEPTPVIITSNTNMCWVIDGNTTTYE
HKTPLQERMFKLELTTPLSPDFGKITKREVRQFFSWGA-AYEGVPEPVFQVP----------KTTSQAI---
-----------------KRAMTSTE-QSPPLKVQKVASHSLTQ-----KPHWTERLPEDVH-----------
----------LHAYRKWKA-ERD--QIQKGGVGTT--------TAEE---------------SSEGEG---P
FYYKQKCSKHLH-LEALKYQCLEC--SR-------------ANWEI---------NNCRPHGV---------
--------ANCKECFP---------------------------INE--------------------------
------------------------------------------------------------------------
-----------------------------------NA-----------
>Dependoparvovirus_ltt164par2|QLF86430.1/1-623 MAG: replication protein
[Dependoparvovirus sp.]
--------------MSSRDSERP--------RDDQSPSLLRECYSLTHSLDSLRAIPYRMSFYELIIQLPND
YSH----LSDGFFDTFNE-P----VPEV---QSDWDCSLV-----------DTLTWNVAYHFKEKIL-EWAD
GTSK------SKSPDHFIQLEKG-EEYDHLHCCIETCVAESHVLGRYVNKLKVRL--AGLLEGYVHK--EDW
ISISKTSSS--NSKNRVND---KNYILYYLLGK-------TQPD---------AVWAWSDIEEF---QEALL
DNTVRKSLLDKYLKENPPAPKV----------------------------KKTPVV-DTAAAQRYM------
DLVDWLVEKGITTEKQWLLEDRQSFRSHQAGQGTARHIKEALKAAAQEILLTKCAKDYLVIKDVDFAT-IE-
ENRIYRIMKLNGYDPHMIAAIFYRWCNREYGKRNTIW-LHGPATTGKTNIAEAVAHAV-PFYGCVNWTNDTF
PFNDCINKLIIWWEEGKMTAKTVETAKAILGGSKVRVDQKCKGSEELEPTPVIITSNTNMCWVVDGNTTTYE
HKEPLQERMFKLELTTQLDPSFGKITKEEVRQFFSWGA-LYEGTPTSEFMVS----------KAT-------
---------------GTKRKAEEAP-PTGLKKLLT-------------EKHWTDKMSEDVH-----------
----------LQTYRKWKE-SRE-KDLMENSVQKTIESF--K---------------------------EQE
YIYKMKCSKHLH-LDVMKYPCVEC--MK-------------ANYEI---------NCCNPHKL---------
--------KDCKECFP---------------------------INS--------------------------
------------------------------------------------------------------------
-----------------------------------S------------
>CanaryDependo1|WOP79071.1/1-688 replication protein [Canary dependoparvovirus
1]
MHDIRSLSSNHTAALTSSDLSPPIRDYLQDYKDSESST---HSHSLARRRGETDGTEKTMEFYEIVIQLPND
FSS----LSEDFIEAFSSAE----VPSL---ETDWDVSLV-----------DPLTWKVADAFVEHVI-AWAR
STSK------GKTPSYFIQQEQG-EEYVHLHCCIETVVSPSNVLGRYVNKLKTVL--AGLLEGYVHK--EDW
ISVTKAGGV--CSKNRTTN---TGYIVYYLLGK-------TQPD---------AIWAWSDLEEF---QAALL
DNAARKALLEKYVQDHPTQQKP----------------------------KKTPVI-DTANAQRYM------
DLIDWLVEKGITTEKQWLLEDRISFRSHQAGQGTARHIKEALKAAAQEILLTKCAKDYLVMKDGDFED-IK-
ENRIYKIMKINGYDPHMVACIFAKWCNREYGKRNTVW-LHGPATTGKTNIAEAIAHSV-PFYGCVNWTNDTF
PFNDCVNKLIIWWEEGKMTAKTVETAKAILGGSKVRVDQKCKGSEELEPTPVIITSNTNMCWVVDGNTTTYE
```

```
HKTPLQERMFKLELTVQLKPDFGKITKQEVRQFFTWGA-TYPGVPSSEFAVR----------KSAPVAVV--
----VEEKRQMAPPVGEKRKMESSSDQSPLRKMLVEGSGSLAQTT--NKTHWTEKLGEDVH-----------
----------LQTYRKWKA-TRE-AEMMKNSVRTTIENF--NNSEGT---------------EGGGEEEEKT
YIYKLKCSKHMH-LDVMKYPCLEC--SR-------------ANWEI---------NCCRPHKV---------
--------KDCIECFP---------------------------YPR--------------------------
------------------------------------------------------------------------
-----------------------------------EGEMKQ-------
>Dependoparvovirus_sis142par1|QKE54964.1/1-672 MAG: replication protein
[Parvoviridae sp.]
---------------MSSDLSPPITVRRLEYKESESRT---HSHSLTHRRRETDGAEERMEFYEIVIQLPND
FSS----LSEDFIETFSSAE----VPAV---DTDWDVSLV-----------DPLTWKVADVFVEHVI-SWSR
TTSK------GKTPSYFIQQEQG-EEYVHLHCCIETVVSPSNVLGRYVNKLKTVL--AGLLEGYVHK--EDW
ISVTKAGGV--CSKNRTTN---TGYIVYYLLGK-------TQPD---------AIWAWSDLEEF---QPALL
DNAARKALLEKYVQEHPIQQKP----------------------------KKTPVI-DTANAQRYM------
DLIDWLVEKGITTEKQWLLEDRISFRSHQAGQGTARHIKEALKAAAQEILLTKCARDYLVMKEGEFGD-IQ-
ENRIYKIMKINGYDPHMVACIFAKWSNREYGKRNTIW-LHGPATTGKTNIAEAIAHSV-PFYGCVNWTNDTF
PFNDCVNKLIIWWEEGKMTAKTVETAKAILGGSKVRVDQKCKGSEELEPTPVIITSNTNMCWVVDGNTTTYE
HKTPLQERMFKLELTVQLKPDFGKITKQEVRQFFTWGA-TYPGVPSSEFAVR----------KSAPVAVV--
----VEEKRQTMPPVGEKRKMEDASEQSPLRKLLVEGSGSLAQTT--KKPHWTEKLGEDVH-----------
----------LQTYRKWKA-TRE-AEMMKNSVKTTIESF--KSPGET---------------EGGGEGEE-T
YMYKMKCSKHMH-LDVMKYPCLEC--SR-------------ANWEI---------NCCRPHRV---------
--------KDCIECFP---------------------------YPR--------------------------
------------------------------------------------------------------------
-----------------------------------DGEMKQ-------
>FelineDependoparvo|QJQ50419.1/1-624 non-structural replication protein [Feline
dependoparvovirus]
----------------------------------------------------------MAQYYEIVMKLPND
ISTQVPGILESSVDVLTSQK--CELPS----LSYWDLTLV-----------PKAVVSLASCLVRVIIAFWQC
INPI-------AACPYFIQLEKG-KEWHHLHILLSDAACDSLVLGRYTNKLRHRL-VDAVYDGIEPD-IPDW
FCVHKTRL---GGKNKKVG---EDYIFRYLLSK-------VQPD---------VLWCWTDLHHL---QPLVL
DNRCRQELMVKAD----------------------RAAAESESTDQSEAADRAPVI-SGIGAANYS------
RLVEWLVEQGITSEKQWLETDKNSYRSFHANANSSRQIRAALENARVEMLLLKSAGDYLIGDSWPSD--IE-
SNKVYKLFTLNRYDPALVGGILLRWCQKLWGKRNTIW-LTGPASTGKTNLAEAIAHAV-PIYGCVNWTNENF
PFNDCTDKMIIWWEEGKMTAKLVEPAKAILGGSKVRVDQKCKQSVQIEPTPVIITSNIDMTLVIDGNSITRE
HEEPLQHRMWKIVLDSVLPPTWGKITSAEVKSFLAWAS-DQNEIVQPVFEVP----------RV--------
-----------------QTPIH-------------------------EQVMVDLTLPELDS-----------
-------VPCNR--GSSTPPSEE-PVIVPCATPPA------VRPSTP-----------------LPSPRYVR
SVT-YVCLEHDR------GDCDQC-SEE---------------------------EICFT------------
----QTDWLQCDERMS--TISEESG------------------IEPLLTPTPSPPSF---------------
-----------------------------------------EMYGWSPIT----------------------
-----------------------------LHEAF-DVDFPDPEE----
>DesmodusRotundus|MG745677.1_cds_AVR53758.1_1/1-717
--------------MGDLYWTAAER---------------WESDSSTHGYREGVILGKMLFFYELIIRLPND
LARDCPGMSETCMDALLHSS--VELPP----KAVWDMSKV-----------NLGMVSMAQCIVRILVCFWQM
LNPL-------VDTPYFIQLEEG-KQYYHLHCLLSWKACDSLVLGRYIKKVKERI-IEAAFGGIEPG-TTEW
FAVHKTRT---GGKNKVVD---ALYIERYLLPK-------KQSE---------VQWAWTDIPEY---KDAVL
DASRRAMFQLQMP----------------------VACLEDMPGD--PGAVPGPSI-SGSSAENYC------
RLVDWLVSEGITSERQWLQKDKLSYRSFHANSNSSRQIKAALENAKAEMLLTRTAGDYLVGGTLHNP--ID-
TNKIFLLFCLNNYDPQVAATILLKWCRCEWGKRNTVW-FTGPASTGKTNLAEAIAHAV-PLYGCVNWTNENF
PFNDCVGKMLIWWEEGKMTAKIVESAKAILGGSKVRVDQKCKNSIQVDPTPVIITSNVDMTLVIDGNTITAE
HREPLEHRMWKFTFEHQLEPTWGKICKEEVLDFFCWAA-ERPVEVAPTYCVP----------KVAGGGVLPP
PEVDLTEDMDDEDIPAAQQPIEVDF--------------ALSTEIDGQQVVVSGPPPSPE------------
--------PGTRSVGTST---EE-PAPLLSTTPPAESATLKSPPRSPDPQE------GPSSLIAPPAPKKSR
YGTRLVCLEHNQ------EECEIC-GEE---------------------------LCLFTAGFPTPETEDSS
SSTSSVD-LQCHETLS--DMSGLSD------------------VSPPSPPTPDPPV----------------
---------------------------------------------GSPVP----------------------
-----------------------------LIDLFDDMDYMPPFRYQPE
```

```
>SnakeParvo1|NC_006148.1_cds_YP_068093.1_1/1-562
-----------------------------------------------------------MAFYEVVFRLPRD
NNNLL--DEDRY---------QPELKE----EDDWPEEYLT--------SEDASFIGLAYAVLSEIRRFFG-
-----------KELQWFAQVEWCPTAGYHMHVLLNHPKLSNQTYGRKVNELACRI--VDTFGLINPE---EV
ISTHYVKSN--YGHKKVRVIHLESYLKNYFFRK-TLAPPNYTEEGDYKREEEVVLWAFTNIVAW---KPFVR
NLIKRSELA--------------------------TVPKQPEN---PAGDGPAPRV--TAGTRHFM------
ETIDWLVKHGITTEREFCHANRPLYLSMLASTSGAGQIKRALDQAKHMMTSTMSAEDYLTTEEDVIEP-PT-
ENRIYKIMKLNRYDPELAAALFYGWTCKNFGKRNTIW-LYGPATTGKTIIAQAIAHAV-KLFAGVNWTNENF
PFCNCPGKLLIWWEEGKMTNKMVETAKCILGGSAVPVDIKGKPAEMCPQTPCIITSNTNMCQVYDGNSSSFE
HQEPLEERMFMFRLNTKLPSTFGKITEEEVKQFITWGR-SLKVQVPHQFRVP----------TTGEY-----
-----------------KRP-----------------------------------APEA-------------
----------KAHSSDEPP-KEK-VAR----------------------------------------IDDSL
TRY-----------------VNN--IDE-------------SATSREMFLEIANTNQCMLHHC---------
--------FSCTECYP--------------------------------------------------------
------------------------------------------------------------------------
----------------------------EL---LDDMDKEQ-------
>BeardedDragon|NC_027429.1_cds_YP_009154712.1_1/1-565
------------------------------------------------------------MPYEMIFRVPKE
DEDAWCEDNDRY---------EARLAE----GDDWPQERLE--------EDDRWFVNLCYAARGIFKKYFG-
-----------KSLTYFMQVEWSPADGHHMHCVLDDPKLKANNYNYHLDQLSMKM--KTEFKW-KSA---NM
LQKSVQRAA--NRHLKLRYCQFESYIKNYFYKKEVISPEEEQPNGDFERVRDCVLWAFTNLDNW---KPSVR
NIILRNQL---------------------------KAPDQVTT---VPG-GPKPRQ--GANVEAFM------
ETIDWLVKNGITTERQFCQANRTLYLSMLATNSGAGQIKRALEQAKHMMQSTMTAADYLTREERVETC-SE-
TNRIRVIMEKNGYDPLLAANIFNGWLNKEYGKRNTIW-LYGPATTGKTIIAQAIAHGA-VLFGGVNWTNENF
PFCNCPGKLLIWWEEGKMTQKMVETAKCILGGAVVPVDIKGKMAELCETTPVIITSNTNMCQVFDGNSSSFE
HTEPLEERMFMFRLNKKLEPDFGKVTLDEVKEFITWGR-DNPVQVPYQFRVP----------SVATPP-QKS
I-----------NEVLGKRR-----------------------------------AISD-------------
----------GA--GEETR-STK-LVL----------------------------------------LNDSL
TRY-----------------CNNI-TER-------------VNTR-----EIAQNNQCMLHHV---------
--------FNCSECYP--------------------------------------------------------
------------------------------------------------------------------------
----------------------------EL---LDDCDMEQ-------
>SlowLoris|AIZ50117.1/1-582 NS1 [Slow loris parvovirus 1]
--------------MTE-------------DRPDLSPL-------------------NGKGFWELVIKLVNS
PWTENSTLFSKY-DWIEFCD--LEGSD--DPWYDWPEDEI-----------DIYMAILGIKAIKAITRVLRE
RSKN-------KTCNYFGQIEQG-GEFFHIHLLFEVDGFVSFLLGRMFETLRQTL-RNSVYFGYPFE-VSSE
IAITKVKT---GGRNKVQD---GSYIVNYLLKK-------IPPGE--------VQYAISNIECL---RPYCN
SVRNRRALLESVP-------------V-------------------SVERFSEPIIMKGKTVDKFM------
QTLQWCVDEGVTSETIWYKKNPASFRSYQVSAQSRAQAKSILTQAKMEIQISKRLSDYLCREPKENEL-FS-
ENYVSLLFEANGYSASKAAATLARWAAHQSGKRNTIW-LWGPPTTGKTLLASAIANCS-PMFGNVNWNNANF
PFNDCHKQLLIWWEEGSMLQKFVECAKALLGGTSVRVDRKGTDSALVLRTPVIITSNTDMTCVVDGPVKSWE
HKEALEDRMIKYNFERRLPMNLRSITEEEIRQFF-WFG-SCLQCPPLEFLVP-------------------P
DGCD--------SETAYQKLSSLFA---------------------APLGDSTIKTPDL-------------
----N---------------SSR----------------------------------YIDDGDEGPSERSVK
RRRLSLCSVSTE--------------EA------------------------ASAASCLL------------
--------DLCSGSFS--DGSE-----------------G--------------------------------
------------------------------------------------------------------------
---------------------------GSFREALGN------------
>BatTadarida|UJO02142.1/1-400 Rep [Tadarida brasiliensis associated
dependoparvovirus]
------------------------------------------------------------------------
------------------------------------------------------------------------
------------------------------------------------------------------------
------------------------------------------------------------------------
-----------------------------------------------------------------M------
ALVKWLVDAGIATERQWMQEDADSYLSYNATGASRGQIKSSLENACRIMLSTKRAADYLVGASPPED--VT-
QNRIYRLFALNGYDPAYAGSVLLGWCRSGFGKRNTVW-LFGPATTGKTNLAEAISHAV-PFYGCVNWTNENF
```

PFNDCVDKMIIWWEEGKMTAKVVEAAKAILGGSKVRVDQKCKSSQPIEPTPVIITSNTNMCEVVDGNSTTFE
HRQPLEDRMFRFELTVRLQPTFGKITKTEVKEFLKWAE-INSVEVEPDFVVK----------KNF-------
------------VQSDSGGG------------------------------------VKRA-------------
----------GEPLKGAPS-PKR-VFF-YGSSPTETDA-----AAAPC-----------QPIGTEFDCVNFA
ERYVSKCSKHLS-WSNMRFPCKAC--ER-------------MNADV---------NICQAHGT---------
--------HDCVDCFP-----Q----------------------PL-----PEPCV----------------
---------------VHDMCDASIRDADF---YV--------------------------------------
--------------------------------CTDDLNKEQ-------
>MarsupialAAV1|AZP54391.1/1-428 rep protein, partial [Marsupial adeno-associated virus 1]

---AWTNLEEY---EEILL
NIPARLQLAATAA-ASLPLPSSGRR-----------QAAETQ----SSSGRTAPLY-YGKHTQKYM------
DLVAWLVEEGITSEKQWIVENQESYLSFQATSNGARQIKAALDNASKIMNLTKTAADYLITKHESNFDNIE-
ENRIYKIFDMNGYDPLYAGNILTGWCKREFGKRNTIW-LYGKATTGKTNIAEVISHSV-PFWGCVNWTNENF
PFNDCVDKMIIWWEEGKMTSKVVETAKAILGGAKVRVDQKCKSSVQLDSTPVIITSNTDMCYVVDGNTTTFE
HKQPSQDRMFQFLLMKRLPDDFGKVTREEVRQFFKWAN-ENKVDVTREFTVK-KRAPSESPAK---------
------------TDDDRKRK-----------------------------WNFLKAPPT-------------
---------AEPPHKKRAT-APK-ASF-PFRDRTNEEII---ERDAP----------------TQESDLEFL
KRFGLDLKQSEN--
----------SESNFK-----------------------------------PDEIE----------------
--NC---------
--------------------------------DLDDIQDEQ-------
>CanaryDependo2PROBABLYMISSESCTERM|WOP79082/1-501 replication protein [Canary dependoparvovirus 2]
---MNSSPVP
VSPRTPGASPS-----------SFFPDVSLSSLDSDEDDVDGPLDRATSGSDEPDSGVMSGYEFEFVSRFLS
VCRSVCGSMFRRPFVYFAQLEYGAECGAHVHLMVPVSMCGSQVIARWMKNVSRSM---------QTG-KSNW
GFTWTMAHLR-NGKVRMVD---VHYLFRYLLKK-------VAPE---------CGGRWTSFKVFEPDQEGKV
ADETVKRVMEVYE-------------MWMRECEMIRGEFESK----TKKLASSPTS-IKTTKRQRMCVDVMQ
DLVRWFVNKKVTSMQKWMKVDMDHYIKYQSYSTYRPMIKPAMEMATSILLNNGTLIDFLTGVELGR---LQ-
YNRIEDVFRRNGYDPMLAAALFFKWAKRELGKRNTIL-LYGPPTTGKTVIASAICHVVDPFYGNVNWNNENF
PFNDCVEKMLIWWEEGRITAKNVEAAKCILGGVSCRVDRKGKESIEIRATPVIITSNLDMTAVYEGNTVNFD
HKTALEDRMTCFNLRCRLEHDFGRVTSEEVYDWFAKGDLHSGRDIPDTFNFP--------------------
----

--YM------------------------------

>BankVoleMgAAV2_FRAME3_CORRECT_Cterm_Startnotsure|QHD57625.1/1-50 start of frameshift not sure
----

---------------------------------------F--------------------------------
---CTPS------

--PDVVH
DNHDVTCKHGKH------LFCADC--E--------------FVKQK---------RV--DNKY---------
--------LEC--P-------------------

-----------------------------------DSNKEQ-------
>PygmyChameleon|A0A0G3ZB75|A0A0G3ZB75_9VIRU/1-111 Nonstructural protein (Fragment) OS=Pygmy chameleon parvovirus OX=1670664 PE=4 SV=1

---------------------FGKVTEQEVKEFITWGR-SLDIEVPHQFRVP----------VSGAY-----
-----------------KRP-----------------------------------APEA-------------
----------EAHSSDEQP-KEK-VAR--LDDSL
TRY-----------------ANN--IDK-------------SATGREAFLEIATTNQCMLHHT---------
--------FSCTECYP--

----------------------------EL---VDDLMKEQ-------
>RatAAV1|Q2VJ49|Q2VJ49_9VIRU/1-104 Rep (Fragment) OS=Rat adeno-associated virus 1 OX=341227 PE=4 SV=1
---QVPVP
HVFSVPTCSAAAV---------QKRPT---
----------SPSPPFHGETARD---
-ADRKTRKK-----------------PRYDRPK---------PE----------------------------
---PAK------------D---

QN--
----CLDK---WLEESP------------------------------PPRPSVVHD------LQDSGDTVFD
--FRVV----------R---------

--------------------------E---
--------VEC--P--------------------

-----------------------------------DSNKEQ-------
>AAVp01|FJ688147.1_cds_ACN42943.1_1/1-253

---CVNWTNENF
PFNDCVDKMLIWWEEGKMTNKVVESAKAILGGSRVRVDQKCKSSAQIDATPVIVTSNTNMCIVVDGNSTTFE
HQQPLEDRMFKFELTKRLPPDFGKITKREVKDFFAWAE-ANLVPVTHEFRVP----------K-G-------
------------AEKSLKRP-----------------------------------LSDV-------------
----------TDT-SYKSP-EKR-ARV--SFAPETPDCS--DETADP----------------APPRPIDWT
SRYDCRCDSHAR-VETVDEMCEEC--EY-------------LNRGK---------NGCIPHKM---------
--------NYCQICHD----------------------------VP-----PWL------------------
----------------------KEKVSDV---
-------------------------------VDLDDANKEQ-------
>Serpentine2|ACJ66590.1/1-213 non-structural protein 1, partial [Serpentine adeno-associated virus 2]


```
------------------------------------------------------------------------
------------------------------------------------------------------------
------------------------------------------------------------------------
------------------------------------------------------------------------
PFCNCPGKMLIWWEEGKMTQKIVETAKCILGGTRVPVDVKCKMAEICEGTPVIITSNTNMCQVFDGNSSSFE
HTEPLQERMFKIRLNIKLPSDFGRVTKQEVQDFIRWGS-DHPMEISHVFETP----------KEAPPLIQTP
I----------------KNR-----------------------------------EKEA-------------
----------ETSFEEQPP-SKR-VCL----------------------------------------DSEKQ
VRHTTE-------------ICNNI-VPR-------------ANLL-----ELANTTQCMLHGT---------
--------FTCMECYP--------------------------------------------------------
------------------------------------------------------------------------
----------------------------ELTECVDDVDVEQ-------
>CornSnake|AKM49966.1/1-218 nonstructural protein, partial [Corn snake
parvovirus]
------------------------------------------------------------------------
------------------------------------------------------------------------
------------------------------------------------------------------------
------------------------------------------------------------------------
------------------------------------------------------------------------
------------------------------------------------------------------------
------------------------------------------------------AYTV-KLFAGVNWTNENF
PFCNCPGKFLIWWEEGKMTNKMVETAKCILGGAAVPVDIKGKPAEMCMQTPVIITSNTDMCQVYDGNSSSFE
HTEPLEERIFMFRLNYKLAPNFGKVTEQEVKEFITWGR-GLNIDIPYQFRVP----------TSGSY-----
-----------------KRR-----------------------------------GSED-------------
----------AASFSLEPP-SKKDVNP----------------------------------------QISAA
TKY-----------------VCNL-VDK-------------ASVS-----ELAKTNQCMLHHA---------
--------FECTECYP--------------------------------------------------------
------------------------------------------------------------------------
----------------------------EL---LDDMDKEQ-------
```
