# Supplementary material for: The Adeno-Associated Virus Replication Protein Rep78 Contains a Strictly C-Terminal Sequence Motif Conserved Across Dependoparvoviruses
Source: Viruses. 2024 Nov 12;16(11):1760. doi: 10.3390/v16111760 (PMC11598897; doi:10.3390/v16111760)
Supplement: Supplementary file 1 [file viruses-16-01760-s001.zip › Suppl File S3_Protein from Avian AAV isolate BR_DF12 encoded by a reading frame overlapping that of Rep78 and ending with DDx3EQ.pdf]

Suppl File S3: **putative ‘X protein’ from Avian AAV isolate BR_DF12 encoded by a reading frame overlapping that of Rep78 and ending with a DDx3EQ motif**

The C-terminal DDx3EQ motif is underlined.

```
>AvianAAV (isolate BR_DF12)|NCBI Reference Sequence NC_077033.1, ORF 1195-2043
MRQGLSGRQRPDVRRHRGQQDLQDHENERLRSTRGRGHLLEVVQQRVRQE
EHRVAARAGDHGENEHRRGHRARGALLRVRQLDERDLSLQRLRQQADHLV
GGGENDGEDRRNGQSHPRGVQGACRSEMQRQRGAGTDARDHHEQHQHVLG
DRRQHHHVRAQDAAAREDVQAGADDASEPGLWKNHQKRGAAILQLGRRLR
RRARTGLSGSKDNVASHQKSDDVNRAKSASKSPESSLSLTHSETPLDGTP
TGGRALARLQEVESGEGSDPEGGCWHDDCGGEQ
```
