# Supplementary material for: The Adeno-Associated Virus Replication Protein Rep78 Contains a Strictly C-Terminal Sequence Motif Conserved Across Dependoparvoviruses
Source: Viruses. 2024 Nov 12;16(11):1760. doi: 10.3390/v16111760 (PMC11598897; doi:10.3390/v16111760)
Supplement: Supplementary file 1 [file viruses-16-01760-s001.zip › Suppl File S4 - Multiple sequence alignment of enterovirus 2A proteases that have a Cterminal DDx3EQ motif.pdf]

Suppl File S4 - Multiple sequence alignment of enterovirus 2A proteases that have a Cterminal DDx3EQ motif

```
>EnterovirusD_Proteinase|NP_740416.1/1-143 proteinase 2A [Enterovirus D] | Length: 143 | #Patterns: 1
GGAFVGSYKIINYHLATDEEKERSVYVDWQSDVLVTTVAAHGKHQIARCRCNTGVYY-CKHKNRSYPVCFEG
PGIQWINESDYYPARYQTNTLLAMGPCQPGDCGGLLVCSHGVIGLVTAG-GEGIVAFTDIRNLLWLEDDAME
Q
>AXP08076.2/126-268 polyprotein, partial [Enterovirus D]
GGAFVGSYKIINYHLATDQEKQDAVYVDWQSDILVTTIAAHGKHQIARCKCNTGVYY-CRHKDRSYPVYFEG
PGIQWIEENEYYPARYQTNTLLASGPVEAGDCGGLLVCPHGVIGLVTAG-GNGVVAFTDIRNLLWLEDEAME
Q
>QBA83439.1/867-1009 polyprotein [enterovirus D68]QBA83463.1 polyprotein [enterovirus D68]QBA83469.1 polyprotein
[enterovirus D68]
GGAFVGSFKIINYHLATTEERQSAIYVDWQSDVLVTPIAAHGRHQIARCKCNTGVYY-CRHRDKSYPVCFEG
PGIQWIEQNEYYPARYQTNVLLAAGPAEAGDCGGLLVCPHGVIGLLTAG-GGGIVAFTDIRNLLWLDTDAME
Q
>QTZ20033.1/878-1020 polyprotein, partial [Raccoon dog enterovirus]
GGAHVGPYKIINYHLATDEEKRDACWVDWSRDLLVCLTKSHGTQQIARCSCRCGVYF-CNSANKSYPVCFEG
PGIQYFEKNDYYPERYQSHVLLACGVAEPGDCGGVLACPHGVIGLVTGG-APGVVAFADVRDLLWVEDDAME
Q
>NP_653149.1/876-1018 genome polyprotein [Enterovirus H]AAF85765.1 A2 polyprotein [A-2 plaque virus]
GGVVVGQYKIINYHLATTEDREKEIWSDPRRDLLVCASSVHGNNWIARCACRTGVYW-SRTYGKCFPVCFQG
PGIEKFQASEYYPERYQTHVLLAMGPAQPGDCGGLLCCPHGVIGLLTGG-DEGRVAFADIRDLLWVEDDVME
Q
>YP_009246449.1/870-1011 polyprotein [Enterovirus SEV-gx]AMW87255.1 polyprotein [Enterovirus SEV-gx]
GGAHIGQYKVVNHHLLDPED--DPIWDSPQRDLAVILAPSHGKDVVARCKCRCGIYW-CRSKRKYYPVCFDE
PSFRYFDDNEYYPARFQSHVLLASGFAEPGDCGGILQCPHGVIGLVTGGDGQGTVAFADIRDLLWLEDDAME
Q
>AAS88605.2/126-268 polyprotein, partial [Echovirus E13]
GAVYVGNYRVVNRHLATHNDWQNCVWEDYNRDLLVSTTTAHGCDTIARCLCTAGVYY-CASRNKHYPVTFEG
PGLVEVQESEYYPKRYQSHVLLAAGFSEPGDCGGILRCEHGVIGIVTMG-GEGVVGFADVRDLLWLEDDAME
Q
>AAA42933.1/859-1002 polyprotein, partial [Coxsackievirus B3]
RGSVCGDYRLVNRHSATSADWQNCVWESYNRDLLVSTTTTHGCDIIARCQCTTGVYFLCVQKSKHIPISFEG
PGLVEVQDSEYYPRRYQSHVLLAAGFSEPGDCGGILRCEHGVIGIVTMG-GEGVVGFADIRDLLWLEDDAME
Q
>WBR19480.1/268-410 polyprotein, partial [Coxsackievirus A4]WBR19482.1 polyprotein, partial [Coxsackievirus A4]
GAVYVGNYRVVNRHLATHNDWANLVWEDSSRDLLVSSTTAQGCDTIARCNCQTGVYY-CNSRRKHYPVSFSK
PSLIFVEASEYYPARYQSHLMLAAGYSEPGDCGGILRCQHGVVGIVSTG-GNGLVGFADVRDLLWLDEEAME
```

Q
>ALU66466.1/872-1014 polyprotein [Enterovirus A89]
GAVYVGNYRIVNRHLAIPQDWTNLVWEDVNRDLLISSTTAHGCDTIARCQCTTGVYF-CKSRSKHYPVSFSK
PGLVFVNESMYYPARYQSHVLLAPGHAEPGDCGGILRCQHGVVGIVSTG-GDGLVGFADLRDILWLDDEVME
Q
>ABV25903.1/865-1007 polyprotein [enterovirus A124]
GAIYVGNYRVVNRHLATQQDWDNLVWEDYNRDLLVSQTTAHGCDTIARCDCTTGVYF-CKSMMKHYPVSFQG
PGLVHVKKNEYYPERYQSHVLLATGHSEPGDCGGILRCQHGVIGIVTMG-GDNLVGFADIRDLFWLDDEVME
Q
>QWT72253.1/862-1004 polyprotein [enterovirus A122]
GAVYVCNYRVINRHLATEEDWKNLVWEDYQRDLLVSNTKAHGCDTIARCKCQTGVYF-CKSQNKHYPISFQG
PGLVHVQANEYYPERYQSHVLLANGISKAGDCGGILRCQHGVIGLVTMG-GDGLVGFADIRDLLWLEDDVME
Q
>QOI17237.1/862-1004 polyprotein [enterovirus A122]
GAVYVCNYRIVNRHLATQSDWDNLEWEDYQRDILVSRTTAHGCDKIARCNCTTGVYF-CRSTSKHYPVSFQG
PGLVHVQANEYYPERYQSHVLLANGIAVPGDCGGILRCPHGVIGLVTMG-GDGLVGFADVRDLLWLEDEVME
Q
>AFR77805.1/870-1012 polyprotein [Enterovirus A90]
GAVYVGNYKIVNRHLATKEDWSNLVWEDYNRDLLVTSVCAQGCDIIARCSCKAGVYF-CKSMNKHYPISFQG
PGIVEVQANEFYPHRYQTHVLLGHGTSIPGDCGGILRCQHGVIGLVTMG-GDGLVGFADLRDLFWLDDEAME
Q
>ANN47502.1/871-1013 polyprotein [Enterovirus A121]
GAVYVGNYRIVNRHLATQKDWDNLVWESYERDLLVSTTTAQGCDTIARCKCRHGVYY-CNSMRKHYPVSFGE
PAFIYVDENEFYPARYQSHVISSAGIAVPGDCGGILRCEHGVIGLVTMG-GPNIVGFADIRDLLWLDDEVME
Q
>WBR19531.1/260-402 polyprotein, partial [Enterovirus A]
GAIYVGNYRIVNRHLATKKDWENLVWESYERDLLVSTITAHGCDKIARCKCRAGVYF-CKSMNRHYPVTFGE
PAFFHVDANEYYPERYQSHVISSAGIALPGDCGGILRCEHGVIGIVSIG-GPNLVGFADIRDLLWLDDEVME
Q
>APC23144.1/91-233 polyprotein, partial [enterovirus J103]
GAVYVGNYRIVNRHLANEFDAQSTVWDSYERDLLVSTTTAHGCDTIARCRCNTGVYF-CRSKGKHYPVCFQG
PGLTWIEANEYYPARYQSHVLLASGPAEPGDCGGILRCQHGVIGMVTAG-GNGLVAFADLRDLLWLEDDAME
Q
>AHY21610.1/863-1005 polyprotein [Enterovirus sp.]
GAVYVGNYRIVNRHLANEFDAANTVWESYERDLLVSTTTAHGCDTIARCSCTTGVYF-CRSKGKHYPVSFQG
PGLVEVQANEYYPHRYQSHVLLAHGFSEPGDCGGILRCQHGVIGLVTMG-GDGLVGFADLRDLLWLEDDAME
Q
>YP_003359170.1/8-150 2A [Enterovirus J]

GAVFVGNYKITNLHLASTFDRESEVWSSYERDLIVSSTTAHGCDKLARCTCNTGVYF-CKSANKHFPVCFQG
PGLTFIEANEYYPARYQSHVLLAVGHAQPGDCGGILRCEHGVVGILTAG-GNGLVAFADLRDLLWIEDDAME
Q
>AAL69622.2/853-995 polyprotein [enterovirus A125]
GSVYVGSYKITNLHLATEFDLGSEIWRSYERDLIVSTTTASGCDNIARCNCNSGVYF-CKSKSKHYPVVFQG
PGLVQVGANDYYPDRYQSHVLLGVGPAEAGDCGGILRCQHGVIGILTAG-GDGLVAFADIRDLLWIEDDAME
Q
>QKE11198.1/843-985 polyprotein [Enterovirus J]
GAAYVAGYKVMNRHLATPEDWNNLVWDDYNRDLLVCTSGAQGADYIARCKCNTGVYY-CKSKGKHYPVSFQG
PGLAWIEANEYYPARYQSHVLLANGHAEPGDCGGIIRCIHGVIGLVTAG-GDGVVAFADIRDLLWIEDDCME
Q
>WPN04299.1/8-150 polyprotein, partial [Goat enterovirus]
GAVYVGNYKIMNRHLATQAEWDNLEWEDYNRDLIVSRVNAHGADKLARCNCSAGIYY-CKSRGKHYPVTFEG
PGIQWVDANNYYPGRYQSHMLLGIGFCEPGDCGGILRCQHGVIGIITAG-GPSLVAFADLRDLFWVEHEAME
Q
>ULF99594.1/847-989 MAG: polyprotein [Enterovirus sp.]
GAVYVGNYKIVNRHLATQKDWENLEWEEYNRDVLVSRVNAHGADKLARCRCNAGVYY-CKSRNKYYPVTFEG
PGIQLIDANQYYPEHYRTHVLLGIGPCKPGDCGGILRCQHGVIGFITAG-GPNLVAFADLRDLFWVEHEAME
Q
>WEM32007.1/847-989 polyprotein [Enterovirus F]
GAVYVGNYKIMNRHLAERSDWDNLVWESYERDLLVARVDAHGCDLIARCQCTAGVYY-CKSRMKHYPVIVTP
PSLVQIGASDYYPERYQSHVDLGIGFAEPGDCGGILRCQHGVIGILTAG-GNNMVAFADIRDLLWIEDDVME
Q
>WEM32010.1/840-982 polyprotein [Bovine enterovirus type 2]
GAVCVGSYRILNRHLATQADWENVVWESYERDLLVMRHDAAGSDVIARCNCTTGVYY-CKSRNKHYPVVVTP
PSLVHVDANDYYPERYQSHVLLGIGFAEPGDCGGILRCQHGCMGILTAG-GNNLVAFADLRDLLWIEDDAME
Q
>AKA64452.1/842-984 polyprotein [Dromedary camel enterovirus 19CC]
GAAYVGSYKIMNRHLASHDDWHRLVWESYGRDLLVSRVDAQGCDVIARCDCTTGVYY-CKSRNKHYPVVVTP
PSLAFIDESEYYPARYQSHVTLGVGFAEPGDCGGLLRCQHGVMGILTAG-GESLVAFADIRDLLWIEDDAME
Q
>QSX72499.1/855-997 polyprotein [Bovine enterovirus GX20-1]
GAIYVGNYKIVNRHLATYADWENEVWQSYHRDLLVTRVDAHGCDTIARCSCRTGIYY-CKSRDKHYPIVVTP
PSIFKIEANEYYPERMQTHILLGIGPGEPGDCGGILRCEHGVMGILTVG-GGDLVGFADIRDLLWIEDDAME
Q
>UUB82806.1/840-982 polyprotein [Enterovirus F]
GAIYVGNYKIVNRHLAHEVDWQKHVWDSYNRDLLVTRVDAHGCDKIARCNCRAGVYY-CRSSDKHFPVVVSE
PAVYLVEANEYYPEHYQTHVLLGIGIARPGDCGGILRCQHGVMGILTVG-GNNLVAFADVRDLLWVEDDVME

```
Q
>APC23145.1/16-158 polyprotein, partial [enterovirus A122]
GAVYVCNYKIVNRHLATREDRENLVWEDHRRDLLVSSTKAHGCDVIARCKCQSGVYF-CRSENKYYPVSFQN
PGLVHVRANKCHPERYQSHVLVANGISKASDCGGVLRCQHGVIGIVATS-SDNSVGFADLRDLLWLEDDVVE
Q
>AAL69623.2/857-999 polyprotein [Simian agent 5]
GAVYTCSYKILNRHLASPDDWKNVVWEDWNRDLLVTTVQAHGCHQVARCSCTTGVYY-SKYYNRFYPVSFQG
PGIVQMAANEYYPERAQTHVLLANGPAQPGDCGGLLCCTHGVVGILTAG-GDGLVAFADIRDLLWLDDEPME
Q
>WBR19554.1/270-412 polyprotein, partial [Coxsackievirus A11]
KAVYVAGYKICNYHLATPEDLQNAVSVMWDRDLLVTESRAQGVDTIARCACRTGIYY-CESRRKYYPVSFVG
PTFQYMEANDYYPARYQSHMLIGHGFASPGDCGGILRCQHGVIGIITAG-GEGIVAFSDIRDLHAYEEEAME
Q
>UEU84371.1/893-1035 polyprotein, partial [Coxsackievirus A24]
KAVYVAGYKICNYHLATPEDHDNAVSVLWNRDLLVVESRAQGTDTIARCNCKAGVYY-CESKRKYYPVTITE
PTFQYMEANDYYPARYQTHMLLGHGFAEPGDCGGILRCNHGVIGIITAG-GNGIVAFADIRDLWVYEEEAME
Q
>WPN77717.1/886-1028 polyprotein [Coxsackievirus A21]
KAVYVAGYKICNYHLATPSDHLNAISVLWDRDLMVVESRAQGTDTIARCSCRCGVYY-CESRRKYYPVTFTG
PTFRFMEANDYYPARYQSHMLIGCGFAEPGDCGGILRCTHGVIGIITAG-GEGVVAFADIRDLWVYEEEAME
Q
>AWU65871.1/896-1038 MAG: polyprotein, partial [Enterovirus C96]
KAVYVAGYKICNYHLATQEDMNNAVNIMWNRDLLVTQSRAQGTDTIARCHCKCGVYY-CESQRKYYPITVTG
PTFQFMEANEYFPARYQTHMLLGHGFANPGDCGGILRCNHGVMGIITAG-GNGIVAFADIRDLYMYEEEAME
Q
>AIX97183.1/883-1025 polyprotein [Enterovirus C105]
KAIYVAGYKICNYHLATQEDMEHAVAVMWDRDLMVVESRAQGIDTIARCSCKCGVYY-CESMRKYYPVTVEG
PTFRYMDANEYYPARYQSHMLIGAGFAMPGDCGGILRCTHGVMGLITAG-GEGVVAFADVRDLFAYEEEAME
Q
>QER78690.1/897-1038 polyprotein, partial [Enterovirus C96]
QAVYVAGYKIINYHLATSEDYRRCVRSMWERDLMVVESRAQGTDQIARCTCTTGVYY-CSSRNKHYPITITR
PTFQWMEANDYYPARYQSHMTMGHGFAEPGDCGGILRCQHGVMGLITAG-GNGIVAFADIRDLYV-EEEAME
Q
>ABM54518.1/890-1031 polyprotein [Enterovirus C99]
KGIFVAGYKIINYHLATAEDFRNCVRSMWERDLMVVESKAQGVDQIARCSCKTGVYY-CASRQKYYPITVTN
PTFQWMEKNEYYPGRYQSHMILGHGFAEPGDCGGILRCQHGVMGLITAG-GNGIVAFADVRDLYA-EDEAME
Q
>UBM82730.1/271-412 polyprotein, partial [Coxsackievirus A13]
```

KAVYVAGYKIVNYHLASPEDYRNCVRSTWERDLMVVESKAQGTDLIARCSCKTGVYY-CESRNKFYPITITP
PTFQWMEKNDFYPARYQSHMTIGSGFAAPGDCGGILRCQHGVIGLITAG-GNGLVAFADIRDLYV-EEEAME
Q
>ABM21509.1/893-1034 polyprotein [Coxsackievirus A13]
KGVYVAGYKIINYHLATPEDYRHCVKSMWERDLMVVESRAQGIDTIARCCCKTGVYY-CESRNKHYPITITS
PTFQWMERNDFYPARYQTHLTIGSGFAAPGDCGGIIRCQHGVMGLVTAG-GEGVVAFADIRDLYL-EDEAME
Q
>BAE20393.1/894-1035 polyprotein [Coxsackievirus A18]
KAVYVAGYKIVNYHLATASDFRNCVRSLWQRDLMVVESKAQGIDQIARCSCRCGVYY-CESRNKHYPITISS
PTFQWMEKNDYYPARYQTHITIGSGFAAPGDCGGVLRCQHGVMGLITAG-GEGVVAFADIRDLYV-EDEAME
Q
>WAB71077.1/223-364 polyprotein, partial [Coxsackievirus A13]
KAVYVAGYKIINYHLATAIDYQQCVRSMWERDIMIVESRAQGKDRIARCECRSGVYY-CESRGRHYPIIITR
PTFQWMEKNDFYPARYQSHMTIGHGFAAPGDCGGILRCQHGVMGLITAG-GNGLVAFADIRDLYV-EDEAME
Q
