# Supplementary material for: The Adeno-Associated Virus Replication Protein Rep78 Contains a Strictly C-Terminal Sequence Motif Conserved Across Dependoparvoviruses
Source: Viruses. 2024 Nov 12;16(11):1760. doi: 10.3390/v16111760 (PMC11598897; doi:10.3390/v16111760)
Supplement: Supplementary file 1 [file viruses-16-01760-s001.zip › Suppl File S5 - Multiple sequence alignment of CEP57L1 orthologs that have a Cterminal DDx3EQ motif.pdf]

Suppl File S5 - Multiple sequence alignment of CEP57L1 orthologs that have a Cterminal DDx3EQ motif

```
>Centrosomal_protein_CEP57L1|NP_001077004.1/1-460 centrosomal protein CEP57L1 isoform 1 [Homo sapiens]NP_001258781.1 centrosomal protein CEP57L1 isoform 1 [Homo sapiens]NP_001337581.1 centrosomal protein CEP57L1 isoform 1 [Homo sapiens]NP_001337582.1 centrosomal protein CEP57L1 isoform 1 [Homo sapiens]NP_776191.1 centrosomal protein CEP57L1 isoform 1 [Homo sapiens]Q8IYX8.1 RecName: Full=Centrosomal protein CEP57L1; AltName: Full=Centrosomal protein 57kDa-like protein 1; AltName: Full=Centrosomal protein of 57 kDa-related protein; Short=Cep57R; AltName: Full=Cep57-related protein [Homo sapiens]ADQ32441.1 chromosome 6 open reading frame 182, partial [synthetic construct]SJX39480.1 unnamed protein product, partial [Human ORFeome Gateway entry vector]AAH33448.1 Chromosome 6 open reading frame 182 [Homo sapiens]AIC53592.1 CEP57L1 [synthetic construct]EAW48366.1 chromosome 6 open reading frame 182, isoform CRA_f [Homo sapiens]
------------------------------------------------------------------------
------------------------------------------------------------------------
------------------------------------------------------------------------
------------------------------------------------------------------------
-----------------------------------------------MDSELMHSIV----GSYHKPP----
-E-------RV-FVPSFTQNEPS------QNCH--PA-----NLEVTSPKILHSPN-------SQ-------
-------ALILALKTLQEKIHRLELERTQAEDNLNILSREAAQYKKALENETNERNLAHQELIKQKKDISIQ
LSSAQSRCTLLEKQLEYTKRMVLNVEREKNMILEQQ------------------------------------
-----------AQLQREKEQDQMKLYAKLEKLDVLEKECFRLTTTQKTAEDKIKHLEEKLKEEEHQRKLFQD
KASELQTGLEISKIIMSSVSNLKHSKE---KKKSSK--------------------------KTKCIKRRPP
WQICSKFGALPFVAE-------K-------------------------MRQHRD-P--HILQKPFNVTETRC
LPK----PSRT-TS-WC-KAIPPDSEKSISICDNLSELLMAMQDELDQMSMEHQE---LLKQMKETESHSVC
DDIECELECLLKKMEIKGEQISKLKKHQDSVC--KLQQKVQNSKM-SEASGIQQEDSYPKGSKNIKNS--PR
KCLTDTNLFQK-NSSFHPIR-VHNLQMKLRRDDIMWEQ
>BAF82599.1/1-460 unnamed protein product [Homo sapiens]
------------------------------------------------------------------------
------------------------------------------------------------------------
------------------------------------------------------------------------
------------------------------------------------------------------------
-----------------------------------------------MDSELMHSIV----GSYHKPP----
-E-------RV-FVPSFTQNEPS------QNCH--PA-----NLEVTSPKILHSPN-------SQ-------
-------ALILALKTLQEKIHRLELERTQAEDNLNILSREAARYKKALENETNERNLAHQELIKQKKDISIQ
LSSAQSRCTLLEKQLEYTKRMVLNVEREKNMILEQQ------------------------------------
-----------AQLQREKEQDQMKLYAKLEKLDVLEKECFRLTTTQKTAEDKIKHLEEKLKEEEHQRKLFQD
KASELQTGLEISKIIMSSVSNLKHSKE---KKKSSK--------------------------KTKCIKRRPP
WQICSKFGALPFVAE-------K-------------------------MRQHRD-P--HILQKPFNVTETRC
LPK----PSRT-TS-RC-KAIPPDSEKSISICDNLSELLMAMQDELDQMSMEHQE---LLKQMKETESHSVC
DDIECELECLLKKMEIKGEQISKLKKHQDSVC--KLQQKVQNSKM-SEASGIQQEDSYPKGSKNIKNS--PR
KCLTDTNLFQK-NSSFHPIR-VHNLQMKLRRDDIMWEQ
>XP_055246713.1/1-463 centrosomal protein CEP57L1 isoform X2 [Gorilla gorilla gorilla]
------------------------------------------------------------------------
------------------------------------------------------------------------
------------------------------------------------------------------------
----------------------------------------------------------------MKI-----
-----------------------------------------------MDSELMHSIV----GSYHKPP----
-E-------RV-FVPSFTQNEPS------QNCH--PA-----NLEVTSPKILHSPN-------SQ-------
-------ALILALKTLQEKIHRLELERTQAEDNLNILSREAAQYKKALENETNERNLAHQELIKQKKDISIQ
LSSAQSRCTLLEKQLEYTKRMVLNVEREKNMILEQQ------------------------------------
-----------AQLQREKEQDQMKLYAKLEKLDVLEKECFRLTTTQKTAEDKIKHLEEKLKEEEHQRKLFQD
KASELQTGLEISKIIMSSVSNLKHSKQ---KKKSSK--------------------------KTKCIKRGPP
WQICSKFGALPFVAE-------K-------------------------MRQHRD-P--HILQKPFNVTETRC
LPK----PSRT-TF-WC-KAIPPDSEKSISICDNLSELLMAMQDELDQMSMEHQE---LLKQMKETESHSVC
DDIECELECLLKKMEIKGEQISKLKKHQDSVC--KLQQKVQNSKM-SEASGIQQEDSYPKGSKNIKNS--PR
KCLTDTNLFQK-NSSFHPIR-VHNLQMKLRRDDIMWEQ
```

>XP_003255595.2/1-460 centrosomal protein CEP57L1 isoform X1 [Nomascus leucogenys]XP_030665399.1 centrosomal protein CEP57L1 isoform X1 [Nomascus leucogenys]

```
------------------------------------------------------------------------
------------------------------------------------------------------------
------------------------------------------------------------------------
------------------------------------------------------------------------
-----------------------------------------------MDSELMHSIV----GSYHKPP----
-E-------RV-FVPSLIQNEPS------QNCH--PA-----NLEVTSPKILHSPN-------SQ-------
-------ALILALKTLQEKIHRLELERTQAEDNLNILSREAAQYKKALENETNERNLAHQELIKQKKDISIQ
LSSAQSRCTLLEKQLEYTKRMVLNVEREKNMILEQQ------------------------------------
-----------AQLQREKEQDQMKLYAKLEKLDVLEKECFRLTTTQKTAEDKIKHLEEKLKEEEHQRKLFQD
KASELQTGLEISKIIMSSVSNLKHSKE---KKKSSK--------------------------KTKCIKRGPP
WQICSKFGALPFVAE-------K-------------------------MRQHRD-P--HILQKPFNVTETRC
LPK----PSRT-TS-WC-KAIPPDSEKSISICDNLSELLMAMQDELDQMSMEHQE---LLKQMKKTESHSVC
DDIECELECLLKKMEIKGEQISKLKKHQDSVR--KLLQKVQNSKM-SEASGIQQEDSYPRGSKNIKNI--PR
KCLTDTNLFQK-NSSSHPIR-IHNLQMKLRRDDIMWEQ
```

>XP_032012140.1/1-460 centrosomal protein CEP57L1 isoform X1 [Hylobates moloch]XP_058291440.1 centrosomal protein CEP57L1 isoform X1 [Hylobates moloch]XP_058291441.1 centrosomal protein CEP57L1 isoform X1 [Hylobates moloch]XP_058291442.1 centrosomal protein CEP57L1 isoform X1 [Hylobates moloch]

```
------------------------------------------------------------------------
------------------------------------------------------------------------
------------------------------------------------------------------------
------------------------------------------------------------------------
-----------------------------------------------MDSELMHSIV----GSYHKPP----
-E-------RV-FVPSFIQNEPS------QNCH--PA-----NLEVTSPKILHSPN-------SQ-------
-------ALILALKTLQEKIHRLELERTQAEDNLNILSREAAQYKKALENETNERNLAHQELIKQKKDISIQ
LSSAQSRCTLLEKQLEYTKRMVLNVEREKNMILEQQ------------------------------------
-----------AQLQREKEQDQMKLYAKLEKLDVLEKECFRLTTTQKTAEDKIKHLEEKLKEEEHQRKLFQD
KASELQTGLEISKIIMSSVSNLKHSKE---KKKSSK--------------------------KTKCIKRGPP
WQICSKFGALPFVAE-------K-------------------------MRQHRD-P--HILQKPFNVTETRC
LPK----PSRT-TF-WC-KAIPPDSEKSISICDNLSELLMAMQDELDQMSMEHQE---LLKQMKKTESHSVC
DDIECELECLLKKMEIKGEQISKVKKHQDSVR--KLLQKVQNSKM-SEASGIQQEDSYPRGSKNIKSI--PR
KCLTDTNLFQK-NSSSHPIR-VHNLQMKLRRDDIMWEQ
```

>EHH53476.1/1-460 hypothetical protein EGM_14123 [Macaca fascicularis]

```
------------------------------------------------------------------------
------------------------------------------------------------------------
------------------------------------------------------------------------
------------------------------------------------------------------------
-----------------------------------------------MDSELMHSIV----GSYHKPP----
-E-------RV-FVPSFTQNEPS------QNCH--PA-----NLEVTSSKILHSPN-------SQ-------
-------ALILALKTLQEKIHHLELERTQAEDNLNILSREAAQYKKALENETNERNLAHQELIKQKKDISIQ
LSSAQSRCTLLEKQLEYTKRMVLNVEREKNMILEQQ------------------------------------
-----------AQLQREKEQDQIKLYAKLEKLDVLEKECFRLTTTQKTAEDKIKHLEEKLKEEEHQRKLFQD
KASELQTGLEISKIIMSSVSNLKHSKE---KKTSSK--------------------------KTKCIKRGPP
WQICSKFGALPFVAE-------K-------------------------RRQHRG-P--HILQKSFNVAETRC
LPR----PSRT-TS-WC-KAIPRDSEKSISICDNLSELLMAMQDELDQMSMEHQE---LLKQMKETESHSVC
DDIECELECLVKKMEIKGEQISKLKKHQDSVR--KLQQKVQNSKM-SEASGIQQEDSNPKGSKNIKNS--PR
KCLTDTKLFQK-NSSFHPIR-VHNLQMKLRRDDIMWEQ
```

>XP_011831335.1/1-463 PREDICTED: centrosomal protein CEP57L1 isoform X1 [Mandrillus leucophaeus]

```
------------------------------------------------------------------------
------------------------------------------------------------------------
------------------------------------------------------------------------
----------------------------------------------------------------MKI-----
-----------------------------------------------MDSELMHSIV----GSYHKPP----
-E-------RV-FVPSFTQNEPS------QNCH--PA-----NLEVTSSKILHSPN-------SQ-------
-------ALILALKTLQEKIHHLELERTQAEDNLNILSREAAQYKKALENETNERNLAHQELIKQKKDISIQ
LSSAQSRCTLLEKQLEYTKRMVLNVEREKNMILEQQ------------------------------------
-----------AQLQREKEQDQIKLYAKLEKLDVLEKECFRLTTTQKTAEDKIKHLEEKLKEEEHQRKLFQD
```

```
KASELQTGLEISKIIMSSVSNLKHSKE---KKTSSK--------------------------KTKCIKRGPP
WRICSKFGALPFVAE-------K-------------------------RRQHRG-P--HILQKPFNVAETRC
LPR----PSRT-TS-WC-KAIPRDSEKSISICDNLSELLMAMQDELDQMSMEHQE---LLKQMKETESHSVC
DDIECELECLVKKMEIKGEQISKLKKHQDSVR--KLQQKVQNSKM-SEASGIQQEDSNRKGSKNIKNS--PR
KCLTDTKLFQK-NSSFHPIR-VHNLQMKLRRDDIMWEQ
>XP_011808205.1/1-463 PREDICTED: centrosomal protein CEP57L1 isoform X1
[Colobus angolensis palliatus]
------------------------------------------------------------------------
------------------------------------------------------------------------
------------------------------------------------------------------------
----------------------------------------------------------------MKI-----
-----------------------------------------------MDSELMHSIV----GSYHKPP----
-E-------RI-FVPSFTQNEPS------QNCH--PA-----NLEVTSPKILHSPN-------SQ-------
-------ALILALKTLQEKIHRLELERTQAEDNLNILSREAAQYKKALENETNERNLAHQELIKQKKDISIQ
LSSAQSRCTLLEKQLEYTKRMVLNVEREKNMILEQQ------------------------------------
-----------AQLQREKEQDQMKLYAKLEKLDVLEKECFRLTTTQKTAEDKIKHLEEKLKEEEHQRKLFQD
KASELQTGLEISKIIMSSVSNLKHSKE---KKTSSK--------------------------KTKCIKRGPP
WQICSKFGALPFVAE-------K-------------------------RRQHRG-P--HILQKPFNVAETGC
LPR----PSRT-TS-WC-KAIPPDSEKSISICDNLSELLMAMQDELDQMSMEHQE---LLKQMKETESHSVC
DDIECELECLVKKMEIKGEQISKLKKHQESVR--KLQQKVQNSKM-SEASGIQQEDSNPKGSKNIKHS--LR
KCLTDTSLFQK-NRSFHPIR-VHNLQMKLRRDDIMWEQ
>XP_054413950.1/1-490 centrosomal protein CEP57L1 isoform X4 [Pongo abelii]
------------------------------------------------------------------------
------------------------------------------------------------------------
------------------------------------------------------------------------
----------------MEEWYNAT--------------------------------------ELSKI-----
-----------------------------------------------MDSELMHSIV----GSYHKPP----
-E-------RV-FVQSFTQNEPS------QNCH--PA-----NLEVTSPKILHSPN-------SQ-------
-------ALILALKTLQEKIHRLELERTQAEDNLNILSREAAQYKKALENETNERNLAHQELIKQKKDISIQ
LSSAQSRCTLLEKQLEYTKRMVLNVEREKNMILEQQ------------------------------------
-----------AQLQREKEQDQMKLYAKLEKLDVLEKECFRLTTTQKTAEDKIKHLEEKLKEEEHQRKLFQD
KASELQTGLEISKIIMSSVSNLKHFKE---RKKSSK--------------------------KTKCIKRGPP
WQICSKFGALPFVAE-------K------STSAR-CSVNASM-QNFLQMRQHRD-P--HILQKPFNVTETRC
LPK----PSRT-TS-WC-KAIPPDSEKSISICDNLSELLMAMQDELDQMSIEHQE---LLKQMKETESHSVC
DDIECELECLLKKMEIKGEQISKLKKHQDSVR--KLQQKVQNSKM-SEASGIQQEDSYPKGSKNIKNS--PR
KCLTDTNLFQK-NSSFHPIR-VHNLQMKLRRDDIMWEQ
>XP_054413949.1/1-492 centrosomal protein CEP57L1 isoform X3 [Pongo abelii]
------------------------------------------------------------------------
------------------------------------------------------------------------
----------------------------------------------------MIHL-P--------------
-------Q-PLKVLG-------------------------------------------------LEI-----
-----------------------------------------------MDSELMHSIV----GSYHKPP----
-E-------RV-FVQSFTQNEPS------QNCH--PA-----NLEVTSPKILHSPN-------SQ-------
-------ALILALKTLQEKIHRLELERTQAEDNLNILSREAAQYKKALENETNERNLAHQELIKQKKDISIQ
LSSAQSRCTLLEKQLEYTKRMVLNVEREKNMILEQQ------------------------------------
-----------AQLQREKEQDQMKLYAKLEKLDVLEKECFRLTTTQKTAEDKIKHLEEKLKEEEHQRKLFQD
KASELQTGLEISKIIMSSVSNLKHFKE---RKKSSK--------------------------KTKCIKRGPP
WQICSKFGALPFVAE-------K------STSAR-CSVNASM-QNFLQMRQHRD-P--HILQKPFNVTETRC
LPK----PSRT-TS-WC-KAIPPDSEKSISICDNLSELLMAMQDELDQMSIEHQE---LLKQMKETESHSVC
DDIECELECLLKKMEIKGEQISKLKKHQDSVR--KLQQKVQNSKM-SEASGIQQEDSYPKGSKNIKNS--PR
KCLTDTNLFQK-NSSFHPIR-VHNLQMKLRRDDIMWEQ
>KAI4019342.1/1-477 centrosomal protein 57 like 1 [Homo sapiens]
------------------------------------------------------------------------
------------------------------------------------------------------------
------------------------------------------------------------------------
------------------------------------------------------------------------
-----------------------------------------------MDSELMHSIV----GSYHKPP----
-E-------RV-FVPSFTQNEPS------QNCH--PA-----NLEVTSPKILHSPN-------SQ-------
-------ALILALKTLQEKIHRLELERTQAEDNLNILSREAAQYKKALENETNERNLAHQELIKQKKDISIQ
LSSAQSRCTLLEKQLEYTKRMVLNVEREKNMILEQQ------------------------------------
-----------AQLQREKEQDQMKLYAKLEKLDVLEKECFRLTTTQKTAEDKIKHLEEKLKEEEHQRKLFQD
```

KASELQTGLEISKIIMSSVSNLKHSKE---KKKSSK-------------------------KTKCIKRRPP
WQICSKFGALPFVAE-------K------STSAS-CSVNASM-QNFLQMRQHRD-P--HILQKPFNVTETRC
LPK----PSRT-TS-RC-KAIPPDSEKSISICDNLSELLMAMQDELDQMSMEHQE---LLKQMKETESHSVC
DDIECELECLLKKMEIKGEQISKLKKHQDSVC--KLQQKVQNSKM-SEASGIQQEDSYPKGSKNIKNS--PR
KCLTDTNLFQK-NSSFHPIR-VHNLQMKLRRDDIMWEQ
>NP_001337585.1/1-477 centrosomal protein CEP57L1 isoform 9 [Homo sapiens]KAI2543356.1 centrosomal protein 57 like 1 [Homo sapiens]

--MDSELMHSIV----GSYHKPP----
-E-------RV-FVPSFTQNEPS------QNCH--PA-----NLEVTSPKILHSPN-------SQ-------
-------ALILALKTLQEKIHRLELERTQAEDNLNILSREAAQYKKALENETNERNLAHQELIKQKKDISIQ
LSSAQSRCTLLEKQLEYTKRMVLNVEREKNMILEQQ------------------------------------
-----------AQLQREKEQDQMKLYAKLEKLDVLEKECFRLTTTQKTAEDKIKHLEEKLKEEEHQRKLFQD
KASELQTGLEISKIIMSSVSNLKHSKE---KKKSSK-------------------------KTKCIKRRPP
WQICSKFGALPFVAE-------K------STSAS-CSVNASM-QNFLQMRQHRD-P--HILQKPFNVTETRC
LPK----PSRT-TS-WC-KAIPPDSEKSISICDNLSELLMAMQDELDQMSMEHQE---LLKQMKETESHSVC
DDIECELECLLKKMEIKGEQISKLKKHQDSVC--KLQQKVQNSKM-SEASGIQQEDSYPKGSKNIKNS--PR
KCLTDTNLFQK-NSSFHPIR-VHNLQMKLRRDDIMWEQ
>XP_055116857.1/1-477 centrosomal protein CEP57L1 isoform X1 [Symphalangus syndactylus]XP_055116872.1 centrosomal protein CEP57L1 isoform X1 [Symphalangus syndactylus]XP_055116878.1 centrosomal protein CEP57L1 isoform X1 [Symphalangus syndactylus]XP_055116888.1 centrosomal protein CEP57L1 isoform X1 [Symphalangus syndactylus]XP_055116896.1 centrosomal protein CEP57L1 isoform X1 [Symphalangus syndactylus]

--MDSELMHSIV----GSYHKPP----
-E-------RV-FVPSFIQNEPS------QNCR--PA-----NLEVTSPKILHSPN-------SQ-------
-------ALILALKTLQEKIHRLELERTQAEDNLNILSREAAQYKKALENETNERNLAHQELIKQKKDISIQ
LSSAQSRCTLLEKQLEYTKRMVLNVEREKNMILEQQ------------------------------------
-----------AQLQREKEQDQMKLYAKLEKLDVLEKECFRLTTTQKTAEDKIKHLEEKLKEEEHQRKLFQD
KASELQTGLEISKIIMSSVSNLKHSKE---KKKSSK-------------------------KTKCIKRGPP
WQICSKFGALPFVAE-------K------STSAS-CSVNASM-QNFLQMRQHRD-P--HILQKPFNVTETRC
LPK----PSRT-TS-WC-KAIPPDSEKSISICDNLSELLMAMQDELDQMSMEHQE---LLKQMKKTESHSVC
DDIECELECLLKKMEIKGEQISKLKKHQDSVR--KLLQKVQNSKM-SEASGIQQEDSYPRGSKNIKNI--PR
KCLTDTNLFQK-NSSSHPIR-VHNLQMKLRRDDIMWEQ
>XP_030784049.1/1-492 centrosomal protein CEP57L1 isoform X1 [Rhinopithecus roxellana]

---MP---------------
-------R-RRS----LSPVLE--SQI-----
--MDSELMHSIV----GSYHKPP----
-E-------RV-FVPSFTQNEPS------QNCH--PA-----NLEVTSPKILHSPN-------SQ-------
-------ALILALKTLQEKIHRLELERTQAEDNLNILSRETAQYKKALENETNERNLAHQELIKQKKDISIQ
LSSAQSRCTLLEKQLEYTKRMVLNVEREKNMILEQQ------------------------------------
-----------AQLQREKEQDQMKLYAKLEKLDVLEKECFRLTTTQKTAEDKIKHLEEKLKEEEHQRKLFQD
KASELQTGLEISKIIMSSVSNLKHSKQ---KKTSSK-------------------------KTKCIKRGPP
WQICSKFGALPFVAE-------K------FTSAN-CSVNASM-QNLLQRRQHRG-P--HILQKPFNVAETRC
LPR----PSRT-TS-WC-KAIPPDSEKSISISDNLSELLMAMQDELDQMSMEHQE---LLKQMKETESHSVC
DDIECELECLVKKMEIKGEQISKLKKHEESVR--KLQQKVQNSKM-SEASGIQQEDSNPKGSKNIKHS--PR
KCLTDTNLFQK-NSSFHPIR-VHNLQMKLRRDDIMWEQ
>XP_033090695.1/1-492 centrosomal protein CEP57L1 isoform X1 [Trachypithecus francoisi]XP_033090696.1 centrosomal protein CEP57L1 isoform X1 [Trachypithecus francoisi]XP_033090697.1 centrosomal protein CEP57L1 isoform X1 [Trachypithecus francoisi]

--MP--------------
-------R-RRS----LSPVLE--SQI-----
---MDSELMHSIV----GSYHKPP----
-E-------RV-FVPSFTQNEPS------QNCH--PA-----NLEVTSPKILHSPN-------SQ-------
-------ALILALKTLQEKIHRLELERTQAEDNLNILSREAAQYKKALENETNERNLAHQELIKQKKDISIQ
LSSAQSRCTLLEKQLEYTKRMVLNVEREKNMILEQQ------------------------------------
-----------AQLQREKEQDQMKLYAKLEKLDVLEKECFRLTTTQKTAEDKIKHLEEKLKEEEHQRKLFQD
KASELQTGLEISKIIMSSVSNLKHSKE---KKTSSK--------------------------KTKCIKRGPP
WQICSKFGALPFVAE-------K------STSAN-CSVNASM-QNLLQRRQHRG-P--HILQKPFNVAETRC
LPR----PSRT-SS-WC-KAIPPDSEKSISISDNLSELLMAMQDELDQMSMEHQE---LLKQMKETESHSVC
DDIECELECLVKKMEIKGEQISKLKKHQESVR--KLQQKVQNSKM-SEASGIQQEDSNPKGSKNIKHS--PR
KCLTDTNLFQK-NSSFHPIR-VHNLQMKLRRDDIMWEQ
>XP_011757193.1/1-477 centrosomal protein CEP57L1 isoform X1 [Macaca nemestrina]XP_011757194.1 centrosomal protein CEP57L1 isoform X1 [Macaca nemestrina]XP_011757195.1 centrosomal protein CEP57L1 isoform X1 [Macaca nemestrina]

---MDSELMHSIV----GSYHKPP----
-E-------RV-FVPSFTQNEPS------QNCH--PA-----NLEVTSSKILHSPN-------SQ-------
-------ALILALKTLQEKIHHLELERTQAEDNLNILSREAAQYKKALENETNERNLAHQELIKQKKDISIQ
LSSAQSRCTLLEKQLEYTKRMVLNVEREKNMILEQQ------------------------------------
-----------AQLQREKEQDQIKLYAKLEKLDVLEKECFRLTTTQKTAEDKIKHLEEKLKEEEHQRKLFQD
KASELQTGLEISKIIMSSVSNLKHSKE---KKTSSK--------------------------KTKCIKRGPP
WQICSKFGALPFVAE-------K------STSAN-CSVNASM-QNLLQRRQHRG-P--HILQKPFNVAETRC
LPR----PSRT-TS-WC-KAIPRDSEKSISICDNLSELLMAMQDELDQMSMEHQE---LLKQMKETESHSVC
DDIECELECLVKKMEIKGEQISKLKKHQDSVR--KLQQKVQNSKM-SEASGIQQEDSNPKGSKNIKNS--PR
KCLTDTKLFQK-NSSFHPIR-VHNLQMKLRRDDIMWEQ
>XP_025237730.1/1-477 centrosomal protein CEP57L1 isoform X3 [Theropithecus gelada]

---MDSELMHSIV----GSYHKPP----
-E-------RV-FVPSFTQNEPS------QNCH--PA-----NLEVTSSKILHSPN-------SQ-------
-------ALILALKTLQEKIHHLELERTQAEDNLNILSREAAQYKKALENETNERNLAHQELIKQKKDISIQ
LSSAQSRCTLLEKQLEYTKRMVLNVEREKNMILEQQ------------------------------------
-----------AQLQREKEQDQIKLYAKLEKLDVLEKECFRLTTTQKTAEDKIKHLEEKLKEEEHQRKLFQD
KASELQTGLEISKIIMSSVSNLKHSKE---KKTSSK--------------------------KTKCIKRGPP
WQICSKFGALPFVAE-------K------STSAN-CSVNASM-QNLLQRRQHRG-P--HILQKPFNVAETRC
LPR----PSRT-TS-WC-KAIPRDSEKSISIFDNLSELLMAMQDELDQMSMEHQE---LLKQMKETESHSVC
DDIECELECLVKKMEIKGEQISKLKKHQDSVR--KLQQKVQNSKM-SEASGVQQEDSNPKGSKNIKNS--PR
KCLTDTKLFQK-NSSFHPIR-VHNLQMKLRRDDIMWEQ
>XP_045247621.1/1-479 centrosomal protein CEP57L1 isoform X2 [Macaca fascicularis]

---MI-----
---MDSELMHSIV----GSYHKPP----
-E-------RV-FVPSFTQNEPS------QNCH--PA-----NLEVTSSKILHSPN-------SQ-------
-------ALILALKTLQEKIHHLELERTQAEDNLNILSREAAQYKKALENETNERNLAHQELIKQKKDISIQ
LSSAQSRCTLLEKQLEYTKRMVLNVEREKNMILEQQ------------------------------------
-----------AQLQREKEQDQIKLYAKLEKLDVLEKECFRLTTTQKTAEDKIKHLEEKLKEEEHQRKLFQD
KASELQTGLEISKIIMSSVSNLKHSKE---KKTSSK--------------------------KTKCIKRGPP
WQICSKFGALPFVAE-------K------STSAN-CSVSASM-QNLLQRRQHRG-P--HILQKPFNVAETRC

LPR----PSRT-TS-WC-KAIPRDSEKSISICDNLSELLMAMQDELDQMSMEHQE---LLKQMKETESHSVC
DDIECELECLVKKMEIKGEQISKLKKHQDSVR--KLQQKVQNSKM-SEASGIQQEDSNPKGSKNIKNS--PR
KCLTDTKLFQK-NSSFHPIR-VHNLQMKLRRDDIMWEQ
>XP_050645103.1/1-479 centrosomal protein CEP57L1 isoform X2 [Macaca thibetana thibetana]

---MI-----
---MDSELMHSIV----GSYHKPP----
-E-------RV-FVPSFTQNEPS------QNCH--PA-----NLEVTSSKILHSPN-------SQ-------
-------ALILALKTLQEKIHHLELERTQAEDNLNILSREAAQYKKALENETNERNLAHQELIKQKKDISIQ
LSSAQSRCTLLEKQLEYTKRMVLNVEREKNMILEQQ------------------------------------
-----------AQLQREKEQDQIKLYAKLEKLDVLEKECFRLTTTQKTAEDKIKHLEEKLKEEEHQRKLFQD
KASELQTGLEISKIIMSSVSNLKHFKE---KKTSSK-------------------------KTKCIKRGPP
WQICSKFGALPFVAE-------K------STSAN-CSVNASM-QNLLQRRQHRG-P--HILQKPFNVAETRC
LPR----PSRT-TS-WC-KAIPRDSEKSISICDNLSELLMAMQDELDQMSMEHQE---LLKQMKETESHSVC
DDIECELECLVKKMEIKGEQISKLKKHQDSVR--KLQQKVQNSKM-SEASGIQQEDSNPKGSKNIKNS--PR
KCLTDTKLFQK-NSSFHPIR-VHNLQMKLRRDDIMWEQ
>XP_009204582.2/1-487 centrosomal protein CEP57L1 isoform X1 [Papio anubis]XP_011905828.1 PREDICTED: centrosomal protein CEP57L1 isoform X1 [Cercocebus atys]

---MP---------------
-------L-HS------SP--AQI-----
---MDSELMHSIV----GSYHKPP----
-E-------RV-FVPSFTQNEPS------QNCH--PA-----NLEVTSSKILHSPN-------SQ-------
-------ALILALKTLQEKIHHLELERTQAEDNLNILSREAAQYKKALENETNERNLAHQELIKQKKDISIQ
LSSAQSRCTLLEKQLEYTKRMVLNVEREKNMILEQQ------------------------------------
-----------AQLQREKEQDQIKLYAKLEKLDVLEKECFRLTTTQKTAEDKIKHLEEKLKEEEHQRKLFQD
KASELQTGLEISKIIMSSVSNLKHSKE---KKTSSK-------------------------KTKCIKRGPP
WQICSKFGALPFVAE-------K------STSAN-CSVNASM-QNLLQRRQHRG-P--HILQKPFNVAETRC
LPR----PSRT-TS-WC-KAIPRDSEKSISICDNLSELLMAMQDELDQMSMEHQE---LLKQMKETESHSVC
DDIECELECLVKKMEIKGEQISKLKKHQDSVR--KLQQKVQNSKM-SEASGVQQEDSNPKGSKNIKNS--PR
KCLTDTKLFQK-NSSFHPIR-VHNLQMKLRRDDIMWEQ
>XP_028702525.1/1-487 centrosomal protein CEP57L1 isoform X1 [Macaca mulatta]XP_050645102.1 centrosomal protein CEP57L1 isoform X1 [Macaca thibetana thibetana]

---MP---------------
-------L-HS------SL--AQI-----
---MDSELMHSIV----GSYHKPP----
-E-------RV-FVPSFTQNEPS------QNCH--PA-----NLEVTSSKILHSPN-------SQ-------
-------ALILALKTLQEKIHHLELERTQAEDNLNILSREAAQYKKALENETNERNLAHQELIKQKKDISIQ
LSSAQSRCTLLEKQLEYTKRMVLNVEREKNMILEQQ------------------------------------
-----------AQLQREKEQDQIKLYAKLEKLDVLEKECFRLTTTQKTAEDKIKHLEEKLKEEEHQRKLFQD
KASELQTGLEISKIIMSSVSNLKHFKE---KKTSSK-------------------------KTKCIKRGPP
WQICSKFGALPFVAE-------K------STSAN-CSVNASM-QNLLQRRQHRG-P--HILQKPFNVAETRC
LPR----PSRT-TS-WC-KAIPRDSEKSISICDNLSELLMAMQDELDQMSMEHQE---LLKQMKETESHSVC
DDIECELECLVKKMEIKGEQISKLKKHQDSVR--KLQQKVQNSKM-SEASGIQQEDSNPKGSKNIKNS--PR
KCLTDTKLFQK-NSSFHPIR-VHNLQMKLRRDDIMWEQ
>XP_045247620.1/1-487 centrosomal protein CEP57L1 isoform X1 [Macaca fascicularis]

---MP---------------
-------L-HS------SL--AQI-----
---MDSELMHSIV----GSYHKPP----
-E-------RV-FVPSFTQNEPS------QNCH--PA-----NLEVTSSKILHSPN-------SQ-------

-------ALILALKTLQEKIHHLELERTQAEDNLNILSREAAQYKKALENETNERNLAHQELIKQKKDISIQ
LSSAQSRCTLLEKQLEYTKRMVLNVEREKNMILEQQ------------------------------------
-----------AQLQREKEQDQIKLYAKLEKLDVLEKECFRLTTTQKTAEDKIKHLEEKLKEEEHQRKLFQD
KASELQTGLEISKIIMSSVSNLKHSKE---KKTSSK--------------------------KTKCIKRGPP
WQICSKFGALPFVAE-------K------STSAN-CSVSASM-QNLLQRRQHRG-P--HILQKPFNVAETRC
LPR----PSRT-TS-WC-KAIPRDSEKSISICDNLSELLMAMQDELDQMSMEHQE---LLKQMKETESHSVC
DDIECELECLVKKMEIKGEQISKLKKHQDSVR--KLQQKVQNSKM-SEASGIQQEDSNPKGSKNIKNS--PR
KCLTDTKLFQK-NSSFHPIR-VHNLQMKLRRDDIMWEQ
>XP_008005587.1/1-487 centrosomal protein CEP57L1 isoform X1 [Chlorocebus sabaeus]

--MP--------------
-------L-HS------SP---AQI-----
---MDSELMHSIV----GSYHKPP----
-E-------RV-FVPSFTQNEPS------QNCQ--PA-----NLEVTSSKILHSPN-------SQ-------
-------ALILALKTLQEKIHRLELERTQAEDNLNILSREAAQYKKALENETNERNLAHQELIKQKKDISIQ
LSSAQSRCTLLEKQLEYTKRMVLNVEREKNMILEQQ------------------------------------
-----------AQLQREKEQDQIKLYAKLEKLDVLEKECFRLTTTQKTAEDKIKHLEEKLKEEEHQRKLFQD
KASELQTGLEISKIIMSSVSNLKHSKE---KKTSSK--------------------------KTKCIKRGPP
WQICSKFGALPFVAE-------K------STSAN-CSVNASM-QNLLQRRQHRG-P--HILQKAFNVAETRC
LPR----PSRT-TS-WC-KAIPRDSEKSISICDNLSELLMAMQDELDQMSMEHQE---LLKQMKETESHSVC
DDIECELECLVKKMEIKGEQISKLKKHQDSVR--KLQQKVQNSKM-REASGIQQEDSNPKGSKNIKNS--PR
KCLTDTKLFQK-NSSFHPIR-VHNLQMKLRRDDIMWEQ
>NP_001337583.1/1-477 centrosomal protein CEP57L1 isoform 3 [Homo sapiens]NP_001337584.1 centrosomal protein CEP57L1 isoform 3 [Homo sapiens]NP_001337586.1 centrosomal protein CEP57L1 isoform 3 [Homo sapiens]NP_001337587.1 centrosomal protein CEP57L1 isoform 3 [Homo sapiens]

---MDSELMHSIV----GSYHKPP----
-E-------RV-FVPSFTQNEPS------QNCH--PA-----NLEVTSPKILHSPN-------SQ-------
-------ALILALKTLQEKIHRLELERTQAEDNLNILSREAAQYKKALENETNERNLAHQELIKQKKDISIQ
LSSAQSRCTLLEKQLEYTKRMVLNVEREKNMILEQQ------------------------------------
-----------AQLQREKEQDQMKLYAKLEKLDVLEKECFRLTTTQKTAEDKIKHLEEKLKEEEHQRKLFQD
KASELQTGLEISKIIMSSVSNLKHSKE---KKKSSK--------------------------KTKCIKRRPP
WQICSKFGALPFVAE-------K------VRGDK-MKIVVQK-NSCFVMRQHRD-P--HILQKPFNVTETRC
LPK----PSRT-TS-WC-KAIPPDSEKSISICDNLSELLMAMQDELDQMSMEHQE---LLKQMKETESHSVC
DDIECELECLLKKMEIKGEQISKLKKHQDSVC--KLQQKVQNSKM-SEASGIQQEDSYPKGSKNIKNS--PR
KCLTDTNLFQK-NSSFHPIR-VHNLQMKLRRDDIMWEQ
>XP_024213116.1/1-480 centrosomal protein CEP57L1 isoform X10 [Pan troglodytes]

--MKI-----
---MDSELMHSIV----GSYHKPP----
-E-------RV-FVPSFTQNEPS------QNCH--PA-----NLEVTSPKILHSPN-------SQ-------
-------ALILALKTLQEKIRRLELERTQAEDNLNILSREAAQYKKALENETNERNLAHQELIKQKKDISIQ
LSSAQSRCTLLEKQLEYTKRMVLNVEREKNMILEQQ------------------------------------
-----------AQLQREKEQDQMKLYAKLEKLDVLEKECFRLTTTQKTAEDKIKHLEEKLKEEEHQRKLFQD
KASELQTGLEISKIIMSSVSNLKHSKE---KKKSSK--------------------------KTKCIKRRPP
WQICSKFGALPFVAE-------K------VRGDK-MKIVVQK-NSCFVMRQHRD-P--HILQKPFNVTETRC
LPK----PSRT-TS-WC-KAIPPDSEKSISICDNLSELLMAMQDELDQMSMEHQE---LLKQMKETESHSVC
DDIECELECLLKKMEIKGEQISKLKKHQDSVC--KLQQKVQNSKM-SEASGIQQEDSYPKGSKNIKHS--PR
KCLTDTNLFQK-NSSFHPIR-VHNLQMKLRRDDIMWEQ
>XP_054542842.1/1-486 centrosomal protein CEP57L1 isoform X6 [Pan troglodytes]

--MVAFYL----------------

--SQI-----
---MDSELMHSIV----GSYHKPP----
-E-------RV-FVPSFTQNEPS------QNCH--PA-----NLEVTSPKILHSPN-------SQ-------
-------ALILALKTLQEKIRRLELERTQAEDNLNILSREAAQYKKALENETNERNLAHQELIKQKKDISIQ
LSSAQSRCTLLEKQLEYTKRMVLNVEREKNMILEQQ------------------------------------
-----------AQLQREKEQDQMKLYAKLEKLDVLEKECFRLTTTQKTAEDKIKHLEEKLKEEEHQRKLFQD
KASELQTGLEISKIIMSSVSNLKHSKE---KKKSSK--------------------------KTKCIKRRPP
WQICSKFGALPFVAE-------K------VRGDK-MKIVVQK-NSCFVMRQHRD-P--HILQKPFNVTETRC
LPK----PSRT-TS-WC-KAIPPDSEKSISICDNLSELLMAMQDELDQMSMEHQE---LLKQMKETESHSVC
DDIECELECLLKKMEIKGEQISKLKKHQDSVC--KLQQKVQNSKM-SEASGIQQEDSYPKGSKNIKHS--PR
KCLTDTNLFQK-NSSFHPIR-VHNLQMKLRRDDIMWEQ
>XP_025237731.1/1-479 centrosomal protein CEP57L1 isoform X4 [Theropithecus gelada]

---MI-----
---MDSELMHSIV----GSYHKPP----
-E-------RV-FVPSFTQNEPS------QNCH--PA-----NLEVTSSKILHSPN-------SQ-------
-------ALILALKTLQEKIHHLELERTQAEDNLNILSREAAQYKKALENETNERNLAHQELIKQKKDISIQ
LSSAQSRCTLLEKQLEYTKRMVLNVEREKNMILEQQ------------------------------------
-----------AQLQREKEQDQIKLYAKLEKLDVLEKECFRLTTTQKTAEDKIKHLEEKLKEEEHQRKLFQD
KASELQTGLEISKIIMSSVSNLKHSKE---KKTSSK--------------------------KTKCIKRGPP
WQICSKFGALPFVAE-------K------VRGDK-MKIVVQK-NSCFIRRQHRG-P--HILQKPFNVAETRC
LPR----PSRT-TS-WC-KAIPRDSEKSISIFDNLSELLMAMQDELDQMSMEHQE---LLKQMKETESHSVC
DDIECELECLVKKMEIKGEQISKLKKHQDSVR--KLQQKVQNSKM-SEASGVQQEDSNPKGSKNIKNS--PR
KCLTDTKLFQK-NSSFHPIR-VHNLQMKLRRDDIMWEQ
>XP_025237728.1/1-480 centrosomal protein CEP57L1 isoform X1 [Theropithecus gelada]

--MKI-----
---MDSELMHSIV----GSYHKPP----
-E-------RV-FVPSFTQNEPS------QNCH--PA-----NLEVTSSKILHSPN-------SQ-------
-------ALILALKTLQEKIHHLELERTQAEDNLNILSREAAQYKKALENETNERNLAHQELIKQKKDISIQ
LSSAQSRCTLLEKQLEYTKRMVLNVEREKNMILEQQ------------------------------------
-----------AQLQREKEQDQIKLYAKLEKLDVLEKECFRLTTTQKTAEDKIKHLEEKLKEEEHQRKLFQD
KASELQTGLEISKIIMSSVSNLKHSKE---KKTSSK--------------------------KTKCIKRGPP
WQICSKFGALPFVAE-------K------VRGDK-MKIVVQK-NSCFIRRQHRG-P--HILQKPFNVAETRC
LPR----PSRT-TS-WC-KAIPRDSEKSISIFDNLSELLMAMQDELDQMSMEHQE---LLKQMKETESHSVC
DDIECELECLVKKMEIKGEQISKLKKHQDSVR--KLQQKVQNSKM-SEASGVQQEDSNPKGSKNIKNS--PR
KCLTDTKLFQK-NSSFHPIR-VHNLQMKLRRDDIMWEQ
>XP_026310193.1/1-505 centrosomal protein CEP57L1 isoform X1 [Piliocolobus tephrosceles]

---MI-----
---MDSELMHSIV----GSYHKPP----
-E-------RV-FVPSFTQNEPS------QNCH--PA-----NLEVTSPKILHSPN-------SQ-------
-------ALILALKTLQEKIHRLELERTQAEDNLNILSREAAQYKKALENETNERNLAHQELIKQKKDISIQ
LSSAQSRCTLLEKQLEYTKRMVLNVEREKNMILEQQ------------------------------------
-----------AQLQREKEQDQMKLYAKLEKLDVLEKECFRLTTTQKTAEDKIKHLEEKLKEEEHQRKLFQD
KASELQTGLEISKIIMSSVSNLKHSKE---KKTSSKDIWSPAWWLMPVIPTVWEAKASASFEKTKCIKRGPP
WQICSKFGALPFVAE-------K------STSAN-CSVNASM-QNLLQRRQHRG-P--PILQKPFNVAETRC
LPR----PSRT-TS-WC-KAIPPDSEKSISICDNLSELLMAMQDELDQMSMEHQE---LLKQMKETESHSVC
DDIECELECLVKKMEIKGEQISKLKKHQESVC--KLQQKVQNSKM-SEASGIQQEDSNPKGSKNIKHS--PR
KCLTDTNLFQK-NSSFHPIR-VHNLQMKLRRDDIMWEQ

>XP_009450074.2/1-498 centrosomal protein CEP57L1 isoform X5 [Pan troglodytes]XP_054969999.1 centrosomal protein CEP57L1 isoform X1 [Pan paniscus]

---MRDCELGQAL---------------
--PEALSLRP--------------
-------E-AVSVPGRLDSVLEKG--------------------------------------K-EKI-----
---MDSELMHSIV----GSYHKPP----
-E-------RV-FVPSFTQNEPS------QNCH--PA-----NLEVTSPKILHSPN-------SQ-------
-------ALILALKTLQEKIRRLELERTQAEDNLNILSREAAQYKKALENETNERNLAHQELIKQKKDISIQ
LSSAQSRCTLLEKQLEYTKRMVLNVEREKNMILEQQ------------------------------------
-----------AQLQREKEQDQMKLYAKLEKLDVLEKECFRLTTTQKTAEDKIKHLEEKLKEEEHQRKLFQD
KASELQTGLEISKIIMSSVSNLKHSKE---KKKSSK--------------------------KTKCIKRRPP
WQICSKFGALPFVAE-------K------------------------MRQHRD-P--HILQKPFNVTETRC
LPK----PSRT-TS-WC-KAIPPDSEKSISICDNLSELLMAMQDELDQMSMEHQE---LLKQMKETESHSVC
DDIECELECLLKKMEIKGEQISKLKKHQDSVC--KLQQKVQNSKM-SEASGIQQEDSYPKGSKNIKHS--PR
KCLTDTNLFQK-NSSFHPIR-VHNLQMKLRRDDIMWEQ
>XP_018885257.3/1-498 centrosomal protein CEP57L1 isoform X1 [Gorilla gorilla gorilla]

---MRDCELGQAP---------------
--PEALSLRP--------------
-------E-AVSVPGLLDSVLEKG--------------------------------------K-EKI-----
---MDSELMHSIV----GSYHKPP----
-E-------RV-FVPSFTQNEPS------QNCH--PA-----NLEVTSPKILHSPN-------SQ-------
-------ALILALKTLQEKIHRLELERTQAEDNLNILSREAAQYKKALENETNERNLAHQELIKQKKDISIQ
LSSAQSRCTLLEKQLEYTKRMVLNVEREKNMILEQQ------------------------------------
-----------AQLQREKEQDQMKLYAKLEKLDVLEKECFRLTTTQKTAEDKIKHLEEKLKEEEHQRKLFQD
KASELQTGLEISKIIMSSVSNLKHSKQ---KKKSSK--------------------------KTKCIKRGPP
WQICSKFGALPFVAE-------K------------------------MRQHRD-P--HILQKPFNVTETRC
LPK----PSRT-TF-WC-KAIPPDSEKSISICDNLSELLMAMQDELDQMSMEHQE---LLKQMKETESHSVC
DDIECELECLLKKMEIKGEQISKLKKHQDSVC--KLQQKVQNSKM-SEASGIQQEDSYPKGSKNIKNS--PR
KCLTDTNLFQK-NSSFHPIR-VHNLQMKLRRDDIMWEQ
>XP_054413947.1/1-515 centrosomal protein CEP57L1 isoform X1 [Pongo abelii]

---MRDCELGQAP---------------
--PEALSLRP--------------
-------E-AVSVPGRLESVLEKG--------------------------------------K-EKI-----
---MDSELMHSIV----GSYHKPP----
-E-------RV-FVQSFTQNEPS------QNCH--PA-----NLEVTSPKILHSPN-------SQ-------
-------ALILALKTLQEKIHRLELERTQAEDNLNILSREAAQYKKALENETNERNLAHQELIKQKKDISIQ
LSSAQSRCTLLEKQLEYTKRMVLNVEREKNMILEQQ------------------------------------
-----------AQLQREKEQDQMKLYAKLEKLDVLEKECFRLTTTQKTAEDKIKHLEEKLKEEEHQRKLFQD
KASELQTGLEISKIIMSSVSNLKHFKE---RKKSSK--------------------------KTKCIKRGPP
WQICSKFGALPFVAE-------K------STSAR-CSVNASM-QNFLQMRQHRD-P--HILQKPFNVTETRC
LPK----PSRT-TS-WC-KAIPPDSEKSISICDNLSELLMAMQDELDQMSIEHQE---LLKQMKETESHSVC
DDIECELECLLKKMEIKGEQISKLKKHQDSVR--KLQQKVQNSKM-SEASGIQQEDSYPKGSKNIKNS--PR
KCLTDTNLFQK-NSSFHPIR-VHNLQMKLRRDDIMWEQ
>XP_054346002.1/1-515 centrosomal protein CEP57L1 isoform X2 [Pongo pygmaeus]

---MRDCELGQAP---------------
--PEALSLRP--------------
-------E-AVSVPGRLESVLEKG--------------------------------------K-EKI-----
---MDSELMHSIV----GSYHKPP----
-E-------RV-FVHSFTQNEPS------QNCH--PA-----NLEVTSPKILHSPN-------SQ-------
-------ALILALKTLQEKIHRLELERTQAEDNLNILSREAAQYKKALENETNERNLAHQELIKQKKDISIQ
LSSAQSRCTLLEKQLEYTKRMVLNVEREKNMILEQQ------------------------------------
-----------AQLQREKEQDQMKLYAKLEKLDVLEKECFRLTTTQKTAEDKIKHLEEKLKEEEHQRKLFQD
KASELQTGLEISKIIMSSVSNLKHFKE---RKKSSK--------------------------KTKCIQRGPP
WQICSKFGALPFVAE-------K------STSAR-CSVNASM-QNFLQMRQHRD-P--HILQKPFNVTETRC
LPK----PSRT-TS-WC-KAIPPDSEKSISICDNLSELLMAMQDELDQMSIEHQE---LLKQMKETESHSVC

DDIECELECLLKKMEIKGEQISKLKKHQDSVR--KLQQKVQNSKM-SEASGIQQEDSYPKGSKNIKNS--PR
KCLTDTNLFQK-NSSFHPIR-VRNLQMKLRRDDIMWEQ
>XP_008993125.3/1-462 centrosomal protein CEP57L1 isoform X3 [Callithrix jacchus]

---MI-----
---MDSELMHSIV----GSYRKPP----
-E-------RV-FVPSFTKNESS------QNCH--PA-----NLEVTSSKIIHSPN-------SQ-------
-------ALILALKTLQEKIHRLELERTQAEDNLNTLSKEAAQYKKALENETNERNLAHQELIKQKKDISVQ
LSSAQSRCTLLEKQLEYTKRMVLNVEREKNMILEQQ------------------------------------
-----------AQLQREKEQDEMKLYAKLEKLDVLEKECFRLTATQKTAEDKIKYLEERLKEEEHQRKLFQD
KASELQTGLEISKIIMSSVSNLKHSKE---KKKSSK--------------------------KTKCVKRGSP
RQVCSKFGALPFVAE-------K-------------------------MRQHRG-P--HILQKSSNETEPIC
LPK----PSRT-TS-WC-KAIPTDSEKSISICDNLSELLMAMQDELDQMSVEHQE---LLKQMKETESHSVF
NDIECELECLVKKMEIKGTQISKLKKHQDSVR--KLQQKVQNSKM-SKASGIQQEGSNPKGSRNIKNS--PR
KCLTDTNLFQK-NSSFHPIP-VHNLQVKLRRDDIMWEQ
>XP_032130971.1/1-460 centrosomal protein CEP57L1 isoform X1 [Sapajus apella]XP_032130972.1 centrosomal protein CEP57L1 isoform X1 [Sapajus apella]XP_032130973.1 centrosomal protein CEP57L1 isoform X1 [Sapajus apella]XP_032130974.1 centrosomal protein CEP57L1 isoform X1 [Sapajus apella]

---MDSELMHSIV----GSYHKPP----
-E-------RV-FVPSFTQNESS------QNCH--PA-----NLEVTSSKILHSPN-------SP-------
-------ALILALKTLQEKIHRLELERTQAEDNLNILSKEAAQYKKALENETNERNLAHQELIKQKKDISIQ
LSSAQSRCTLLEKQLEYTKRMVLNVEREKNMILEQQ------------------------------------
-----------AQLQREKEQDEMKLYAKLEKLDVLEKECFRLTATQKTAEDKIKYLEERLKEEEHQRKLFQD
KASELQTGLEISKIIMSSVSNLKHSKE---KKKSSK--------------------------KTKCVKRGPP
RQICSKFGALPFVAE-------K-------------------------MRQHRG-P--HILQKSSNETEPRC
LPK----PSRT-TS-WC-KAIPTDSEKSISICDNLSELLMAMQDELDQMSMEHQE---LLKQMKETESHSVC
NDIECELECLVKKMEIKGEQISKLKKHQDSVR--KLQQKVQNSKM-SKASGIQQEGSDLKGSRNIKNS--PR
KCLTDTNLFQK-NSSFHPIR-VHNLQVKLRRDDIMWEQ
>XP_017352877.1/1-460 centrosomal protein CEP57L1 isoform X1 [Cebus imitator]XP_017352878.1 centrosomal protein CEP57L1 isoform X1 [Cebus imitator]XP_017352879.1 centrosomal protein CEP57L1 isoform X1 [Cebus imitator]XP_037583358.1 centrosomal protein CEP57L1 isoform X1 [Cebus imitator]

---MDSKLMHSIV----GSYHKPP----
-E-------RV-FVPSFTQNESS------QNCH--PA-----NLEVTSSKILHSPN-------SP-------
-------ALILALKTLQEKIHRLELERTQAEDNLNILSKEAAQYKKALENETNERNLAHQELIKQKKDISIQ
LSSAQSRCTLLEKQLEYTKRMVLNVEREKNMILEQQ------------------------------------
-----------AQLQREKGQDEMKLYAKLEKLDVLEKECFRLTATQKTAEDKIKYLEERLKEEEHQRKLFQD
KASELQTGLEISKIIMSSVSNLKHSKE---KKKSSK--------------------------KTKCVKRGPP
RQICSKFGALPFVAE-------K-------------------------MRQHRG-P--HILQKSSNETEPRC
LPK----SSRT-TS-WC-KAIPTDSEKSISICDNLSELLMAMQDELDQMSMEHQE---LLKQMKETESHSVC
NDIECELECLVKKMEIKGEQISKLKKHQDSVC--KLQQKVQNSKM-SKASGIQQEGSDLKGSRNIKNS--PR
KCLTDTNLFQK-NSTFHPIR-VHNLQVKLRRDDIMWEQ
>XP_012293822.1/1-462 centrosomal protein CEP57L1 isoform X4 [Aotus nancymaae]

---MI-----
---MDSELMHSIV----GSYHKPP----
-K-------RV-FVPSFTENESS------QNCH--PA-----NLEVTSSKILHSPN-------SP-------

-------ALILALKTLQEKIHRLELERTQAEDNLNTLSIEAARYKKALENETNERNLAHQELMKQKKDISIQ
LSSAQSRCTLLEKQLEYTKRMVLNVEREKNMILEQQ------------------------------------
-----------AQLQREKEQDEMKLYAKLEKLDILEKECFRLTATQKTAEDKIKYLEERLKEEEHQRKLFQD
KASELQTGLEISKIIMSSVSNLKHSKE---KKKSSK-------------------------KTKCVKRGPP
RQICSKFGALPFVAE-------K-------------------------MRQHRG-P--HILQKSSNETEPRC
LPK----PSRT-TS-WC-KAIPTDSEKSISICDNLSELLMAMQDELDQMSMEHQE---LLKQMKETESHSVC
NDIECELECLIKKMEIKGEQISKLKKHQDSVR--KLQQKVQNSKM-SKASGIQQEGSSPKGSRNIKNS--PR
KCLTDTNLFQK-NNSFHPIR-VHNLQVKLRRDDIMWEQ
>XP_039323313.1/1-475 centrosomal protein CEP57L1 isoform X1 [Saimiri boliviensis boliviensis]
------------------------------MICL-------------------PQP---------------
----
--------------PKVL--
---G-LQI-----
---MDSELMHSIV----GSYHKPP----
-E-------RV-FVPSFTQNESS------QNCH--PA-----NLEVTSSKILHSPN-------SP-------
-------ALILALKTLQEKIHRLELERTQAEDNLNTLSKEAAQYKKALENETNERNLAHQELIKQKKDISIQ
LSSAQSRCTLLEKQLEYTKRMVLNVEREKNMILEQQ------------------------------------
-----------AQLQREKEQDEIKLYAKLEKLDVLEKECFRLTATQKTAEDKIKYLEERLKEEEHQRKLFQD
KASELQTGLEISKILMSSVSNLKHSKE---KKKSSK-------------------------KTKCVKRGPP
GQICSKFGALPFVAG-------K-------------------------MRQHRG-P--HILQKSSNETEPRC
LPK----PSRT-TS-WC-KAIHTDSERSISICDNLSELLMAMQDELDQMSMEHQE---LLKQMKETESHSMC
NDIECELECLVKKMEIKGEQISKLKKHQDSVR--KLQQKVQNSKM-SKASGIQQEGSKPKGSRSVKNS--PR
KCLTDTNLFQK-NNSFHPIR-VHNLQVKLRRDDIMWEQ
>XP_015304030.1/1-448 PREDICTED: centrosomal protein CEP57L1 isoform X3 [Macaca fascicularis]

---MDSELMHSIV----GSYHKPP----
-E-------RV-FVPSFTQNEPS------QNCH--PA-----NLEVTSSKILHSPN-------SQ-------
-------ALILALKTLQEKIHHLELERTQAEDNLNILSREAAQYKKALENETNERNLAHQELIKQKKDISIQ
LSSAQSRCTLLEKQLEYTKRMVLNVEREKNMILEQQ------------------------------------
-----------AQLQREKEQDQIKLYAKLEKLDVLEKECFRLTTTQKTAEDKIKHLEEKLKEEEHQRKLFQD
KASE---KTKCIKRGPP
WQICSKFGALPFVAE-------K------STSAN-CSVNASM-QNLLQRRQHRG-P--HILQKSFNVAETRC
LPR----PSRT-TS-WC-KAIPRDSEKSISICDNLSELLMAMQDELDQMSMEHQE---LLKQMKETESHSVC
DDIECELECLVKKMEIKGEQISKLKKHQDSVR--KLQQKVQNSKM-SEASGIQQEDSNPKGSKNIKNS--PR
KCLTDTKLFQK-NSSFHPIQ-VHNLQMKLRRDDIMWEQ
>XP_008575469.1/1-476 PREDICTED: centrosomal protein CEP57L1 isoform X1 [Galeopterus variegatus]

---MDTELMHSIV----GSYLKPP----
-E-------RV-FVPSFTQHESS------QNCH--PV-----NSEVISPKMLHSPN-------SQ-------
-------ALILALKTLQEKIHRLELERTQAEDNLNILSREAAQYKKALEIETNERNLAHEELIKQKKDISIQ
LSSAQSRCTLLEKQLEYTKRMVLNVEREKNMILEQQ------------------------------------
-----------AQLQREKEQDQMKLHAKLEKLDVLEKECFRLTTTQKTAEDKIKHLEEKLKEEEHQRKLFQD
KASELQTGLEISKILMSSVSNPKHSKE---KRKSSK-------------------------KAKCLNRGPP
QQIYSKFGALPFVAE-------K------FASAG-CSVNASM-QSLLQMTQHRG-S--HTLQKP-EVTKPRC
LYK----PTRT-TS-QF-KAVPSDSKVSISICDNLSELLMAMQDELDQMSVEHQE---LLKQMKETESHSVC
DDIECELEHLVKKMEIKGEQISKLKKHQVSVR--KLQQKVQNSKM-SETSGIRQEDSNPKGSKNIKNS--PR
KCLSETNPFQK-NSDFRPIQ-VHNLQVKLRRDDIMWEQ
>XP_012506624.1/1-460 PREDICTED: centrosomal protein CEP57L1 isoform X1 [Propithecus coquereli]

---MNSELMHSVV----GSYLKPP----
-E-------RM-FVPSFTQNESS------QNRQ--PM-----NLEVTSSKVFHSPN-------SQ-------
-------ALILALKTLQEKIHRLELERTQAEDNLNILSREAAQYKKALENETNERNLAHQELIKQKKEISMQ
LSSAQSRCTLLEKQLEYTKRMVLNVEREKNMILEQQ------------------------------------
-----------AQLQREKEQDQMRLHAKLEKLDVLEKECFRLTATQKAAEDKIKHLEEKLTEEEHQRKLFQD
KASELQTGLEINRILMSSVSNPKQSKE---KKKSSK--------------------------KTKCLKRGPP
QQIYSKFGALPFVSE-------K-------------------------MMQHCG-P--HILQKPSEVTEPRC
FYK----PIRK-TS-QC-KAVPSDSEKSVSICDNLSELLMAMQDELDQMSVEHQE---LLKQMKETESYSVC
DDIECELERLVKKMEIKGEQISKLKKHQDSVR--KLQQKVQNSKM-SEASGIQREDIYPKGSKNIKNS--PR
KCLNETNAFQK-NNNFHPIR-VHNLQVKLRRDDIMWEQ
>XP_045400104.1/1-479 centrosomal protein CEP57L1 isoform X2 [Lemur catta]XP_045400105.1 centrosomal protein CEP57L1 isoform X2 [Lemur catta]

---MI-----
---MNSELMHSVV----GSYLKPP----
-E-------RM-VVPSFTQNESS------QNHK--PM-----DLEVTSSKVFHSPN-------SQ-------
-------ALILALKTLQEKIYRLELERTQAEDNLNILSREAAQYKKALENETNERNLAHQELIKQKKEISIQ
LSSAQSRCTLLEKQLEYTKRMVLNVEREKNMILEQQ------------------------------------
-----------AQLQREKEQDQMRLHAKLEKLDVLEKECFRLTTTQKTAEDKIKHLEEKLKEEEHQRKLFQD
KASELQTGLEINRILMSSVSNPKYSKE---KKKSSK--------------------------KTKCLKRGPP
QQIYSKFGALPFVSE-------K------SSSAS-CSVNASV-QNLLQMMQHCG-P--HILQKPSEVTEPRC
LYK----PIRK-TS-LC-KAVPSDSEKSVSICDNLSELLMAMQDELDQMSVEHQE---LLKQMKETESYSVC
DDIECELERLVKKMEIKGEQISKLKKHQASVR--KLQQKVQNSKM-SEASGIQREDIYPKGSKNIKNS--PR
KCLSETNPFQK-NNNFHPVR-VHNLQMKLRRDDIMWEQ
>XP_045400103.1/1-488 centrosomal protein CEP57L1 isoform X1 [Lemur catta]

---MQGM-----------------

-------A-ESV--GLI-----
---MNSELMHSVV----GSYLKPP----
-E-------RM-VVPSFTQNESS------QNHK--PM-----DLEVTSSKVFHSPN-------SQ-------
-------ALILALKTLQEKIYRLELERTQAEDNLNILSREAAQYKKALENETNERNLAHQELIKQKKEISIQ
LSSAQSRCTLLEKQLEYTKRMVLNVEREKNMILEQQ------------------------------------
-----------AQLQREKEQDQMRLHAKLEKLDVLEKECFRLTTTQKTAEDKIKHLEEKLKEEEHQRKLFQD
KASELQTGLEINRILMSSVSNPKYSKE---KKKSSK--------------------------KTKCLKRGPP
QQIYSKFGALPFVSE-------K------SSSAS-CSVNASV-QNLLQMMQHCG-P--HILQKPSEVTEPRC
LYK----PIRK-TS-LC-KAVPSDSEKSVSICDNLSELLMAMQDELDQMSVEHQE---LLKQMKETESYSVC
DDIECELERLVKKMEIKGEQISKLKKHQASVR--KLQQKVQNSKM-SEASGIQREDIYPKGSKNIKNS--PR
KCLSETNPFQK-NNNFHPVR-VHNLQMKLRRDDIMWEQ
>XP_012608546.1/1-488 centrosomal protein CEP57L1 isoform X2 [Microcebus murinus]
--MR----------
---GLTVSVGL-----------------

--I-----
---MNSDLVHSVV----GSYLKPP----
-E-------RM-FVSSFTQNESS------QNRQ--PM-----NLEVTPSKVFHSPN-------SQ-------
-------ALILALKTLQEKIHRLELERTQAEDNLNLLSREAAQYKKALENETNERNLAHQELIKQKKEISVQ
LSSAQSRCTLLEKQLEYTKRMVLNVEREKNMILEQQ------------------------------------
-----------AQLQREKEQDQMRLHAKLEKLDVLEKECFRLTTTQKTAEDKIKHLEEKLTEEEHQRKLFQD
KASELQTGLEINRILMSSVSNPKHSKE---KKKSSK--------------------------KMKCLKRGAP
QQIYSNFGALPFVSE-------K------SSSAS-CSVNASV-QNLLQMMQRCG-P--HILQKPSEVTEPRC
LYK----PIRK-TS-QC-KAIPSDSEKSVSICDNLSELLMALQDELDQMSVEHQE---LLKQMKETESYSVC
DDIECELEHLVKKMEIKGEQISKLKKHQDSVR--KLQQKVQNSKM-SEASGIQREDICPKGSKNIKNS--PR
KCLNETNPFQK-NNNFHPIR-VHNLQVKLRRDDIMWEQ
>XP_012608545.1/1-508 centrosomal protein CEP57L1 isoform X1 [Microcebus murinus]
---MRLQR----------

---HETCAEGRFPF--------------
--PGVWSLWP--------------
-------G-ERE--GKI-----
---MNSDLVHSVV----GSYLKPP----
-E-------RM-FVSSFTQNESS------QNRQ--PM-----NLEVTPSKVFHSPN-------SQ-------
-------ALILALKTLQEKIHRLELERTQAEDNLNLLSREAAQYKKALENETNERNLAHQELIKQKKEISVQ
LSSAQSRCTLLEKQLEYTKRMVLNVEREKNMILEQQ------------------------------------
-----------AQLQREKEQDQMRLHAKLEKLDVLEKECFRLTTTQKTAEDKIKHLEEKLTEEEHQRKLFQD
KASELQTGLEINRILMSSVSNPKHSKE---KKKSSK--------------------------KMKCLKRGAP
QQIYSNFGALPFVSE-------K------SSSAS-CSVNASV-QNLLQMMQRCG-P--HILQKPSEVTEPRC
LYK----PIRK-TS-QC-KAIPSDSEKSVSICDNLSELLMALQDELDQMSVEHQE---LLKQMKETESYSVC
DDIECELEHLVKKMEIKGEQISKLKKHQDSVR--KLQQKVQNSKM-SEASGIQREDICPKGSKNIKNS--PR
KCLNETNPFQK-NNNFHPIR-VHNLQVKLRRDDIMWEQ
>XP_053447335.1/1-474 centrosomal protein CEP57L1 isoform X1 [Nycticebus coucang]XP_053447336.1 centrosomal protein CEP57L1 isoform X1 [Nycticebus coucang]XP_053447338.1 centrosomal protein CEP57L1 isoform X1 [Nycticebus coucang]XP_053447339.1 centrosomal protein CEP57L1 isoform X1 [Nycticebus coucang]XP_053447340.1 centrosomal protein CEP57L1 isoform X1 [Nycticebus coucang]XP_053447341.1 centrosomal protein CEP57L1 isoform X1 [Nycticebus coucang]

---MNSELVHSVV----GSYLKPP----
-E-------RV-FVPSFTQNESS------QTCQ--PK-----NLEVTSAKVFHSPN-------SQ-------
-------ALILALRTLQEKIHRLELERTQAEDNLNTLSKEAAQYKKALENETNERNLAHEELIKQKKDISIQ
LCSAQSRCTLLEKQLEYTKRMVLNVEREKNMILEQQ------------------------------------
-----------AQLQREKEQDQMKLHAKLEKLDVLEKECFRLTTTQKTAEDKIKHLEEKLREEEHQRKLFQD
KASELQTGLEISRILMSSVSNPKHSRE---KKTSSK--------------------------KTKYLKTKPP
WHIHSKLGTLPLVTE-------K------SSSAS-CSVNAGV-QNLMQMMQHCD-L--HSLQKP---SEPSC
LYK----PIRK-TC-QF-KGVPSGSEKSISICDNVSELLMAMQDELDQMTMEHEE---LLKQMKETESYSVC
DDIECELERLVKKMEMKEEQISKLKKHQDSVR--KLQQKVQNSKM-SEASGIQREDINPKGSKNIKNS--PR
KCFNETNPFLK-NDNFHPIR-VHNLQMKLRRDDIMWEQ
>XP_021565199.1/1-482 centrosomal protein CEP57L1 [Carlito syrichta]

--------------------MKN---------------------------------------KLYSV-----
---MDSELMHSVV----GSYLKPP----
-E-------RV-FVPSVTQNETS------QSHH--PV-----NLEVTAPKMIHSPN-------NH-------
-------ALILALKTLQEKIHRLELERTQAEDNLNILSREAAQYKKALENETNERNLAHQELIKQKKDISIQ
LSSAQSRCTLLEKQLEYTKRMVLNVEREKNMILEQQ------------------------------------
-----------AQLQREKEQDQMKLHAKLEKLDVLEKECFRLTTTQKTAEDKIKHLEEKLKEEEHQRKLFQD
KASELQTGLEINRILMSSVSNLKNSKE---KKKSSK--------------------------KTKCLKRGPP
QQNYSKWDALPFVAE-------K------SASAS-CSVNASI-HSLLQMLQHSG-P--HALQKSSRVAKPRC
RYR----PPST-AS-WR-G--PPDTRKPISICDNLSELLIAMQDELDQMHMEYQE---LLEQMKGTESHSVC
DDIECELEHLVKKMEIKGEQISKLKKHQDSVC--KLQQKVQNFEL-SEASGIQREDSNPKGSKNIKNS--PR
KCLTETNPFQK-NRNFHP-R-VHNLQVKLRRDDILWEQ
>XP_036104663.1/1-476 centrosomal protein CEP57L1 isoform X1 [Molossus molossus]XP_036104664.1 centrosomal protein CEP57L1 isoform X1 [Molossus molossus]XP_036104665.1 centrosomal protein CEP57L1 isoform X1 [Molossus molossus]XP_036104666.1 centrosomal protein CEP57L1 isoform X1 [Molossus molossus]KAF6461602.1 centrosomal protein 57 like 1 [Molossus molossus]

---MDSELMHSIV----GSYLKPP----
-E-------RV-FVPSFTQNDEL-----SQNHH--SV-----NFEVTSPKMLHSPN-------SQ-------
-------ALTLALKTLQEKIHRLEVERTQAEDNLNILSIEAAQYKKALEDETNERNLVHQELIKQKKDISIQ

LSSAQSRCTLLEKQLEYTKRMVLNVEREKNMILEQQ------------------------------------
-----------AQLQREKEQDHMKLQAKLEKLDILEKECFKLTTTQKTAEDKIKHLEEKLKEEEHQRKLFQD
KASELQTGLEINRILMSSVSNLKQCKE---KKKSSK-------------------------KTKCLKRGPP
QQIYSKFGSLPVVAE-------K------SSSAR-HSVNASI-QNLLQMMQHCG-P--HIIQKPAEVPEPRC
LYK----PIRT-TS-QC-KALSPDSEKSISICDNLSELLMAMQDELDQMSIEHQE---LLNQMKETESHSVC
ENIEYELEHLVKKMKIKGEQISKLIKHQDSVR--KLQQKVQNSKM-SGASGV-REDSSPTGTKNVKDS--PR
KCWLKSS-LQK-NGNSHPAQ-VHNLQMKLRRDDIMWEQ
>XP_036269216.1/1-477 centrosomal protein CEP57L1 isoform X1 [Pipistrellus kuhlii]KAF6362664.1 centrosomal protein 57 like 1 [Pipistrellus kuhlii]

--MDSELMHSIV----GSYRKPP----
-E-------RM-FAPSFTQNDEL-----SRNHL--SV-----NFEVTSPKKLHSPN-------SQ-------
-------ALILALKTLQEKIHRLELERTQAEDNLNILSREAAQYKKALEDETNERNLVHQELIKQKKDISIQ
LSSAQARCTLLEKQLEYTKRMVLNVEREKNMILEQQ------------------------------------
-----------TQLQREKEQDQMKLQAKLEKLDLLEKECFKLTTTQKTAEDKIKHLEEKLKEEEHQRKLFQD
KASQLQTGLEINRILMSSVSNPNLSKE---KKKSSK-------------------------KTKSLRRGPP
QQMYSKFGSVPMVAE-------K------PARAC-HSVNASV-QNLLQMVQHYG-P--QILQKPAEAAEPRC
LYK----PART-AS-QC-KAVPPDSRTSVSICDNLSELLMEMQEELDQMGVEHQE---LLNQMKETESHTVC
EDIECELEHLVKKMEIKREQISKLMKHQDSVR--KLQQKVQNSKM-REASYIQREDSNPKGLKNIKYS--PR
KCLVNHS-LQK-NSNFHPTQ-VHNLQMKLRRDDIMWEQ
>XP_054578609.1/1-476 centrosomal protein CEP57L1 isoform X1 [Eptesicus fuscus]

--MDSELMHSIV----GSYIKPP----
-E-------RM-FAPSFTQNDEL-----SRNHR--SV-----NFEVTSPKMLHSPN-------SQ-------
-------ALILALKTLQEKIHRLELERTQAEDNLNILSREAAQYKKALEDETNERNLVHQELIKQKKDISIQ
LSSAQARCTLLEKQLEYTKRMVLNVEREKNMILEQQ------------------------------------
-----------TQLQREKEQDQMKLQAKLDKLDLLEKECFKLTTTQKTAEDKIKHLEEKLKEEEHQRKLFQD
KASELQTGLEINRILMSSVSNPKLSKE---KKKSSK-------------------------KTKCLKRGPP
QQMYSKFGSMPMVAE-------K------PARSC-HSVNASV-QNLLQMVQHYG-P--QILQKP-EVAEPRC
LYK----PTRT-AS-QC-KVVPPDSEKSVSICDNLSELLMAMQEELDQMGVEHQE---LLNQMKETESHAVC
EDIECELEHLVKKMEIKREQISKLMKHQDSVR--KLQQKVQNSKM-SEASCIQREDSNPKGLKNIKYS--PR
KCLLNHS-LQK-NSNFHPTQ-VHNLQMKLRRDDIMWEQ
>XP_005883839.1/1-477 PREDICTED: centrosomal protein CEP57L1 [Myotis brandtii]XP_014386864.1 PREDICTED: centrosomal protein CEP57L1 [Myotis brandtii]XP_014386865.1 PREDICTED: centrosomal protein CEP57L1 [Myotis brandtii]

--MDSELMHSIV----GSYIKPP----
-E-------RI-FAPSFTQNDEL-----PRNHH--SV-----NFEVTSPKMLQSPN-------SQ-------
-------ALILALKTLQEKIHRLELERTQAEDNLNTLSREAAQYKKALEDETNERNLVHQELIKQKKDISIQ
LSSAQTRCTLLEKQLEYTKRMVLNVEREKNMILEQQ------------------------------------
-----------TQLQREKEQDQMKLQAKLEKLDLLEKECFKLTTTQKTAEDKIKHLEEKLKEEEHQRKLFQD
KASELQTGLEINRILMSSVSNPKQSKE---KKKSSK-------------------------KTKCLKRGPP
QQMYSKFGSMPMVSE-------K------PARAC-HSVNASV-QNLLQMVQHYG-P--QILQKPAEVAEPRC
LYK----PTRT-TS-QC-KVVPPHSKKSVSICDNLSELLIAMQEELDQMSVEHQE---LLNQMKETESHAVC
EDIECELEHLVKKMEIKREQISKLMKHQDSVR--KLQQKVQNSKM-SEASCIQREDSNPKGLKNIKYS--PR
KCLLNHS-LQK-NSNFHPTP-VHNLQMKLRRDDIMWEQ
>XP_006777265.1/1-477 PREDICTED: centrosomal protein CEP57L1 isoform X1 [Myotis davidii]

--MDSELMHSIV----GSYIKPP----
-E-------RI-FAPSFTQNDEL-----PRNHH--SV-----NFKVTSPKMLQSPN-------SQ-------
-------ALILALKTLQEKIHRLELERTQAEDNLNTLSREAAQYKKALEDETNERNLVHQELIKQKKDISIQ
LSSAQNRCTLLEKQLEYTKRMVLNVEREKNMILEQQ------------------------------------
-----------TQLQREKEQDQMKLQAKLEKLDLLEKECFKLTTTQKTAEDKIKHLEEKLKEEEHQRKLFQD
KASELQTGLEINRILMSSVSNPKQPKE---KKKSSK-------------------------KTKCLKRGPP
QQMYSKFGSMPMVSE-------K------PARAC-HSVNASV-QNLLQMVQHYG-P--QILQKPAEVAEPRC
IYK----PTRT-TS-QC-KVVPPHSKKSVSICDNLSELLIAMQEELDQMSVEHQE---LLNQMKETESHAVF
EDIECELEHLVKKMEIKREQISKLMKHQDSVR--KLQQKVQNSKM-SEASRIQREDSNPKGLKNIKYS--PR
KCLLNHS-LQK-NSNFHPTP-VHNLQMKLRRDDIMWEQ
>XP_059556759.1/1-477 centrosomal protein CEP57L1 isoform X2 [Myotis daubentonii]

--MDSELMHSIV----GSYIKPP----
-E-------RI-FAPSFTQNDEL-----PRNHH--SV-----NFKVTSPKMLQSPN-------SQ-------
-------ALILALKTLQEKIHRLELERTQAEDNLNTLSREAAQYKKALEDETNERNLVHQELIKQKKDISIQ
LSSAQNRCTLLEKQLEYTKRMVLNVEREKNMILEQQ------------------------------------
-----------TQLQREKEQDQMKLQAKLEKLDLLEKECFKLTTTQKTAEDKIKHLEEKLKEEEHQRKLFQD
KASELQTGLEINRILMSSVSNPKQPKE---KKKSSK-------------------------KTKCLKRGPP
QQMYSKFGSMPMVSE-------K------PARAC-HSVNASV-QNLLQMVQHYG-P--QILQKPAEVAEPRC
LYK----PTRT-TS-QC-KVVPPHSKKSVSICDNLSELLIAMQEELDQMSVEHQE---LLNQMKETESHAVF
EDIECELEHLVKKMEIKREQISKLMKHQDSVR--KLQQKVQNSKM-SEASCIQREDSNPKGLKNIKYS--PR
KCLLNHC-LQK-NSNFHPTP-VHNLQMKLRRDDIMWEQ
>XP_036174365.1/1-477 centrosomal protein CEP57L1 isoform X1 [Myotis myotis]XP_036174366.1 centrosomal protein CEP57L1 isoform X1 [Myotis myotis]KAF6341047.1 centrosomal protein 57 like 1 [Myotis myotis]

--MDSELMHSIV----GSYIKPP----
-E-------RI-FAPSFTQNDEL-----PRNHH--SV-----NFKVTSPKMLQSPN-------SQ-------
-------ALILALKTLQEKIHRLELERTQAEDNLNTLSREAAQYKKALEDETNERNLVHQELIKQKKDISIQ
LSSAQNRCTLLEKQLEYTKRMVLNVEREKNMILEQQ------------------------------------
-----------TQLQREKEQDQMKLQAKLEKLDLLEKECFKLTTTQKTAEDKIKHLEEKLKEEEHQRKLFQD
KASELQTGLEINRILMSSVSNPKQPKE---KKKSSK-------------------------KTKCLKRGPP
QQMYSKFGPMPMVSE-------K------PARAC-HSVNASV-QNLLQMVQHYG-P--QILQKPAEVAEPRC
LYK----PTRR-TS-QC-KVVPPHSKKSVSICDNLSELLIAMQEELDQMSVEHQE---LLNQMKETESHAVF
EDIECELEHLVKKMEIKREQISKLMKHQDSVR--KLQQKVQNSKM-SEASCIQREDSNPKGLKNIKYS--PR
KCLLNHS-LQK-NSNFHPTP-VHNLQMKLRRDDIMWEQ
>XP_023620903.1/1-499 centrosomal protein CEP57L1 isoform X2 [Myotis lucifugus]

---M--------------
------LT-PGPDAHCK---SGRIVHT----------DYT--------------------------I-----
--MDSELMHSIV----GSYIKPP----
-E-------RI-FAPSFTQNDEL-----PRNHH--SV-----NFEVTSPKMLQSPN-------SQ-------
-------ALILALKTLQEKIHRLELERTQAEDNLNTLSREAAQYKKALEDETNERNLVHQELIKQKKDISIQ
LSSAQTRCTLLEKQLEYTKRMVLNVEREKNMILEQQ------------------------------------
-----------TQLQREKEQDQMKLQAKLEKLDLLEKECFKLTTTQKTAEDKIKHLEEKLKEEEHQRKLFQD
KASELQTGLEINRILMSSVSNPKQPKE---KKKSSK-------------------------KTKCLKRGPP
QQMYSKFGSMPMLSE-------K------PARAC-HSVNASV-QNLLQMVQHYG-P--QILQKPAEVAEPRC
LYK----PTRT-TS-QC-KVVPPHSKKSVSICDNLSELLIAMQEELDQMSVEHQE---LLNQMKETESHAVC
EDIECELEHLVKKMEIKREQISKLMKHQDSVR--KLQQKVQNSKM-SEASCIQREDSNPKGLKNIKYS--PR
KCLLNHS-LQK-NSNFHPTP-VHNLQMKLRRDDIMWEQ
>XP_054425783.1/1-476 centrosomal protein CEP57L1 [Pteronotus parnellii mesoamericanus]

--MDSELMHSIV----GSYLTPP----
-E-------RV-FVPPFTQNDEL-----SQNHH--SG-----NFEATSSKMLHSPN-------SQ-------
-------ALILALKTLQEKIHRLELERTQAEDNLNILSKEAAQYKKSLENETNERNLVHEELIKQKKDISIQ
LSSAQSRCTLLEKQLEYTKRMVLNVEREKNMILEQQ------------------------------------
-----------AQLQREKEQDQMKLQAKLEKLDVLEKECFKLTTTQKTAEDKIKHLEEKLKEEEHQRKLFQD
KASELQTGLEINRILMSSVSNPKQSKE---KKKSSK-------------------------KTKCLKRRPP
QQTYSKFGSLPIVAE--------------SAGAR-HSVNTSM-QNVLQMMQHYG-S--HILQKPAEVTEPRC
LYK----PTKT-AL-QR-KAIAPDSEKSVSICDNLSELLMAMQDELDQMSIEHQE---LLNQMKETESHSVC
EDIECELEHLVKKMEIKGEQISKLMKHQDSVR--KLQQKVQNSKM-SEASSFQQEDSNPKGSKNIKNS--PR
KCLLNNS-LQK-NSNFHPIQ-VHNLQMKLRRDDIMWEQ
>XP_016073035.1/1-477 PREDICTED: centrosomal protein CEP57L1 [Miniopterus natalensis]XP_016073036.1 PREDICTED: centrosomal protein CEP57L1 [Miniopterus natalensis]XP_016073037.1 PREDICTED: centrosomal protein CEP57L1 [Miniopterus natalensis]

--MDSELMHSIV----GSYLKPP----
-E-------RV-FVPSFTQNDEL-----SQNHH--SV-----NFEVTSPKMLRSPN-------SQ-------
-------ALILALKTLQEKIHRLELERTQAEDNLNILSREAAQYKKALEDETNERNLVHQELIKQKKDISIQ
LSSAQSRCTLLEKQLEYTKRMVLNVEREKNMILEQQ------------------------------------
-----------AQLQREKEQDHMKLQAKLEKLDTLEKECFKLTTTQKTAEDKIKHLEEKLKEEEHQRKLFQD
KASELQTGLEINRILMSSVSNPKRSKE---RKKSSK-------------------------KTKCLKRGPP
QQLYSKSGSLPIVAE-------K------SASAH-HSANTSM-QNLLQMMQHYG-S--HILQKPTEVTEPRC
LYK----PTRT-TS-QC-KVVPPNSEKSVSICDNLSELLMAMQDELDQMSIEHQE---LLNQMKETESHAVS
EDIECELEHLVKKMEIKGEQISKLIKHQDSVR--KLQQKVQNSKM-REASGIQREDSNPKGSQNIKNR--PR
KCLLNNS-LQK-NSNFHPTQ-VHNLQMKLRRDDIMWEQ
>XP_037014924.2/1-476 centrosomal protein CEP57L1 isoform X1 [Artibeus jamaicensis]

--MDSELMHSIV----GSYLKPP----
-E-------RT-FVPPFTQNDEL-----SQNHH--SG-----NFEATSSKMLRSPN-------SQ-------
-------ALILALKTLQEKIRRLELERTQAEDNLNILSREAAEYKKALENETNERNLVHQELIKQKKDISIQ
LSSAQSRCILLEKQLEYTKRMVLNVEREKNMILEQQ------------------------------------
-----------AQLQREKEQDQMKLQAKLEKLDVLEKECFKLTTTQKTAEDKIKHLEEKLKEEEHQRKLFQD
KASELQTGLEVNRILMSSVSNSKRSKE----KKSSK-------------------------KTKCLKREPP
QQIYSKFGSLPIVAE-------K------SATAR-HSVNASM-QNLLHMMQHYG-P--HSLQKPAEVSEPRC
RYK----PTKT-AS-QC-KVVPLDSEKPASICDNLSELLMAMQDELDQMSIEHQE---LLSQMKDTESHSVC
EDIECELEHLIKKMEIKGEQISKLMKHQDNVR--KLQQKFQNSKM-NESSGIQREDRNPKGSKNIKNS--PR
KCLLNNS-LQK-NSNFHPIQ-VHNLQTKLRRDDIMWEQ
>XP_036920904.1/1-477 centrosomal protein CEP57L1 [Sturnira hondurensis]XP_036920905.1 centrosomal protein CEP57L1 [Sturnira hondurensis]

--MDSELMHSIV----GSYLKPP----
-E-------RT-FVPPFTQNDEL-----SQNHH--SG-----NFEATSSKMLRSPN-------SQ-------
-------ALILALKTLQEKIRRLELERTQAEDNLNILSKEAAEYKKALENETSERNLVHQELIKQKKDISIQ
LSSAQSRCILLEKQLEYTKRMVLNVEREKNMILEQQ------------------------------------
-----------AQLQREKEQDQMKLQAKLEKLDVLEKECFKLTTTQKTAEDKIKHLEEKLKEEEHQRKLFQD
KASELQTGLEVNRILMSSVSNSKRSKE---KKKSSK-------------------------KTKCLKREPP
QQIYSKFGSLPIVAE-------K------PASAR-HPVNASV-QNLLHMMQHHG-P--PVLQKPADVSEPRC

RYK----PTKT-TS-QC-KVVPLDSEKSVSICDNLSELLVAMQDELDQMSIEHQE---LLSQMKETESHSVC
EDIECELEHLIKKMEIKGEQISKLMKHQDSVR--KLQQKVQNSKV-NESSSIQREDRNPKGSKNIKNS--PR
KCLLNNS-LQK-NSNFHPIQ-VHNLQTKLRRDDIMWEQ
>XP_045698744.1/1-477 centrosomal protein CEP57L1 [Phyllostomus hastatus]XP_045698745.1 centrosomal protein CEP57L1 [Phyllostomus hastatus]

---MDSELMHSIV----GSYLKPP----
-E-------RA-FVPPFTQNDEL-----SQNHH--SG-----NFEAASSKMLRSPN-------SQ-------
-------ALILALKTLQEKIHRLELERTQAEDNLNILSREAAEYKKALENETNERNLVHQELIKQKKDISIQ
LSSAQSRCILLEKQLEYTKRMVLNVEREKNMILEQQ------------------------------------
-----------AQLQREKEQDQMKLQAKLEKLDVLEKECFKLTTTQKTAEDKIKHLEEKLKEEEHQRKLFQD
KASELQTGLEVNRILMSSVSNPKQSKE---KKKSSK-------------------------KTKCLKREPP
QQIYSKFGSLPIVAE-------K------SAGAR-HSVNASM-QNLLRMMQHYA-P--RILQKPAEATEPRC
LYR----PTRT-TS-QC-KAVPLDSEKSVSICDNLSELLMAMQDELDQMSIEHQE---LLNQMKETESQSVC
EDIECELEHLIKKMEIKGEQISKLMKHQDNVC--KLQQKVQNSKM-NESSSIQREDRNPKGSKNIKNS--PR
KCLLNNS-LQK-NSSFHPIQ-VHNLQMKLRRDDIMWEQ
>KAF6113142.1/1-484 centrosomal protein 57 like 1 [Phyllostomus discolor]
---MSPQL

-------R---I-----
---MDSELMHSIV----GSYLKPP----
-E-------RA-FVPPFTQNDEL-----SQNYH--SG-----NFEAASSKMLRSPN-------SQ-------
-------ALILALKTLQEKIHRLELERTQAEDNLNILSREAAEYKKALENETNERNLVHQELIKQKKDISIQ
LSSAQSRCILLEKQLEYTKRMVLNVEREKNMILEQQ------------------------------------
-----------AQLQREKEQDQMKLQAKLEKLDVLEKECFKLTTTQKTAEDKIKHLEEKLKEEEHQRKLFQD
KASELQTGLEVNRILMSSVSNPKQSKE---KKKSSK-------------------------KTKCLKREPP
QQIYSKFGSLPIVAE-------K------SAGAR-HSVNASM-QNLLRMMQHYA-P--RILQKPAEVTEPSC
LCR----PTRT-TS-QC-KAVPLDSEKSVSICDNLSELLMAMQDELDQMSIEHQE---LLNQMKETESQSVC
EDIECELEHLIKKMEIKGEQISKLMKHQDSVC--KLQQKIQNSKM-NESSSIQREDRNPKGSKNIKNS--PR
KCLLNNS-LQK-NSSFHPIQ-VHNLQMKLRRDDIMWEQ
>XP_045044597.2/1-506 centrosomal protein CEP57L1 [Desmodus rotundus]
---MSRQR

-------R-ATSGPPVRA-QPPTVA-----------------G-----------------CYACVMI-----
---MDSELMHSIV----GSYLKPP----
-E-------RV-FVPPFTQNCEL-----SQNHH--SG-----NFEATSSKMLRSPN-------SQ-------
-------ALILALKTLQEKIHRLELERTQAEDNLNILSKEAAEYKKALENETNERNLVHQELIKQKKDISIQ
LSSAQSRCILLEKQLEYTKRMVLNVEREKNMILEQQ------------------------------------
-----------AQLQREKEQDQMKLQAKLEKLDVLEKECFKLTATQKTAEDKIKHLEEKLKEEEHQRKLFQD
KASELQTGLEINRILMSSVSNPKQSKE---KKKSSK-------------------------KTKCLKKGPP
QQIYSNFGSLPIVAE-------K------SASAS-HSVHASM-QNLLKMMQHYG-P--HILQKPAEVTEPRC
VYK----PTKT-TS-QC-KAVPPDSEKSVSICDNLSELLMAMQDELDQMSIEHQE---LLNQMKETESHSVC
EDIECELEHLIKKMEIKGEQISKLMKHQDSVR--KLEQKVQNSKM-SEASSMQREDRNPKGSKNIKNS--PR
KCLLNNS-PQK-NSNFHPIQ-VHNLQLKLRRDDIMWEQ
>XP_025300257.1/1-474 centrosomal protein CEP57L1 [Canis lupus dingo]XP_025300258.1 centrosomal protein CEP57L1 [Canis lupus dingo]XP_025300259.1 centrosomal protein CEP57L1 [Canis lupus dingo]XP_025300260.1 centrosomal protein CEP57L1 [Canis lupus dingo]XP_035553454.1 centrosomal protein CEP57L1 [Canis lupus dingo]XP_038410815.1 centrosomal protein CEP57L1 [Canis lupus familiaris]XP_038410816.1 centrosomal protein CEP57L1 [Canis lupus familiaris]XP_038410817.1 centrosomal protein CEP57L1 [Canis lupus familiaris]XP_038410818.1 centrosomal protein CEP57L1 [Canis lupus familiaris]XP_038410819.1 centrosomal protein CEP57L1 [Canis lupus familiaris]XP_038410820.1 centrosomal protein CEP57L1 [Canis lupus familiaris]XP_038540252.1 centrosomal protein CEP57L1 [Canis lupus

familiaris]XP_038540253.1 centrosomal protein CEP57L1 [Canis lupus
familiaris]XP_038540254.1 centrosomal protein CEP57L1 [Canis lupus
familiaris]XP_038540255.1 centrosomal protein CEP57L1 [Canis lupus
familiaris]XP_038540256.1 centrosomal protein CEP57L1 [Canis lupus
familiaris]XP_038540257.1 centrosomal protein CEP57L1 [Canis lupus familiaris]

---MDSELMHSIV----GSYLKPP----
-E-------RV-FFPSFTQNNES-----SQTPH--YA-----NFEGTSPKMLHSPN-------SQ-------
-------ALMLALKTLQEKIHRLELERTQAEDNLNILSREAAEYKKALEKETNERNLAHQELIKQKKDITIQ
LSSAQSRCTLLEKQLEYTKRMVLNVEREKNMILEQQ------------------------------------
-----------AQLQREKEQDHMKLQAKLEKLDVLEKECFKLTTTQKTAEDKIKHLEEKLKEEEHQRKLFQD
KASELQTGLEINRILMSSVANPKSSKE---KKKSLK--------------------------KTRCLKERLP
QQIYSKSGSLPIVAK-------K------SSSAS-HSVNTSV-QSLLQVTQHYG-P--HTHQKL---TEPRC
LYK----PTRT-TS-QG-KAEPSDSKHSISIGDNLSELLMAMQDELDQMNMKHEE---LLNQMKETESRSVC
EDLECELEHLVKKMEIKGEQISKLIKHQENVR--KLQQKVQQSKM-SEASGIQREDNNLKGSKNIKNS--SR
KCLLTNS-LQK-NSNFHPVQ-VHNLQMKLRRDDIMWEQ
>XP_005627744.1/1-474 centrosomal protein CEP57L1 [Canis lupus
familiaris]XP_005627745.1 centrosomal protein CEP57L1 [Canis lupus
familiaris]XP_005627747.1 centrosomal protein CEP57L1 [Canis lupus
familiaris]XP_005627748.1 centrosomal protein CEP57L1 [Canis lupus
familiaris]XP_038532483.1 centrosomal protein CEP57L1 [Canis lupus
familiaris]XP_038532486.1 centrosomal protein CEP57L1 [Canis lupus familiaris]

---MDSELMHSIV----GSYLKPP----
-E-------RV-FFPSFTQNNES-----SQTPH--YA-----NFEGTSPKMLHSPN-------SQ-------
-------ALMLALKTLQEKIHRLELERTQAEDNLNILSREAAEYKKALEKETNERNLAHQELIKQKKDITIQ
LSSAQSRCTLLEKQLEYTKRMVLNVEREKNMILEQQ------------------------------------
-----------AQLQREKEQDHMKLQAKLEKLDVLEKECFKLTTTQKTAEDKIKHLEEKLKEEEHQRKLFQD
KASELQTGLEINRILMSSVANPKSSKE---KKKSLK--------------------------KTRCLKERLP
QQIYSKSGSLPIVAK-------K------SSSAS-HSVNTSV-QSLLQVTQHYG-P--HTHQKL---TESRC
LYK----PTRT-TS-QG-KAEPSDSKHSISIGDNLSELLMAMQDELDQMNMKHEE---LLNQMKETESRSVC
EDLECELEHLVKKMEIKGEQISKLIKHQENVR--KLQQKVQQSKM-SEASGIQREDNNLKGSKNIKNS--SR
KCLLTNS-LQK-NSNFHPVQ-VHNLQMKLRRDDIMWEQ
>XP_025861568.1/1-477 centrosomal protein CEP57L1 isoform X1 [Vulpes
vulpes]XP_025861569.1 centrosomal protein CEP57L1 isoform X1 [Vulpes
vulpes]XP_025861570.1 centrosomal protein CEP57L1 isoform X1 [Vulpes
vulpes]XP_025861571.1 centrosomal protein CEP57L1 isoform X1 [Vulpes vulpes]

---MDSELMHSIV----GSYLKPP----
-E-------RV-IFPSFTQNNES-----SQTPH--YA-----NFEGTSPKMLHSPN-------SQ-------
-------ALMLALKTLQEKIHRLELERTQAEDNLNILSREAAEYKKALEKETNERNLAHQELIKQKKDITIQ
LSSAQSRCTLLEKQLEYTKRMVLNVEREKNMILEQQ------------------------------------
-----------AQLQREKEQDHMKLQAKLEKLDVLEKECFKLTTTQKTAEDKIKHLEEKLKEEEHQRKLFQD
KASELQTGLEINRILMSSVANPKSSRE---KKKSLK--------------------------KTRCLKEGPP
QQIYSKSGSLPIVAK-------K------SSSAS-HSVNTSM-QSLLQVTHHYG-P--HTHQKL---TEPRC
LYK----PTRT-TS-QA-KAEPSDSKHSISIGDNLSELLMAMQDELDQMNMKHEELLNLLNQMKETESHSVC
EDLECELEHLVKKMEVKGEQISKLIKHQENVR--KLQQKVQQSKM-SEASGIQREDNNLKGSKNIKNS--SR
KCLLTNS-LQK-NSNFHPVQ-VHNLQMKLRRDDIMWEQ
>XP_041609770.1/1-477 centrosomal protein CEP57L1 [Vulpes
lagopus]XP_041609772.1 centrosomal protein CEP57L1 [Vulpes
lagopus]XP_041609781.1 centrosomal protein CEP57L1 [Vulpes
lagopus]XP_041609788.1 centrosomal protein CEP57L1 [Vulpes lagopus]

---MDSELMHSIV----GSYLKPP----
-E-------RV-FFPSFTQNNES-----SQTPH--YA-----NFEGTSPKMLHSPN-------SQ-------
-------ALMLALKTLQEKIHRLELERTQAEDNLNILSREAAEYKKALEKETNERNLAHQELIKQKKDITIQ
LSSAQSRCTLLEKQLEYTKRMVLNVEREKNMILEQQ------------------------------------
-----------AQLQREKEQDHMKLQAKLEKLDVLEKECFKLTTTQKTAEDKIKHLEEKLKEEEHQRKLFQD
KASELQTGLEINRILMSSVANPKSSRE---KKKSLK--------------------------KTRCLKEGPP
QQIYSKSGSLPIVAK-------K------YSSAS-HSVNTSM-QNLLQVTQHYG-P--HTHQKL---TEPRC
LYK----PTRT-TS-QA-KAEPSDSKHSISIGDNLSELLMAMQDELDQMNMKHEELLNLLNQMKETESHSVC
EDLECELEHLVKKMEVKGEQISKLIKHQENVR--KLQQKVQQSKM-SEASGIQREDNNLKGSKNIKNS--SR
KCLLTNS-LQK-NSNFHPVQ-VHNLQMKLRRDDIMWEQ
>CAD7678214.1/1-476 unnamed protein product [Nyctereutes procyonoides]

---MI-----
---MDSELMHSIV----GSYLKPP----
-E-------RV-FFPSFTQNNES-----SQTPH--YA-----NIEGTSPKMLHSPN-------SQ-------
-------ALMLALKTLQEKIHRLELERTQAEDNLNILSREAAEYKKALEKETNERNLAHQELIKQKKDITIQ
LSSAQSRCTLLEKQLEYTKRMVLNVEREKNMILEQQ------------------------------------
-----------AQLQREKEQDHMKLQAKLEKLDVLEKECFKLTTTQKTAEDKIKHLEEKLKEEEHQRKLFQD
KASELQTGLEINRILMSSVSNPKSSKE---KKKSLK--------------------------KTRCLKERPP
QQIYSKSGSLPIVAK-------K------SSSAS-HSVNTSM-QSLLQVAQHYG-P--HTHQKL---TEPRC
LYK----PIRT-TS-QG-KAEPSDSKHSISIGDNLSELLMAMQDELDQMNMKHEE---LLNQMKETESHSVR
EDLECELEHLVKKMEIKGEQISKLIKHQENVR--KLQQKVQQSKM-SEASGIQREDNNLKGSKNIKNS--SR
KCLLTNS-LQK-NSSFHPVQ-VHNLQMKLRRDDIMWEQ
>XP_032712828.1/1-475 LOW QUALITY PROTEIN: centrosomal protein CEP57L1-like
[Lontra canadensis]

---MDSELMHSIV----GSYHKP-----
---------XV-FIPSFTQNDAA-----SQTHH--ST-----NIEATFPKMLHSPK-------SQ-------
-------ALILALKTLQEKIHHLELERTQAEDNLNILSREAAQYKKALEKETNERILAHQELIKQKKDITLQ
LSSAQSRCTLLEKQLEYTKRMVLNVEREKNMILEQQ------------------------------------
-----------AQLQREKEQDHRKQQAKLEKLDVLEKECFKFTTTQKTAEDKIKHLEEKLKEEEHQRKLFQD
KASELQTGLEINRILMSSVSTPKHSKE---KKKSLK--------------------------KTKCLKGGPP
QQTYSKFGSLPIVAE-------E------SASAS-CFVNSSM-QSILQLMQHYG-P--HTCQKLPEVTEPPC
LYK----PLRT-TS-QG-KAEPSDSKITISIGDSLSELLMAMQDELDQMSMEHEE---LLNQMKDTESHSVC
EDTECELEHLAKKMEIKEEQISKLLKHQDNVR--KLQQKFQNSKT-SEASKIQREENNLKGSKTIKNS--PR
RCLLTNS-LQK-NSNFHPVQ-VHNLQMKLRRDDIMWEQ
>XP_059032896.1/1-479 centrosomal protein CEP57L1 isoform X3 [Mustela
lutreola]XP_059032897.1 centrosomal protein CEP57L1 isoform X3 [Mustela
lutreola]XP_059032898.1 centrosomal protein CEP57L1 isoform X3 [Mustela
lutreola]XP_059032899.1 centrosomal protein CEP57L1 isoform X3 [Mustela
lutreola]XP_059032900.1 centrosomal protein CEP57L1 isoform X3 [Mustela
lutreola]

-------------------------M--I-----
---MDSELMHSIV----GSYHKPP----
-E-------RV-FIPSFTQNDAT-----SQTHH--ST-----NLEATTPKMLHSPN-------SQ-------
-------ALILALKTLQEKIHRLELERTQAEDNLNILSREAAQYKKALEKETNERILAHQELIKQKKDITIQ
LNSAQSRCTVLEKQLEYTKRMVLNVEREKNMILEQQ------------------------------------
-----------AQLQREKEQDHRKLQAKLEKLDVLEKECFKLTTTQKTAEDKIKHLEEKLKEEEHQRKLFQD
KASELQTGLEINRILMSSVSNPKHSKE---KKKSLK--------------------------KTKCLKGGPP

QQTYSKFGSLPIVAE-------K------SASAS-CSVNSSM-QSLLQLMQHYG-P--HTCQKLPEVTEPPC
LYK----PLRT-TS-QG-KAEPSDSKISISIGDSLSELLMAMQDELDQMSMEHEE---LLNQMKDTESHSVC
EDIECELEHLVKKMEIKEEQISKLLKHQANVR--KLQQKVQNSKM-SEASGIQREENNLKGSKPIKNS--PR
KCLLTNS-LQK-NSNFHPVQ-VHNLQMKLRRDDIMWEQ
>XP_004753531.1/1-479 centrosomal protein CEP57L1 isoform X3 [Mustela putorius furo]XP_004753532.1 centrosomal protein CEP57L1 isoform X3 [Mustela putorius furo]XP_004753533.1 centrosomal protein CEP57L1 isoform X3 [Mustela putorius furo]XP_004753534.1 centrosomal protein CEP57L1 isoform X3 [Mustela putorius furo]XP_004753535.1 centrosomal protein CEP57L1 isoform X3 [Mustela putorius furo]

------------------------M---I-----
--MDSELMHSIV----GSYHKPP----
-E-------RV-FIPSFTQNDAT-----SQTHH--ST-----NLEATTPKMLHSPN-------SQ-------
-------ALILALKTLQEKIHRLELERTQAEDNLNILSREAAQYKKALEKETNERILAHQELIKQKKDITIQ
LNSAQSRCTLLEKQLEYTKRMVLNVEREKNMILEQQ------------------------------------
-----------AQLQREKEQDHRKLQAKLEKLDVLEKECFKLTTTQKTAEDKIKHLEEKLKEEEHQRKLFQD
KASELQTGLEINRILMSSVSNPKHSKE---KKKSLK-------------------------KTKCLKGGPP
QQTYSKFGSLPIVAE-------K------SASAS-CSVNSSM-QSLLQLMQHYG-P--HTCQKLPEVTEPPC
LYK----PLRT-TS-QG-KAEPSDSKISISIGDSLSELLMAMQDELDQMSMEHEE---LLNQMKDTESHSVC
EDIECELEHLVKKMEIKEEQISKLLKHQANVR--KLQQKVQNSKM-SEASGIQREENNLKGSKPIKNS--PR
KCLLTNS-LQK-NSNFHPVQ-VHNLQMKLRRDDIMWEQ
>XP_032194755.1/1-479 centrosomal protein CEP57L1 isoform X3 [Mustela erminea]XP_032194756.1 centrosomal protein CEP57L1 isoform X3 [Mustela erminea]XP_032194757.1 centrosomal protein CEP57L1 isoform X3 [Mustela erminea]XP_032194759.1 centrosomal protein CEP57L1 isoform X3 [Mustela erminea]XP_032194760.1 centrosomal protein CEP57L1 isoform X3 [Mustela erminea]

------------------------M---I-----
--MDSELMHSMV----GSYHKPP----
-E-------RV-FIPSFTQNDAA-----SQTHH--ST-----NLEATTPKMLHSPN-------SQ-------
-------ALILALKTLQEKIHRLELERTQAEDNLNILSREAAQYKKALEKETNERILAHQELIKQKKDITIQ
LNSAQSRCTLLEKQLEYTKRMVLNVEREKNMILEQQ------------------------------------
-----------AQLQREKEQDHRKLQAKLEKLDVLEKECFKLTTTQKTAEDKIKHLEEKLKEEEHQRKLFQD
KASELQTGLEINRILMSSVSNPKHSKE---KKKSLK-------------------------KTKCLKGGPP
QQTYSKFGSLPIVTE-------K------SASAS-CSVNSSM-QSLLQLMQHYG-P--HICQKLPEVTEPPC
FYK----PLRT-TS-QG-KAEPSDSKISISIGDSLSELLMAMQDELDQMSMEHEE---LLNQMKDTESHSVC
EDIECELEHLVKKMEIKEEQISKLLKHQDNVR--KLQQKVQNSKM-SEASGIQREENNLKGSKPIKNS--PR
KCLLTNS-LQK-NNNFHPVQ-VHNLQMKLRRDDIMWEQ
>XP_022375893.1/1-479 centrosomal protein CEP57L1 isoform X1 [Enhydra lutris kenyoni]

------------------------M---I-----
--MDSELMHSIV----GSYHKPP----
-E-------RV-FIPSFTQNDAA-----SQTHH--ST-----NLEATSPQMLHSPN-------SQ-------
-------ALLLALKTLQEKIHRLELERTQAEDNLNILSREAAQYKKALEKETNERILAHQELIKQKKDITIQ
LSSAQSRCTLLEKQLEYTKRMVLNVEREKNMILEQQ------------------------------------
-----------AQLQREKEQDHRKLQAKLEKLDVLEKECFKLTTTQKTAEDKIKHLEEKLKEEEHQRKLFQD
KASELQTGLEINRILMSSVSNPKHSKE---KKKSLK-------------------------KTKCLKGGPP
QQTYSKFGSLPIVAE-------K------SASAS-CSVNSSM-QSLLQLMQHYG-P--HTCQKLPEVTEPPC
LYK----PLRT-TS-QG-KAEPSDSKISISVGDSLSELLMAMQDELDQMSMEHEE---LLNQMKDTESHSVC
EDIECELEHLVKKMEIKEEQISKLLKHQDNVR--KLQQKVQNSKM-SEASGIQRDENNLKGSKTIKNS--PR
RCLLTNS-LQK-NSNFHPVQ-VHNLQMKLRRDDIMWEQ
>XP_047589454.1/1-479 centrosomal protein CEP57L1 isoform X3 [Lutra lutra]XP_047589455.1 centrosomal protein CEP57L1 isoform X3 [Lutra

lutra]XP_047589456.1 centrosomal protein CEP57L1 isoform X3 [Lutra
lutra]XP_047589457.1 centrosomal protein CEP57L1 isoform X3 [Lutra
lutra]XP_047589458.1 centrosomal protein CEP57L1 isoform X3 [Lutra lutra]

-------------------------M---I-----
--MDSELMHSIV----GSYHKPP----
-E-------RV-FIPSFTQNDAA-----SQTHH--ST-----NLEATSPKMLHSPN-------SQ-------
-------ALLLALKTLQEKIHRLELERTRAEDNLNILSREAAQYKKALEKETNERILAHQELIKQKKDITIQ
LSSAQSRCTLLEKQLEYTKRMVLNVEREKNMILEQQ------------------------------------
-----------AQLQREKEQDHRKLQAKLEKLDVLEKECFKLTTTQKTAEDKIKHLEEKLKEEEHQRKLFQD
KASELQTGLEINRILMSSVSNPKHSKE---KKKSLK-------------------------KTKCLKGGPP
QQTYSKFGSLPIVAE-------K------SASAS-CSVNSSM-QSLLQLMQHYG-P--HTCQKLPEVTEPPC
LYK----PLRT-TS-QG-KAEPSDSKISISIGDSLSELLMAMQDELDQMSMEHED---LLNQMKDTESHSVC
EDIECELEHLVKKMEIKEEQISKLLKHQDNVR--KLQQKVQNSKM-SEASGIQREENNLKGSKTIKNS--PR
RCLLTNS-LQK-NSNFHPVQ-VHNLQMKLRRDDIMWEQ
>XP_045861838.1/1-479 centrosomal protein CEP57L1 isoform X3 [Meles
meles]XP_045861839.1 centrosomal protein CEP57L1 isoform X3 [Meles
meles]XP_045861840.1 centrosomal protein CEP57L1 isoform X3 [Meles
meles]XP_045861841.1 centrosomal protein CEP57L1 isoform X3 [Meles
meles]XP_045861842.1 centrosomal protein CEP57L1 isoform X3 [Meles
meles]XP_045861843.1 centrosomal protein CEP57L1 isoform X3 [Meles
meles]XP_045861844.1 centrosomal protein CEP57L1 isoform X3 [Meles
meles]XP_045861845.1 centrosomal protein CEP57L1 isoform X3 [Meles meles]

-------------------------M---I-----
--MDSELMHSIV----GSYHKPP----
-E-------RV-FIPSFTQNDAA-----SQTHH--ST-----NLEATSPKMLHSPN-------SQ-------
-------ALILALKTLQEKIHRLELERTQAEDNLNILSREAAQYKKALEKETNERILAHQELIKQKKDITIQ
LSSAQSRCTLLEKQLEYTKRMVLNVEREKNMILEQQ------------------------------------
-----------AQLQREKEQDHRKLQAKLEKLDVLEKECFKLTTTQKTAEDKIKHLEEKLKEEEHQRKLFQD
KASELQTGLEINRILMSSVSNPKHSKE---KKKSLK-------------------------KTKCLKGGPP
QQTYSKFGSLPIVAE-------K------SASAS-CSVNSSM-QSLLQLMQHYG-P--HTCQKLPEVTEPPC
LYK----PLRT-TS-QG-KAEPSDSKISISIGDSLSELLMAMQDELDQMSMEHEE---LLNQMKDTESHSVC
EDIECELEHLVKKMEIKEEQISKLLKHQDNVR--KLQQKVQNSKM-SEASGIRREENNLKGSKTIKNS--PR
KCLLTSS-LQK-NSNFHPVQ-VHNLQMKLRRDDIMWEQ
>XP_032714164.1/1-479 centrosomal protein CEP57L1 isoform X3 [Lontra
canadensis]XP_032714165.1 centrosomal protein CEP57L1 isoform X3 [Lontra
canadensis]

-------------------------M---I-----
--MDSELMHSIV----GSYHKPP----
-E-------RV-FIPSFTQNDAA-----SQTHH--ST-----NVEATSPKMLHSPN-------SQ-------
-------ALILALKTLQEKIHRLELERTQAEDNLNILSREAAQYKKALEKETNERILAHQELIKQKKDITIQ
LSSAQSRCTLLEKQLEYTKRMVLNVEREKNMILEQQ------------------------------------
-----------AQLQREKEQDHRKLQAKLEKLDVLEKECFKLTTTQKTAEDKIKHLEEKLKEEEHQRKLFQD
KASELQTGLEINRILMSSVSNPKHSKE---KKKSLK-------------------------KTKCLKGGPP
QQTYSKFGSLPIVAE-------K------SASAS-CSVNSSM-QSLLQLMQHYG-P--HTCQKLPEVTEPPC
LYK----PLRT-TS-QG-KAEPSDSKISISIGDSLSELLMAMQDELDQMSIEHEE---LLNQMKDTESHSVC
EDIECELEHLVKKMEIKEEQISKLLKHQDNVR--KLQQKVQNSKM-SEASKIQREENNLKGSKTIKNS--PR
RCLLTNS-LQK-NSNFHPVQ-VHNLQMKLRRDDIMWEQ
>XP_044100402.1/1-479 centrosomal protein CEP57L1 isoform X3 [Neogale
vison]XP_044100409.1 centrosomal protein CEP57L1 isoform X3 [Neogale
vison]XP_044100414.1 centrosomal protein CEP57L1 isoform X3 [Neogale
vison]XP_044100419.1 centrosomal protein CEP57L1 isoform X3 [Neogale vison]

---MI-----
---MDSELMHSVV----GSYHKPP----
-E-------RV-FIPSFTQNDAA-----SRTHH--ST-----NLEATSPKMLHSPN-------SQ-------
-------ALILALKTLQEKIHRLELERTQAEDNLNIPSREAAQYKKALEKETNERILAHQELIKQKKDITIQ
LNSAQSRCTLLEKQLEYTKRMVLNVEREKNMILEQQ------------------------------------
-----------AQLQREKEQDHRKLQAKLEKLDVLEKECFKLTTTQKTAEDKIKHLEEKLKEEEHQRKLFQD
KASELQTGLEINRILMSSVSNPKHSKE---KKKSLK--------------------------KTKCLKGGPP
QQTYSKFGSLPIVAE-------K------SASAS-CSVNSSM-QSLLQLMQHYG-P--HTCQKLPEVTEPPC
LYK----PLRT-TS-QG-KAEPSDSKISISIGDSLSELLMAMQDELDQMSMEHEE---LLNQMKDTESHSVC
EDIECELEHLVKKMEIKEEQISKLLKHQDNVR--KLQQKVQNSKM-SEATGIQREENNLKGSKPIKNS--PR
KCLLTNS-LQK-NSNFHPVQ-VHNLQMKLRRDDIMWEQ
>VCW48838.1/1-479 unnamed protein product [Gulo gulo]

---MI-----
---MDSELIHSIV----GSYHKPP----
-E-------RV-FIPSFTQNDAA-----SQTHH--ST-----NLEATSPKMLHSPN-------SQ-------
-------ALILALKTLQEKIHRLELERTQAEDNLNILSREAAQYKKALEKETNERILAHQELIKQKKDITIQ
LSSAQSRCTLLEKQLEYTKRMVLNVEREKNMILEQQ------------------------------------
-----------AQLQREKEQDHRKLQAKLEKLDVLEKECFKLTTTQKTAEDKIKHLEEKLKEEEHQRKLFQD
KASELQTGLEINRILMSSVSNPKYSKE---KKKSLK--------------------------KTKCLKGGPP
QQTYSKFGSLPIVAE-------K------SASAS-CSVNSSM-QSLLQLMQHYG-P--HTCQKRPEVTEPPC
LYK----PLRT-TS-QG-KAEPSDSKISISIGDSLSELLMAMQDELDQMSMEHEE---LLSQMKDTESHSVC
EDIECELEHLVKKMEIKEEQISKLLKHQDNVR--KLQQKVQNSKM-SEASGIQREENNLKGSKTIKNS--PR
KCLLTNS-LQK-NSNFHPVQ-VHNLQMKLRRDDIMWEQ
>XP_059257149.1/1-488 centrosomal protein CEP57L1 isoform X1 [Mustela nigripes]

---M--------------------

-------------------EVERVDL-------------------------------------VSTI-----
---MDSELMHSIV----GSYHKPP----
-E-------RV-FIPSFTQNDAT-----SQTHH--ST-----NLEATTPKMLHSPN-------SQ-------
-------ALILALKTLQEKIHRLELERTQAEDNLNILSREAAQYKKALEKETNERILAHQELIKQKKDITIQ
LNSAQSRCTLLEKQLEYTKRMVLNVEREKNMILEQQ------------------------------------
-----------AQLQREKEQDHRKLQAKLEKLDVLEKECFKLTTTQKTAEDKIKHLEEKLKEEEHQRKLFQD
KASELQTGLEINRILMSSVSNPKHSKE----KKSLK--------------------------KTKCLKGGPP
QQTYSKFGSLPIVAE-------K------SASAS-CSVNSSM-QSLLQLMQHYG-P--HTCQKLPEVTEPPC
LYK----PLRT-TS-QG-KAEPSDSKISISIGDSLSELLMAMQDELDQMSMEHEE---LLNQMKDTESHSVC
EDIECELEHLVKKMEIKEEQISKLLKHQANVR--KLQQKVQNSKM-SEASGIQREENNLKGSKPIKNS--PR
KCLLTNS-LQK-NSNFHPVQ-VHNLQMKLRRDDIMWEQ
>XP_059032894.1/1-489 centrosomal protein CEP57L1 isoform X1 [Mustela lutreola]

---M--------------------

-------------------EVESVDL-------------------------------------VSTI-----
---MDSELMHSIV----GSYHKPP----
-E-------RV-FIPSFTQNDAT-----SQTHH--ST-----NLEATTPKMLHSPN-------SQ-------
-------ALILALKTLQEKIHRLELERTQAEDNLNILSREAAQYKKALEKETNERILAHQELIKQKKDITIQ
LNSAQSRCTVLEKQLEYTKRMVLNVEREKNMILEQQ------------------------------------
-----------AQLQREKEQDHRKLQAKLEKLDVLEKECFKLTTTQKTAEDKIKHLEEKLKEEEHQRKLFQD
KASELQTGLEINRILMSSVSNPKHSKE---KKKSLK--------------------------KTKCLKGGPP
QQTYSKFGSLPIVAE-------K------SASAS-CSVNSSM-QSLLQLMQHYG-P--HTCQKLPEVTEPPC
LYK----PLRT-TS-QG-KAEPSDSKISISIGDSLSELLMAMQDELDQMSMEHEE---LLNQMKDTESHSVC
EDIECELEHLVKKMEIKEEQISKLLKHQANVR--KLQQKVQNSKM-SEASGIQREENNLKGSKPIKNS--PR
KCLLTNS-LQK-NSNFHPVQ-VHNLQMKLRRDDIMWEQ
>XP_004753528.1/1-489 centrosomal protein CEP57L1 isoform X1 [Mustela putorius furo]

---M--------------------

-------------------EVESVDL-------------------------------------VSTI-----
---MDSELMHSIV----GSYHKPP----
-E-------RV-FIPSFTQNDAT-----SQTHH--ST-----NLEATTPKMLHSPN-------SQ-------
-------ALILALKTLQEKIHRLELERTQAEDNLNILSREAAQYKKALEKETNERILAHQELIKQKKDITIQ
LNSAQSRCTLLEKQLEYTKRMVLNVEREKNMILEQQ------------------------------------
-----------AQLQREKEQDHRKLQAKLEKLDVLEKECFKLTTTQKTAEDKIKHLEEKLKEEEHQRKLFQD
KASELQTGLEINRILMSSVSNPKHSKE---KKKSLK--------------------------KTKCLKGGPP
QQTYSKFGSLPIVAE-------K------SASAS-CSVNSSM-QSLLQLMQHYG-P--HTCQKLPEVTEPPC
LYK----PLRT-TS-QG-KAEPSDSKISISIGDSLSELLMAMQDELDQMSMEHEE---LLNQMKDTESHSVC
EDIECELEHLVKKMEIKEEQISKLLKHQANVR--KLQQKVQNSKM-SEASGIQREENNLKGSKPIKNS--PR
KCLLTNS-LQK-NSNFHPVQ-VHNLQMKLRRDDIMWEQ
>XP_032194753.1/1-489 centrosomal protein CEP57L1 isoform X1 [Mustela erminea]

---M--------------------

-------------------EVESVDL-------------------------------------VSTI-----
---MDSELMHSMV----GSYHKPP----
-E-------RV-FIPSFTQNDAA-----SQTHH--ST-----NLEATTPKMLHSPN-------SQ-------
-------ALILALKTLQEKIHRLELERTQAEDNLNILSREAAQYKKALEKETNERILAHQELIKQKKDITIQ
LNSAQSRCTLLEKQLEYTKRMVLNVEREKNMILEQQ------------------------------------
-----------AQLQREKEQDHRKLQAKLEKLDVLEKECFKLTTTQKTAEDKIKHLEEKLKEEEHQRKLFQD
KASELQTGLEINRILMSSVSNPKHSKE---KKKSLK--------------------------KTKCLKGGPP
QQTYSKFGSLPIVTE-------K------SASAS-CSVNSSM-QSLLQLMQHYG-P--HICQKLPEVTEPPC
FYK----PLRT-TS-QG-KAEPSDSKISISIGDSLSELLMAMQDELDQMSMEHEE---LLNQMKDTESHSVC
EDIECELEHLVKKMEIKEEQISKLLKHQDNVR--KLQQKVQNSKM-SEASGIQREENNLKGSKPIKNS--PR
KCLLTNS-LQK-NNNFHPVQ-VHNLQMKLRRDDIMWEQ
>XP_045861836.1/1-489 centrosomal protein CEP57L1 isoform X1 [Meles meles]

---M--------------------

-------------------EVGSVDL-------------------------------------VSTI-----
---MDSELMHSIV----GSYHKPP----
-E-------RV-FIPSFTQNDAA-----SQTHH--ST-----NLEATSPKMLHSPN-------SQ-------
-------ALILALKTLQEKIHRLELERTQAEDNLNILSREAAQYKKALEKETNERILAHQELIKQKKDITIQ
LSSAQSRCTLLEKQLEYTKRMVLNVEREKNMILEQQ------------------------------------
-----------AQLQREKEQDHRKLQAKLEKLDVLEKECFKLTTTQKTAEDKIKHLEEKLKEEEHQRKLFQD
KASELQTGLEINRILMSSVSNPKHSKE---KKKSLK--------------------------KTKCLKGGPP
QQTYSKFGSLPIVAE-------K------SASAS-CSVNSSM-QSLLQLMQHYG-P--HTCQKLPEVTEPPC
LYK----PLRT-TS-QG-KAEPSDSKISISIGDSLSELLMAMQDELDQMSMEHEE---LLNQMKDTESHSVC
EDIECELEHLVKKMEIKEEQISKLLKHQDNVR--KLQQKVQNSKM-SEASGIRREENNLKGSKTIKNS--PR
KCLLTSS-LQK-NSNFHPVQ-VHNLQMKLRRDDIMWEQ
>XP_032714162.1/1-489 centrosomal protein CEP57L1 isoform X1 [Lontra canadensis]

---M--------------------

-------------------EVESVDL-------------------------------------VSTI-----
---MDSELMHSIV----GSYHKPP----
-E-------RV-FIPSFTQNDAA-----SQTHH--ST-----NVEATSPKMLHSPN-------SQ-------
-------ALILALKTLQEKIHRLELERTQAEDNLNILSREAAQYKKALEKETNERILAHQELIKQKKDITIQ
LSSAQSRCTLLEKQLEYTKRMVLNVEREKNMILEQQ------------------------------------
-----------AQLQREKEQDHRKLQAKLEKLDVLEKECFKLTTTQKTAEDKIKHLEEKLKEEEHQRKLFQD
KASELQTGLEINRILMSSVSNPKHSKE---KKKSLK--------------------------KTKCLKGGPP
QQTYSKFGSLPIVAE-------K------SASAS-CSVNSSM-QSLLQLMQHYG-P--HTCQKLPEVTEPPC
LYK----PLRT-TS-QG-KAEPSDSKISISIGDSLSELLMAMQDELDQMSIEHEE---LLNQMKDTESHSVC
EDIECELEHLVKKMEIKEEQISKLLKHQDNVR--KLQQKVQNSKM-SEASKIQREENNLKGSKTIKNS--PR
RCLLTNS-LQK-NSNFHPVQ-VHNLQMKLRRDDIMWEQ
>XP_047589452.1/1-489 centrosomal protein CEP57L1 isoform X1 [Lutra lutra]

---M--------------------

-------------------EVESVDL-------------------------------------VSTI-----
---MDSELMHSIV----GSYHKPP----
-E-------RV-FIPSFTQNDAA-----SQTHH--ST-----NLEATSPKMLHSPN-------SQ-------
-------ALLLALKTLQEKIHRLELERTRAEDNLNILSREAAQYKKALEKETNERILAHQELIKQKKDITIQ
LSSAQSRCTLLEKQLEYTKRMVLNVEREKNMILEQQ------------------------------------
-----------AQLQREKEQDHRKLQAKLEKLDVLEKECFKLTTTQKTAEDKIKHLEEKLKEEEHQRKLFQD
KASELQTGLEINRILMSSVSNPKHSKE---KKKSLK--------------------------KTKCLKGGPP
QQTYSKFGSLPIVAE-------K------SASAS-CSVNSSM-QSLLQLMQHYG-P--HTCQKLPEVTEPPC
LYK----PLRT-TS-QG-KAEPSDSKISISIGDSLSELLMAMQDELDQMSMEHED---LLNQMKDTESHSVC
EDIECELEHLVKKMEIKEEQISKLLKHQDNVR--KLQQKVQNSKM-SEASGIQREENNLKGSKTIKNS--PR
RCLLTNS-LQK-NSNFHPVQ-VHNLQMKLRRDDIMWEQ
>XP_044100395.1/1-486 centrosomal protein CEP57L1 isoform X2 [Neogale vison]

---M--------------------

-------------------EVESVD--SI-----
---MDSELMHSVV----GSYHKPP----
-E-------RV-FIPSFTQNDAA-----SRTHH--ST-----NLEATSPKMLHSPN-------SQ-------
-------ALILALKTLQEKIHRLELERTQAEDNLNIPSREAAQYKKALEKETNERILAHQELIKQKKDITIQ
LNSAQSRCTLLEKQLEYTKRMVLNVEREKNMILEQQ------------------------------------
-----------AQLQREKEQDHRKLQAKLEKLDVLEKECFKLTTTQKTAEDKIKHLEEKLKEEEHQRKLFQD
KASELQTGLEINRILMSSVSNPKHSKE---KKKSLK--------------------------KTKCLKGGPP
QQTYSKFGSLPIVAE-------K------SASAS-CSVNSSM-QSLLQLMQHYG-P--HTCQKLPEVTEPPC
LYK----PLRT-TS-QG-KAEPSDSKISISIGDSLSELLMAMQDELDQMSMEHEE---LLNQMKDTESHSVC
EDIECELEHLVKKMEIKEEQISKLLKHQDNVR--KLQQKVQNSKM-SEATGIQREENNLKGSKPIKNS--PR
KCLLTNS-LQK-NSNFHPVQ-VHNLQMKLRRDDIMWEQ
>XP_044100391.1/1-489 centrosomal protein CEP57L1 isoform X1 [Neogale vison]

---M--------------------

-------------------EVESVDS-------------------------------------VSTI-----
---MDSELMHSVV----GSYHKPP----
-E-------RV-FIPSFTQNDAA-----SRTHH--ST-----NLEATSPKMLHSPN-------SQ-------
-------ALILALKTLQEKIHRLELERTQAEDNLNIPSREAAQYKKALEKETNERILAHQELIKQKKDITIQ
LNSAQSRCTLLEKQLEYTKRMVLNVEREKNMILEQQ------------------------------------
-----------AQLQREKEQDHRKLQAKLEKLDVLEKECFKLTTTQKTAEDKIKHLEEKLKEEEHQRKLFQD
KASELQTGLEINRILMSSVSNPKHSKE---KKKSLK--------------------------KTKCLKGGPP
QQTYSKFGSLPIVAE-------K------SASAS-CSVNSSM-QSLLQLMQHYG-P--HTCQKLPEVTEPPC
LYK----PLRT-TS-QG-KAEPSDSKISISIGDSLSELLMAMQDELDQMSMEHEE---LLNQMKDTESHSVC
EDIECELEHLVKKMEIKEEQISKLLKHQDNVR--KLQQKVQNSKM-SEATGIQREENNLKGSKPIKNS--PR
KCLLTNS-LQK-NSNFHPVQ-VHNLQMKLRRDDIMWEQ
>XP_004406751.1/1-476 PREDICTED: centrosomal protein CEP57L1 isoform X1
[Odobenus rosmarus divergens]

---MDSELIHGIV----GSYLKPP----
-E-------RV-FIPSFIQNDES-----SQTHH--CA-----NLEATSPKMLHSPN-------SQ-------
-------ALILALKTLQEKIHRLELERTQAEDNLNILSREAAQYKKALEKEANERNLAQQELIKQKKDITIQ
LSSAQSRCTLLEKQLEYTKRMVLNVEREKNMILEQQ------------------------------------
-----------AQLQREKEQDHRKLQAKLEKLDILEKECFKLTTTQKTAEDKIKHLEEKLKEEEHQRKLFQD
KASELQTGLEISRILMSSVSNPKHCKE----KKSLK--------------------------KTRCLKGGPP
QQIHSKFGSLPVVAE-------K------SASAN-YSVNASM-QSLLQLMKHYG-P--HNCQKLTEVTEPRC
LYK----PTRT-TS-HS-KAEPSDSKNSVSIGDNLSELLMAMQDELDQMSMEHEE---LLSQMKETESHSVC
EDIECELEHLVKKMEIKGEQISKLLKHQDNVR--KLQQKVQNSKM-SEASGIQREESNLKGSKNIKNS--PR
KCLLTNS-LQK-NSKFHPVQ-VHNLQMKLRRDDIMWEQ

>XP_021548772.1/1-479 centrosomal protein CEP57L1 isoform X1 [Neomonachus schauinslandi]XP_021548773.1 centrosomal protein CEP57L1 isoform X1 [Neomonachus schauinslandi]

---MI-----
---MDSELMHSIV----GSYLKPP----
-E-------RV-FIPSFTQNDES-----SQTHH--SA-----NLEATSPKMLHSPN-------SQ-------
-------ALILALKTLQEKIHRLELERTQAEDNLNILSREAAQYKKALEKETNERNLAHQELIKQKKDITIQ
LSSAQSRCTLLEKQLEYTKRMVLNVEREKNMILEQQ------------------------------------
-----------AQLQREKEQDHRKLQAKLEKLDVLEKECFKLTTTQKTAEDKIKHLEEKLKEEEHQRKLFQD
KASELQTGLEINRILMSSVSNPKHCKE---KKKSLK--------------------------KTRCLKGGPP
QQIHSEFGSLPVVAE-------K------SASAN-YPVNASM-QSLLQLMQRYG-P--HNCQKLTEVTEPRC
LYK----PTRT-TS-QG-KAEPSDSKNSISIGDNLSELLMAMQDELDQMSMEHEE---LLNQMKETESHLVC
EDIECELEHLVKKMEIKGEQISKLLKHQDNVR--KLQQKVQNSKM-SEASGIQREESNLKGSKNIKNS--PR
KCLLTNS-LQK-NSNFHPVQ-VHNLQMKLRRDDIMWEQ
>XP_034847655.1/1-480 centrosomal protein CEP57L1 [Mirounga leonina]XP_034847656.1 centrosomal protein CEP57L1 [Mirounga leonina]XP_034847657.1 centrosomal protein CEP57L1 [Mirounga leonina]XP_034847658.1 centrosomal protein CEP57L1 [Mirounga leonina]XP_045721235.1 centrosomal protein CEP57L1 isoform X1 [Mirounga angustirostris]XP_045721236.1 centrosomal protein CEP57L1 isoform X1 [Mirounga angustirostris]XP_045721237.1 centrosomal protein CEP57L1 isoform X1 [Mirounga angustirostris]XP_045721238.1 centrosomal protein CEP57L1 isoform X1 [Mirounga angustirostris]XP_054361462.1 centrosomal protein CEP57L1 isoform X1 [Mirounga angustirostris]

---MI-----
---MDSELMHSIV----GSYLKPP----
-E-------RV-FIPSFTQNDES-----SQTHH--SA-----NLEATSPKMLHSPN-------SQ-------
-------ALILALKTLQEKIHRLELERTQAEDNLNILSREAAQYKKALEKETNERNLAHQELIKQKKDITIQ
LSSAQSRCTLLEKQLEYTKRMVLNVEREKNMILEQQ------------------------------------
-----------AQLQREKEQDHRKLQAKLEKLDVLEKECFKLTTTQKTAEDKIKHLEEKLKEEEHQRKLFQD
KASELQTGLEINRILMSSVSNPKHCKEK--KKKSLK--------------------------KTRCLKGGPP
QQIHSEFGSLPVVAE-------K------SASAN-YPVNASM-QSVLQLMQHYG-P--HNCQKLTEVTEPRC
LYK----PTRT-TS-QG-KAEPSDSKNSISIGDNLSELLMAMQDELDQMSMEHEE---LLNQMKETESHLVC
EDIECELEHLVKKMEIKGEQISKLLKHQDNVR--KLQQKVQNSKM-SEASGIQREESNLKGSKNIKNS--PR
KCLLTNS-LQK-NSNFHPVQ-VHNLQMKLRRDDIMWEQ
>XP_035931190.1/1-478 LOW QUALITY PROTEIN: centrosomal protein CEP57L1 [Halichoerus grypus]

---MDSELMHSIV----GSYLKPP----
-E-------RV-FIPSFTQNDES-----SQTHH--SA-----NLEATSPKMLHSPN-------SQ-------
-------ALILALKTLQEKIHRLELERTQAEDNLNSLSREAAQYXKALEKETNERNLAHQELIKQKKDITIQ
LSSAQSRCTLLEKQLEYTKRMVLNVEREKNMILEQQ------------------------------------
-----------AQLQREKEQDHRKLQAKLEKLDVLEKECFKLTTTQKTAEDKIKHLEEKLKEEEHQRKLFQD
KASELQTGLEINRILMSSVSNPKHCKEK--KKKSLK--------------------------KTRCLKGGPP
QQIHSKFGSLPVVAE-------K------SASAN-YPVNASM-QSLLQLMQHYG-P--HNCQKLTEVTEPRC
LYK----PTRT-TS-QG-KAEPSDSKNSISIGDNLSELLMAMQDELDQMSMEHEE---LLNQMKETESHSVC
EDIECELEHLVKKMEIKGEQISKLLKHQDNVR--KLQQKVQNSKM-SEASGIQREDSNLKGSKNIKNS--PR
KCLLTNS-LQK-NSNFHPVQ-VHNLQMKLRRDDIMWEQ
>XP_032263989.1/1-480 centrosomal protein CEP57L1 isoform X1 [Phoca vitulina]XP_032263991.1 centrosomal protein CEP57L1 isoform X1 [Phoca vitulina]XP_032263992.1 centrosomal protein CEP57L1 isoform X1 [Phoca vitulina]XP_032263993.1 centrosomal protein CEP57L1 isoform X1 [Phoca

vitulina]XP_032263994.1 centrosomal protein CEP57L1 isoform X1 [Phoca vitulina]XP_032263995.1 centrosomal protein CEP57L1 isoform X1 [Phoca vitulina]

---MI-----
---------------------------------------MDSELMHSIV----GSYLKPP----
-E-------RV-FIPSFTQNDES-----SQTHH--SA-----NLEATSPKMLHSPN-------SQ-------
-------ALILALKTLQEKIHRLELERTQAEDNLNSLSREAAQYKKALEKETNERNLAHQELIKQKKDITIQ
LSSAQSRCTLLEKQLEYTKRMVLNVEREKNMILEQQ--------------------------------
-----------AQLQREKEQDHRKLQAKLEKLDVLEKECFKLTTTQKTAEDKIKHLEEKLKEEEHQRKLFQD
KASELQTGLEINRILMSSVSNPKHCKEK--KKKSLK------------------------KTRCLKGGPP
QQIHSKFGSLPAVAE-------K------SASAN-YPVDASM-QSLLQLMQHYG-P--HNCQKLTEVTEPRC
LYK----PTRT-TS-QG-KAEPSDSKNSISIGDNLSELLMAMQDELDQMSMEHEE---LLNQMKETESHSVC
EDIECELEHLVKKMEIKGEQISKLLKHQDNVR--KLQQKVQNSKM-SEASGIQREDSNLKGSKNIKNS--PR
KCLLTNS-LQK-NSNFHPVQ-VHNLQMKLRRDDIMWEQ
>XP_027459005.1/1-477 centrosomal protein CEP57L1 [Zalophus californianus]XP_027459008.1 centrosomal protein CEP57L1 [Zalophus californianus]

---------------------------------------MDSELMHSIV----GSYLKPP----
-E-------RV-FIPSFIQNDES-----SQTHH--CA-----NLEATSPKMLHSPN-------SQ-------
-------ALILALKTLQEKIHRLELERTQAEDNLNILSREAAQYKKALEKETHERNLAHQELIKQKKDITIQ
LSSAQSRCTLLEKQLEYTKRMVLNVEREKNMILEQQ--------------------------------
-----------AQLQREKEQDHRKLQAKLENLDILEKECFKLTTTQKTAEDKIKHLEEKLKEEEHQRKLFQD
KASELQTGLEISRILMSSVSNPKHCKE---KKKSLK------------------------KTRCLKGGPP
QQIHSKFGSLPVVAE-------K------SASAN-YSVNASM-QSLLQLMKHYG-P--HNCQKLTEVTEPRC
LYK----PTRT-TF-QG-KAEPSDSKNSISIGDNLSELVMAMQDELDQMSMEHKE---LLNQMKETESHSVC
EDIECELEHLVKKMEIKGEQISKLLKHQDNVR--KLQQKVQNSKM-SEASGVQREESNLKGSKNIKNS--PR
KCLLTNS-LQK-NSNFHPVQ-VHNLQMKLRRDDIMWEQ
>XP_027960087.1/1-477 centrosomal protein CEP57L1 isoform X1 [Eumetopias jubatus]

---------------------------------------MDSELMHSIV----GSYLKPP----
-E-------RV-FIPSFIQNDES-----SQTHH--CA-----NLEATSPKMLHSPN-------SQ-------
-------ALILALKTLQEKIHRLELERTQAEDNLNILSREAAQYKKALEKETHERNLAHQELIKQKKDITIQ
LSSAQSRCTLLEKQLEYTKRMVLNVEREKNMILEQQ--------------------------------
-----------AQLQREKEQDHRKLQAKLENLDILEKECFKLTTTQKTAEDKIKHLEEKLKEEEHQRKLFQD
KASELQTGLEISRILMSSVSNPKHCKE---KKKSLK------------------------KTRCLKGGPP
QQIHSKFGSLPVVAE-------K------SASAN-YSVNASM-QSLLQLMKHYG-P--HNCQKLTEVTEPRC
LYK----PTRT-TF-QG-KAEPSDSKNSISIGDNLSELVMAMQDELDQMSMEHEE---LLNQMKETESHSVC
EDIECKLEHLVKKMEIKGEQISKLLKHQDNVR--KLQQKVQNSKM-SEASGVQREESNLKGSKNIKNS--PR
KCLLTNS-LQK-NSNFHPVQ-VHNLQMKLRRDDIMWEQ
>XP_025719481.1/1-479 centrosomal protein CEP57L1 isoform X1 [Callorhinus ursinus]XP_025719486.1 centrosomal protein CEP57L1 isoform X1 [Callorhinus ursinus]

---MI-----
---------------------------------------MDSELMHSIV----GSYLKPP----
-E-------RV-FIPSFIQNDES-----SQTHH--CA-----NLEATSPKMLHSPN-------SQ-------
-------ALILALKTLQEKIHRLELERTQAEDNLNILSREAAQYKKALEKETHERNLAHQELIKQKKDITIQ
LSSAQSRCTLLEKQLEYTKRMVLNVEREKNMILEQQ--------------------------------
-----------AQLQREKEQDHRKLQAKLENLDILEKECFKLTTTQKTAEDKIKHLEEKLKEEEHQRKLFQD

KASELQTGLEISRILMSSVSNPKHCKE---KKKSLK-------------------------KTRCLKGGPP
QQIHSKFGSLPVVAE-------K------SASVN-YSVNASM-QSLLQLMKHYG-P--HNCQKLTEVTEPRC
LYK----PTRT-TF-QG-KAEPSDSKNSISIGDNLSELVMAMQDELDQMSMEHEE---LLNQMKETESHSVC
EDIECELEHLVKKMEIKGEQISKLLKHQDNVR--KLQQKVQNSKM-SEASGVQREESNLKGSKNIKNS--PR
KCLLTNS-LQK-NSNFHPVQ-VHNLQMKLRRDDIMWEQ
>XP_002917389.1/1-479 centrosomal protein CEP57L1 isoform X1 [Ailuropoda melanoleuca]XP_011220816.1 centrosomal protein CEP57L1 isoform X1 [Ailuropoda melanoleuca]XP_011220817.1 centrosomal protein CEP57L1 isoform X1 [Ailuropoda melanoleuca]XP_019653200.1 centrosomal protein CEP57L1 isoform X1 [Ailuropoda melanoleuca]XP_034524848.1 centrosomal protein CEP57L1 isoform X1 [Ailuropoda melanoleuca]

---MI-----
--MDSELMHSMV----GSYLKPP----
-E-------RV-FIPSFSQNYES-----SQTHH--SA-----NLEVTAPKMLHSPN-------SQ-------
-------ALILALKTLQEKIHRLELERTQAEDNLNVLSREAAQYKKALEKETNERNLAHQELIKQKKDITIQ
LSSAQSRCTLLEKQLEYTKRMVLNVEREKNMILEQQ------------------------------------
-----------AQLQREKEQDHRKLQAKLEKLDVLEKECFKLTTTQKTAEDKIKHLEEKLKEEEHQRKLFQD
KASELQTGLEINRILMSSVSNPKRSKE---KKKSLK-------------------------KTKCLKGGPP
QQIYSKLGSPPITAE-------K------SSSAS-YSVNASM-QSLLQLMQHYG-P--HTCQKLTEVTEPRC
LYK----PTRI-TS-QG-KAEPSDSKNSISIGDNLSELLMAMQDELDQMSMEHEE---LLNQMKETESHSVC
EDIECELEYLVKKMEIKGEQISKLMKHQDNVR--KLQQKVQNSKM-NEASGIQREESNLKGSKNIKNS--PR
KCLLTNS-LQK-NSNFHPVQ-VHNLQMKLRRDDIMWEQ
>XP_008704460.2/1-479 centrosomal protein CEP57L1 isoform X1 [Ursus maritimus]XP_040483544.1 centrosomal protein CEP57L1 isoform X1 [Ursus maritimus]

---MI-----
--MDSELMHSMV----GSYLKPP----
-E-------RV-FIPSFSQNYES-----SQTHH--SA-----NLEVTAPKMLHSPN-------SQ-------
-------ALILALKTLQEKIHRLELERTQAEDNLNVLSREAAQYKKALEKETNERNLAHQELIKQKKDITIQ
LSSAQSRCTLLEKQLEYTKRMVLNVEREKNMILEQQ------------------------------------
-----------AQLQREKEQDHRKLQAKLEKLDVLEKECFKLTTTQKTAEDKIKHLEEKLKEEEHQRKLFQD
KASELQTGLEINRILMSSVSNPKRSKE---KKKSLK-------------------------KTKCLKGGPP
QQIYSKLGSPPITAE-------K------SSSAS-SSVNASM-QSLLQLMQHYR-P--HTCQKLTEVTEPRC
LYK----PTRT-TS-QG-KAEPSDSKNSISIGDNLSELLMAMQDELDQMSMEHEE---LLNQMKETESHSVC
EDIECELEYLVKKMEIKGEQISKLMKHQDNVR--KLQQKVQNSKM-NEASGIQREESNLKGSKNIKNS--PR
KCLLTNS-LQK-NSNFHPVQ-VHNLQMKLRRDDIMWEQ
>XP_045654386.1/1-479 centrosomal protein CEP57L1 isoform X1 [Ursus americanus]XP_045654387.1 centrosomal protein CEP57L1 isoform X1 [Ursus americanus]

---MI-----
--MDSELMHSMV----GSYLKPP----
-E-------RV-FIPSFSQNYES-----SQTHH--SA-----NLEVTAPKMLHSPN-------SQ-------
-------ALILALKTLQEKIHRLELERTQAEDNLNVLSREAAQYKKALEKETNERNLAHQELVKQKKDITIQ
LSSAQSRCTLLEKQLEYTKRMVLNVEREKNMILEQQ------------------------------------
-----------AQLQREKEQDHRKLQAKLEKLDVLEKECFKLTTTQKTAEDKIKHLEEKLKEEEHQRKLFQD
KASELQTGLEINRILMSSVSNPKRSKE---KKKSLK-------------------------KTKCLKGGPP
QQIYSKLGSPPITAE-------K------SSSAS-SSVNASM-QSLLQLMQHYR-P--HTCQKLTEVTEPRC
LYK----PTRT-TS-QG-KAEPSDSKNSISIGDNLSELLMAMQDELDQMSMEHEE---LLNQMKETESHSVC
EDIECELEYLVKKMEIKGEQISKLMKHQDNVR--KLQQKVQNSKM-NEASGIQREESNLKGSKNIKNS--PR
KCLLTNS-LQK-NSNFHPVQ-VHNLQMKLRRDDIMWEQ
>XP_044244766.1/1-479 centrosomal protein CEP57L1 isoform X1 [Ursus arctos]XP_044244767.1 centrosomal protein CEP57L1 isoform X1 [Ursus

arctos]XP_057167195.1 centrosomal protein CEP57L1 isoform X1 [Ursus arctos]XP_057167196.1 centrosomal protein CEP57L1 isoform X1 [Ursus arctos]

---MI-----
--MDSELMHSMV----GSYLKPP----
-E-------RV-FIPSFSQNYES-----SQTHH--SA-----NLEVTAPKMLHSPN-------SQ-------
-------ALILALKTLQEKIHRLELERTQAEDNLNVLSREAAQYKKALEKETNERNLAHQELIKQKKDITIQ
LSSAQSRCTLLEKQLEYTKRMVLNVEREKNMILEQQ------------------------------------
-----------AQLQREKEQDHRKLQAKLEKLDVLEKECFKLTTTQKTAEDKIKHLEEKLKEEEHQRKLFQD
KASELQTGLEISRILMSSVSNPKRSKE---KKKSLK-------------------------KTKCLKGGPP
QQIYSKLGSPPITAE-------K------SSSAS-SSVNASM-QSLLQLMQHYR-P--HTCQKLTEVTEPRC
LYK----PTRT-TS-QG-KAEPSDSKNSISIGDNLSELLMAMQDELDQMSMEHEE---LLNQMKETESHSVC
EDIECELEYLVKKMEIKGEQISKLMKHQDNVR--KLQQKVQNSKM-NEASGIQREESNLKGSKNIKNS--PR
KCLLTNS-LQK-NSNFHPVQ-VHNLQMKLRRDDIMWEQ
>XP_030171964.1/1-477 centrosomal protein CEP57L1 isoform X1 [Lynx canadensis]XP_030171965.1 centrosomal protein CEP57L1 isoform X1 [Lynx canadensis]

--MDSELMHSIV----GSYLKPP----
-E-------RV-FVPSFAQNNES-----SQTYR--SA-----NLEVTSPKMLHSPN-------GQ-------
-------ALILALKTLQEKIHRLELERTQAEDNLNILSREAAQYKEALEKETNERNLVHQELIKQKKDITIQ
LNSAQSRCTLLEKQLEYTKRMVLNVEREKNMILEQQ------------------------------------
-----------AQLQREKEQDHMKLQAKLEKLDVLEKECFKLTATQKTAEGKIKHLEEKLKEEEHQRKLFQD
KASELQTGLEISRILMSSVSNKKCSKE---KKKSSK-------------------------KTKCLKRGPR
QQIYSKFESLPIMAE-------K------SASTG-HSVNRSM-QSLLQMMQHYG-P--HILQKPTEVTELRC
LYK----PTRT-ST-QC-RAEPSDSKNSLSICDNLSELLMAMQDELDQMSMEHEE---LLNQMKETESHSVC
EDIECELEHLVKKMEIKGEQISKLMKHQDNVR--KLQQKIQNSKM-SEASGIQREDSSPKGSKNIKNS--PR
KCLLTNS-LQK-NSNFHPVR-VHNLQMKLRRDDIMWEQ
>XP_046958537.1/1-477 centrosomal protein CEP57L1 [Lynx rufus]XP_046958538.1 centrosomal protein CEP57L1 [Lynx rufus]

--MDSELMHSIV----GSYLKPP----
-E-------RV-FVPSFAQNNES-----SQTYR--SA-----NLEVTSPKMLHSPN-------GQ-------
-------ALILALKTLQEKIHRLELERTQAEDNLNILSREAAQYKEALEKETNERNLVHQELIKQKKDITIQ
LNSAQSRCTLLEKQLEYTKRMVLNVEREKNMILEQQ------------------------------------
-----------AQLQREKKQDHMKLQAKLEKLDVLEKECFKLTATQKTAEGKIKHLEEKLKEEEHQRKLFQD
KASELQTGLEISRILMSSVSNKKCSKE---KKKSSK-------------------------KTKCLKRGPR
QQIYSKFESLPIMAE-------K------SASTG-HSVNRSM-QSLLQMMQHYG-P--HILQKPTEVTELRC
LYK----PTRT-ST-QC-RAEPSDSKNSLSICDNLSELLMAMQDELDQMSMEHEE---LLNQMKETESHSVC
EDIECELEHLVKKMEIKGEQISKLMKHQDNVR--KLQQKIQNSKM-SEASGIQREDSSPKGSKNIKNS--PR
KCLLTNY-LQK-NSNFHPVR-VHNLQMKLRRDDIMWEQ
>XP_058590893.1/1-477 centrosomal protein CEP57L1 isoform X1 [Neofelis nebulosa]XP_058590894.1 centrosomal protein CEP57L1 isoform X1 [Neofelis nebulosa]

--MDSELMHSIV----GSYLKPP----
-E-------RV-FVPSFAQNNES-----SQTYR--SV-----NLEVTSPKMLHSPN-------GQ-------
-------ALILALKTLQEKIHRLELERTQAEDNLNILSREAAQYKEALEKETNERNLVHQELKKQKKDITIQ
LNSAQSRCTLLEKQLEYTKRMVLNVEREKNMILEQQ------------------------------------
-----------AQLQREKEQDHMKLQAKLEKLDVLEKECFKLTATQKTAEGKIKHLEEKLKEEEHQRKLFQD

KASELQTGLEISRILMSSVSNKKCSKE---KKKSSK--------------------------KTKCLKRGPR
QQIYSKFESLPIMAE-------K------SASAG-HSVNRNM-QSLLQMMQHYG-P--HILQKPTEVTELRC
LYK----PTRT-SA-QC-KAEPSDSKNSLSICDNLSELLMAMQDELDQMSMEHEE---LLNQMKETESHSVC
EDIECELEHLVKKMEIKGEQISKLMKHQDNVR--KLQQKIQNSKM-SEASGIQREDSSPKGSKNIKNS--PR
KCLLTNS-LQK-NSNFHPVR-VHNLQMKLRRDDIMWEQ
>XP_025780923.1/1-477 centrosomal protein CEP57L1 [Puma concolor]

---MDSELMHSIV----GSYLKPP----
-E-------RV-FVPSFAQNNET-----SQTYR--SA-----NLEVTSPKMLHSPN-------GQ-------
-------ALILALKTLQEKIHRLELERTQAEDNLNILSREAAQYKEALEKETNERNLVHQELIKQKKDITIQ
LNSAQVRCTLLEKQLEYTKRMVLNVEREKNMILQQQ------------------------------------
-----------AQLQREKEQDHMKLQAKLEKLDVLEKECFKLTATQKTAEGKIKHLEEKLKEEEHQRKLFQD
KASELQTGLEISRILMSSVSNKKCSKE---KKKSSK--------------------------KTKCLKRGPR
QQIYSKFESLPIMAE-------K------SASAG-HSVNRRM-QSLLQMMQHYG-P--HILQKPTEVTELRC
LYK----PTRT-SA-QC-KAEPSDSKNSLSICDNLSELLMAMQDELDQMSMEHEE---LLNQMKETESHSVC
EDIECELEHLVKKMEIKGEQISKLMKHQDNVR--KLQQKIQKSKM-SEASGIQREDSSPKGSKNIKNS--PR
KCLLTNS-LQK-NSNFHPVR-VHNLQMKLRRDDIMWEQ
>XP_045355182.1/1-479 centrosomal protein CEP57L1 isoform X2 [Leopardus geoffroyi]

---MI-----
---MDSELMHSIV----GSYLKPP----
-E-------RV-FVPSFAQNNES-----SQTYR--SA-----NLEVTSPKMLHSPN-------GQ-------
-------ALILALKTLQEKIHRLELERTQAEDNLNILSREAAQYKEALEKETNERNLVHQELIKQKKDITIQ
LNSAQSRCTLLEKQLEYTKRMVLNVEREKNMILEQQ------------------------------------
-----------AQLQREKEQDHMKLQAKLEKLDVLEKECFKLTATQKTAEGKIKHLEEKLKEEEHQRKLFQD
KASELQTGLEISRILMSSVSNKKCSKE---KKKSSK--------------------------KTKCLKRGPR
QQIYSKFESLPIMAE-------K------SASAG-HSVNRSM-QSLLQMMQHYG-P--HILQKPTEVTELRC
LYK----PTRT-SA-QC-KAEPSDSKNSLSICDNLSELLMAMQDELDQMSMEHEE---LLNQMKETESHSVC
EDIECELEHLVKKMEIKGEQISKLMKHQDNVR--KLQQKIQNSKM-SEASGIQREDSSPKGSKNIKNS--PR
KCLLTNS-LQK-NSNFHPVR-VHNLQMKLRRDDIMWEQ
>XP_047713949.1/1-479 centrosomal protein CEP57L1 isoform X1 [Prionailurus viverrinus]

---MI-----
---MDSELMHSIV----GSYLKPP----
-E-------RV-FVPSFAQNNES-----SQTYR--SA-----NLEVTSPKMLHSPN-------GQ-------
-------ALILALKTLQEKIHRLELERTQAEDNLNILSREAAQYKEALEKETNERNLVHQELIKQKKDITIQ
LNSAQVRCTLLEKQLEYTKRMVLNVEREKNMILEQQ------------------------------------
-----------AQLQREKEQDHMKLQAKLEKLDVLEKECFKLTATQKTAEGKIKHLEEKLKEEEHQRKLFQD
KASELQTGLEISRILMSSVSNKKCSKE---KKKSSK--------------------------KTKCLKRGPR
QQIYSKFESLPIMAE-------K------SASAG-HSVNRSM-QSLLQMMQHYE-P--HILQKPTEVTELRC
LYK----PTRT-SA-QC-KAEPSDSKNSLSICDNLSELLMAMQDELDQMSMEHEE---LLNQMKETESHSVC
EDIECELEHLVKKMEIKGEQISKLMKHQDNVR--KLQQKIQNSKM-TEASGIQREDSSPKGSKNIKNS--PR
KCLLTNS-LQK-NSNFHPVR-VHNLQMKLRRDDIMWEQ
>XP_011280817.2/1-479 centrosomal protein CEP57L1 isoform X1 [Felis catus]

---MI-----
---MDSELMHSIV----GSYLKPP----
-E-------RV-FVPSFAQNNES-----SQTYR--SA-----NLEVTSPKMLHSPN-------GQ-------
-------ALILALKTLQEKIRRLELERTQAEDNLNILSREAAHYKEALEKETNERNLVHQELIKQKKDITIQ
LNSAQVRCTLLEKQLEYTKRMVLNVEREKNMILEQQ------------------------------------

-----------AQLQREKEQDHMKLQAKLEKLDVLEKECFKLTATQKTAEGKIKHLEEKLKEEEHQRKLFQD
KASELQTGLEISRILMSSVSNKKCSKE---KKKSSK-------------------------KTKCLKRGPH
QQIYSKFESLPIMAE-------K------SASAG-HSVNRSM-QSLLQMMQHYG-P--HILQKPTEVTELRC
LYK----PTRT-SA-QC-KAEPSDSKNSLSICDNLSELLMAMQDELDQMSMEHEE---LLNQMKETESHSVC
EDIECELEHLVKKMEIKGEQISKLMKHQDNVR--KLQQKIQNSKM-SEASGIQREDSSPKGSKNIKNS--PR
KCLLTNS-LQK-NSNFHPVR-VHNLQMKLRRDDIMWEQ
>XP_040330267.1/1-479 centrosomal protein CEP57L1 isoform X1 [Puma
yagouaroundi]

---MI-----
---MDSELMHSIV----GSYLKPP----
-E-------RV-FVPSFAQNNET-----SQTYR--SA-----NLEVTSPKMLHSPN-------GQ-------
-------ALILALKTLQEKIHRLELERTQAEDNLNILSREAAQYKEALEKETNERNLVHQELIKQKKDITIQ
LNSAQVRCTLLEKQLEYTKRMVLNVEREKNMILEQQ------------------------------------
-----------AQLQREKEQDHMKLQAKLEKLDVLEKECFKLTATQKTAEGKIKHLEEKLKEEEHQRKLFQD
KASELQTGLEISRILMSSVSNKKCSKE---KKKSSK-------------------------KTKCLKRGPR
QQIYSKFESLPITAE-------K------SASAG-HSVNRSM-QSLLQMMQHYG-P--HILQKPTEVTELRC
LYK----PTRT-SA-QC-KAEPSDSKNSLSICDNLSELLMAMQDELDQMSMEHKE---LLNQMKETESHSVC
EDIECELEHLVKKMEIKGEQISKLMKHQDNVR--KLQQKIQKSKM-SEASGIQREDSSPKGSKNIKNS--PR
KCLLTNS-LQK-NSNFHPVR-VHNLQMKLRRDDIMWEQ
>XP_053078370.1/1-479 centrosomal protein CEP57L1 isoform X1 [Acinonyx jubatus]

---MI-----
---MDSELMHSIV----GSYLKPP----
-E-------RV-FVPSFAQNNET-----SQTYC--SA-----NLEVTSPKMLHSPN-------GQ-------
-------ALILALKTLQEKIHRLELERTQAEDNLNILSREAAQYKEALEKETNERNLVHQELIKQKKDITVQ
LNSAQVRCTLLEKQLEYTKRMVLNVEREKNMILEQQ------------------------------------
-----------AQLQREKEQDHMKLQAKLEKLDVLEKECFKLTATQKTAEGKIKHLEEKLKEEEHHRKLFQD
KASELQTGLEISRILMSSVSNKKCSKK---KKKSSK-------------------------KTKCLKRGPR
QQIYSKFESLPIMAE-------K------SASAG-HSVNRSM-QSLLQMMQHYG-P--HILQKPTEVTELRC
LYK----PTRT-SA-QC-KAEPSDSKNSLSICDNLSELLMAMQDELDQMSMEHEE---LLNQMKETESHSVC
EDIECELEHLVKKMEIKGEQISKLMKHQDNVR--KLQQKIQNSKM-SEASGIQREDSSPKGSKNIKSS--PR
KCLLTNS-LQK-NSNFHPVR-VHNLQMKLRRDDIMWEQ
>XP_042842884.1/1-479 centrosomal protein CEP57L1 isoform X1 [Panthera
tigris]XP_042842885.1 centrosomal protein CEP57L1 isoform X1 [Panthera
tigris]XP_042842886.1 centrosomal protein CEP57L1 isoform X1 [Panthera
tigris]XP_042842888.1 centrosomal protein CEP57L1 isoform X1 [Panthera tigris]

---MI-----
---MDSELMHSIV----GSYLKPP----
-E-------RV-FVPSFAQNNES-----SQTYR--SA-----NLEVTSPKMLHSPN-------GQ-------
-------ALILALKTLQEKIHRLELERTQAEDNLNILSREAAQYKEALEKETNERNLVHQELKKQKKDIMIQ
LNSAQSRCTLLEKQLEYTKRMVLNVEREKNMILEQQ------------------------------------
-----------AQLQREKEQEHMKLQAKLEKLDVLEKECFKLTATQKTAEGKIKHLEEKLKEEEHQRKLFQD
KASELQTGLEISRILMSSVSNKKCSKE---KKKSSK-------------------------KTKCLKRGPH
QQIYSKFESLPIMAE-------K------SASAG-HSVNRSM-QSLLQMMQHYG-P--HILRKPTEVTELRC
LYK----PTRT-SA-QC-KAEPSDSKNSLSICDNLSELLMAMQGELDQMSMEHEE---LLNQMKETESHSVC
EDIECELEHLVKKMEMKGEQISKLMKHQDNVR--KLQQKIQNSKM-SEASGIQREDSSPKGSKNIKNS--PR
KCLLTNS-LQK-NSNFHPVR-VHNLQMKLRRDDIMWEQ
>XP_060472328.1/1-479 centrosomal protein CEP57L1 isoform X1 [Panthera
onca]XP_060472329.1 centrosomal protein CEP57L1 isoform X1 [Panthera onca]

---MI-----

--MDSELMHSIV----GSYLKPP----
-E-------RV-FVPSFAQNNES-----SQTYR--SA-----NLEVTSPKMLHSPN-------GQ-------
-------ALILALKTLQAKIHRLELERTQAEDNLNILSREAAQYKEALEKETNERNLVHQELKKQKKDITIQ
LNSAQSRCTLLEKQLEYTKRMVLNVEREKNMILEQQ------------------------------------
-----------AQLQREKEQDHMKLQAKLEKLDVLEKECFKLTATQKTAEGKIKHLEEKLKEEEHQRKLFQD
KASELQTGLEISRILMSSVSNKKCSKE---KKKSSK-------------------------KTKCLKRGPH
QQIYSKFESLPIMAE-------K------SASAG-HSVNRSM-QSLLQMMQHYG-P--HILRKPTEVTELRC
LYK----PTRT-SA-QC-KAEPFDSKNSLSICDNLSELLMAMQGELDQMSMEHEE---LLNQMKETESHSVC
EDIECELEHLVKKMEIKGEQISKLMKHQDNVR--KLQQKIQNSKM-SEASGIQREDSSPKGSKNIKNS--PR
KCLLTNS-LQK-NSNFHPVR-VHNLQMKLRRDDIMWEQ
>XP_049510075.1/1-479 centrosomal protein CEP57L1 isoform X1 [Panthera uncia]XP_049510076.1 centrosomal protein CEP57L1 isoform X1 [Panthera uncia]XP_049510077.1 centrosomal protein CEP57L1 isoform X1 [Panthera uncia]

--MI-----
--MDSELMHSIV----GSYLKPP----
-E-------RV-FVPSFAQNNES-----SQTYR--SA-----NLEVTSPKMLHSPN-------GQ-------
-------ALILALKTLQEKIHRLELERTQAEDNLNILSREAAQYKEALEKETNERNLVHQELKKQKKDITIQ
LNSAQSRCTLLEKQLEYTKRMVLNVEREKNMIVEQQ------------------------------------
-----------AQLQREKEQDHMKLQAKLEKLDVLEKECFKLTATQKTAEGKIKHLEEKLKEEEHQRKLFQD
KASELQTGLEISRILMSSVSNKKCSKE---KKKSSK-------------------------KTKCLKRGPH
QQIYSKFESLPIMAE-------K------SASAG-HSVNRSM-QSLLQMMQHYG-P--HILRKPTEVTELRC
LYK----PTRT-SA-QC-KAEPSDSKNSLSICDNLSELLMAMQGELDQMSMEHEE---LLNQMKETESHSVC
EDIECELEHLVKKMEIKGEQISKLMKHQDNVR--KLQQKIQNSKM-SEASGIQREDSSPKGSKNIKNS--PR
KCLLTNS-LQK-NSNFHPVR-VHNLQMKLRRDDIMWEQ
>XP_042795454.1/1-479 centrosomal protein CEP57L1 isoform X1 [Panthera leo]XP_042795455.1 centrosomal protein CEP57L1 isoform X1 [Panthera leo]XP_042795456.1 centrosomal protein CEP57L1 isoform X1 [Panthera leo]

--MI-----
--MDSELMHSIV----GSYLKPP----
-E-------RV-FVPSFAQNNES-----SQTYR--SA-----NLEVTSPKMLHSPN-------GQ-------
-------ALILALKTLQAKIHRLELERTQAEDNLNILSREAAQYKEALEKETNERNLVHQELKKQKKDITIQ
LNSAQSRCTLLEKQLEYTKRMVLNVEREKNMILEQQ------------------------------------
-----------AQLQREKEQDHMKLQAKLEKLDVLEKECFKLTATQKTAEGKIKHLEEKLKEEEHQRKLFQD
KASELQTGLEISRILMSSVSNKKCSKE---KKKSSK-------------------------KTKCLKRGPH
QQIYSKFESLPIMAE-------K------SASAG-HSVNRSM-QSLLQMMQPYG-P--HILRKPTEVTELRC
LYK----PTRT-SA-QC-KAEPSDSKNSLSICDNLSELLMAMQGELDQMSMEHKE---LLNQMKETESHSVC
EDIECELEHLVKKMEMKGEQISKLMKHQDNVR--KLQQKIQNSKM-SEASGIQREDSSPKGSKNIKNS--PR
KCLLTNS-LQK-NSNFHPVR-VHNLQMKLRRDDIMWEQ
>XP_019298581.2/1-477 centrosomal protein CEP57L1 isoform X1 [Panthera pardus]XP_019298582.2 centrosomal protein CEP57L1 isoform X1 [Panthera pardus]XP_053760568.1 centrosomal protein CEP57L1 isoform X1 [Panthera pardus]

--MDSELMHSIV----GSYLKPP----
-E-------RV-FVPSFAHNNES-----SQTYR--SA-----NLEVTSPKMLHSPN-------GQ-------
-------ALILALKTLQAKIHRLELERTQAEDNLNILSREAAQYKEALEKETNERNLVHQELKKQKKDITIQ
LNSAQSRCTLLEKQLEYTKRMVLNVEREKNMILEQQ------------------------------------
-----------AQLQREKEQDHMKLQAKLEKLDVLEKECFKLTATQKTAEGKIKHLEEKLKEEEHQRKLFQD
KASELQTGLEISRILMSSVSNKKCSKE---KKKSSK-------------------------KTKCLKRGPH
QQIYSKFESLPIMAE-------K------SASAG-HSVNRSM-QSLLQMMQPYG-P--HILQKPTEVTELRC
LYK----PTRT-SA-QC-KAEPSDSKNSLSICDNLSELLMAMQGELDQMSMEHKE---LLNQMKETESHSVC
EDIECELEHLVKKMEMKGEQISKLMKHQDNVR--KLQQKIQNSKM-SEASGIQREDSSPKGSKNIKNS--PR
KCLLTNS-LQK-NSNFHPVR-VHNLQMKLRRDDIMWEQ

>KAF0873796.1/1-477 CE57L protein, partial [Crocuta crocuta]

---MDSELMHSIV----GSYLKPP----
-E-------RV-FVPSLTQSDES-----SQTYR--SV-----NLEGTSPKMLHSPN-------SQ-------
-------ALILALKTLQEKIHRLELERTQAEDNLNILSREAAQYKKALEKETNERNLAHQELIKQKKDITIQ
LNSAQSRCTLLEKQLEYTKRMVLNVEREKNMILEQQ------------------------------------
-----------AQLQREKEQDHMKLQAKLEKLDVLEKECFKLTTTQKTAEDKIKHLEEKLKEEEHQRKLFQD
KASELQTGLEMSRILMSSVSNQKRSKE---KKKSSK--------------------------KTKCLKRGSR
QQTYSKFESLPNMAE-------K------SASAG-CSVNRSM-QSLLQMMQRYG-P--HILQKPTEVTEPRC
LYE----PSRT-TA-QC-KAEHSDSKNSLSICDNLSELLMAMQDELGQMSMEHEE---LLNQMKETESPSVC
EDIECELEHLVKKMEIKEEQISKLMKHQDNVR--KLQQKVQNSKI-SEASGIQQEDNNPKGSKNIKNS--PR
KCLLTNS-LQK-NSNFHPVQ-VHNLQMKLRRDDIMWEQ
>XP_039093337.1/1-483 centrosomal protein CEP57L1 isoform X1 [Hyaena hyaena]

--MI------------------

--PNSAI-----
---MDSELMHSIV----GSYLKPP----
-E-------RV-FVPSLTQSDES-----SQTYR--SV-----NLEVTSPKMLHSPN-------SQ-------
-------ALILALKTLQEKIHRLELERTQAEDNLNILSREAAQYKKALEKETNERNLAHQELIKQKKDITIQ
LNSAQSRCTLLEKQLEYTKRMVLNVEREKNMILEQQ------------------------------------
-----------AQLQREKEQDHMKLQAKLEKLDVLEKECFKLTTTQKTAEDKIKHLEEKLKEEEHQRKLFQD
KASELQTGLEMSRILM-SVSNQKRSKE---KKKSSK--------------------------KTKCLKRGSH
QQTYSKFESLPNMAE-------K------SASAG-CSVNRSM-QSLLQMMQRYG-P--HILQKPTEVTEPRC
LYE----PSRT-TA-QC-KAEHSDSKNSLSICDNLSELLMAMQDELGQMSMEHKE---LLNQMKETESPSVC
EDIECELEHLVKKMEIKEEQISKLMKHQDNVR--KLQQKVQNSKI-SEASGIQQEDNNPKGSKNIKNS--PR
KCLLTNS-LQK-NSNFHPVQ-VHNLQMKLRRDDIMWEQ
>XP_029801124.1/1-477 centrosomal protein CEP57L1 isoform X1 [Suricata suricatta]

--MRI-----
---MDSELMHSIV----GSYLKPP----
-E-------RV-FVPSFIQNDES-----SQTHR---------NLEVTSPKMLHSPN-------SQ-------
-------ALILALKTLQEKIHRLELERTQAEDNLNILSREAAEYKKALEKETNERDLAHQELIKQKKDITIQ
LNSAQSRCTLLEKQLEYTKRMVLNVEREKNMILEQQ------------------------------------
-----------AQLQREKEQDHMKLQAKLEKLDVLEKECFKLTTTQKTAEDKIKHLEEKLKEEEHQRKLFQD
KASELQTGLEISRILMSSVSNQKRSKE----KKSSK--------------------------KTKCLKRGPC
QQIYSRFESLPIMAE-------K------SASAG-RSVNRST-QSLLQMQQHYG-P--YILQKPTEVTKPSC
LYK----PSRT-TA-QC-KAEPSDSKYSLSICDNLTELLMTMQDELDQMSVEHEE---LLNQMKETESHSVC
EDIECELEHLVKKMEIKGEQISKLMKHQDNIR--KLQQKVQNSKM-SEASGIRREDSNLKGSKNIKNS--PR
KCLLTNC-IQK-NNNFHPVE-VHNLQMKLRRDDIMWEQ
>XP_014636636.1/1-453 PREDICTED: centrosomal protein CEP57L1 isoform X2 [Ceratotherium simum simum]

---MI-----
---MDPELMHSIV----GSYLKPP----
-E-------RV-FVPSFTQNDES-----TQTHH--SV-----NLEVTSPKMLHSPN-------SQ-------
-------ALILALKTLQEKIHRLELERTQAEDNLNILSREAAQYKKALENETNERNLAHQELIKQKKDISIQ
LSSAQSRCTLLEKQLEYTKRMVLNVEREKNMILEQQ------------------------------------
-----------AQLQREKEQDHMKLQAKLEKLDVLEKECFKLTTTQKTAEDKIKYLEEKLKEEEHQRKLFQD
KASELQTGLEINRILMSSVSNPKCSKE---KKKSS-------------------------------------
----------------------K------SASAS-HSRNASR-QNLLQMMQHYG-P--HVLQKRAEVTEPRC
LYK----PTRT-TS-RC-EAVPPDSGKSVCICDNLSELLMAMQDELDQMSMEHQE---LLNQMKETESHSVF
ENIECELEHLVKKMEIKGEQISKLKKHQDNVH--KLQQKVQNSRT-SEASGIQQEDSNPNRSKNVKNT--PR

KCLPANP-LQK-NSNFHPIR-VHNLQMKLRRDDIMWEQ
>XP_058422487.1/1-479 centrosomal protein CEP57L1 isoform X2 [Diceros bicornis minor]

---MI-----
---------------------------------------MDPELMHSIV----GSYLKPP----
-E-------RV-FVPSFTQNDES-----TQTHH--SV-----NLEVTSPKMLHSPN-------SQ-------
-------ALILALKTLQEKIHRLELERTQAEDNLNILSREAAQYKKALENETNERNLAHQELIKQKKDISIQ
LSSAQSRCTLLEKQLEYTKRMVLNVEREKNMILEQQ----------------------------------
-----------AQLQREKEQDHMKLQAKLEKLDVLEKECFKLTTTQKTAEDKIKYLEEKLKEEEHQRKLFQD
KASELQTGLEINRILMSSVSNPKCSKE---KKKSSK------------------------KTKCLKRGPP
QQIYSKFGSLPIVVE-------K------SASAS-HSRNASR-QNLLQMMQHYG-P--HVLQKRAEVTEPRC
LYK----PTRT-TS-QC-KAVPPDSGKSICICDNLSELLMAMQDELDQMSMEHQE---LLNQMKETESHSAF
ENIECELEHLVKKMEIKGEQISKLKKHQDSVR--KLQQKVQNSKT-SEASGIQREDSNPNTSKNVKNS--PR
KCLPANP-LQK-NSNFHPIR-VHNLQMKLRRDDIMWEQ
>XP_004422251.2/1-487 PREDICTED: centrosomal protein CEP57L1 isoform X4 [Ceratotherium simum simum]

---------------------------------------MHTSLVYL-----------------
----
---FI-----
---------------------------------------MDPELMHSIV----GSYLKPP----
-E-------RV-FVPSFTQNDES-----TQTHH--SV-----NLEVTSPKMLHSPN-------SQ-------
-------ALILALKTLQEKIHRLELERTQAEDNLNILSREAAQYKKALENETNERNLAHQELIKQKKDISIQ
LSSAQSRCTLLEKQLEYTKRMVLNVEREKNMILEQQ----------------------------------
-----------AQLQREKEQDHMKLQAKLEKLDVLEKECFKLTTTQKTAEDKIKYLEEKLKEEEHQRKLFQD
KASELQTGLEINRILMSSVSNPKCSKE---KKKSSK------------------------KTKCLKRGPP
QQIYSKFGSLPIVVE-------K------SASAS-HSRNASR-QNLLQMMQHYG-P--HVLQKRAEVTEPRC
LYK----PTRT-TS-RC-EAVPPDSGKSVCICDNLSELLMAMQDELDQMSMEHQE---LLNQMKETESHSVF
ENIECELEHLVKKMEIKGEQISKLKKHQDNVH--KLQQKVQNSRT-SEASGIQQEDSNPNRSKNVKNT--PR
KCLPANP-LQK-NSNFHPIR-VHNLQMKLRRDDIMWEQ
>XP_058422486.1/1-516 centrosomal protein CEP57L1 isoform X1 [Diceros bicornis minor]

---------------------------------------MPIFLYFIWDA-------------
--ATA----WL-------------
------DK-RCIGARPGS-EPANPGP------------------------------PQRSI-----
---------------------------------------MDPELMHSIV----GSYLKPP----
-E-------RV-FVPSFTQNDES-----TQTHH--SV-----NLEVTSPKMLHSPN-------SQ-------
-------ALILALKTLQEKIHRLELERTQAEDNLNILSREAAQYKKALENETNERNLAHQELIKQKKDISIQ
LSSAQSRCTLLEKQLEYTKRMVLNVEREKNMILEQQ----------------------------------
-----------AQLQREKEQDHMKLQAKLEKLDVLEKECFKLTTTQKTAEDKIKYLEEKLKEEEHQRKLFQD
KASELQTGLEINRILMSSVSNPKCSKE---KKKSSK------------------------KTKCLKRGPP
QQIYSKFGSLPIVVE-------K------SASAS-HSRNASR-QNLLQMMQHYG-P--HVLQKRAEVTEPRC
LYK----PTRT-TS-QC-KAVPPDSGKSICICDNLSELLMAMQDELDQMSMEHQE---LLNQMKETESHSAF
ENIECELEHLVKKMEIKGEQISKLKKHQDSVR--KLQQKVQNSKT-SEASGIQREDSNPNTSKNVKNS--PR
KCLPANP-LQK-NSNFHPIR-VHNLQMKLRRDDIMWEQ
>KAJ8788693.1/1-450 hypothetical protein J1605_022438 [Eschrichtius robustus]

---------------------------------------MDSELMHSIV----GSYLKPP----
-E-------RV-FVPSVTQSDES-----SQTHY--SV-----NLEVTSPKMLHSPN-------SQ-------
-------ALILALKTLQEKIHRLELERTQAEDNLNILSREAAQYKKALEDETKERNLAHEELIKQKKDISMQ
LSSAQSHCTLLEKQLEYTKRMVLNVEREKNMILEQQ----------------------------------
-----------AQLQREKEQDHMKLQAKLEKLDVLEKECFKLTTTQKTAEGKIKHLEEKLKEEEHQRKLFQD
KASQLQTGLEISRILMSSVSNPKCSKE---KKKSS-----------------------------------
--------------------K------SASAG-HSRNAST-QN-LQTMQHYR-P--HILQKLAEVSEPRC

LYK----PSRT-TS-QC-EAVPSDSEKSISICNNLSELLMAMQDELDQMSMEYQD---LLNQMKETESQSVC
ENIECELEHLVKKMEIKGEQISKLMKHQDSVR--KLQQKVQNSKM-SEASAIQKEDSNCKGSKNIKNS--PR
KCLLTNS-PQK-NSNFRPIR-VHNLQMKLRRDDIMWEQ
>XP_032507489.1/1-476 centrosomal protein CEP57L1 isoform X1 [Phocoena sinus]

---MDFELMHSIV----GSYLKPP----
-E-------RV-FVPSIAQSDES-----SQTHY--SV-----NLEVTSPKMLHSPN-------SQ-------
-------ALILALKTLQEKIHRLELERTQAEDNLNILSREAAQYKKALEYETKERNLAHEELIKQKKDISMQ
LSSAQSHCTLLEKQLEYTKRMVLNVEREKNMILEQQ------------------------------------
-----------AQLQREKEQDHMKLQAKLEKLDVLEKECFKLTTTQKTAEGKIKHLEEKLKEEEHQRKLFQD
KASQLQTGLEISRILMSSISNPKRSKE---KKKSSK--------------------------KTKCLKRGPP
QQIYSKFGSLPIVAE-------K------SASAG-HSRNAST-QN-LQTMQHYR-P--HSLQKLAEVSEPRC
LYK----PSKS-TS-QC-EAVPSDSEKSISICNNLSELLMAMQDELDQMTMEYQD---LLNQMKETESQSVC
ENIECELEYLVKKMEIKGEQISKLMKHQDNVR--KLQQKVQNLKI-SEASAIQKEDSNCKGSKNIKNS--PR
KCLLTNS-PQN-NSNFRPIR-VHNLQMKLRRDDIMWEQ
>XP_024611566.1/1-476 centrosomal protein CEP57L1 isoform X1 [Neophocaena asiaeorientalis asiaeorientalis]

---MDSELMHSIV----GSYLKPP----
-E-------RV-FVPSIAQSDES-----SQTHY--SV-----NLEVTSPKMLHSPN-------SQ-------
-------ALILALKTLQEKIHRLELERTQAEDNLNILSREAAQYKKALEYETKERNLAHEELIKQKKDISMQ
LSSAQSHCTLLEKQLEYTKRMVLNVEREKNMILEQQ------------------------------------
-----------AQLQREKEQDHMKLQAKLEKLDVLEKECFKLTTTQKTAEGKIKHLEEKLKEEEHQRKLFQD
KASQLQTGLEISRILMSSISNPKRSKE---KKKSSK--------------------------KTKCLKRGPP
QQIYSKFGSLPIVAE-------K------SASAG-HSRNAST-QN-LQTMQHYR-P--HSLQKLAEVSEPRC
LYK----PSKS-TS-QS-EAVPSDSEKSISICNNLSELLMAMQDELDQMTMEYQD---LLNQMKETESQSVC
ENIECELEYLVKKMEIKGEQISKLMKHQDNVR--KLQQKVQNLKI-SEASAIQKEDSNCKGSKNIKNS--PR
KCLLTNS-PQN-NSNFRPIR-VHNLQMKLRRDDIMWEQ
>XP_029079750.1/1-476 centrosomal protein CEP57L1 [Monodon monoceros]XP_029079751.1 centrosomal protein CEP57L1 [Monodon monoceros]XP_029079752.1 centrosomal protein CEP57L1 [Monodon monoceros]

---MDSELMHSIV----GSYLKPP----
-E-------RV-FVPSIAQSDES-----SQTHY--SV-----NLEVTSPKMLHSPN-------SQ-------
-------ALILALKTLQEKIHRLELERTQAEDNLNILSREAAQYKKALEDETKERNLAHEELIKQKKDISMQ
LSSAQSHCTLLEKQLEYTKRMVLNVEQEKNMILEQK------------------------------------
-----------AQLQREKEQDHMKLQAKLEKLDVLEKECFKLTTTQKTAEGKIKHLEEKLKEEEHQRKLFQD
KASQLQTGLEISRILMSSISNPKRSKE---KKKSSK--------------------------KTKCLKRGPP
QQIYSKFGSLPIVAE-------K------SASAG-HSRNTST-QN-LQTMQHYR-P--HILQKLAEVSEPRC
LYK----PSKS-TS-QC-EAVPSDSEKSISICNNLSELLMAMQDELDQMTMEYQD---LLNQMKETESQSVC
ENIECELEHLVKKMEIKGEQISKLMKHQDNVR--KLQQKVQNLKI-SEASAIQKEDSNCKGSKNIKNS--PR
KCLLTNS-PQN-NSNFRPIR-VHNLQMKLRRDDIMWEQ
>XP_022409709.1/1-476 centrosomal protein CEP57L1 isoform X1 [Delphinapterus leucas]XP_022409719.1 centrosomal protein CEP57L1 isoform X1 [Delphinapterus leucas]XP_022409742.1 centrosomal protein CEP57L1 isoform X1 [Delphinapterus leucas]XP_030616155.1 centrosomal protein CEP57L1 isoform X1 [Delphinapterus leucas]

---MDSELMHSIV----GSYLKPP----

-E-------RV-FVPSIAQSDES-----SQTHY--SV-----NLEVTSPKMLHSPN-------SQ-------
-------ALILALKTLQEKIHRLELERTQAEDNLNILSREAAQYKKALEDETKERNLAHEELIKQKKDISMQ
LSSAQSHCTLLEKQLEYTKRMVLNVEQEKNMILEQK------------------------------------
-----------AQLQREKEQDHMKLQAKLEKLDVLEKECFKLKTTQKTAEGKIKHLEEKLKEEEHQRKLFQD
KASQLQTGLEISRILMSSISNPKRSKE---KKKSSK-------------------------KSKCLKRGPP
QQIYSKFGSLPIVAE-------K------SASAG-HSRNAST-QN-LQTVQHYR-P--HILQKLAEVSEPRC
LYK----PSKS-TS-QC-EAVPSDSEKSISICNNLSELLMAMQDELDQMTMEYQD---LLNQMKEAESQSVC
ENIECELEHLVKKMEIKGEQISKLMKHQDNVR--KLQQKVQNLKI-SEASAIQKEDSNCKGSKNIKNS--PR
KCLLTNS-PQN-NSNFRPIR-VHNLQMKLRRDDIMWEQ
>XP_061063702.1/1-476 centrosomal protein CEP57L1 isoform X1 [Eubalaena glacialis]XP_061063703.1 centrosomal protein CEP57L1 isoform X1 [Eubalaena glacialis]

--MDSELMHSIV----GSYLKPP----
-E-------RV-FVPSVTQSDES-----SQTHY--SV-----NLEVTSPKMLHSPN-------SQ-------
-------ALILALKTLQEKIHRLELERTQAEDNLNILSREAAQYKKALEDETKERNLAHEELIKQKKDISMQ
LSSAQSHCTLLEKQLEYTKRMVLNVEREKNMILEQQ------------------------------------
-----------AQLQREKEQDHMKLQAKLEKLDVLEKECFKLTTTQKTAEGKIKHLEEKLKEEEHQRKLFQD
KASQLQTGLEISRIFMSSVSNPKCSKE---KKKSSK-------------------------KTKCLKRGPP
QQIYSKFGSLPIVAE-------K------SASAG-HSRNAST-QN-LQTMQHYR-P--HILQKLAEVSEPRC
LYK----PSRT-TS-QC-EAVPSDSEKSISICNNLSELLMAMQDELDQMTMEYQD---LLNQMKETESQSVC
ENIECELEHLVKKMEIKGEQISKLMKHQDSVR--KLQQKVQNSKM-SEASAIQKEDSNCKGSKNIKNS--PR
KCLLTNS-PQK-NRNFRPVR-VHNLQMKLRRDDIMWEQ
>XP_036727945.1/1-476 centrosomal protein CEP57L1 isoform X1 [Balaenoptera musculus]XP_036727946.1 centrosomal protein CEP57L1 isoform X1 [Balaenoptera musculus]XP_036727947.1 centrosomal protein CEP57L1 isoform X1 [Balaenoptera musculus]

--MDSELMHSIV----GSYLKPP----
-E-------RV-FVPSVTQSDES-----SQTHY--SV-----NLEVTSPKMLHSPN-------SQ-------
-------ALILALKTLQEKIHRLELERTQAEDNLNILSREAAQYKKALEDETKERNLAHEELIKQKKDISMQ
LSSAQSHCTLLEKQLEYTKRMVLNVEREKNMILEQQ------------------------------------
-----------AQLQREKEQDHMKLQAKLEKLDVLEKECFKLTTTQKTAEGKIKHLEEKLKEEEHQRKLFQD
KASQLQTGLEISRILMSSVSNPKCSKE---KKKSSK-------------------------KTKCLKRGPP
QQIYSKFGSLPIVAE-------K------SASAG-HSRNAST-QN-LQTMQHYR-P--RILQKLAEVSEPRC
LYK----PSRT-TS-QC-EAVPSDSEKSISICNNLSELLMAMQDELDQMSMEYQD---LLNQMKETESQSVC
ENIECELEHLVKKMEIKGEQISKLMKHQDSVR--KLQQKVQNSKM-SEASAIQKEDSNCKGSKNIKNS--PR
KCLLTNS-PQK-NSNFRPIR-VHNLQMKLRRDDIMWEQ
>XP_007190728.2/1-476 centrosomal protein CEP57L1 isoform X1 [Balaenoptera acutorostrata]

--MDSELMHSIV----GSYLKPP----
-E-------RV-FVPSVTQSDES-----SQTHY--SV-----NLEVTSSKMLHSPN-------SQ-------
-------ALILALKTLQEKIHRLELERTQAEDNLNILSREAAQYKKALEDETKERNLAHEELIKQKKDISMQ
LSSAQSHCTLLEKQLEYTKRMVLNVEREKNMILEQQ------------------------------------
-----------AQLQREKEQDHMKLQAKLEKLDVLEKECFKLTTTQKTAEGKIKHLEEKLKEEEHQRKLFQD
KASQLQTGLEISRILMSSVSNPKCSKE---KKKSSK-------------------------KTKCLKRGPP
QQIYSKFGSLPIVAE-------K------SASAG-HSRNAST-QN-LQTMQHYR-P--HILQKLAEVSEPRC
LYK----PSRT-TS-QC-EAVPSDSEKSISICNNLSELLMAMQDELDQMTMEYQD---LLNQMKETESQSVC
ENIECELEHLVKKMEIKGEQISKLMKHQDSVR--KLQQKVQNSKM-GEASTIQKEDSNCKGSKNIKNS--PR
KCLLTNS-PQK-NSNLRPIR-VHNLQMKLRRDDIMWEQ
>XP_058939611.1/1-476 centrosomal protein CEP57L1 [Kogia breviceps]

---MDSELMHSIV----GSYLKPP----
-E-------RV-FVPSITQSDES-----SQTHY--SV-----NLEVTSPKMLHGPN-------SQ-------
-------ALILALKTLQEKIHRLELERTQAEDNLNILSREAAQYKKALEDETKERNLAHEELIKQKKDISMQ
LSSAQSHCTLLEKQLEYTKRMVLNVEREKNMILEQQ------------------------------------
-----------AQLQREKEQDHMKLQAKLEKLDVLEKECFKLTTTQKTAEGKIKHLEEKLKEEEHQRKLFQD
KASQLQTGLEISRILMSSVSNPKRSKE---KKKSSK--------------------------KTKCLKRGPP
QQIYSKFGSLPIVAE-------K------SASAG-HSRNAST-QN-LQTMQHYR-P--HILQKLAEVSEPRC
LYK----PSRK-TS-QC-EAVPSDSEKSISICNNLSELLMAMQDELDQMTMEYQD---LLNQMKETESQSVC
ENIECELEHLVKKMEIKGEQISKLMKHQDSVH--KLQQKVQNLKM-SEASAIQKEDSNCKGSKNIKNS--PR
KCLLTNS-PQK-NSNFRPIR-VHNLQMKLRRDDIMWEQ
>XP_007100608.1/1-476 centrosomal protein CEP57L1 [Physeter
catodon]XP_028350365.1 centrosomal protein CEP57L1 [Physeter
catodon]XP_028350366.1 centrosomal protein CEP57L1 [Physeter catodon]

---MDSELMHSIV----GSYLKPP----
-E-------RV-FVPSITQSDES-----SQTHY--SV-----NLEVTSPKMLHSPN-------SQ-------
-------ALILALKTLQEKIHRLEVERTQAEDHLNILSREAAQYKKALEDETKERNLAHEELIKQKKDISMQ
LSSAQSHCTLLEKQVEYTKRMVLNVEREKNMILEQQ------------------------------------
-----------AQLQREKEQDHMKLRAKLEKLDVLEKECFKLTTTQKTAEGKIKHLEEKLKEEEHQRKLFQD
KASQLQTGLEISRILMSSVSNPKRSKE---KKKSSK--------------------------KTKCLKRGSP
QQIYSKFGSLPIVAE-------K------SASAG-HSRNAST-QN-LQTMQHYR-P--HILQKLAEVSEPRC
LYK----PSRK-TS-QC-EAVPSDSEKSISICNNLSELLMAMQDELDQMTMEYQD---LLNQMKETESQSVC
ENIECELEHLVKKMEIKGEQISKLMKHQDSVH--KLQQKVQNLKM-SEASAIQKEDSNCKGSKNIKNS--PR
KCLLTNS-PQK-NSNFRPIR-VHNLQMKLRRDDIMWEQ
>XP_026944978.1/1-476 centrosomal protein CEP57L1 isoform X1 [Lagenorhynchus
obliquidens]XP_026944982.1 centrosomal protein CEP57L1 isoform X1
[Lagenorhynchus obliquidens]

---MDSELMHSIV----GSYRKPP----
-E-------RV-FVPSIAQSVES-----SQTHY--SV-----NLEVTSPKMLRSPN-------SQ-------
-------ALILALKTLQEKIHRLELERTQAEDNLNILSREAAQYKKALEDETKERNLAHEELIKQKKDISMQ
LSSAQSHCTLLEKQLEYTKRMVLNVEREKNMILEQQ------------------------------------
-----------AQLQREKEQDHMKLQAKLEKLDVLEKECFILTTTQKTAEGKIKHLEEKLKEEEHQRKLFQD
KASQLQTELEISRILMSSVSNPKRSKE---KKKSSK--------------------------KTKCLKRGPP
QHIYSKFGSLPIVAE-------K------SASAG-HSRNAST-PN-LQTMQHYR-P--HILQKLAEVSEPRC
LYK----PSKS-TS-QC-EAVPSDSERSISVCNNLSELLMAMQDELDQMTMEYQD---LLNQMKETESQSVC
ENIECELEHLVKKMEIKGEQISKLMKHQDNVR--KLQQKVQNLKI-SEASAIQKEDSNYKGSKNIKNS--PR
KCLLTNS-PQK-NSNFRPIR-VHNLQMKLRRDDIMWEQ
>XP_019785105.1/1-476 centrosomal protein CEP57L1 isoform X1 [Tursiops
truncatus]XP_033723499.1 centrosomal protein CEP57L1 isoform X1 [Tursiops
truncatus]XP_060023439.1 centrosomal protein CEP57L1 isoform X1 [Lagenorhynchus
albirostris]XP_060023440.1 centrosomal protein CEP57L1 isoform X1
[Lagenorhynchus albirostris]XP_060023441.1 centrosomal protein CEP57L1 isoform
X1 [Lagenorhynchus albirostris]

---MDSELMHSIV----GSYRKPP----
-E-------RV-FVPSIAQSVES-----SQTHY--SV-----NLEVTSPKMLRSPN-------SQ-------
-------ALILALKTLQEKIHRLELERTQAEDNLNILSREAAQYKKALEDETKERNLAHEELIKQKKDISMQ

LSSAQSHCTLLEKQLEYTKRMVLNVEREKNMILEQQ------------------------------------
-----------AQLQREKEQDHMKLQAKLEKLDVLEKECFKLTTTQKTAEGKIKHLEEKLKEEEHQRKLFQD
KASQLQTELEISRILMSSVSNPKRSKE---KKKSSK-------------------------KTKCLKRGPP
QQIYSKFGSLPIVAE-------K------SASAG-HSRNAST-PN-LQTMQHYR-P--HILQKLAEVSEPRC
LYK----PSKS-TS-QC-EAVPSDSEKSISVCNNLSELLMAMQDELDQMTMEYQD---LLNQMKETESQSVC
ENIECELEHLVKKMEIKGEQISKLMKHQDNVC--KLQQKVQNLKM-SEASAIQKEDSNCKGSKNIKNS--PR
KCLLTNS-PQK-NSNFPPIR-VHNLQMKLRRDDIMWEQ
>XP_030738746.1/1-476 centrosomal protein CEP57L1 isoform X1 [Globicephala melas]XP_030738747.1 centrosomal protein CEP57L1 isoform X1 [Globicephala melas]XP_060139474.1 centrosomal protein CEP57L1 [Globicephala melas]

--MDSELMHSIV----GSYRKPP----
-E-------RV-FVPSIAQSVES-----SQTHY--SV-----NLEVTSPKMLRSPN-------SQ-------
-------ALILALKTLQEKIHRLELERTQAEDNLNILSREAAQYKKALEDETKERNLAHEELIKQKKDISMQ
LSSAQSHCTLLEKQLEYTKRMVLNVEREKNMILEQQ------------------------------------
-----------AQLQREKEQDHMKLQAKLEKLDVLEKECFKLTTTQKTAEGKIKHLEEKLKEEEHQRKLFQD
KASQLQTELEISRILMSSVSNPKRSKE---KKKSSK-------------------------KTKCLKRGPP
QQIYSKFGSLPIVAE-------K------SASAG-HSRNAST-PN-LQTMQHYR-P--HILQKLAEVSEPRC
LYK----PSKS-TS-QC-EAVPSDSEKSISICNNLSELLMAMQDELDQMTMEYQD---LLNQMKETESQSVC
ENIECELEHLVKKMEIKGEQISKLMKHQDNVC--KLQQKVQNLKM-SEASAIQKEDSNCKGSKNIKNS--PR
KCLLTNS-PQK-NSNFPPIR-VHNLQMKLRRDDIMWEQ
>TEA38723.1/1-476 hypothetical protein DBR06_SOUSAS610069 [Sousa chinensis]

--MDSELMHSIV----GSYRKPP----
-E-------RV-FVPSIAQSVES-----SQTHY--SV-----NLEVTSPKMLRSPN-------SQ-------
-------ALILALKTLQEKIHRLELERTQAEDNLNILSREAAQYKKALEDETKERNLAHEELIKQKKDISMQ
LSSAQSHCTLLEKQLEYTKRMVLNVEREKTMILEQQ------------------------------------
-----------AQLQREKEQDHMKLQAKLEKLDVLEKECFKLTTTQKTAEGKIKHLEEKLKEEEHQRKLFQD
KASQLQTELEISRILMSSVSNPKRSKE---KKKSSK-------------------------KTKCLKRGPP
QQIYSKFGSLPIVAE-------K------SASAG-HSRNAST-PN-LQTMQHYR-P--HILQKLAEVSEPRC
LYK----PSKS-TS-QC-EAVPSDSEKSISVCNNLSELLMAMQDELDQMTMEYQD---LLNQMKETESQSVC
ENIECELDHLVKKMEIKGEQISKLMKHQDNVC--KLQQKVQNLKM-SEASAIQKEDSNCKGSKNIKNS--PR
KCLLTNS-PQK-NSNFPPIR-VHNLQMKLRRDDIMWEQ
>XP_059886108.1/1-476 centrosomal protein CEP57L1 isoform X1 [Delphinus delphis]

--MDSELMHSIV----GSYRKPP----
-E-------RV-FVPSIAQSVES-----SQTHY--SV-----NLEVTSPKMLRSPN-------SQ-------
-------ALILALKTLQEKIHRLELERTQAEDNLNILSREAAQYKKALEDETKERNLAHEELIKQKKDISMQ
LSSAQSHCTLLEKQLEYTKRMVLNVEREKNMILEQQ------------------------------------
-----------AQLQREKEQDHMKLQAKLEKLDVLEKECFKLTTTQKTAEGKIKHLEEKLKEEEHQRKLFQD
KASQLQTELEISRILMSSVSNPKRSKE---KKKSSK-------------------------KTKCLKRGPP
QQIYSKFGSLPIVAE-------K------SASAG-HSRNAST-PN-LQTMQHYR-P--HILQKLAEVSEPRC
LYK----PSKS-TS-QC-EAVPSDSEKSISVCNNLSELLMAMQDELDQMTMEYQD---LLNQMKETESQSVC
ENIECELEHLVKKMEIKGEQISKLMKHQDNVC--KLQQKVQNLKM-SEASAIQKEDSNRKGSKNIKNS--PR
KCLLTNS-PQK-NSNFPPIR-VHNLQMKLRRDDIMWEQ
>XP_004264790.1/1-476 centrosomal protein CEP57L1 isoform X1 [Orcinus orca]

--MDSELMHSIV----GSYRKPP----

-E-------RV-FVPSIAQSVES-----SQTHY--SV-----NLEVTSPKMLRSPN-------SQ-------
-------ALILALKTLQEKIHRLELERTQAEDNLNILSREAAQYKKALEDETKERNLAHEELIKQKKDISMQ
LSSAQSHCTLLEKQLEYTKRMVLNVEREKNMILEQQ------------------------------------
-----------AQLQREKEQDHMKLQAKLEKLDVLEKECFKLTTTQKTAEGKIKHLEEKLKEEEHQRKLFQD
KASQLQTELEISRILMSSVSNPKRSKE---KKKSSK--------------------------KTKCLKRGPP
QQIYSKFGSLPIVAE-------K------SASAG-HSRNAST-PN-LQTMQHYR-P--HILQKLAEVSEPRC
LYK----PSKS-TS-QC-EAVPSDSEKSISVCNNLSELLMAMQDELDQMTMEYQD---LLNQMKETESQSVC
ENIECELEHLVKKMEIKGEQISKLMKHQDNVC--KLQQKVQNLKI-SEASAIQKEDSNCKGSKNIKNS--PR
KCLLTNS-PQK-NSNFHPIR-VHNLQMKLRRDDIMWEQ
>XP_059970653.1/1-476 centrosomal protein CEP57L1 isoform X1 [Mesoplodon densirostris]XP_059970654.1 centrosomal protein CEP57L1 isoform X1 [Mesoplodon densirostris]

---MDSELMHSIV----GSYLKPP----
-E-------RV-FVPSITQSDES-----SQTHY--SV-----NIEVTSPKMLHSPN-------SQ-------
-------ALILALKTLQEKIHRLDLERTQAEDNLKILSREAAQYKKALEDETKERNLAHEELIKQKKDISMQ
LSSAQSHCTLLEKQLEYTKRMVLNVEREKNMILEQQ------------------------------------
-----------AQLQREKEQDHMKLQAKLEKLDVLEKECFKLTTTQKTAEGKIKHLEEKLKEEEHQRKLFQD
KASQLQTGLEISRILMSSLSNPKRSKE---KKKSSK--------------------------KTKCLKGGPP
PQIYSKFGSLPIVAE-------K------SASAG-HSRNART-QN-LQTMQHYR-P--HILQKLAEVSEPRC
LYK----PSRT-TS-QC-EDVPSDSEKSISICNNLSELLMAMQDELDQMTMEYQD---LLNQMKETESQSVC
ENIECALEHLVKKMEIKGEQISKLMKHQDSVR--KLQQKVQNLKM-SEASAIQKEDSNCNGSKNIKNS--PR
KCFLTNS-PQK-SSNFRPIG-VHNLQMKLRRDDIMWEQ
>XP_057593912.1/1-478 centrosomal protein CEP57L1 isoform X3 [Hippopotamus amphibius kiboko]

---MI-----
---MDSELMHSIV----GSYLKPP----
-E-------RV-FVPSITQSDES-----SQTHY--SV-----NLEVTSPKMLHSPN-------SQ-------
-------ALILALKTLQEKIRRLELERTQAEDNLNILSREAAQYKKALENETNERNLAHEELIKQKKDISMQ
LSSAQSRCTLLEKQLEYTKRMVLNVEREKNMILEQQ------------------------------------
-----------AQLQREKEQDHMKLQAKLEKLDVLEKECFKLTTTQKIAEDKIKHLEEKLKEEEHQRKLFQD
KASQLQTGLEISRILMSSVSNPKRSKE---KKKSSK--------------------------KTKCLKRGPH
QQIYSKFRSLPIVPE-------K------SASAS-HSVNSST-QN-LETMQHYG-P--HILQKPAEVTEPGC
LYK----PSRT-TS-QC-KAVPSDSEKSISICNNLSELLMAMQNELDQMTTEYQD---LLNQMKETESQSVC
EDIECELEHLVKKMEIKGEQISRLMKHQDSVR--KLQQKVQNSKM-NEASATQQEDSNCKGSKNIKNS--PR
RCLLTNC-LQK-NSNFRPIR-VHNLQMKLRRDDIMWEQ
>KAB0355585.1/1-460 hypothetical protein FD755_021526 [Muntiacus reevesi]

---MDSELMHSIV----GSYLKPP----
-E-------RV-CVPSVTQSDES-----SQTRY--SV-----NLEVTSSKMLRSPN-------SQ-------
-------ALILALKTLQEKIHRLELERTQAEDNLNILSREAAQYKKALENETSERNLAHEELIKQKKDISMQ
LSSAQSRCIFLEKQLEYTKRMVLNVEREKNMILEQQ------------------------------------
-----------AQLQREKEQDHMKLHAKLEKLDVLEKECFKLTTTQKTAEDKIKHLEEKLKEEEHQRKLFQD
KASQLQTGLEINRILMSSVTNPRHFKE---KKKSSK--------------------------KTKCLKRRPS
QQICSKFGTLPIMAE-------K------SASAS-HSANAST-QN-LQTVPHYG-P--HILQKPAEVTELRC
LYK----PSRT-TS-QC-KAVPSDSEKSISICNNL----------------EYQD---LLNQMKETESQSVY
EDIECELERLVKKMEIKGEQISKLMQHQDSVR--KLQQKVQNSKR-SETSAIQQEDSNCKGTKNIKNS--SR
KCLLTNS-LQK-NSNFRPIQ-VHNLQMKLRRDDIMWEQ
>XP_005905906.1/1-461 PREDICTED: centrosomal protein CEP57L1 isoform X2 [Bos mutus]

---MDSELMHSIV----GSYLKPP----
-E-------RVMCVPSITQSDES-----SQTHY--SV-----NLELTSSKMLRSPN-------SQ-------
-------ALILALKTLQEKIHRLELERTQAEDNLNILSREAAQYKKALENETSERNLAHEELIKQKKDISMQ
LSSAQSRCILLEKQLEYTKRMVLNVEREKNMILEQQ----------------------------------
-----------AQLQREKEQDHMKLHAKLAKLDVLEKECFKLTTTQKTAEDKIKHLEEKLKEEEHQRKLFQD
KASQLQTGLEISRILMSSVTNPRHFKE---KKKSSK------------------------KIKCLKRRPP
QQICSKFETLPIMAE-------K-----------------------VRQHSG-P--HILQKPAEVTELRC
LYK----PSRT-TS-QC-KAVPSDSEKSISICHNLSELLMAMQDELDQMTMGYQD---LLNQMKETESQSVC
EDIECELERLVKKMEIKGEQISKLMQHQESVR--KLQQKVQNSKM-SETSAIQQEDSNCKGTKNIKNS--SR
KCLLTNS-LQK-NNNCRPVR-VHNLQMKLRRDDIMWEQ
>XP_005684649.1/1-476 PREDICTED: centrosomal protein CEP57L1 isoform X1 [Capra hircus]XP_005684650.1 PREDICTED: centrosomal protein CEP57L1 isoform X1 [Capra hircus]XP_013821896.1 PREDICTED: centrosomal protein CEP57L1 isoform X1 [Capra hircus]KAJ1058520.1 hypothetical protein K5549_008640 [Capra hircus]

---MNSELMHSIV----GSYLKPP----
-E-------RV-CVPSVTQSDES-----SQTHY--SV-----NLEVTSSKMLHSPN-------SQ-------
-------ALILALKTLQEKIHRLELERTQAEDNLNILSREAAQYKKALENETSERNLAHEELIKQKKDISMQ
LSSAQSRCILLEKQLEYTKRMVLNVEREKNMILEQQ----------------------------------
-----------AQLQREKEQDHMKLHAKLEKLDVLEKECFKLTTTQKTAEDKIKHLEEKLKEEEHQRKLFQD
KASQLQTGLEISRILMSSVTNPRHFKE---KKKSSK------------------------KTKCLKRGPP
QQICSKFGTLPIMTE-------K------SASAS-HSANAST-QN-LQTVQHSG-P--RILQKPAEVTELRC
LYK----PSRT-TS-QC-KAVPSDSEKSISICNNLSELLMAMRDELDQMTMEYQD---LLNQMKETESQSVC
EDIECELERLVKKMEIKGEQISKLMQHQDSVH--KLQQKVQNSKM-SETSAIQQEDSNCKGTKNIKNS--SR
KCLLTNS-LQK-NSNCRPIR-VHNLQMKLRRDDIMWEQ
>XP_052501864.1/1-476 centrosomal protein CEP57L1 [Budorcas taxicolor]

---MDSELMHSIV----GSYLKPP----
-E-------RV-CVPSVTQSDES-----SQTHY--SV-----NLEVTSSKMLHSPN-------SQ-------
-------ALILALKTLQEKIHRLELERTQAEDNLNILSREAAQYKKALENETSERNLAHEELIKQKKDISMQ
LRSAQSRCILLEKQLEYTKRMVLNVEREKNMILEQQ----------------------------------
-----------AQLQREKEQDHMKLHAKLEKLDVLEKECFKLTATQKTAEDKIKHLEEKLKEEEHQRKLFQD
KASQLQTGLEISRILMSSVTNPRHFKE---KKKSSK------------------------KTKCLKRGPP
QQICSKFGTLPIMAE-------K------SASAS-HSANAST-QN-LQTVQHSG-P--RILQKPAEVTELRC
LYK----PSRT-TS-QC-KAVPSDSEKSISICNNLSELLMAMQDELDQMTMEYQD---LLNQMKETESQSVC
EDIECELERLVKKMEIKGEQISKLMQHQDSVR--KLQQKVQNSKM-SETSAIQQEDSNCKGTKNIKNS--SR
KCLLTNS-LQK-NSNCRPIR-VHNLQMKLRRDDIMWEQ
>XP_040094470.1/1-478 centrosomal protein CEP57L1 isoform X1 [Oryx dammah]

---MI-----
---MDSELMHSIV----GSYLKPP----
-E-------RV-CVPSVTQSDES-----SQTHY--SV-----NLEVTSSKMLRSPN-------SQ-------
-------ALILALKTLQEKIHRLELERTQAEDNLNILSREAAQYKKALENETSERNLAHEELIKQKKDISMQ
LSSAQSRCILLEKQLEYTKRMVLNVEREKNMILEQQ----------------------------------
-----------AQLQREKEQDHMKLHAKLEKLDVLEKECFKLTTTQKTAEDKIKHLEEKLKEEEHQRKLFQD
KASQLQTGLEISRILMSSVTNPRHFKE---KKKSSK------------------------KTKCLKRGPP
QQICSKFGTLPIMAE-------K------SASAS-HSADAST-QN-LQTVQHSG-P--RILQKPAEVTELRC
LYK----PSRT-TS-QC-KAVPSDSEKSISICNNLSELLMAMQDELDQMTIEYQD---LLNQMKETESQSVC
EDIECELECLVKKMEIKGEQISKLMQHQDSVR--KLQQKVQNSKM-SETSAIQQEDSNCKGTKNIKNS--SR
KCLLTNS-LQK-NSNCRPIR-VHNLQMKLRQDDIMWEQ

>XP_020732270.1/1-476 centrosomal protein CEP57L1 isoform X1 [Odocoileus virginianus texanus]XP_020732271.1 centrosomal protein CEP57L1 isoform X1 [Odocoileus virginianus texanus]

---MDSELMHSIV----GSYLKPP----
-E-------RV-CVPSVTQSDES-----SQTRY--SV-----NLEVTSSKMLRSPN-------SQ-------
-------ALILALKTLQEKIHRLELERTQAEDNLNILSREAAQYKKALENETSERNLAHEELIKQKKDISMQ
LSSAQSRCILLEKQLEYTKRMVLNVEREKNMILEQQ------------------------------------
-----------AQLQREKEQDHMKLHAKLEKLDVLEKECFKLTTTQKTAEDKIKHLEEKLKEEEHQRKLFQD
KASQLQTGLEINRILMSSVTNPRHFKE---KKKSSK--------------------------KTKCLKRGPS
QQICSKFGTLPIMAE-------K------SARAS-HSANAST-QN-LQTVPHYG-P--HILQKPAEVTEFRC
LYK----PSRT-TS-QC-KAVPSDSEKSISICNNLSELLMAMQDELDQMTREYQD---LLNQMKETESQSVC
EDIECELERLVKKMEIKGEQISKLMQHQDSVR--KLQQTVQNSKM-SETSAIQQEDSNCKGTKNIKNS--PR
KCLLTNS-LQK-NSNFRPIR-VHNLQMKLRQDDIMWEQ
>CAI9164227.1/1-476 unnamed protein product [Rangifer tarandus platyrhynchus]CAI9701787.1 unnamed protein product [Rangifer tarandus platyrhynchus]

---MDSELMHSIV----GSYLKPP----
-E-------RV-CVPSVTQSDES-----SQTRY--SV-----NLEVTSSKMLRSPN-------SQ-------
-------ALILALKTLQEKIHRLELERTQAEDNLNILSREAAQYKKALENETSERNLAHEELIKQKKDISMQ
LSSAQSRCILLEKQLEYTKRMVLNVEREKNMILEQQ------------------------------------
-----------AQLQREKEQDHMKLHAKLEKLDVLEKECFKLTTTQKTAEDKIKHLEEKLKEEEHQRKLFQD
KASQLQTGLEINRILMSSVTNPRHFKE---KKKSSK--------------------------KTKCLKRGPS
QQICSKYGTLPIMAE-------K------SARAS-HSANAST-QN-LQTVPHYG-P--HILQKPAEVTEFRC
LYK----PSRT-TS-QC-KAVPSDSEKSISICNNLSELLMAMQDELDQMTMEYQD---LLNQMKETESQSVC
EDIDCELERLVKKMEIKGEQISKLMQHQDNVR--KLQQKVQNSKM-SETSAIQQEDSNCKGTKNIKNS--SR
KCLLTNS-LQK-NSNFRPIR-VHNLQMKLRQDDIMWEQ
>XP_043295128.1/1-478 centrosomal protein CEP57L1 isoform X1 [Cervus canadensis]XP_043746006.1 centrosomal protein CEP57L1 isoform X1 [Cervus elaphus]

---MI-----
---MDSELMHSIV----GSYLKPP----
-E-------RV-CVPSVTQSDES-----SQTRY--SV-----NLEVTSSKMLRSPN-------SQ-------
-------ALILALKTLQEKIHRLELERTQAEDNLNILSREAAQYKKALENETSERNLAHEELIKQKKDISMQ
LSSAQSRCILLEKQLEYTKRMVLNVEREKNMILEQQ------------------------------------
-----------AQLQREKEQDHMRLHAKLEKLDVLEKECFKLTTTQKTAEDKIKHLEEKLKEEEHQRKLFQD
KASQLQTGLEINRILMSSVTNPRHFKE---KKKSSK--------------------------KTKCLRRGPS
QQICSKFGTLPIVAE-------K------SASAS-HSANAST-QN-LQTVPHYG-P--HILQKPAEVTELRC
LYK----PSRT-TS-QC-KAVPSDSEKSISICNNLSELLMAMQDELDQMTMEYQD---LLNQMKETESQSVC
EDIECELERLVKKMEIKGEQISKLMQHQDSVR--KLQQKVQNSKM-SETSAIQQEDGNCKGTKNIKNS--SR
KCLLTNS-LQK-NSNFRPIR-VHNLQMKLRRDDIMWEQ
>XP_060987595.1/1-478 centrosomal protein CEP57L1 isoform X1 [Dama dama]XP_060987596.1 centrosomal protein CEP57L1 isoform X2 [Dama dama]

---MI-----
---MDSELMHSIV----GSYLKPP----
-E-------RV-CVPSVTQSDES-----SQTRY--SV-----NLEVTSSKMLRSPN-------SQ-------
-------ALILALKTLQEKIHRLELERTQAEDNLNILSREAAQYKKALENETSERNLAHEELIKQKKDISMQ
LSSAQSRCILLEKQLEYTKRMVLNVEREKNMILEQQ------------------------------------

-----------AQLQREKEQDHMKLHAKLEKLDVLEKECFKLTTTQKTAEDKIKHLEEKLKEEEHQRKLFQD
KASQLQTGLEINRILMSSVTNPRHFKE---KKKSSK-------------------------KTKCLKRGPS
QQICSKFGTLPIMAE-------K------SASAS-HSANAST-QN-LKTVPHYG-P--HILQQPAEVTELRC
LYK----PSRT-TS-QC-KAVPSDSEKSISICNNLSELLMAMQDELDQMTMEYQD---LLNQMKETESQSVC
EDIECELERLVKKMEIKGEQISKLMQHQDSVR--KLQQKVQNSKM-SETSALQQEDGNCKGTKNIKNS--SR
KCLLTNS-LQK-NSNFRPIR-VHNLQMKLRRDDIMWEQ
>KAB0346858.1/1-476 hypothetical protein FD754_011715 [Muntiacus muntjak]

---MDSELMHSIV----GSYLKPP----
-E-------RV-CVPSVTQSDES-----SQTRY--SV-----NLEVTSSKMLRSPN-------SQ-------
-------ALILALKTLQEKIHRLELERTQAEDNLNILSREAAQYKKALENETSERNLAHEELIKQKKDISMQ
LSSAQSRCIFLEKQLEYTKRMVLNVEREKNMILEQQ------------------------------------
-----------AQLQREKEQDHMKLHAKLEKLDVLEKECFKLTTTQKTAEDKIKHLEEKLKEEEHQRKLFQD
KASQLQTGLEINRILMSSVTNPRHFKE---KKKSSK-------------------------KTKCLKRRPS
QQICSKFGTLPIMAE-------K------SASAS-HSANAST-QN-LQTVPHYG-P--HILQKPAEVTELRC
LYK----PSRT-TS-QC-KAVPSDSEKSISICNNLSELLMAMQDELDQMTMEYQD---LLNQMKETESQSVC
EDIECELERLVKKMEIKGEQISKLMQHQDSVR--KLQQKVQNSKM-SETSAIQQEDSNCKGTKNIKNS--SR
KCLLTNS-LQK-NSNFRPIQ-VHNLQMKLRRDDIMWEQ
>XP_055289516.1/1-476 centrosomal protein CEP57L1 [Moschus berezovskii]XP_055289517.1 centrosomal protein CEP57L1 [Moschus berezovskii]XP_055289518.1 centrosomal protein CEP57L1 [Moschus berezovskii]

---MDSELMHSIV----GSYLKPP----
-E-------RV-CVPSVTQSDES-----SQTHY--SV-----NLEVTSSKMLRSPN-------SQ-------
-------ALILALKTLQEKIHRLELERTQAEDNLNVLSREAAQYKKALENETCERNLAHEELIKQKKDISMQ
LSSAQSRCILLEKQLEYTKRMVLNVEREKKMILEQQ------------------------------------
-----------AQLQREKEQDHMKLHAKLEKLDVLEKECFKLTATQKTAEDKIKHLEEKLKEEEHQRKLFQD
KAAQLQTGLEINRILMSSVTNPRHFKE---KKKSSK-------------------------KTKCIKRGPP
QQICSQFGTLPVTAE-------K------SASAS-HSVNAST-QN-LQTVQHYG-P--HILQKPAEVTELRC
LYK----PSRT-TS-QC-KAVPSDSEKSISVCNNLSELLMAMQDELDQMTLEYQD---LLNQMKETESQSVC
EDIACELERLVKKMEIKGEQISKLMQHQDSVR--KLQQKVQNSKM-SETSFIQQEDSNCKGTKNIKNS--SR
KCLLTNS-LQK-NSHFRPNQ-VHNLQMKLRRDDIMWEQ
>NP_001039709.1/1-476 centrosomal protein CEP57L1 [Bos taurus]XP_005210841.1 centrosomal protein CEP57L1 isoform X1 [Bos taurus]XP_027407060.1 centrosomal protein CEP57L1 isoform X1 [Bos indicus x Bos taurus]XP_027407061.1 centrosomal protein CEP57L1 isoform X1 [Bos indicus x Bos taurus]XP_059745676.1 centrosomal protein CEP57L1 isoform X1 [Bos taurus]XP_059745677.1 centrosomal protein CEP57L1 isoform X1 [Bos taurus]AAI14171.1 Chromosome 6 open reading frame 182 ortholog [Bos taurus]DAA26256.1 TPA: hypothetical protein LOC519522 [Bos taurus]

---MDSELMHSIV----GSYLKPP----
-E-------RV-CVPSITRSDES-----SQTHY--SV-----NLELTSSQMLRSPN-------SQ-------
-------ALILALKTLQEKIHRLELERTQAEDNLNILSREAAQYKKALENETSERNLAHEELIKQKKDISMQ
LSSAQSRCILLEKQLEYTKRMVLNVEREKNMILEQQ------------------------------------
-----------AQLQREKEQDHMKLHAKLAKLDVLEKECFKLTTTQKTAEDKIKHLEEKLKEEEHQRKLFQD
KASQLQTGLEISRILMSSVTNPRHFKE---KKKSSK-------------------------KIKCLKRRPP
QQICSKFETLPIMAE-------K------SASAS-HSANAST-HS-LQTVQHSG-P--HILQKPAEVTELRC
LYK----PSRT-TS-QC-KAVPSDSEKSISICHNLSELLMAMQDELDQMTMGYQD---LLNQMKETESQSVC
EDIECELERLVKKMEIKGEQISKLMQHQESVR--KLQQKVQNSKM-SETSAIQQEDSNCKGTKNIKNS--SR
KCLLTNS-LQK-NNNCRPIR-VHNLQMKLRRDDIMWEQ

>XP_010854934.1/1-476 PREDICTED: centrosomal protein CEP57L1 isoform X1 [Bison bison bison]XP_010854935.1 PREDICTED: centrosomal protein CEP57L1 isoform X1 [Bison bison bison]XP_061283627.1 centrosomal protein CEP57L1 isoform X1 [Bos javanicus]XP_061283628.1 centrosomal protein CEP57L1 isoform X1 [Bos javanicus]XP_061283629.1 centrosomal protein CEP57L1 isoform X1 [Bos javanicus]XP_061283630.1 centrosomal protein CEP57L1 isoform X1 [Bos javanicus]

--MDSELMHSIV----GSYLKPP----
-E-------RV-CVPSITQSDES-----SQTHY--SV-----NLELTSSKMLRSPN-------SQ-------
-------ALILALKTLQEKIHRLELERTQAEDNLNILSREAAQYKKALENETSERNLAHEELIKQKKDISMQ
LSSAQSRCILLEKQLEYTKRMVLNVEREKNMILEQQ------------------------------------
-----------AQLQREKEQDHMKLHAKLAKLDVLEKECFKLTTTQKTAEDKIKHLEEKLKEEEHQRKLFQD
KASQLQTGLEISRILMSSVTNPRHFKE---KKKSSK-------------------------KIKCLKRRPP
QQICSKFETLPIMAE-------K------SASAS-HSANAST-HS-LQTVQHSG-P--HILQKPAEVTELRC
LYK----PSRT-TS-QC-KAVPSDSEKSISICHNLSELLMAMQDELDQMTMGYQD---LLNQMKETESQSVC
EDIECELERLVKKMEIKGEQISKLMQHQESVR--KLQQKVQNSKM-SETSAIQQEDSNCKGTKNIKNS--SR
KCLLTNS-LQK-NNNCRPIR-VHNLQMKLRRDDIMWEQ
>XP_005905905.1/1-477 PREDICTED: centrosomal protein CEP57L1 isoform X1 [Bos mutus]ELR48999.1 Cep57-related protein [Bos mutus]

--MDSELMHSIV----GSYLKPP----
-E-------RVMCVPSITQSDES-----SQTHY--SV-----NLELTSSKMLRSPN-------SQ-------
-------ALILALKTLQEKIHRLELERTQAEDNLNILSREAAQYKKALENETSERNLAHEELIKQKKDISMQ
LSSAQSRCILLEKQLEYTKRMVLNVEREKNMILEQQ------------------------------------
-----------AQLQREKEQDHMKLHAKLAKLDVLEKECFKLTTTQKTAEDKIKHLEEKLKEEEHQRKLFQD
KASQLQTGLEISRILMSSVTNPRHFKE---KKKSSK-------------------------KIKCLKRRPP
QQICSKFETLPIMAE-------K------SASAS-HSANAST-HS-LQTVQHSG-P--HILQKPAEVTELRC
LYK----PSRT-TS-QC-KAVPSDSEKSISICHNLSELLMAMQDELDQMTMGYQD---LLNQMKETESQSVC
EDIECELERLVKKMEIKGEQISKLMQHQESVR--KLQQKVQNSKM-SETSAIQQEDSNCKGTKNIKNS--SR
KCLLTNS-LQK-NNNCRPVR-VHNLQMKLRRDDIMWEQ
>XP_019822815.1/1-476 PREDICTED: centrosomal protein CEP57L1 isoform X1 [Bos indicus]

--MDSELMHSIV----GSYLKPP----
-E-------RV-CVPSITQSDES-----SQTHY--SV-----NLELTSSKMLRSPN-------SQ-------
-------ALILALKTLQEKIHRLELERTQAEDNLNILSREAAQYKKALENETSERNLAHEELIKQKKDISMQ
LSSAQSRCILLEKQLEYTKRMVLNVEREKNMILEQQ------------------------------------
-----------AQLQREKEQDHMKLHAKLAKLDVLEKECFKLTTTQKTAEDKIKHLEEKLKEEEHQRKLFQD
KASQLQTGLEISRILMSSVTNPRHFKE---KKKSSK-------------------------KIKCLKRRPP
QQICSKFETLPIMAE-------K------SASAS-XSANAST-HS-LQTVQHSG-P--HILQKPAEVTELRC
LYK----PSRT-TS-QC-KAVPSDSEKSISICHNLSELLMAMQDELDQMTMGYQD---LLNQMKETESQSVC
EDIECELERLVKKMEIKGEQISKLMQHQESVR--KLQQKVQNSKM-SETSAIQQEDSNCKGTKNIKNS--SR
KCLLTNS-LQK-NNNCRPIR-VHNLQMKLRRDDIMWEQ
>MXQ85630.1/1-489 hypothetical protein [Bos mutus]
---MAVAKQKT----------
---LL-------------------

-------S-E--I-----
--MDSELMHSIV----GSYLKPP----
-E-------RV-CVPSITQSDES-----SQTHY--SV-----NLELTSSKMLRSPN-------SQ-------
-------ALILALKTLQEKIHRLELERTQAEDNLNILSREAAQYKKALENETSERNLAHEELIKQKKDISMQ
LSSAQSRCILLEKQLEYTKRMVLNVEREKNMILEQQ------------------------------------

-----------AQLQREKEQDHMKLHAKLAKLDVLEKECFKLTTTQKTAEDKIKHLEEKLKEEEHQRKLFQD
KASQLQTGLEISRILMSSVTNPRHFKE---KKKSSK--------------------------KIKCLKRRPP
QQICSKFETLPIMAE-------K------SASAS-HSANAST-HS-LQTVQHSG-P--HILQKPAEVTELRC
LYK----PSRI-TS-QC-KAVPSDSEKSISICHNLSELLMAMQDELDQMTMGYQD---LLNQMKETESQSVC
EDIECELERLVKKMEIKGEQISKLMQHQESVR--KLQQKVQNSKM-SETSAIQQEDSNCKGTKNIKNS--SR
KCLLTNS-LQK-NNNCRPVR-VHNLQMKLRRDDIMWEQ
>XP_055391226.1/1-476 centrosomal protein CEP57L1 [Bubalus carabanensis]XP_055391228.1 centrosomal protein CEP57L1 [Bubalus carabanensis]XP_055391229.1 centrosomal protein CEP57L1 [Bubalus carabanensis]

---MDSELMHSIV----GSYLKPP----
-E-------RV-CVPSVTQSDES-----SQTHY--SV-----NLEVTSSKMLRSPN-------SQ-------
-------ALILALRTLQEKIHRLELERTQAEDNLNILSREAAQYKKALENETSERNLAHEELIKQKKDISMQ
LSSAQSRCILLEKQLEYTKRMVLNVEREKNMILEQQ------------------------------------
-----------AQLQREKEQDHMKLHAKLAKLDVLEKECFKLTTTQKTAEDKIKHLEEKLKEEEHQRKLFQD
KASQLQTGLEISRILMSSVTNPRHFKE---KKKSSK--------------------------KTKCLKRRPP
QQICSKFGTLPIMAE-------K------SASAS-HSENAST-QS-LQTVQHSG-P--HILQKPAEVTELRC
LYK----PSRT-TS-QC-KAVHSDSEKSISICHNLSELLMAMQDELDQMTMEYQD---LLNQMKETESQSVC
EDIECELERLVKKMEIKGEQISKLMQHQETVR--KLQQKVQNSKM-SETSAIQQEDSNCKGTKNIKNS--SR
KCLLTNS-LQK-NNNCRPIR-VHNLQMKLRRDDIMWEQ
>XP_006075526.4/1-484 centrosomal protein CEP57L1 isoform X1 [Bubalus bubalis]

---M--------------------

-------T-ESVG--LI-----
---MDSELMHSIV----GSYLKPP----
-E-------RV-CVPSVTQSDES-----SQTHY--SV-----NLEVTSSKMLRSPN-------SQ-------
-------ALILALRTLQEKIHRLELERTQAEDNLNILSREAAQYKKALENETSERNLAHEELIKQKKDISMQ
LSSAQSRCILLEKQLEYTKRMVLNVEREKNMILEQQ------------------------------------
-----------AQLQREKEQDHMKLHAKLAKLDVLEKECFKLTTTQKTAEDKIKHLEEKLKEEEHQRKLFQD
KASQLQTGLEISRILMSSVTNPRHFKE---KKKSSK--------------------------KTKCLKRRPP
QQICSKFGTLPIMAE-------K------SASAS-HSENAST-QS-LQTVQHSG-P--HILQKPAEVTELRC
LYK----PSRT-TS-QC-KAVHSDSEKSISICHNLSELLMAMQDELDQMTMEYQD---LLNQMKETESQSVC
EDIECELEHLVKKMEIKGEQISKLMQHQETVR--KLQQKVQNSKM-SETSAIQQEDSNCKGTKNIKNS--SR
KCLLTNS-LQK-NNNCRPIR-VHNLQMKLRRDDIMWEQ
>XP_060275490.1/1-490 centrosomal protein CEP57L1 isoform X1 [Ovis aries]
--MPQI----------
--CL--------------------

-------T-HKAN---FWR-----
---MDSELMHSIV----GSYLKPP----
-E-------RV-CVPSVTQSDES-----SQTHY--SV-----NLEVTSSKMLHSPN-------SQ-------
-------ALILALKTLQEKIHRLELERTQAEDNLNILSREAAQYKKALENETSERNLAHEELIKQKKDISMQ
LSSAQSRCILLEKQLEYTKRMVLNVEREKNMILEQQ------------------------------------
-----------AQLQREKEQDHMKLHAKLEKLDVLEKECFKLTTTQKTAEDKIKHLEEKLKEEEHQRKLFQD
KASQLQTGLEISRILMSSVTNPRHFKE---KKKSSK--------------------------KTKCLKRGPP
QQICSKFGTLPIIAE-------K------SASAS-HSANAST-QN-LQTVQHSG-P--RILQKPAEVTELRC
LYK----TSRT-TS-QC-KAVPSDSEKSISIYNNLSELLMAMQDELDQMTMEYQD---LLNQMKETESQSVC
EDIECELERLVKKMEIKGEQISKLMQHQDSVR--RLQQKVQNSKM-SKTSAIQQEDSNCKGTKNIKNS--SR
KCLLTNS-LQK-NSNCRPIR-VHNLQMKLRRDDIMWEQ
>XP_031536026.1/1-486 centrosomal protein CEP57L1 isoform X1 [Vicugna pacos]

---MQ-------------------

-------------------ESSNSNR-------------------------------------DAMI-----
---MDSELMHSIV----GSYLRPP----
-E-------RV-FVPSFTQSDES-----S--HY--SM-----NLEVTATKMLHSPN-------SQ-------
-------AIILALKTLQEKIHRLELERTQAEDNLNVISREAAQYKKALENETNERNLAHQELIKQKKDISMQ

LSSAQSRCTLLEKQLEYTKRMVLNVEREKNMILEQQ------------------------------------
-----------AQLQREKEQDHMKLQAKLEKLDVLEKECFKLTTTQKTSEDKIKHLEEKLKEEEHQRKLFQD
KASQLQTGLEINRILMSSASNPKHFNE---KKKSSK--------------------------KTKCLKRGPP
QQIYSKFGSQPIVAE-------K------SASAS-HSLNPSM-QNLLQTTQHYR-T--HTLQKPAEVAEPAC
LHK----PSRT-TS-QC-KALPSDSEKSISICNNLSELLMAMQDELDQMSMEYQE---LLNQIKETESQSVC
EDIECKLEHLAKKMEIKGEQISKLMKHQDSVH--KLQQKVYNSKM-SETSAIQQEDGNCKGSKNIKN----R
NCWLSNS-LQK-NSNFRPIR-VHNLQMKLRRDDIMWEQ
>XP_032340562.1/1-488 centrosomal protein CEP57L1 isoform X1 [Camelus ferus]XP_032340563.1 centrosomal protein CEP57L1 isoform X1 [Camelus ferus]

---MQ-------------------

-------------------ESSNSNR-------------------------------------DAMI-----
---MDSELMHSIV----GSYLRPP----
-E-------RV-FVPSFTQSDES-----S--HY--SM-----NLEVTATKMLHSPN-------SQ-------
-------AIILALKTLQEKIHRLELERTQAEDNLNVISREAAQYKKALENETNERNLAHQELIKQKKDISMQ
LSSAQSRCTLLEKQLEYTKRMVLNVEREKNMILEQQ------------------------------------
-----------AQLQREKEQDHMKLQAKLEKLDVLEKECFKLTTTQKTSEDKIKHLEEKLKEEEHQRKLFQD
KASQLQTGLEINRILMSSASNPKHFNE---KKKSSK--------------------------KTKCLKRGPP
QQIYSKFGSQPIVAE-------K------SASAS-HSLNPSM-QNLLQTTQHYR-T--HTLQKPAEVAEPAC
LHK----PSRT-TS-QC-KALHSDSEKSISICNNLSELLMAMQDELDQMSMEYQE---LLNQIKETESQSVC
EDIECKLERLVKKMEIKGEQISKLMKHQDSVH--KLQQKVHNSKM-SETSAIQQEDGNCKGSKNIKNS--PR
NCWLSNS-LQK-NSNFRPIQ-VHNLQMKLRRDDIMWEQ
>XP_031311996.1/1-488 centrosomal protein CEP57L1 isoform X1 [Camelus dromedarius]

---MQ-------------------

-------------------ESSNSNR-------------------------------------DAMI-----
---MDSELMHSIV----GSYLRPP----
-E-------RV-FVPSFTQSDES-----S--HY--SM-----NLEVTATKMLHSPN-------SQ-------
-------AIILALKTLQEKIHRLELERTQAEDNLNVISREAAQYKKALENETNERNLAHQELIKQKKDISMQ
LSSAQSRCTLLEKQLEYTKRMVLNVEREKNMILEQQ------------------------------------
-----------AQLQREKEQDHMKLQAKLEKLDVLEKECFKLTTTQKTSEDKIKHLEEKLKEEEHQRKLFQD
KASQLQTGLEINRILMSSASNPKHFNE---KKKSSK--------------------------KTKCLKRGPP
QQIYSKFGSQPIVAE-------K------SASTS-HSLNPSM-QNLLQTTQHYR-T--HTLQKPAEVAEPAC
LHK----PSRT-TS-QC-KALHSDSEKSISICNNLSELLMAMQDELDQMSMEYQE---LLNQIKETESQSVC
EDIECKLERLVKKMEIKGEQISKLMKHQDSVH--KLQQKVHNSKM-SETSAIQQEDGNCKGSKNIKNS--PR
NCWLSNS-LQK-NSNFRPIQ-VHNLQMKLRRDDIMWEQ
>XP_010963698.2/1-473 LOW QUALITY PROTEIN: centrosomal protein CEP57L1 [Camelus bactrianus]

---MQ-------------------

-------------------ESSNSNR-------------------------------------DAMI-----
---MDSELMHSIV----GSXIRPP----
-------------------------------XY--SM-----NLEVTATKMLHSPN-------SQ-------
-------AIILALKTLQEKIHRLELERTQAEDNLNVISREAAQYKKALENETNERNLAHQELIKQKKDISMQ
LSSAQSRCTLLEKQLEYTKRMVLNVEREKNMILEQQ------------------------------------
-----------AQLQREKEQDHMKLQAKLEKLDVLEKECFKLTTTQKTSEDKIKHLEEKLKEEEHQRKLFQD
KASQLQTGLEINRILMSSASNPKHFNE---KKKSSK--------------------------KTKCLKRGPP
QQIYSKFGSQPIVAE-------K------SASAS-HSLNPSM-QNLLQTTQHYR-T--HTLQKPAEVAEPAC
LHK----PSRT-TS-QC-KALHSDSEKSISICNNLSELLMAMQDELDQMSMEYQE---LLNQIKETESQSVC
EDIECKLERLVKKMEIKGEQISKLMKHQDSVH--KLQQKVHNSKM-SETSAIQQEDGNCKGSKNIKNS--PR
NCWLSNS-LQK-NSNFRPIQ-VHNLQMKLRRDDIMWEQ
>XP_013848197.1/1-487 centrosomal protein CEP57L1 isoform X2 [Sus scrofa]
---MDGRK----------
--EL--------------------

-------H-K--I-----
---MDSELMHSIV----GSYLNPP----

-E-------RV-FVPSFTQSNES-----SQTHY--SV-----NLEVTSPKMLRSPN-------NQ-------
-------ALILALKTLQEKIYRLELERTQAEDNLNILSREAAQYKKALENETSERNLAHEELIKQKKDISMQ
LSSAQSRCTLLEKQLEYTKRMVLNVEREKDMILEQQ------------------------------------
-----------AQLQREKEQDHMKLQAKLEKLDVLEKECFKLTTTQKTAEDKIKHLEEKLKEEEHQRKLFQD
KASQLQTGLAINRILMASASNPKCSKE---KKKSSK-------------------------KTKCLKRGPP
QQIYSKFGSLPVVAE-------K------SASAS-HSVNAST-QNLLQTMQHYG-P--HILQKPAKVIEPRY
LYK----PSRT-TS-QC-KAVPSDSEKSISICDNLSELLMAMQDELDQMSIEYQE---LLSQMKETESQSVC
EDIECELEHLVKKMEIKGERISKLMKHQDSVR--KLQQKVQSSKM-SEASAIQQEDSNLKGSKNIKNS--PR
KCLLTNS-LQK-NSSFHPIR-VHNLQMKLRRDDILWEQ
>XP_047618303.1/1-519 centrosomal protein CEP57L1 isoform X1 [Phacochoerus africanus]
--MVGKS----------
---CV-------------------

-------R-SGKRDAVET-SAPRCYLC------FCSVRWAHPG-----------------WTQYPCI-----
--MDSELMHSIV----GNYLNPP----
-E-------RV-FVPSFTQSNES-----SQTHY--SV-----NLEVTSPKMLRSPN-------NQ-------
-------ALILALKTLQEKIYRLELERTQAEDNLNILSREAAQYKKALENETSERNLAHEELIKQKKDISMQ
LSSAQSRCTLLEKQLEYTKRMVLNVEREKDMILEQQ------------------------------------
-----------AQLQREKEQDHMKLQAKLEKLDVLEKECFKLTTTQKTAEDKIKHLEEKLKEEEHQRKLFQD
KASQLQTGLAINRILMASVSNPKCSKE---KKKSSK-------------------------KTKCLKRGPP
QQIYSKFGSLPVVAE-------K------SASAS-HSVNAST-QNLLQTMQHYG-P--HILQKPAKVIEPRY
LYK----PSRT-TS-QC-KAVPSDSEKSISICDNLSELLMAMQDELDQMSIEYQE---LLSQMKETESQSVC
EDIECELEHLVKKMEIKGERISKLMKHQDSVR--KLQQKVQSSKM-SEASAIQQQDSNLKGSKNIKNS--PR
KCLLTNS-LQK-NSSFHPIR-VHNLQMKLRRDDILWEQ
>XP_013848196.1/1-519 centrosomal protein CEP57L1 isoform X1 [Sus scrofa]
--MVGKS----------
---CI-------------------

-------R-SGKRDAVET-SAPRCYLC------FCSVRWAHAG-----------------WTQYPCI-----
--MDSELMHSIV----GSYLNPP----
-E-------RV-FVPSFTQSNES-----SQTHY--SV-----NLEVTSPKMLRSPN-------NQ-------
-------ALILALKTLQEKIYRLELERTQAEDNLNILSREAAQYKKALENETSERNLAHEELIKQKKDISMQ
LSSAQSRCTLLEKQLEYTKRMVLNVEREKDMILEQQ------------------------------------
-----------AQLQREKEQDHMKLQAKLEKLDVLEKECFKLTTTQKTAEDKIKHLEEKLKEEEHQRKLFQD
KASQLQTGLAINRILMASASNPKCSKE---KKKSSK-------------------------KTKCLKRGPP
QQIYSKFGSLPVVAE-------K------SASAS-HSVNAST-QNLLQTMQHYG-P--HILQKPAKVIEPRY
LYK----PSRT-TS-QC-KAVPSDSEKSISICDNLSELLMAMQDELDQMSIEYQE---LLSQMKETESQSVC
EDIECELEHLVKKMEIKGERISKLMKHQDSVR--KLQQKVQSSKM-SEASAIQQEDSNLKGSKNIKNS--PR
KCLLTNS-LQK-NSSFHPIR-VHNLQMKLRRDDILWEQ
>XP_032959462.1/1-486 centrosomal protein CEP57L1 isoform X7 [Rhinolophus ferrumequinum]XP_032959463.1 centrosomal protein CEP57L1 isoform X7 [Rhinolophus ferrumequinum]XP_032959464.1 centrosomal protein CEP57L1 isoform X7 [Rhinolophus ferrumequinum]XP_032959465.1 centrosomal protein CEP57L1 isoform X7 [Rhinolophus ferrumequinum]XP_032959467.1 centrosomal protein CEP57L1 isoform X7 [Rhinolophus ferrumequinum]XP_032959468.1 centrosomal protein CEP57L1 isoform X7 [Rhinolophus ferrumequinum]XP_032959469.1 centrosomal protein CEP57L1 isoform X7 [Rhinolophus ferrumequinum]

--MI-----
--MDSELMHSIV----GSYLKPP----
-E-------RV-FVPSFIQSDES-----SQNHY--SV-----NFGVSAVKMLPSPN-------SQ-------
-------ALILALKTLQEKIHRLELERTQAEDNLNILSREAAQYKQALENETNERNLVHQELIKQKKDISIQ
LSSAQSRCTLLEKQLEYTKRMVLNVEREKNMILEQQ------------------------------------
-----------AQLQREKEQDHMKLQAKLEKLDVLEKECFKLTTTQKTAEDKIKHLEEKLKEKEHQRKLFQD
KASELQTGLEINRILMSSVSNPKRSRE---KKKSSK-------------------------KTKCLKRGAS
QQVYSKFGSLPIVAEKVRGAKTK------SASAS-HSVNASM-QNLLQMMQHYG-P--HILQKPPAVTEPRC
LYK----PTRT-TS-HC-KAVPPDPEKSISICDNLSELLMAMQDEFDQMSMEHRK---LLNQMEETESRSVC
EDIECELEHLVKKMEIKGEQISKLMKHQDSVR--KLQQKIQNSKM-SDASGIQREDSNPKGSKNIKNS--PR

KCLLTNS-PQK-NSNFHPIR-VHNLQMKLRRDDIMWEQ
>XP_019590544.1/1-486 PREDICTED: centrosomal protein CEP57L1 isoform X1 [Rhinolophus sinicus]XP_019590545.1 PREDICTED: centrosomal protein CEP57L1 isoform X1 [Rhinolophus sinicus]XP_019590546.1 PREDICTED: centrosomal protein CEP57L1 isoform X1 [Rhinolophus sinicus]

---MI-----
---MDSELMHSIV----GSYLKPP----
-E-------RV-CVPSFIQSDES-----SQNHH--SV-----NFGVTAAKMLPTPN-------SQ-------
-------ALILALKTLQEKIHRLELERTQAEDNLNILSREAAQYKQALEIETNERNLVHQELIKQKKDISIQ
LSSAQCRCTFLEKQLEYTKRMVLNVEREKNMILEQQ------------------------------------
-----------AQLQREKEQDHMKLQAKLEKLDVLEKECFKLTTTQKTAEDKIKHLEEKLKEKEHQRKLFQD
KASELQTGLEINRILMSSVSNPKRSRE---KKKSAK--------------------------KPKCLKRGAS
QQVCSKFGSLPVVAEKVRGAKTK------SASAS-RSVNASM-QNLLQMMQHYG-P--HILQKPPEVTEPRC
LYK----PTRK-TS-HC-KAVPPDPEKSISICDNLSELLMAMQDELDQMSMEHRK---LLNQMEETESRSVC
EDIECELEHLVKKMEIKGEQISKLMKHQDSVR--KLQQKFQNSKM-SDTSGIQREDSNPKGSKNIKNS--PR
KCLLTNS-LQK-NSNFHPIR-VHNLQMKLRRDDIMWEQ
>XP_032959458.1/1-513 centrosomal protein CEP57L1 isoform X3 [Rhinolophus ferrumequinum]
--MPGV
L--EDGCLE-------------------
---VW---------------
-------S-H----LVFW-ITDVK--------------------------------------WSGYI-----
---MDSELMHSIV----GSYLKPP----
-E-------RV-FVPSFIQSDES-----SQNHY--SV-----NFGVSAVKMLPSPN-------SQ-------
-------ALILALKTLQEKIHRLELERTQAEDNLNILSREAAQYKQALENETNERNLVHQELIKQKKDISIQ
LSSAQSRCTLLEKQLEYTKRMVLNVEREKNMILEQQ------------------------------------
-----------AQLQREKEQDHMKLQAKLEKLDVLEKECFKLTTTQKTAEDKIKHLEEKLKEKEHQRKLFQD
KASELQTGLEINRILMSSVSNPKRSRE---KKKSSK--------------------------KTKCLKRGAS
QQVYSKFGSLPIVAEKVRGAKTK------SASAS-HSVNASM-QNLLQMMQHYG-P--HILQKPPAVTEPRC
LYK----PTRT-TS-HC-KAVPPDPEKSISICDNLSELLMAMQDEFDQMSMEHRK---LLNQMEETESRSVC
EDIECELEHLVKKMEIKGEQISKLMKHQDSVR--KLQQKIQNSKM-SDASGIQREDSNPKGSKNIKNS--PR
KCLLTNS-PQK-NSNFHPIR-VHNLQMKLRRDDIMWEQ
>XP_019514536.1/1-479 PREDICTED: centrosomal protein CEP57L1 isoform X3 [Hipposideros armiger]XP_019514537.1 PREDICTED: centrosomal protein CEP57L1 isoform X3 [Hipposideros armiger]XP_019514538.1 PREDICTED: centrosomal protein CEP57L1 isoform X3 [Hipposideros armiger]XP_019514539.1 PREDICTED: centrosomal protein CEP57L1 isoform X3 [Hipposideros armiger]

---MI-----
---MDSELMHSIV----GSYLKPP----
-E-------RV-FVPSFTQIDES-----SQNHH--SV-----NFGVTAPKMLHSPN-------SQ-------
-------ALILALKSLQEKIHRLELERTQAEDNLNILSREAAQYKKALEIETNERNLVHQELIKQKKDISIQ
LSSAQSRCTLLEKQLEYTKRMVLNVEREKNMILEQQ------------------------------------
-----------AQLQREKEQDHMKLQAKLEKLDVLEKECFKLTTTQKSAEDKIKHLEEKLKEEEHQRKLFQD
KASELQTGLEINRILMSSASIPKGSKE---KKKSSK--------------------------KTKCLKRGTS
QQVYSKFGSLPVVAE-------K------SASAS-HSVNASM-QNLLQLMQHYG-P--HILRKPAEVAEPRC
LYK----PTRT-VS-QC-KAVPPDPEKSVSICDNLSELLMAMQDELDQMSMEHQK---LLNQMEETESRSVC
EDIECELEHLVKKMEIKGEQISKLMKHQDSVR--KLQQKVQNSKM-SDASCIQQEDSNPKESKNIKNS--PR
KCSLTNS-LQK-NGNFHPVR-VHNLQMKLRRDDIMWEQ
>XP_032959459.1/1-501 centrosomal protein CEP57L1 isoform X4 [Rhinolophus ferrumequinum]
--MLLARSVCDRNYSPHK
P--LRRCL--------------------
--W---------------
-------R-RASRPLARW-ETES--VSLI-----
---MDSELMHSIV----GSYLKPP----

-E-------RV-FVPSFIQSDES-----SQNHY--SV-----NFGVSAVKMLPSPN-------SQ-------
-------ALILALKTLQEKIHRLELERTQAEDNLNILSREAAQYKQALENETNERNLVHQELIKQKKDISIQ
LSSAQSRCTLLEKQLEYTKRMVLNVEREKNMILEQQ------------------------------------
-----------AQLQREKEQDHMKLQAKLEKLDVLEKECFKLTTTQKTAEDKIKHLEEKLKEKEHQRKLFQD
KASELQTGLEINRILMSSVSNPKRSRE---KKKSSK-------------------------KTKCLKRGAS
QQVYSKFGSLPIVAE--------------------------------KMMQHYG-P--HILQKPPAVTEPRC
LYK----PTRT-TS-HC-KAVPPDPEKSISICDNLSELLMAMQDEFDQMSMEHRK---LLNQMEETESRSVC
EDIECELEHLVKKMEIKGEQISKLMKHQDSVR--KLQQKIQNSKM-SDASGIQREDSNPKGSKNIKNS--PR
KCLLTNS-PQK-NSNFHPIR-VHNLQMKLRRDDIMWEQ
>XP_032959456.1/1-525 centrosomal protein CEP57L1 isoform X1 [Rhinolophus ferrumequinum]
--MLLARSVCDRNYSPHK
P--LRRCL--------------------
--W---------------
-------R-RASRPLARW-ETES---VSLI-----
---MDSELMHSIV----GSYLKPP----
-E-------RV-FVPSFIQSDES-----SQNHY--SV-----NFGVSAVKMLPSPN-------SQ-------
-------ALILALKTLQEKIHRLELERTQAEDNLNILSREAAQYKQALENETNERNLVHQELIKQKKDISIQ
LSSAQSRCTLLEKQLEYTKRMVLNVEREKNMILEQQ------------------------------------
-----------AQLQREKEQDHMKLQAKLEKLDVLEKECFKLTTTQKTAEDKIKHLEEKLKEKEHQRKLFQD
KASELQTGLEINRILMSSVSNPKRSRE---KKKSSK-------------------------KTKCLKRGAS
QQVYSKFGSLPIVAEKVRGAKTK------SASAS-HSVNASM-QNLLQMMQHYG-P--HILQKPPAVTEPRC
LYK----PTRT-TS-HC-KAVPPDPEKSISICDNLSELLMAMQDEFDQMSMEHRK---LLNQMEETESRSVC
EDIECELEHLVKKMEIKGEQISKLMKHQDSVR--KLQQKIQNSKM-SDASGIQREDSNPKGSKNIKNS--PR
KCLLTNS-PQK-NSNFHPIR-VHNLQMKLRRDDIMWEQ
>XP_019514533.1/1-517 PREDICTED: centrosomal protein CEP57L1 isoform X1 [Hipposideros armiger]
---MLARSVCDRDYSPHK
P--LRRCL--------------------
--W---------------
-------R-PGSRPLARS-ETES---VSLI-----
---MDSELMHSIV----GSYLKPP----
-E-------RV-FVPSFTQIDES-----SQNHH--SV-----NFGVTAPKMLHSPN-------SQ-------
-------ALILALKSLQEKIHRLELERTQAEDNLNILSREAAQYKKALEIETNERNLVHQELIKQKKDISIQ
LSSAQSRCTLLEKQLEYTKRMVLNVEREKNMILEQQ------------------------------------
-----------AQLQREKEQDHMKLQAKLEKLDVLEKECFKLTTTQKSAEDKIKHLEEKLKEEEHQRKLFQD
KASELQTGLEINRILMSSASIPKGSKE---KKKSSK-------------------------KTKCLKRGTS
QQVYSKFGSLPVVAE-------K------SASAS-HSVNASM-QNLLQLMQHYG-P--HILRKPAEVAEPRC
LYK----PTRT-VS-QC-KAVPPDPEKSVSICDNLSELLMAMQDELDQMSMEHQK---LLNQMEETESRSVC
EDIECELEHLVKKMEIKGEQISKLMKHQDSVR--KLQQKVQNSKM-SDASCIQQEDSNPKESKNIKNS--PR
KCSLTNS-LQK-NGNFHPVR-VHNLQMKLRRDDIMWEQ
>XP_012575795.1/1-479 PREDICTED: centrosomal protein CEP57L1 [Condylura cristata]

---MDSELMHSIV----GSYFKPP----
-E-------RV-FVPSFTQNKEA-----SQNHH--SV-----NLDVNATKMLHSPN-------SQ-------
-------ALVLALKSLQEKIHRLELERTQAEDNLNILSREAAQYKKALEDETNERNLAHQELIKQKKDISIQ
LSSAQSRCILLEKQLEYTKRMVLNVEREKSMILEQQ------------------------------------
-----------AQLQREKEQDHMKLQAKLEKLDILEKECFKLTTTQKTAEDKIKHLEEKLKEEEHQRKLFQD
KASELQTGLEINRILMSSVSNPKCCKE---KKKSSK-------------------------KTKCLKKEPP
QQSYSKFGSLPFGTE---EGKMK------TTSANLHSTNTGM-QNLLEMMQHYG-P--RNLQKPADMTKPRC
LNE----P----TS-SC-KTILPDSKKSISTCDSLSELLMAMQDELDQMSMEHQD---LLNQMKDTESHLVS
EDIENELQHLVKKMRIKGEQISKLKKHQDSVR--KLQQKVHTSMM-NEASSIQREDSSPKASKDIRRS--PR
KCLLTNP-LQK-NSNFHPIQ-VHNLQMKLRKDDIMWEQ
>XP_039725947.1/1-460 centrosomal protein CEP57L1 isoform X1 [Pteropus giganteus]XP_039725952.1 centrosomal protein CEP57L1 isoform X1 [Pteropus giganteus]XP_039725962.1 centrosomal protein CEP57L1 isoform X1 [Pteropus giganteus]XP_039725971.1 centrosomal protein CEP57L1 isoform X1 [Pteropus

giganteus]XP_039725980.1 centrosomal protein CEP57L1 isoform X1 [Pteropus giganteus]XP_039725990.1 centrosomal protein CEP57L1 isoform X1 [Pteropus giganteus]

---------------------------------------MDSELMHSIV----GSYLKPP----
-E-------SV-FVPSFTENDES-----SQNHH--SV-----NFEVTSPKMLHSPN-------SQ-------
-------ALILALKTLQEKIHRLELERTQAEDNLNILSREAAQYKKALEIETNERNLVHQELIKQKKDISIQ
LSSAQSRCTLLEKQLEYTKRMVLNVEREKNIILEQQ------------------------------------
----------AQLQREKEQDHMKLQAKLEKLDVLEKECFKLTATQKTAEDKIKHLEEKLKEEEHHRKLFQD
KASELQTGLEINRILMSSVSNPKRSKE---KKKSSK-------------------------KTKCLKREPP
QQIYSKFESPPIAAE-------K------------------------MVQHYG-P--HILQKPAEVIERRC
LYK----PTRT-TS-QC-KAVAPDSKKSISICDNLSELLVAMQDELDQMSIEHQE---LLNQMKEAESHSVC
EDLECELEHLVKKMKIKEEQISKLLKHQDSVR--KLQQKVQNPKM-SEASGIQREDNNPKGSKNIKNS--PR
KCLLTNS-LQK-NSNFHPIR-VHNLQMKLRRDDIMWEQ
>KAF6504361.1/1-477 centrosomal protein 57 like 1 [Rousettus aegyptiacus]

---------------------------------------MDSELMHSIV----GSYLKPP----
-E-------SV-FVPSFTKNDES-----SENHH--SV-----NFDVTSPKMLHSPN-------SQ-------
-------ALILALKTLQEKIHRLELERTQAEDNLNILSREAAQYKKALETETNERNLVHQELIKQKKDISIQ
LSSAQSRCTLLEKQLEYTKRMVLNVEREKNIILEQQ------------------------------------
----------AQLQREKEQDHMKLQAKLEKLDVLEKECFKLTATQKSAEDKIKHLEEKLKEEEHHRKLFQN
KASELQTGLEINRILMSSVSNPKRSKE---KKKSSK-------------------------KTKCLKREPP
QQIYSKFESPHIAAE-------K------SASAS-HSVNASI-QNLLQMVQHYG-P--HILQKPAEVIECRC
LYK----PTRA-AS-QH-KAVAPDSKKSIPICDNLSELLVAMQDELDQMSIEHQE---LLNQMKEAESHSVC
EDLECELEHLVKKMKIKEEQISKLLKHQDSIH--KLQQKIQNPKM-SEASGVQREDNKPKGSKNIKNS--PR
NCLLTNS-LQK-NSDFHPVR-VHNLQMKLRRDDIMWEQ
>XP_011363144.1/1-501 centrosomal protein CEP57L1 isoform X1 [Pteropus vampyrus]
---MLPAHSICDRNYSPQE
P---------------------------------------LRRCL-------------------
---W--------------
-------R-PAIQPLART-VFESVG-------------------------------------LI-----
---------------------------------------MDSELMHSIV----GSYLKPP----
-E-------SV-FVPSFTENDES-----SQNHH--SV-----NFEVTSPKMLHSPN-------SQ-------
-------ALILALKTLQEKIHRLELERTQAEDNLNILSREAAQYKKALEIETNERNLVHQELIKQKKDISIQ
LSSAQSRCTLLEKQLEYTKRMVLNVEREKNIILEQQ------------------------------------
----------AQLQREKEQDHMKLQAKLEKLDVLEKECFKLTATQKTAEDKIKHLEEKLKEEEHHRKLFQD
KASELQTGLEINRILMSSVSNPKRSKE---KKKSSK-------------------------KTKCLKREPP
QQIYSKFESPPVAAE-------K------------------------MVQHYG-P--HILQKPAEVIERRC
LYK----PTRT-TS-QC-KAVAPDSKKSISICDNLSELLVAMQDELDQMSIEHQE---LLNQMKEAESHSVC
EDLECELEHLVKKMKIKEEQISKLLKHQDSVR--KLQQKVQNPKM-SEASGIQREDNNPKGSKNIKNS--PR
KCLLTNS-LQK-NSNFHPIR-VHNLQMKLRRDDIMWEQ
>ELK00625.1/1-505 Cep57-related protein [Pteropus alecto]
---MAPEE---------EE
K---------------------------------------LQEILIHACPK-------------
--SSNNLGNW---------------
-------K-TVLDHNNESELHQRQD-------------------------------------NI-----
---------------------------------------MDSELMHSIV----GSYLKPP----
-E-------SV-FFPSFTENDES-----SQNHH--SV-----NFEVTSPKMLHSPN-------SQ-------
-------ALILALKTLQEKIHRLELERTQAEDNLNILSREAAQYKKALEIETNERNLVHQELIKQKKDISIQ
LSSAQSRCTLLEKQLEYTKRMVLNVEREKNIILEQQ------------------------------------
----------AQLQREKEQDHMKLQAKLEKLDVLEKECFKLTATQKTAEDKIKHLEEKLKEEEHHRKLFQD
KASELQTGLEINRILMSSVSNPKRSKE---KKKSSK-------------------------KTKCLKREPP
QQIYSKFESPPVAAE-------K------------------------MVQHYG-P--HILQKPAEVIERRC
LYK----P-RT-TS-QC-KAVAPDSKKSISICDNLSELLVAMQDELDQMSIEHQE---LLNQMKEAESHSVC

EDLECELEHLVKKMKIKEEQISKLLKHQDSVR--KLQQKVQNPKM-SEASGIQREDNNPKGSKNIKNS--PR
KCLLTNS-LQK-NSNFHPIR-VHNLQMKLRRDDIMWEQ
>XP_042531801.1/1-474 centrosomal protein CEP57L1 isoform X1 [Dipodomys spectabilis]XP_042531890.1 centrosomal protein CEP57L1 isoform X1 [Dipodomys spectabilis]

--MDSELVHSVV----GSYLKPP----
-E-------RV-FAPPFTQNESS------QSCY--PL-----NLEVISPKMLQSPN-------SQ-------
-------ALILALKSLQEKIRRLELERTQAEDNLNILSREAAEYKKALENETNKRNLAHQELIKQKKDISIQ
LSAAQSRCTLLEKQLEYTKRMVLNVEREKTMILEQQ------------------------------------
-----------AQLQKEKEQDQMKLHAKLEKLNVLEKECIRLTTTQKTAEDKIKYLEEKLREEEHHRKLFQD
KASKLQTGLEISKILMSSVSNSKLSRE---KKKSSK-------------------------KT--LKRSPS
QQMYSKLRAPPFVPE-------K------SASAG-YSVNAGI-QTLLQMMYHHG-P--HH-QKPVQVIEPRR
LHK----PART-AS-QG-KTVPCDSGKSISVCDNLSEILMAMEDELDQMNMEREE---LLKQMKESQSHSVC
DDIECELEHLIKKMEIKGEQISKLKKHQDSVQ--KLQQKVQNSKI-NEASGIHREESNPKGTKNIKNS--SR
KCLNETNPFQK-NSSSHPVQ-VHNVQVKLRRDDIMWEQ
>XP_012877800.1/1-474 PREDICTED: centrosomal protein CEP57L1 isoform X1 [Dipodomys ordii]XP_012877801.1 PREDICTED: centrosomal protein CEP57L1 isoform X1 [Dipodomys ordii]XP_012877802.1 PREDICTED: centrosomal protein CEP57L1 isoform X1 [Dipodomys ordii]

--MDSELVHSVV----GSYLKPP----
-E-------RV-FAPPFTQNESS------QSCY--PL-----NLEVISPKMLQSPN-------SQ-------
-------ALILALKSLQEKIRRLELERTQAEDNLNILSREAAEYKKALENETNKRNLAHQELIKQKKDISIQ
LSAAQSRCTLLEKQLEYTKRMVLNVEREKTVILEQQ------------------------------------
-----------AQLQKEKEQDQMKLHAKLEKLNVLEKECIRLTTTQKTAEDKIKYLEEKLREEEHHRKLFQD
KASKLQTGLEISKILMSSVSNSKLSKE---KKKSSK-------------------------KT--LKRGPS
QQMYAKLRAPPFVPE-------K------SASAG-YSVNAGI-QTLLQMMYHHG-P--HH-QKPIQVIEPRR
LHK----PART-AS-QG-KTVPCDSGKSISICDNLSEILMAMEDELDQMNMEREE---LLKQMKESQSHSVC
DDIECELEHLIKKMEIKGEQISKLKKHQDSVQ--KLQQKVQNSKI-NEASGIHREESNPKGTKNIKNS--SR
KCLNETNPFQK-NSSSHPVQ-VHNVQVKLRRDDIMWEQ
>XP_048209683.1/1-474 centrosomal protein CEP57L1 [Perognathus longimembris pacificus]XP_048209684.1 centrosomal protein CEP57L1 [Perognathus longimembris pacificus]XP_048209685.1 centrosomal protein CEP57L1 [Perognathus longimembris pacificus]

--MDSELIHSIV----GSYLKPP----
-E-------RV-FVPPCTQNEPS------QSCY--PV-----NLEVISPKMLQSPN-------SQ-------
-------ALILALKTLQEKIRRLELERTQAEDNLNILSKEAAEYKKALENETNERNLAHQELIKQKKDISIQ
LSAAQSRCTLLEKQLEYTKRMVLNVEREKTVILEQQ------------------------------------
-----------AQLQKEKEQDQMKLHAKLEKLDVLEKECFKLTTTQKTAEDKIKYLEQKLREEEHHRKLFQD
KASKLQTGLEISKMLMSSVSNSKLPKE---KKKSSK-------------------------KT--LKRGPS
QQMYSKLRAPPFVPE-------K------STSAS-CSVNAGI-QTLLQMMHHHV-P--HH-QKPAQVAEPRR
LHK----PARA-TS-QG-KAAPRDSGKATSICDNLSELLMAMQDELDQMNVEREE---LLKQMRETESHSVC
DDIECELEHLVKKMEIKGEQISKLKKHQDSVQ--KLQQKVQNSKI-SEASGIHREDSSPKGTKNIKNS--SR
KSLNETNPFQK-NSSSHPVQ-VHNVRVKLRRDDITWEQ
>XP_020030938.1/1-469 centrosomal protein CEP57L1 [Castor canadensis]

--MDSELIHSVV----GSYLQPP----

-E-------QV-FAPSFIQNESS------QNCH--SV-----NLEVPSPKMLQSPN-------SQ-------
-------ALVLALKTLQEKIHRLELERTQAEDNLNILSREAAQYKKALENETNERNLAHQELIKQKKDISMQ
LSSAQSRCILLEKQLEYTKRMVLNVEREKTMILEQQ------------------------------------
-----------AQLQREKEQDQMKLHAKLEKLDVLEKECFRLTTTQKTAEDKIKYLEEKLKEEEHHRKLFQD
KA--------LSKILMSSVTNSKSSKE---KKKSSK-------------------------KSACLKKGPP
QQTYSKFRAPTFVSE-------K------SASAG-CSMNANM-QNLLQMMQQCG-L--HSLQKPVQVTERRC
LYK----PTRT-TF-QC-QAVPCDSEKSISICDNLSELLMAMQDELDQMSMEHEE---LLKQMKKTESHSVY
DDIECELGHLLKKMEIKGEQISKLKKHQDSVR--KLQQKVQDSKI-SEALGFQREDSNPKGSKNIKNN--SR
KCLNETNPFQK-NISFHPMQ-VHNLRVKLRRDDITWEQ
>XP_047413851.1/1-480 centrosomal protein CEP57L1 isoform X1 [Sciurus carolinensis]

---MGS-----------------

------------------RVQ--I-----
--MNSELIHSIV----GSYLKPP----
-E-------EV-FAPSFTQDES------SQNCH--TV-----NLEVTSPKMLHSPN-------NQ-------
-------ALILALKTLQEKIHHLELERTQAEDNLNILSREAAQYKKALENETNERNLAHQELIKQKKDISLQ
LSSAQSRCTLLEKQLEYTKRMVLNVEREKNMILEQQ------------------------------------
-----------AQLQREKEQDQLRLHAKLEKLDVLEKECFRLTTTQKTAEDKIKHLEEKLKEEEHHRKLFQD
KASELQTGLEISKILMSSVSKSKLSKG---KKKSSK-------------------------KTKCFKRGPP
QQIYAKLRELPFEAE-------K------SAA---CPVNASM-HSLLKMMNHCS-Q--HSLQKP-EMTEPRC
LYR----PTRK-TS-QC-KAVSQDSEKSISICDNLSELLMAMQDELDHMSMEHEE---LLKQMKKTKSRSVC
DDIECELEHLVKKMEIKGEQISKLRKHQDNVR--KLQQKVQNSKM-SDTSGIQREDSNLKGSKNIKNS--PR
KCLNETNPFQK--NNFQPVQ-VRNLQMKLRRDDIMWEQ
>XP_026245427.1/1-481 centrosomal protein CEP57L1 [Urocitellus parryii]

--MDSELIPSVI----GSYIKPP----
-E-------RV-FVSSFTKNESELPPKMNQNCH--PL-----NLEVTSPKMFHSPN-------SQ-------
-------ALILALKTLQEKIHRLELERTQAEDNLNILSREAAQYKKALENETNERNLAHQELIKQKKGISLQ
LRSAQSRCTLLEKQLEYTKRMVLNVEREKNMILEQQ------------------------------------
-----------AQLQREKEQDQLKLHAKLEKLDVLEKECFRLTTTQKTAEDKIKHLEEKLTEEEHQRKLFQD
KASELQTGLEISKILMSSVSKSKLSKE---KKKSSK-------------------------KTKSLKRGPT
QQIYSKLRELPFEAE-------K------SAA---SSANGSM-YSLLKMMHHCS-Q--CSLQKP-EVTEPRC
LYR----PTRK-TS-RY-KAVPQDSEKSISICDNLSELLMAMQDELDQMSMEHEE---LLKQMKETESHSVC
DDIECELERLVKKMEIKGEQISKLRKHQDNVY--KLQQKVQNSKMLSNTSVIQREDSNLKGSKNIKNS--PR
KCLNETNPFQK-NNNFHPVQ-VHNLQMKLRRDDIMWEQ
>XP_027784069.1/1-481 centrosomal protein CEP57L1 [Marmota flaviventris]

--MDSELIPSVI----GSYIKPP----
-E-------RV-FVSSFTKNESELPPKMNQNCH--PL-----NLEVTSPKMFHSPN-------SQ-------
-------ALILALKTLQEKIHRLELERTQAEDNLNILSREAAQYKKALENETNERNLAHQELIKQKKGISLQ
LRSAQSRCTLLEKQLEYTKRMVLNVEREKNMILEQQ------------------------------------
-----------AQLQREKEQDQLKLHAKLEKLDVLEKECFRLTTTQKTAEDKIKHLEEKLTEEEHQRKLFQD
KASELQTGLEISKILMSSVSKSKLSKE---KKKSSE-------------------------KTKSLKRGPT
QQIYSKLRELPFEAE-------K------SAA---SSANGSM-YSLLKMMHHCS-Q--CSLQKP-EVTEPRC
LYR----PTRK-TS-RY-KAVPQDSEKSISICDNLSELLMAMQDELDQMSMEHEE---LLKQMKETESHSVC
DDIECELEHLVKKMEIKGEQISKLRKHQDNVY--KLQQKVQNSKMLSNTSVIQREDSNLKGSKNIKNS--PR
KCLNETNPFQK-NNNFHPVQ-VHNLQMKLRRDDIMWEQ
>XP_013216163.1/1-483 centrosomal protein CEP57L1 isoform X1 [Ictidomys tridecemlineatus]XP_040139118.1 centrosomal protein CEP57L1 isoform X1 [Ictidomys tridecemlineatus]XP_040139119.1 centrosomal protein CEP57L1 isoform X1 [Ictidomys tridecemlineatus]KAG3290037.1 centrosomal protein 57 like 1, transcript variant X1 [Ictidomys tridecemlineatus]

---------------------------------------MDSELIPSVI----GSYIKPP----
-E-------RV-FVSSFTKNESELPPKMNQNCHQSEL-----NLEVTSPKMFHSPN-------SQ-------
-------ALILALRTLQEKIHRLELERTQAEDNLNILSREAAQYKKALENETNERNLAHQELIKQKKGISLQ
LRSAQSRCTLLEKQLEYTKRMVLNVEREKNMILEQQ------------------------------------
-----------AQLQREKEQDQLKLHAKLEKLDVLEKECFRLTTTQKTAEDKIKHLEEKLTEEEHQRKLFQD
KASELQTGLEISKILMSSVSKSKLSKE---KKKSSK-------------------------KTKSLKRGPT
QQIYSKLRELPFEAE-------K------SAA---SSANGSM-YSLLKMMHHCS-Q--CGLQKP-EVTEPRC
LYR----PTRK-TS-RY-KAVPQDSEKSISICDNLSELLMAMQDELDQMSMEHEE---LLKQMKETESHSVC
DDIECELERLVKKMEIKGEQISKLRKHQDNVY--KLQQKIQNSKMLSNTSVIQREDSNLKGSKNIKNS--PR
KCLNETNPFQK-NNNFHPVQ-VHNLQMKLRRDDIMWEQ
>XP_058431096.1/1-483 centrosomal protein CEP57L1 isoform X11 [Marmota monax]XP_058431098.1 centrosomal protein CEP57L1 isoform X11 [Marmota monax]

-----------------M--I-----
---------------------------------------MDSELIPSVI----GSYIKPP----
-E-------RV-FVSSFTKNESELPPKMNQNCH--PL-----NLEVTSPKMFHSPN-------SQ-------
-------ALILALKTLQEKIHHLELERTQAEDNLNILSREAAQYKKALENETNERNLAHQELIKQKKGISLQ
LRSAQSRCTLLEKQLEYTKRMVLNVEREKNMILEQQ------------------------------------
-----------AQLQREKEQDQLKLHAKLEKLDVLEKECFRLTTTQKTAEDKIKHLEEKLTEEEHQRKLFQD
KASELQTGLEISKILMSSVSKSKLSKE---KKKYSK-------------------------KTKSLKRGPT
QQIYSKLRELPFEAE-------K------SAA---SSANGSM-YSLLKMMHHCS-Q--CSLQKP-EVTKPRC
LYR----PTRK-TS-RY-KAVPQDSEKSISICDNLSELLMAMQDELDQMSMEHEE---LLKQMKETESHSVC
DDIECELEHLVKKMEIKGEQISKLRKHQDNVY--KLQQKVQNSKMLSNTSVIQREDSNLKGSKNIKNS--PR
KCLNETNPFRK-NNNFHPVQ-VHNLQMKLRRDDIMWEQ
>VTJ71873.1/1-491 Hypothetical predicted protein [Marmota monax]
--M-------------
--MLYLY---------------

---------------SLQ--I-----
---------------------------------------MDSELIPSVI----GSYIKPP----
-E-------RV-FVSSFTKNESELPPKMNQNCH--PL-----NLEVTSPKMFHSPN-------SQ-------
-------ALILALKTLQEKIHHLELERTQAEDNLNILSREAAQYKKALENETNERNLAHQELIKQKKGISLQ
LRSAQSRCTLLEKQLEYTKRMVLNVEREKNMILEQQ------------------------------------
-----------AQLQREKEQDQLKLHAKLEKLDVLEKECFRLTTTQKTAEDKIKHLEEKLTEEEHQRKLFQD
KASELQTGLEISKILMSSVSKSKLSKE---KKKYSK-------------------------KTKSLKRGPT
QQIYSKLRELPFEAE-------K------SAA---SSANGSM-YSLLKMMHHCS-Q--CSLQKP-EVTKPRC
LYR----PTRK-TS-RY-KAVPQDSEKSISICDNLSELLMAMQDELDQMSMEHEE---LLKQMKETESHSVC
DDIECELEHLVKKMEIKGEQISKLRKHQDNVY--KLQQKVQNSKMLSNTSVIQREDSNLKGSKNIKNS--PR
KCLNETNPFRK-NNNFHPVQ-VHNLQMKLRRDDIMWEQ
>XP_045429148.1/1-451 centrosomal protein CEP57L1 isoform X2 [Pipistrellus kuhlii]

---------------------------------------MDSELMHSIV----GSYRKPP----
-E-------RM-FAPSFTQNDEL-----SRNHL--SV-----NFEVTSPKKLHSPN-------SQ-------
-------ALILALKTLQEKIHRLELERTQAEDNLNILSREAAQYKKALEDETNERNLVHQELIKQKKDISIQ
LSSAQARCTLLEKQLEYTKRMVLNVEREKNMILEQQ------------------------------------
-----------TQLQREKEQDQMKLQAKLEKLDLLEKECFKLTTTQKTAEDKIKHLEEKLKEEEHQRKLFQD
KASQLQTGLEINRILMSSVSNPNLSKE---KKKSSK-------------------------PA--------
--------------------------------RAC-HSVNASV-QNLLQMVQHYG-P--QILQKPAEAAEPRC
LYK----PART-AS-QC-KAVPPDSRTSVSICDNLSELLMEMQEELDQMGVEHQE---LLNQMKETESHTVC
EDIECELEHLVKKMEIKREQISKLMKHQDSVR--KLQQKVQNSKM-REASYIQREDSNPKGLKNIKYS--PR
KCLVNHS-LQK-NSNFHPTQ-VHNLQMKLRRDDIMWEQ

>KAK1338132.1/1-449 hypothetical protein QTO34_001242, partial [Eptesicus nilssonii]

---I-----
---------------------------------------MDSELMHSIV----GSYIKPP----
-E-------RM-FAPSFTQNDEL-----SRNHR--SV-----NFEVTSPKMLHSPN-------SQ-------
-------ALILALKTLQEKIHRLELERTQAEDNLNILSREAAQYKKALEDETNERNLVHQELIKQKKDISIQ
LSSAQARCTLLEKQLEYTKRMVLNVEREKNMILEQQ-----------------------------------
-----------TQLQREKEQDQMKLQAKLEKLDLLEKECFKLTTTQKTAEDKIKHLEEKLKEEEHQRKLFQD
KASE---KTKCLKRGPP
QQMYSKFGSMPMVAE-------K------PARAC-HSVNASV-QNLLQMVQHYG-P--QILQKPAEVAEPRC
LYK----PTRT-TS-QC-KVVPPDSEKSVSICDNLSELLMAMQEELDQMGVEHQE---LLNQMKETESHAVC
EDIECELEHLVKKMEIKREQISKLMKHQDSVR--KLQQKVQNSKM-SEASCIQREDSNPKGLKNIKYS--PR
KCLLNHS-LQK-NSNFHPTQ-VHNLQMKLRRDDIMWEQ
>XP_062042574.1/1-490 centrosomal protein CEP57L1 isoform X1 [Lepus europaeus]

---M------------------
---WD-------------
-------T-STSG---QGFNLI-----
---------------------------------------MDSELMHSIV----GSYLRPP----
-E-------RG-FIPSFTQNESL------QNHY--PV-----NEEVTSSKVLQSPN-------SQ-------
-------ALILALRTLQEKIHRLELERTQAEDNLNILSREAAQYKKALQNETNERNLAHQELIKQKKDISIQ
LSSAQSRCTLLEKQLEYTKRMVLNVEREKNMILEQQ-----------------------------------
-----------ALLQREKEQDQMKLHAKLEKLDVLEKECLRLTATQKTAEDKIKQLEEKLTEEEHHRKLFQD
KASELQTGLEISKILMSSVSNSKISKE---KKK-SK------------------------TNKCLKRGPP
QPVYSKFEALPFETE-------R------SASAG-SSVNASA-QNLLQTARRSG-P--RILQKPSEVTEHRC
LCR----PTRK-AS-WC-RAVPSDSEKSDSFCDNLSELLMAMQDELDQMGVEHQE---LLKQMKDTASHSVY
GDIECELEHLVKKMEIKREQISKLKKHQDSVR--KLQQKVQNPKM-RTAPGIQREDSQPNGSKNRKNS--PR
KCLNETNSFQK-NNSFHPVR-VHNLKVKLRKDDIMWEQ
>XP_010598689.1/1-444 centrosomal protein CEP57L1 isoform X3 [Loxodonta africana]XP_023398774.1 centrosomal protein CEP57L1 isoform X3 [Loxodonta africana]

---------------------------------------MQS---------------------
-----------------------------PT-----GIR----ECLSSSNLEE---WRH-------
-------ALILALKTLQDKIHRLELERTQAEDNLNILSKEAAQYKKALENETNERNLAHQELIKQKKDISIQ
LSSAQSRCTLLEKQLEYTKRMVLNAEREKNIILEQQ-----------------------------------
-----------AQLQREKEQDHMKLHAKLEKLDVLEKECFRLTTTQKTAEDKIKHLEEKLKEEEHQRKLFQD
KASELQTGLEINRILMSSVSNSKHSKG---KKKSSK------------------------KTKCLKRGLP
QESYSKFGAPSFVPG-------K------FVRAS-HSVNASV-QNLLQMVQDCG-L--QTLQKHSKASEPRY
FHK----PSR--IL-QC-KAAPPHPENSVSVCDNLSELLMAMQDELDQMNMEHQE---LLNQMKETQSHSVC
EDIESELEHLVKKMEIKGEQISKLKKHQDSVH--KLQKKLQNSRM-SEASGIQREDSNSKGSKNVKNS--PR
KCSHESSPLQK-NSNFHPKR-VHNLQMKLSKDDIMWEQ
>XP_042636872.1/1-476 centrosomal protein CEP57L1 [Orycteropus afer afer]

---------------------------------------MDSELIDSIV----GSYLKPP----
-E-------RG-FVPSFSQHETS------QNCH--PV-----NVEVTSPKMFHSPN-------SQ-------
-------ALILALKTLQDKIHRLELERTQAEDNLNILSKEAAQYKKALENETNERNLAHQELIKQKKDISIQ
LTSAQSRCTLLEKQLEYTKRMVLNAEREKNIILEQQ-----------------------------------
-----------AQLQREKEQDHMKLHAKLEKLDILEKECFRLTTTQKTAEDKIKHLEEKLKEEEHQRKLFQD
KASELQTGLEINRILMSSVSNLKHSKG---KKKSSK------------------------KTKCLKRGLP
QQSYSTFGAPPFVPG-------K------SVSAR-HSVSASM-QNLLQMTQSCG-P--HTLEKHAKVTEPRC
FPK----PSR--TP-QC-RPAPPNSENSISICDNLSELLMAMQDELDQMSMEHQE---LLNQMKQTQSHSAC

EGIESELEHLVKKMEIKGEQISKLKKHQDSVH--KLQQKLQNSKM-SKASSIQREDSTLKGSKNIKNS--PR
KCSHETSPIQK-NSTFPPKR-VHNLQMKLRKDDIMWEQ
>XP_023583400.1/1-479 centrosomal protein CEP57L1 isoform X1 [Trichechus manatus latirostris]XP_023583401.1 centrosomal protein CEP57L1 isoform X1 [Trichechus manatus latirostris]

---MV-----
--MDSELMHSIV----GSYLKPP----
-E-------RV-FVPSFTPYETS------QNCH--PV-----NMEVTPPKMFYSPN-------NQ-------
------AALILALKTLQDKIHRLELERTQAEDNLNILSKEAAQYKKALENETNERNLAHQELIKQKKDISIQ
LSSAQSRCALLEKQLEYTKRMVLNAEREKNIILEQQ------------------------------------
-----------AQLQREKEQDHLKLHAKLDKLDVLEKECFRLTTTQKTAEDKIKHLEEKLKEEEHQRKLFQD
KASELQTGLEISRILMSSVSNPKHSKG---KKKSSK-------------------------KTKCLQRGLP
QESYLKFGAPPFVPG-------K------LVSTS-HSGNASV-QNLLQMMRDCG-P--HTLQKHSKAPEPRC
LHK----PST--TP-QC-KAVPPDPEKSVSICDNLSELLMAMQDELDQMNMEHQE---LLNQMKETQSHSVC
EDIESELEHLVKKMGIKAEQISKLKKHQNSVR--ELQQKLQNSKM-SEASGIQQEDSNPKGSKNMKNS--PR
KCSHETSPLQK-NSNFHPKR-VRNLQMKLSKDDIMWEQ
>XP_023398740.1/1-478 centrosomal protein CEP57L1 isoform X1 [Loxodonta africana]XP_023398747.1 centrosomal protein CEP57L1 isoform X1 [Loxodonta africana]XP_023398752.1 centrosomal protein CEP57L1 isoform X1 [Loxodonta africana]XP_023398757.1 centrosomal protein CEP57L1 isoform X1 [Loxodonta africana]XP_049752801.1 centrosomal protein CEP57L1 isoform X1 [Elephas maximus indicus]XP_049752896.1 centrosomal protein CEP57L1 isoform X1 [Elephas maximus indicus]XP_049752982.1 centrosomal protein CEP57L1 isoform X1 [Elephas maximus indicus]XP_049753067.1 centrosomal protein CEP57L1 isoform X1 [Elephas maximus indicus]XP_049753167.1 centrosomal protein CEP57L1 isoform X1 [Elephas maximus indicus]XP_049753251.1 centrosomal protein CEP57L1 isoform X1 [Elephas maximus indicus]XP_049753295.1 centrosomal protein CEP57L1 isoform X1 [Elephas maximus indicus]XP_049753386.1 centrosomal protein CEP57L1 isoform X1 [Elephas maximus indicus]XP_049753444.1 centrosomal protein CEP57L1 isoform X1 [Elephas maximus indicus]XP_049753487.1 centrosomal protein CEP57L1 isoform X1 [Elephas maximus indicus]

---MV-----
--MDSELTHSIV----GSYLKPP----
-E-------RV-FVPSFTQYETS------QNCH--PV-----KVEVTPPEVFHSPN-------SQ-------
-------ALILALKTLQDKIHRLELERTQAEDNLNILSKEAAQYKKALENETNERNLAHQELIKQKKDISIQ
LSSAQSRCTLLEKQLEYTKRMVLNAEREKNIILEQQ------------------------------------
-----------AQLQREKEQDHMKLHAKLEKLDVLEKECFRLTTTQKTAEDKIKHLEEKLKEEEHQRKLFQD
KASELQTGLEINRILMSSVSNSKHSKG---KKKSSK-------------------------KTKCLKRGLP
QESYSKFGAPSFVPG-------K------FVRAS-HSVNASV-QNLLQMVQDCG-L--QTLQKHSKASEPRY
FHK----PSR--IL-QC-KAAPPHPENSVSVCDNLSELLMAMQDELDQMNMEHQE---LLNQMKETQSHSVC
EDIESELEHLVKKMEIKGEQISKLKKHQDSVH--KLQKKLQNSRM-SEASGIQREDSNSKGSKNVKNS--PR
KCSHESSPLQK-NSNFHPKR-VHNLQMKLSKDDIMWEQ
>XP_006840005.1/1-459 PREDICTED: centrosomal protein CEP57L1 [Chrysochloris asiatica]

--MDSELMHSIV----GSYLKPP----
-E-------RV-FVPSFSRYETS------QNYH--PV-----NVEVIPPKMFHSPN-------SQ-------
-------ALILALKTLQDKIHRLELERTQAEDNLNILSKEAAQYKKALENETNERNLAHEELIKQKKDISIQ
LSSAQSRCTLLEKQLEYTKRMVLNAEREKNIILEQQ------------------------------------
-----------AQLQREKEQDQMKLHAKLAKLDVLEKECFRLTATQKSAEDKIRYLEEKLKEEEHQRKLFQD
KASELQTGLEINRILVSSVSNPKHCKG---KKKSSK-------------------------KTKCLKRGLP
QQSYSRFGAPPFVSG-------K-------------------------RMQDCG-P--HTLQKHSKAVEPVC

LHE----PSR--VP-QC-RAVSSDSEKSIAICDNLSELLMAMQAELHQMNMEHQE---LLNQMKETHSYLVY
EDIEGELEHLVKKMEVKREQISKLKKHQDSVH--KLQQQLQNSKM-NEASGIQREDSNHKGSKNIKNI--PR
KCSHETSSLQK-NRNFHPKR-VHNLQMKLRKDDIMWEQ
>XP_006157026.1/1-483 centrosomal protein CEP57L1 isoform X1 [Tupaia chinensis]

------------------MLSS--FRV-----
---MDSELMHSIV----GSYIKPP----
-E-------RM-FVPSFTQKESS------QNQQ--PV-----DIEVTSPKMLHSPN-------SQ-------
-------ALILALKTLQEKIHRLELERTQAEDNLNILSREAAEYKKALENETNERNLAHEELIKQKKDISIQ
LSSAQSRCTLLEKQLEYTKRMVLNVEREKNMILEQQ------------------------------------
-----------AQLQREKEQDQMKLHAKLEKLDVLEKECFKLTTTQKTAEDKIKYLEEKLKEEEHQRKLFQD
KASELQTGLEISKILMSSASNRKHSKE---KKKPLK-------------------------KTKCLKRGPS
QQIYSKFGALPFVAE-------K------SASVG-CSVNASM-QNLLQMMEQCS-P--HVLQKP-EAAEPRC
LYK----PMQT-TS-QC-KAVSPAPELSTSICDNLSELLIAMQDEMDQMSMEHQE---LLKQMKETESHSVC
DDIECELERLVKKMEIKAEQISKLRKHQDSVR--KLQQKVQNSKM-SETSGIQREDNNPKGSKNIKNI--PR
KCLNETNPLQK-NSNFHPIR-VHNLQVKLRRDDIMWEQ
>XP_006157035.1/1-457 centrosomal protein CEP57L1 isoform X5 [Tupaia chinensis]

------------------MLSS--FRV-----
---MDSELMHSIV----GSYIKPP----
-E-------RM-FVPSFTQKESS------QNQQ--PV-----DIEVTSPKMLHSPN-------SQ-------
-------ALILALKTLQEKIHRLELERTQAEDNLNILSREAAEYKKALENETNERNLAHEELIKQKKDISIQ
LSSAQSRCTLLEKQLEYTKRMVLNVEREKNMILEQQ------------------------------------
-----------AQLQREKEQDQMKLHAKLEKLDVLEKECFKLTTTQKTAEDKIKYLEEKLKEEEHQRKLFQD
KASELQTGLEISKILMSSASNRKHSKE---KKKPLK-------------------------S---------
------------------------------ASVG-CSVNASM-QNLLQMMEQCS-P--HVLQKP-EAAEPRC
LYK----PMQT-TS-QC-KAVSPAPELSTSICDNLSELLIAMQDEMDQMSMEHQE---LLKQMKETESHSVC
DDIECELERLVKKMEIKAEQISKLRKHQDSVR--KLQQKVQNSKM-SETSGIQREDNNPKGSKNIKNI--PR
KCLNETNPLQK-NSNFHPIR-VHNLQVKLRRDDIMWEQ
>XP_006157032.1/1-457 centrosomal protein CEP57L1 isoform X4 [Tupaia chinensis]XP_006157033.1 centrosomal protein CEP57L1 isoform X4 [Tupaia chinensis]

------------------MTTT--IRY-----
--SELC------------------
----------L-LFPT---------------KE--GF-----DF---------APNFATYVIFLQ-------
-------ALILALKTLQEKIHRLELERTQAEDNLNILSREAAEYKKALENETNERNLAHEELIKQKKDISIQ
LSSAQSRCTLLEKQLEYTKRMVLNVEREKNMILEQQ------------------------------------
-----------AQLQREKEQDQMKLHAKLEKLDVLEKECFKLTTTQKTAEDKIKYLEEKLKEEEHQRKLFQD
KASELQTGLEISKILMSSASNRKHSKE---KKKPLK-------------------------KTKCLKRGPS
QQIYSKFGALPFVAE-------K------SASVG-CSVNASM-QNLLQMMEQCS-P--HVLQKP-EAAEPRC
LYK----PMQT-TS-QC-KAVSPAPELSTSICDNLSELLIAMQDEMDQMSMEHQE---LLKQMKETESHSVC
DDIECELERLVKKMEIKAEQISKLRKHQDSVR--KLQQKVQNSKM-SETSGIQREDNNPKGSKNIKNI--PR
KCLNETNPLQK-NSNFHPIR-VHNLQVKLRRDDIMWEQ
>EHB04194.1/1-459 Cep57-related protein [Heterocephalus glaber]

---MDSELMHSIV----GSYFKPP----
-E-------RV-FVPPFIQDETS------QKCH--PV-----NLEVTAPKMLHSPN-------SQ-------
-------ALTWALQTLQEKIHRLELERTQAEDNLNILSREAAQYKKALENETNERNLAHQELIKQKKDISIQ
LSSAQSRCTLLEKQLEYTKRMVLNVEREKNMILEQQ------------------------------------
-----------AQLQREKEQDQMKLHAKLEKLDVLEKECFRLTTTQKTAEDKIKHLEEKLQEEEHQRKLFQD
KAAELQTGLEISKILMSSVSNSKHSKE---KKKSSK-------------------------KTKCLKREPP

PQIYSKFRAPLF-------------------------------EAKKHKMYHCS-P--HVFPKHAHVIEPRC
LCK----PPRT-VS-QY-NAVPCESEKSVSICDNLSELLMAMQDELDQMSMEHEE---LLKQMKETESHSMC
DDIECELECLVKKMEIKEKQISKLKKHQDSVH--KLQQKVQNSKK-SKALGIQQDSN--QGLKSMKNS--PR
KCLNEANSSQK-SSNFHPIR-VHNIQAKLRRDDIMWEQ
>XP_021107187.1/1-475 centrosomal protein CEP57L1 isoform X4 [Heterocephalus glaber]

---MDSELMHSIV----GSYFKPP----
-E-------RV-FVPPFIQDETS------QKCH--PV-----NLEVTAPKMLHSPN-------SQ-------
-------ALTWALQTLQEKIHRLELERTQAEDNLNILSREAAQYKKALENETNERNLAHQELIKQKKDISIQ
LSSAQSRCTLLEKQLEYTKRMVLNVEREKNMILEQQ------------------------------------
-----------AQLQREKEQDQMKLHAKLEKLDVLEKECFRLTTTQKTAEDKIKHLEEKLQEEEHQRKLFQD
KAAELQTGLEISKILMSSVSNSKHSKE---KKKSSK--------------------------KTKCLRKEPP
PQIYSKFRAPLFEAK-------K------SASAG-CSVNAGR-QSLQHKMYHCS-P--HVFPKHAHVIEPRC
LCK----PPRT-VS-QY-NAVPCESEKSVSICDNLSELLMAMQDELDQMSMEHEE---LLKQMKETESHSMC
DDIECELECLVKKMEIKEKQISKLKKHQDSVH--KLQQKVQNSKK-SKALGIQQDSN--QGLKSMKNS--PR
KCLNEANSSQK-SSNFHPIR-VHNIQAKLRRDDIMWEQ
>XP_005377911.1/1-475 PREDICTED: centrosomal protein CEP57L1 isoform X4 [Chinchilla lanigera]XP_005377912.1 PREDICTED: centrosomal protein CEP57L1 isoform X4 [Chinchilla lanigera]

---MDSELMHSIV----GSYLKPP----
-E-------RL-FVPPFMQDESS------QNCH--PM-----NLKVAPAKVLHSPN-------SQ-------
-------ALILALKTLQEKIHRLELERTQAEDNLNILSREAAQYKKALENETNERNLAHQELIKQKKDISIQ
LSSAQSRCTLLEKQLEYTKRMVLNVEREKNMILEQQ------------------------------------
-----------AQLQREKEQDQMRLHAKLEKLDVLEKECFRLTTTQKTAEDKIKHLEEKLKEEEHQRKLFQD
KAAELQTGLEISKILMSSVSNSKRSKE---KKKSSK--------------------------KTKCLKRGPP
LQIYSKFRAPLFEAK-------K------SASAG-CSVNAGR-QNLQQTTHHCG-P--QILPKRAQVIEPRC
LCK----PPRA-IS-QY-KAVPRESEKSISICDSLSELLMAMQDELDQMSMEHAE---LLKQMKETETHSMC
DDVECELERLVKKMEIKGKQISRLKKHQDSVY--KLQQKVQNSKA-SKASGIQQDGN--QELKNMKNS--PR
KCLNEANSSQK-SSNFHPMQ-VHNIRMKLRRDDIMWEQ
>XP_023578060.1/1-477 centrosomal protein CEP57L1 isoform X1 [Octodon degus]XP_023578061.1 centrosomal protein CEP57L1 isoform X1 [Octodon degus]XP_023578062.1 centrosomal protein CEP57L1 isoform X1 [Octodon degus]XP_023578063.1 centrosomal protein CEP57L1 isoform X1 [Octodon degus]

--M-----
---MDSELMHNIV----GSYLKPP----
-E-------RM-FVPPLIEDESS------QNCH--PV-----NLQVTAAKMIHSPN-------SQ-------
-------ALILALKTLQEKIHRLEIERTQAEDNLNMLSREAAQYKKALENETNERNLAHQELVKQKKDISIQ
LSSAQSRCTLLEKQLEYTKRMVLNVEREKNMILEQQ------------------------------------
-----------AQLQREKEQDQMKLHAKLEKLDVLEKECFRLTTTQKTAEDKIKHLEEKLKEEEHQRKLFQD
KAAELQTGLEISKILMSSVSNSKHYKE---KKKSSK--------------------------KTKCLKRGPP
LQIYSELRAPLIEAK-------K------SASAG-CSVNASW-QDLQQKMPRCG-P--RNLPKCAQVVEPRC
LCK----PPRA-VS-QYCKAVPRGSEKSISICDSLSELLMAMQEELDQMSMEHEE---LLKQMKETETHSVC
DDIEYELERLVKKMEIKGKQISRLKKHQDSVY--KLQQKVHNSKA-SKASGIQQDGN--QELKNMKNS--PR
KCLHEANSSQK-SNNFHPIQ-VHNIQMKLRRDDIMWEQ
>XP_003466085.2/1-475 centrosomal protein CEP57L1 isoform X1 [Cavia porcellus]XP_013003970.1 centrosomal protein CEP57L1 isoform X1 [Cavia porcellus]

---MDSELMHSIV----GSYLKPP----
-E-------RV-FVPPFIRDESS------QHCH--PV-----NLEVTAAKMLHSPN-------SQ-------
-------ALILALKTLQEKIHRLELERTQAEDNLNTLSREAAQYKKALEDETNERNVAHRELIKQKKDISIQ
LSSAQSRCTLLEKQLEYTKRMVLNVEREKSMILEQQ------------------------------------
-----------AQLQKEKEQDQMKLHAKLEKLDILEKECFRLTTTQKTAEDKIKHLEEKLKEEEHQRKLFQD
KAAELQTGLEISKILMSSVSNSKHYKE---KKKSSK--------------------------KTTCLKRGPP
LQIYSNFRAPPFEAT-------K------SASMA-CSVNADR-QHLQQKTHRCG-S--HILPKRAQEIEPRC
LCK----PPRT-VS-QY-KAVPCESENSISICDSLSELLMAMQDELDQMSMEHEE---LLKQMKETETHSVC
DDIECELERLVEKMEIKGKQISKLKKHQDKVY--KLQQKVQNLKA-YKASVIQQAAN--QDLKIMKNS--PR
KCLTKANCSQK-SSNFHPVQ-VHNIQMKLRRDDIMWEQ
>XP_019064749.1/1-544 centrosomal protein CEP57L1 isoform X1 [Fukomys damarensis]
---MHVKNKGNNSGSHRQNR
GH------------------LLDS-----------------------ISRCARSLFNS--------------
--SK-------ETVFESS---SE-
------GR-KDHGSKGRSRETGYEAK-----------------------------------VI-IQI-----
---MDSELMHSIV----GSYLKPP----
-E-------TM-FVPPFIQDESS------QKCH--AV-----NLEVTAPKMRHSPN-------SQ-------
-------ALILALKTLQDKIHRLELERTQAEDNLNILSREAAQYKKALENETNERNLAHQELIKQKKDISIQ
LSSAQSRCTLLEKQLEYTKRMVLNVEREKNMILEQQ------------------------------------
-----------AQLQREKEQDQMKLHAKLEKLDVLEKECFRLTTTQKTAEDKIKHLEEKLQEEEHQRKLFQD
KAAELQTGLEISKILMSSVSNSKHSKE---KKKSSK--------------------------KSKCLKKGPP
LQIYSKFRAPLFEAK-------K------SASAG-CSVNAGR-QSLQHKTYHCS-P--HGFPKGTQVIEPRC
LCK----PPRT-IS-QY-KAVPRESEKSISICDNLSELLMAMQDELDQMSMEHEE---LLKQMKETESHSIC
DNIEYELERLVKKMEIKEKQISKLKTHQDNVH--KLQQKVQNSKT-SKASGIQQDSN--QSLKSMKNS--PR
KCLNKANSSQK-SSNFHPIQ-VHNIQVKLRRDDITWEQ
>XP_037677172.1/1-434 centrosomal protein CEP57L1-like [Choloepus didactylus]

---MDFELMNSVV----GSYLKPP----
-E-------RM-FVPSVTQYESS------QNCH--PV-----NLEVTSPKMPHSPN-------SQ-------
-------AHFLALKTLQDKIHRLELERTQSEDNLNILSREASQYKKALENETNERNLAHQELIKQKKDISIQ
LNSAQSRCILLEKQLEYTNRMVLNAEREKNMVLEQQ------------------------------------
-----------AQLQREKEQDHMMLHAKLEKLDVLEKECFRLTTTQKTAEDKIKHLEEKLKEEEHQRKLFQD
KASELQTGLEINRILMSSISKPKYSKE---MKKSSK------------------------------------
--IMQHCD-Q--RALPKRSEVTEPRC
RCR----PSRR-PS-QC-KAVTPDSEMSISICDNLSEVLMAMQDELDQMNMEHQE---ILNQMKETESHSVC
EDIECELEHLAKKMEIKEEQISKLKKHQDSVC--KLQHKVKNLKM-NEASGIRQEDSNPKRSKNIKNS--PR
KCLHDTNPPQK-NSSLPPKQ-VHNLLMKLRKDYILWEQ
>XP_037698225.1/1-453 centrosomal protein CEP57L1 isoform X4 [Choloepus didactylus]

---MI-----
---MDFELMNSVV----GSYLKPP----
-E-------RM-FVPSVTQYESS------QNCH--PV-----NLEVTSPKMPHSPN-------SQ-------
-------ALFLALKTLQDKIHRLELERTQAEDNLNILSREAAQYKKALENETNERNLAHQELIKQKKDISIQ
LNSAQSRCILLEKQLEYTKRMVLNAEREKNMVLEQQ------------------------------------
-----------AQLQREKEQDHMMLHAKLEKLDILEKECFRLTTTQKTAEDKIKHLEEKLKEEEHQRKLFQD
KASELQTGLEINRILMSSISKPKYSKE---MKKSSK--------------------------L---------
------------------------------ASAS-HSVNANM-QNFLQIMQHCD-Q--RALPKRSEVTEPRC
RCR----PSRR-PS-QC-KAVTPDSEMSISICDNLSELLMAMQDELDQMNMEHQE---ILNQMKETESHSVC
EDIECELEHLVKKMEIKEEQISKLKKHQDSVC--KLQHKVKNLKM-NEASGIRQEDSNPKRSKNIKNS--PR
KCLHDTNPPQK-NSSLPPKQ-VHNLLMKLRKDDILWEQ
>XP_037698217.1/1-479 centrosomal protein CEP57L1 isoform X1 [Choloepus didactylus]XP_037698218.1 centrosomal protein CEP57L1 isoform X1 [Choloepus didactylus]XP_037698219.1 centrosomal protein CEP57L1 isoform X1 [Choloepus

didactylus]XP_037698220.1 centrosomal protein CEP57L1 isoform X1 [Choloepus didactylus]XP_037698221.1 centrosomal protein CEP57L1 isoform X1 [Choloepus didactylus]

---MI-----
---MDFELMNSVV----GSYLKPP----
-E-------RM-FVPSVTQYESS------QNCH--PV-----NLEVTSPKMPHSPN-------SQ-------
-------ALFLALKTLQDKIHRLELERTQAEDNLNILSREAAQYKKALENETNERNLAHQELIKQKKDISIQ
LNSAQSRCILLEKQLEYTKRMVLNAEREKNMVLEQQ------------------------------------
-----------AQLQREKEQDHMMLHAKLEKLDILEKECFRLTTTQKTAEDKIKHLEEKLKEEEHQRKLFQD
KASELQTGLEINRILMSSISKPKYSKE---MKKSSK-------------------------KTKCLKRGPP
QQIYSKFGALPFVAG-------K------LASAS-HSVNANM-QNFLQIMQHCD-Q--RALPKRSEVTEPRC
RCR----PSRR-PS-QC-KAVTPDSEMSISICDNLSELLMAMQDELDQMNMEHQE---ILNQMKETESHSVC
EDIECELEHLVKKMEIKEEQISKLKKHQDSVC--KLQHKVKNLKM-NEASGIRQEDSNPKRSKNIKNS--PR
KCLHDTNPPQK-NSSLPPKQ-VHNLLMKLRKDDILWEQ
>XP_004468459.2/1-480 centrosomal protein CEP57L1 isoform X1 [Dasypus novemcinctus]

---MV-----
---MDFELMNSIV----GSYHKPP----
-E-------RM-FVSSVTQYESS------QNCH--PV-----NSEVTSPKMPHSPN-------SQ-------
-------ALFLALKTLQDKIHRLELERTQAEDNLNILSREAAQYKKALENETNERNLAHQELIKQKKDISIQ
LSSAQSRCTLLEKQLEYTKRMVLNAEREKNMVLEQQ------------------------------------
-----------AQLQREKEQDHMMLHAKLEKLDVLEKECFRLTTTQKTAEDKIKHLEEKLKEEEHQRKLFQD
KASELQTGIEINRILMSSVSKPKYSKE---MKKSSK-------------------------KTKCLKRGLP
LQIYSKFGVLPFVAG-------K------SASAS-HSVNANM-QNFLQMTQHCD-Q--RILQKNSEVTEPRC
LFR----PSRRGPS-QC-KAVPPDSEKSISVCDNLSELLMAMQDELEQMSMEHQE---ILNQMKETESRSIC
EDIECELERLVKKMEIKEEQISKLKKHQDSVR--KLQDKVQNLRM-NEASDIQREDSSPKRAKNIKNS--PR
KCLNQTNPPQK-NSNLHPKQ-VHNLVMKLRKDDIMWEQ
>XP_045010403.1/1-478 centrosomal protein CEP57L1 isoform X1 [Jaculus jaculus]XP_045010404.1 centrosomal protein CEP57L1 isoform X1 [Jaculus jaculus]XP_045010405.1 centrosomal protein CEP57L1 isoform X1 [Jaculus jaculus]

--M-----
---MDSEFIHSIV----GSYLKPP----
-E-------RV-FVPSLTQNESP------QYHH--PV-----NFQATAPKMVNSPN-------SQ-------
-------ALVLALRTLQEKIHRLELERTQAEDNLSILSREAAQYKKALENETNERNLAHQELIKQKKDISMQ
LSSAQSRCTILEKQLEYTKRMVLNVEREKNMILEQQ------------------------------------
-----------AQLQREKEQDQMKLHAKLEKLDVLEKECFRLTTTQQTAEEKIKYLEEKLKEEEHQRKLFQD
KASELQTGLEISKILMSSVSNSKHSKE---KKKSSK-------------------------KSKCIKGRPP
QQIESKLSTPSFKCE-------K------LNSSD-CSVSASL-QNLLQLMPRCG-P--QSFPKPALALEPRC
LHK----PPRT-AS-GC-RTASCSSEKPIVIFDNLSELLMAMQDELNQMSLEHNK---LLTQREQTESRAVC
DDIEWELEQLVKRMEIKGEQISKLKKHQNSVR--KLQQKFQNSWK-SEPSSTLQDDTNPKGSKCMKNS--PR
KYLSETNPFPK-RSNFHPIQ-VHNLQMKLRRDDIMWEQ
>XP_008518740.1/1-453 PREDICTED: centrosomal protein CEP57L1 isoform X2 [Equus przewalskii]XP_008518748.1 PREDICTED: centrosomal protein CEP57L1 isoform X2 [Equus przewalskii]XP_008518756.1 PREDICTED: centrosomal protein CEP57L1 isoform X2 [Equus przewalskii]XP_008518760.1 PREDICTED: centrosomal protein CEP57L1 isoform X3 [Equus przewalskii]XP_014589949.2 centrosomal protein CEP57L1 isoform X2 [Equus caballus]XP_014589955.2 centrosomal protein CEP57L1 isoform X3 [Equus caballus]XP_023506657.1 centrosomal protein CEP57L1 isoform X2 [Equus caballus]


```
-----------------------------------------------------------------MI-----
-----------------------------------------------MDPELMHSIV----GSYLKPP----
-E-------RV-LVPSFTQNDQS-----APTHH--SV-----SLEVNLPKMLHSPN-------SQ-------
-------ALILALKTLQEKIHRLDLERTQAEDNLNVLSREAAQYKKALENETNERNLAHQELIKQKKDITIQ
LSSAQSRCTLLEKQLEYTKRMVLNVEREKNMILEQQ------------------------------------
-----------AQLQREKEQDHLKLQAKLEKLDVLEKECFKLTTTQKTAEDKIKHLEEKLKEEEHHRKLFQD
KASELQTGLEINRILMSSVSNPKRSKE---KKKSS-------------------------------------
----------------------K------SASAS-HSVKASR-QNLLQTMQHYG-P--HILPKRAEVTEPRR
LYK----PTRS-TS-QR-KAVPPDSGKSICICNNVSELLMAMQDELDQMSMKHQE---LLNQIKETESHSVF
ENAECELEHLVKKMEIKVEQISKLKKHQDNVR--KLQQKVQNSKM-SEASGIRQEDNYPNGSNNLKNS--PR
KCLLNNP-LQK-SSNFHPIR-VRNLQMKLRRDDIMWEQ
>XP_014694517.2/1-453 centrosomal protein CEP57L1 isoform X1 [Equus asinus]
------------------------------------------------------------------------
------------------------------------------------------------------------
------------------------------------------------------------------------
-----------------------------------------------------------------MI-----
-----------------------------------------------MDPELMHSIV----GSYLKTP----
-E-------RV-LVPSFTQNDQS-----APTHH--SV-----SLEVNLPKMLHSPN-------SQ-------
-------ALILALKTLQEKIHRLDLERTQAEDNLNVLSREAAQYKKALENETNERNLAHQELIKQKKDITIQ
LSSAQSRCTLLEKQLEYTKRMVLNVEREKNMILEQQ------------------------------------
-----------AQLQREKEQDHLKLQAKLEKLDVLEKECFKLTTTQKTAEDKIKYLEEKLKEEEHHRKLFQD
KASELQTGLEINRILMSSVSNPKRSKE---KKKSS-------------------------------------
----------------------K------SASAS-HSVKASR-QNLLQTMQHYG-P--HILPKRAEVTEPRR
LYK----PTRS-TS-QR-KAVPPDSGKSICICNNVSELLMAMQDELDQMSMKHQE---LLNQIKETESHSVF
ENAECELEHLVKKMEIKVEQISKLKKHQDNVR--KLQQKVQNSKM-SEASGIQQEDNYPNGSNNLKNS--PR
KCLLNNP-LQK-SSNFHPIR-VRNLQMKLRRDDIMWEQ
>XP_008518731.1/1-461 PREDICTED: centrosomal protein CEP57L1 isoform X1 [Equus
przewalskii]XP_023506656.1 centrosomal protein CEP57L1 isoform X1 [Equus
caballus]
------------------------------------------------------------------------
---------------------------------------------------MYFE-----------------
------------------------------------------------------------------------
------------GA-------------------------------------------------QRQI-----
-----------------------------------------------MDPELMHSIV----GSYLKPP----
-E-------RV-LVPSFTQNDQS-----APTHH--SV-----SLEVNLPKMLHSPN-------SQ-------
-------ALILALKTLQEKIHRLDLERTQAEDNLNVLSREAAQYKKALENETNERNLAHQELIKQKKDITIQ
LSSAQSRCTLLEKQLEYTKRMVLNVEREKNMILEQQ------------------------------------
-----------AQLQREKEQDHLKLQAKLEKLDVLEKECFKLTTTQKTAEDKIKHLEEKLKEEEHHRKLFQD
KASELQTGLEINRILMSSVSNPKRSKE---KKKSS-------------------------------------
----------------------K------SASAS-HSVKASR-QNLLQTMQHYG-P--HILPKRAEVTEPRR
LYK----PTRS-TS-QR-KAVPPDSGKSICICNNVSELLMAMQDELDQMSMKHQE---LLNQIKETESHSVF
ENAECELEHLVKKMEIKVEQISKLKKHQDNVR--KLQQKVQNSKM-SEASGIRQEDNYPNGSNNLKNS--PR
KCLLNNP-LQK-SSNFHPIR-VRNLQMKLRRDDIMWEQ
>XP_046532784.1/1-473 centrosomal protein CEP57L1 isoform X1 [Equus
quagga]XP_046532785.1 centrosomal protein CEP57L1 isoform X2 [Equus
quagga]XP_046532786.1 centrosomal protein CEP57L1 isoform X1 [Equus
quagga]XP_046532787.1 centrosomal protein CEP57L1 isoform X1 [Equus
quagga]XP_046532788.1 centrosomal protein CEP57L1 isoform X1 [Equus
quagga]XP_046532789.1 centrosomal protein CEP57L1 isoform X1 [Equus
quagga]XP_046532790.1 centrosomal protein CEP57L1 isoform X1 [Equus
quagga]XP_046532791.1 centrosomal protein CEP57L1 isoform X1 [Equus
quagga]XP_046532792.1 centrosomal protein CEP57L1 isoform X2 [Equus quagga]
------------------------------------------------------------------------
------------------------------------------------------------------------
------------------------------------------------------------------------
-----------------------------------------------------------------MI-----
-----------------------------------------------MDPELMHSIV----GSYLKTP----
-E-------RV-PVPSFTQNDQS-----APTHH--SV-----SLEVNLPKMLHSPN-------SQ-------
-------ALILALKTLQEKIHRLDLERTQAEDNLNVLSREAAQYKKALENETNERNLAHQELIKQKKDITIQ
LSSAQSRCTLLEKQLEYTKRMVLNVEREKNMILEQQRSF---GGKLTPQKYQ--------------------
----VFQSMNMAQLQREKEQDHLKLQAKLEKLDVIEKECFKLTTTQKTAEDKIKYLEEKLKEEEHHRKLFQD
```

KASELQTGLEINRILMSSVSNPKRSKE---KKKSS-------------------------------------
----------------------K------SASAS-HSVKASR-QNLLQTMQHYG-P--HILPKRAEVTEPRR
LYK----PTRS-TS-QR-KAVPPDSGKSICICNNVSELLMAMQDELDQMSMKHQE---LLNQIKETESHSVF
ENAECELEHLVKKMEIKVEQISKLKKHQDNVR--KLQQKVQNSKM-SEASGIQQEDNYPNGSNNLKNS--PR
KCLLNNP-LQK-SSNFHPIR-VRNLQMKLRRDDIMWEQ
>KAI5929711.1/1-451 Centrosomal protein CEP57L1 [Manis javanica]

--MFV-----
---MDSELMHSIV----GSYLKPP----
-E-------RV-VVPSFTESDES-----AQTHR--PV-----NLEVTSPKTLHSPN-------SQ-------
-------ALILALKALQEKIHRLELERTQAEDNLTILSREAAQYKKALDSETNERNLAQQELMKQKKDVSTQ
LSAAQAHCALLEKQLACAKRMVLNAEREKSMILEQQ------------------------------------
-----------AQLQREKEKDRLKLQAKLEKLSVLEKECFKLTATQKTAEDRIKHLEGKLQEEEHHRELFRD
RAAELQTGLEINRILMSSVSNPKRSKD----KKAS-------------------------------------
----------------------K------SARAS-HSVNVSV-QSLLQTMQHCG-P--RGLQGPAVAAGPRC
PRR----PTRA-AS-QG-AAVP-DAEKSISMCDCLSELLIAMHDELDQMGLKHQE---LLNQMEETESGSVC
EDIECELERLVKKMEVKGEQISKLMRHQDNVR--KLQQKIQDSKM-SEASGIQQEDSNSKGSKNMKNS--PR
KCLLTDS-LQK--NNFHPFR-VRNLQMKLRRDDILWEQ
>XP_036758816.2/1-470 centrosomal protein CEP57L1 isoform X1 [Manis pentadactyla]

---MDSELMHSIV----GSYLKPP----
-E-------RV-FVPSFTESDES-----AQTHR--PV-----NLEVTSPKMLHSPN-------SQ-------
-------ALILALKALQEKIHRLELERTQAEDNLTVLSREAAQYKKALDSETNERNLAQQKLMKQKKDISAQ
LSSAQSRCALLEKQLACAKRMVLNAEREKRMILEQQ------------------------------------
-----------AQRQREKEKDRLKLQAKLEKLNVLEKECFKLTTTQKTAEDKIKHLEGKLQEEEHHRKLFRD
RAAELQTGLEINRILMSSVSNPKRSKD----KKASK--------------------------KSKCLNKGPP
QQIQSRFGSLPLVTE-------K------SARAI-HSMNVSV-----QTMQHCG-P--RGLQGPAQAAEPRC
PRR----PTRA-AS-QG-AAVP-DAEKSISMCDCLSELLIAMQDELDQMGLEHQE---LLNQMEETESGSVC
EDIECELQHLVKKMEIKGEQISKLMRHQDNVR--KLQQKIQNSKM-SEASGIQQEDSNYKGSKNIKKS--PR
KCLLTNS-LQK--NNFHPLR-VCNLQMKLRRDDIMWEQ
>XP_036868133.1/1-477 centrosomal protein CEP57L1 isoform X1 [Manis javanica]

--MFV-----
---MDSELMHSIV----GSYLKPP----
-E-------RV-VVPSFTESDES-----AQTHR--PV-----NLEVTSPKTLHSPN-------SQ-------
-------ALILALKALQEKIHRLELERTQAEDNLTILSREAAQYKKALDSETNERNLAQQELMKQKKDVSAQ
LSAAQAHCALLEKQLACAKRMVLNAEREKSMILEQQ------------------------------------
-----------AQLQREKEKDRLKLQAKLEKLSVLEKECFKLTTTQKTAEDRIKHLEGKLQEEEHHRELFRD
RAAELQTGLEINRILMSSLSNPKRSKD----KKASK--------------------------KSKCLNKGLP
QRIQSRFGLLPLETG-------K------SARAS-HSVNVSV-QSLLQTMQHCG-P--RGLQGPAEAAEPRC
PRR----PTRA-AS-QG-AAVP-DAEKSISMCDCLSELLIAMHDELDQMGLKHQE---LLNQMEETESGSVC
EDIECELERLVKKMEVKGEQISKLMRHQDNVR--KLQQKIQDSKM-SEASGIQQEDSNSKGSKNMKNS--PR
KCLLTDS-LQK--NNFHPFR-VRNLQMKLRRDDILWEQ
>XP_030879748.1/1-441 centrosomal protein CEP57L1 [Leptonychotes weddellii]

---MI-----
---MDSELMHSIV----GSYLKPP----
-E-------RV-FIPSFTQNDES-----SQTHH--SA-----NLEATSPKMLHSPN-------SQ-------
-------ALILALKTLQEKIHRLELERTQAEDNLNILSREAAQYKKALEKETNERNLAHQELIKQKKDITIQ
LSSAQSRCTLLEKQLEYTKRMVLNVEREKNMILEQQ------------------------------------
--DKIKHLEEKLKEEEHQRKLFQD

KASELQTGLEINRILMSSVSNPKHCKEK--KKKSLK-------------------------KTRCLKGGPP
QQIHSEFGSLPVVAE-------K------SSSAN-YPVNASM-QSLLQLMQHYG-P--HNCQKLTEVTEPRC
LCK----PTRT-TS-QG-KAEPSDSKNSISIGDNLSELLMAMQDELDQMSMEHEE---LLNQMKETESHLVC
EDIECELEHLVKKMEIKGEQISKLLKHQDNVR--KLQQKVQNSKM-SEASGIQREESNLKGSKNIKNS--PR
KCLLTNS-LQK-NSNFHPVQ-VHNLQMKLRRDDIMWEQ
>XP_059257159.1/1-424 centrosomal protein CEP57L1 isoform X7 [Mustela nigripes]

--MR-------
-------SLILALKTLQEKIHRLELERTQAEDNLNILSREAAQYKKALEKETNERILAHQELIKQKKDITIQ
LNSAQSRCTLLEKQLEYTKRMVLNVEREKNMILEQQ------------------------------------
-----------AQLQREKEQDHRKLQAKLEKLDVLEKECFKLTTTQKTAEDKIKHLEEKLKEEEHQRKLFQD
KASELQTGLEINRILMSSVSNPKHSKE----KKSLK-------------------------KTKCLKGGPP
QQTYSKFGSLPIVAE-------K------SASAS-CSVNSSM-QSLLQLMQHYG-P--HTCQKLPEVTEPPC
LYK----PLRT-TS-QG-KAEPSDSKISISIGDSLSELLMAMQDELDQMSMEHEE---LLNQMKDTESHSVC
EDIECELEHLVKKMEIKEEQISKLLKHQANVR--KLQQKVQNSKM-SEASGIQREENNLKGSKPIKNS--PR
KCLLTNS-LQK-NSNFHPVQ-VHNLQMKLRRDDIMWEQ
>XP_045654391.1/1-444 centrosomal protein CEP57L1 isoform X4 [Ursus americanus]XP_045654392.1 centrosomal protein CEP57L1 isoform X4 [Ursus americanus]XP_045654394.1 centrosomal protein CEP57L1 isoform X4 [Ursus americanus]XP_045654395.1 centrosomal protein CEP57L1 isoform X4 [Ursus americanus]

---MQSPM----G----------
-----------------SRQYLS-----SN-----------NLE----EWRH-------------------
-------ALILALKTLQEKIHRLELERTQAEDNLNVLSREAAQYKKALEKETNERNLAHQELVKQKKDITIQ
LSSAQSRCTLLEKQLEYTKRMVLNVEREKNMILEQQ------------------------------------
-----------AQLQREKEQDHRKLQAKLEKLDVLEKECFKLTTTQKTAEDKIKHLEEKLKEEEHQRKLFQD
KASELQTGLEINRILMSSVSNPKRSKE---KKKSLK-------------------------KTKCLKGGPP
QQIYSKLGSPPITAE-------K------SSSAS-SSVNASM-QSLLQLMQHYR-P--HTCQKLTEVTEPRC
LYK----PTRT-TS-QG-KAEPSDSKNSISIGDNLSELLMAMQDELDQMSMEHEE---LLNQMKETESHSVC
EDIECELEYLVKKMEIKGEQISKLMKHQDNVR--KLQQKVQNSKM-NEASGIQREESNLKGSKNIKNS--PR
KCLLTNS-LQK-NSNFHPVQ-VHNLQMKLRRDDIMWEQ
>XP_032263998.1/1-445 centrosomal protein CEP57L1 isoform X4 [Phoca vitulina]XP_032263999.1 centrosomal protein CEP57L1 isoform X4 [Phoca vitulina]XP_032264000.1 centrosomal protein CEP57L1 isoform X4 [Phoca vitulina]XP_032264001.1 centrosomal protein CEP57L1 isoform X4 [Phoca vitulina]

---MQSPM----GS---------
---------RQ-YLSS---NNPE-----GWRH--
-------ALILALKTLQEKIHRLELERTQAEDNLNSLSREAAQYKKALEKETNERNLAHQELIKQKKDITIQ
LSSAQSRCTLLEKQLEYTKRMVLNVEREKNMILEQQ------------------------------------
-----------AQLQREKEQDHRKLQAKLEKLDVLEKECFKLTTTQKTAEDKIKHLEEKLKEEEHQRKLFQD
KASELQTGLEINRILMSSVSNPKHCKEK--KKKSLK-------------------------KTRCLKGGPP
QQIHSKFGSLPAVAE-------K------SASAN-YPVDASM-QSLLQLMQHYG-P--HNCQKLTEVTEPRC
LYK----PTRT-TS-QG-KAEPSDSKNSISIGDNLSELLMAMQDELDQMSMEHEE---LLNQMKETESHSVC
EDIECELEHLVKKMEIKGEQISKLLKHQDNVR--KLQQKVQNSKM-SEASGIQREDSNLKGSKNIKNS--PR
KCLLTNS-LQK-NSNFHPVQ-VHNLQMKLRRDDIMWEQ
>XP_054361463.1/1-445 centrosomal protein CEP57L1 isoform X4 [Mirounga angustirostris]XP_054361464.1 centrosomal protein CEP57L1 isoform X4 [Mirounga angustirostris]XP_054361465.1 centrosomal protein CEP57L1 isoform X4 [Mirounga

angustirostris]XP_054361466.1 centrosomal protein CEP57L1 isoform X4 [Mirounga angustirostris]

--MQSPM----GS---------
---------RQ-YLSS---NNPE-----GWRH----------------------------
-------ALILALKTLQEKIHRLELERTQAEDNLNILSREAAQYKKALEKETNERNLAHQELIKQKKDITIQ
LSSAQSRCTLLEKQLEYTKRMVLNVEREKNMILEQQ------------------------
-----------AQLQREKEQDHRKLQAKLEKLDVLEKECFKLTTTQKTAEDKIKHLEEKLKEEEHQRKLFQD
KASELQTGLEINRILMSSVSNPKHCKEK--KKKSLK------------------------KTRCLKGGPP
QQIHSEFGSLPVVAE-------K------SASAN-YPVNASM-QSVLQLMQHYG-P--HNCQKLTEVTEPRC
LYK----PTRT-TS-QG-KAEPSDSKNSISIGDNLSELLMAMQDELDQMSMEHEE---LLNQMKETESHLVC
EDIECELEHLVKKMEIKGEQISKLLKHQDNVR--KLQQKVQNSKM-SEASGIQREESNLKGSKNIKNS--PR
KCLLTNS-LQK-NSNFHPVQ-VHNLQMKLRRDDIMWEQ
>XP_013848199.1/1-429 centrosomal protein CEP57L1 isoform X5 [Sus scrofa]

------------------------------------MDSVSL------------------

-------SLILALKTLQEKIYRLELERTQAEDNLNILSREAAQYKKALENETSERNLAHEELIKQKKDISMQ
LSSAQSRCTLLEKQLEYTKRMVLNVEREKDMILEQQ------------------------
-----------AQLQREKEQDHMKLQAKLEKLDVLEKECFKLTTTQKTAEDKIKHLEEKLKEEEHQRKLFQD
KASQLQTGLAINRILMASASNPKCSKE---KKKSSK------------------------KTKCLKRGPP
QQIYSKFGSLPVVAE-------K------SASAS-HSVNAST-QNLLQTMQHYG-P--HILQKPAKVIEPRY
LYK----PSRT-TS-QC-KAVPSDSEKSISICDNLSELLMAMQDELDQMSIEYQE---LLSQMKETESQSVC
EDIECELEHLVKKMEIKGERISKLMKHQDSVR--KLQQKVQSSKM-SEASAIQQEDSNLKGSKNIKNS--PR
KCLLTNS-LQK-NSSFHPIR-VHNLQMKLRRDDILWEQ
>XP_058163233.1/1-429 centrosomal protein CEP57L1 isoform X5 [Dasypus novemcinctus]

------------------MWEA--------------------------------------
-------ALFLALKTLQDKIHRLELERTQAEDNLNILSREAAQYKKALENETNERNLAHQELIKQKKDISIQ
LSSAQSRCTLLEKQLEYTKRMVLNAEREKNMVLEQQ------------------------
-----------AQLQREKEQDHMMLHAKLEKLDVLEKECFRLTTTQKTAEDKIKHLEEKLKEEEHQRKLFQD
KASELQTGIEINRILMSSVSKPKYSKE---MKKSSK------------------------KTKCLKRGLP
LQIYSKFGVLPFVAG-------K------SASAS-HSVNANM-QNFLQMTQHCD-Q--RILQKNSEVTEPRC
LFR----PSRRGPS-QC-KAVPPDSEKSISVCDNLSELLMAMQDELEQMSMEHQE---ILNQMKETESRSIC
EDIECELERLVKKMEIKEEQISKLKKHQDSVR--KLQDKVQNLRM-NEASDIQREDSSPKRAKNIKNS--PR
KCLNQTNPPQK-NSNLHPKQ-VHNLVMKLRKDDIMWEQ
>XP_055246717.1/1-440 centrosomal protein CEP57L1 isoform X4 [Gorilla gorilla gorilla]

-------------------------------------MNEWKNDIM--------------
-----------------PQNFQR------WDCL--VA-----KTSSGIPLIL----------LY------
-------ALILALKTLQEKIHRLELERTQAEDNLNILSREAAQYKKALENETNERNLAHQELIKQKKDISIQ
LSSAQSRCTLLEKQLEYTKRMVLNVEREKNMILEQQ------------------------
-----------AQLQREKEQDQMKLYAKLEKLDVLEKECFRLTTTQKTAEDKIKHLEEKLKEEEHQRKLFQD
KASELQTGLEISKIIMSSVSNLKHSKQ---KKKSSK------------------------KTKCIKRGPP
WQICSKFGALPFVAE-------K------------------------MRQHRD-P--HILQKPFNVTETRC
LPK----PSRT-TF-WC-KAIPPDSEKSISICDNLSELLMAMQDELDQMSMEHQE---LLKQMKETESHSVC

DDIECELECLLKKMEIKGEQISKLKKHQDSVC--KLQQKVQNSKM-SEASGIQQEDSYPKGSKNIKNS--PR
KCLTDTNLFQK-NSSFHPIR-VHNLQMKLRRDDIMWEQ
>XP_014200928.1/1-457 centrosomal protein CEP57L1 isoform X3 [Pan paniscus]XP_016811602.1 centrosomal protein CEP57L1 isoform X13 [Pan troglodytes]

--MNEWKNDIM---------------
-----------------PQNFQR------WDCL--VA-----KTSSGIPPIL-----------LY-------
-------ALILALKTLQEKIRRLELERTQAEDNLNILSREAAQYKKALENETNERNLAHQELIKQKKDISIQ
LSSAQSRCTLLEKQLEYTKRMVLNVEREKNMILEQQ------------------------------------
-----------AQLQREKEQDQMKLYAKLEKLDVLEKECFRLTTTQKTAEDKIKHLEEKLKEEEHQRKLFQD
KASELQTGLEISKIIMSSVSNLKHSKE---KKKSSK--------------------------KTKCIKRRPP
WQICSKFGALPFVAE-------K------VRGDK-MKIVVQK-NSCFVMRQHRD-P--HILQKPFNVTETRC
LPK----PSRT-TS-WC-KAIPPDSEKSISICDNLSELLMAMQDELDQMSMEHQE---LLKQMKETESHSVC
DDIECELECLLKKMEIKGEQISKLKKHQDSVC--KLQQKVQNSKM-SEASGIQQEDSYPKGSKNIKHS--PR
KCLTDTNLFQK-NSSFHPIR-VHNLQMKLRRDDIMWEQ
>XP_025237736.1/1-444 centrosomal protein CEP57L1 isoform X7 [Theropithecus gelada]

--M-----
---RDLEQGTSAS----GGSKLAPWGGF
RS-------RM-F-----------------GVC--PG-----EGE------------------GK-------
-------TLILALKTLQEKIHHLELERTQAEDNLNILSREAAQYKKALENETNERNLAHQELIKQKKDISIQ
LSSAQSRCTLLEKQLEYTKRMVLNVEREKNMILEQQ------------------------------------
-----------AQLQREKEQDQIKLYAKLEKLDVLEKECFRLTTTQKTAEDKIKHLEEKLKEEEHQRKLFQD
KASELQTGLEISKIIMSSVSNLKHSKE---KKTSSK--------------------------KTKCIKRGPP
WQICSKFGALPFVAE-------K-------------------------RRQHRG-P--HILQKPFNVAETRC
LPR----PSRT-TS-WC-KAIPRDSEKSISIFDNLSELLMAMQDELDQMSMEHQE---LLKQMKETESHSVC
DDIECELECLVKKMEIKGEQISKLKKHQDSVR--KLQQKVQNSKM-SEASGVQQEDSNPKGSKNIKNS--PR
KCLTDTKLFQK-NSSFHPIR-VHNLQMKLRRDDIMWEQ
>XP_055981414.1/1-485 centrosomal protein CEP57L1 isoform X1 [Sorex fumeus]

---ME-------------------

--LYGEI-----
---MDSELMNSIL----GSYLKPP----
-E-------RV-FVPSFTQNDES-----SQNHQ--SM-----NLEITSPRVLHSPN-------RQ-------
-------ALILALKTLQEKIHRLELERTQAEDNLNSLSREAAQYKKALENETNERNLAYQELIKQKKNISVQ
LSSAQSRCILLEKQLEYTKRMVLNVEREKNMILEQQ------------------------------------
-----------AHLQREKEQDQLKLQAKLEKLDILEKECFKLTTTQKTAEDKIKYLEEKLTEEGHLRKLIQD
KAAELQTGLEVNRILMSSVSNPNCSKG---KRKSSK--------------------------KTKCLKRAPP
QQTYSTSGPQSFVSE-------K------YACANLHSVNAGV-QNLLQNSQHYR-PRTHSLPAPTAVNEAKH
FYK----STRT-AS-QC-TSAPSDSEKFTSICDNLSELLKAMQDELNQMSMEHQE---LWKQMKETKNPSVC
EDIGGKLEYLVKKMEIKGEQISKLMKHQKSVH--KLQQKVQNSMM-ITTSDTQQEDNNPKGSKNIKFN--PR
KCSLTNS-PQK-NS--HPVR-VQNLQMKLRRDDIMWEQ
>XP_054992528.1/1-516 centrosomal protein CEP57L1 isoform X3 [Sorex araneus]

---MEH------------------
---PE---SLPKADYTQG
RH---QAR-PCHQ------AHKSRGRG---------------------------------IPVFLEI-----
---MDSELMNSML----GSYLKPP----
-E-------RV-FVPSFTQNDES-----SQNHQ--SV-----NLELTSPRVLHSPN-------QQ-------
-------ALILALKTLQEKIHRLELERTQAEDNLNSLSREAAQYKKALENETNERNLAYQELIKQKKNISVQ
LSSAQSRCILLEKQLEYTKRMVLNVEREKNMILEQQ------------------------------------
-----------AHLQREKEQDQLKLQAKLEKLDILEKECFKLTTTQKTAEDKIKYLEEKLMEEGHLRKLIQD
KAAELQTGLEVNRILMSSVSNPNCSKG---KRKSSK--------------------------KTKCLKRAPP

QQTYSTFGPQSFVSE-------K------YACANLHSVNAGI-QNLPQNSQHYR-PCTHSLPIPIAVNDAKH
-DK----STRT-AS-QC-TSAPSDSEKFTSICDNLSELLKAMQDELNQMSMEHQE---LWKQMKETKSPSVC
EDIGGKLEYLVKKMEIKGEQISKLMKHQKNVH--KLQQKVQNSMM-IATSDIQQRDSNPKGSKNIKIS--PR
KCSLTNS-PQK-NS--HPVR-VQNLQMKLRRDDIMWEQ
>XP_008850657.1/1-432 centrosomal protein CEP57L1 isoform X4 [Nannospalax galili]

-----------------MLNTGI------DKLY--DI-----CT----------------------------
--------LVLALKTLQEKIHRLELERAQAEDNLNVLSREAAQYKKALESETNERNLAHQELIKQKKDISIQ
LSSIQSHCILLEKQLEYTKRMVLNVEREKNMILEQQ------------------------------------
-----------AQLQREKEQDQMKLHAKLEKLDVLEKECFRLTTTQQTAEDKIKYLEEKLKEEEHQRKLFED
KACELQTGLEISKILMSSVSNSKYWKE----KKPSK-------------------------KTKYFKKGPP
QQMYSKFRAPSSESE-------K------LVSAG-CSVNANL-QNLLQMVHHCG-P--YSLQKPAEVTEARC
LYK----PLRG-TS-QC-KAIPRNSERPVSICDNLSELLVAMQDELDQMSLEHKE---LLQQMKQTGSHLVC
DDIDQELEQLVKKMEIKGEQISKLKKHQDSVR--KLQQKFQ-----NLSSGIHQEHSNPKGSKNIKNS--PR
RCLNETNPSQK-SSNFHPMQ-VHNLQVKLRRDDITWEQ
>XP_017658502.1/1-477 centrosomal protein CEP57L1 isoform X1 [Nannospalax galili]

---MTFVI-----
--MDSELSHSVV----GSYLKPP----
-Q-------RM-FIPSFTENEPF------QNCY--AV-----NSEVTSSKMFNSPN-------SQ-------
-------ALVLALKTLQEKIHRLELERAQAEDNLNVLSREAAQYKKALESETNERNLAHQELIKQKKDISIQ
LSSIQSHCILLEKQLEYTKRMVLNVEREKNMILEQQ------------------------------------
-----------AQLQREKEQDQMKLHAKLEKLDVLEKECFRLTTTQQTAEDKIKYLEEKLKEEEHQRKLFED
KACELQTGLEISKILMSSVSNSKYWKE----KKPSK-------------------------KTKYFKKGPP
QQMYSKFRAPSSESE-------K------LVSAG-CSVNANL-QNLLQMVHHCG-P--YSLQKPAEVTEARC
LYK----PLRG-TS-QC-KAIPRNSERPVSICDNLSELLVAMQDELDQMSLEHKE---LLQQMKQTGSHLVC
DDIDQELEQLVKKMEIKGEQISKLKKHQDSVR--KLQQKFQ-----NLSSGIHQEHSNPKGSKNIKNS--PR
RCLNETNPSQK-SSNFHPMQ-VHNLQVKLRRDDITWEQ
>XP_006881222.1/1-459 PREDICTED: centrosomal protein CEP57L1 [Elephantulus edwardii]

--MDSELMHSVV----GSYLKPP----
-E-------KV-CVPSFTQHETA-----KNYCHV--------NVDISPPKALHGPN-------SQ-------
-------ALILALRTLQDKIRRLELERTQAEDNLNILTREAAEYKKALENETNERNLAHQELIKQKKDISIQ
LSSAQSRCTLLERQLEYTKRMVLNAEREKTIILEQQ------------------------------------
-----------AQLQKEKEQDHMKLHAKLEKLDVLEKECFRLTATQKTAEDKIKLLEEKLKEEEHHRKLFQD
KACELQTGVEINRILMSSVSNLTHLRK---KKKYSK-------------------------KTKCLKSELP
QQSYSKFGAPSFMSG-------K------------------------MMQNCD-L--HTFLKHAKATDPRC
LQKHSK------IP-QS-RAAPPDPEKFIPIYDNLSELLTAMQDELDQMNMEHQE---LLNQMKETQSPSVC
EDIGNELEHLVKKMEIKREQISKLKKHQDSVH--KLQQKLQNPKM-SEASAILQEDSNSKGSKNLKNS--PG
KRSHETSSLQK-TSHFHPKR-VHNLQMKLRKDDIMWEQ
>XP_017734254.1/1-427 PREDICTED: centrosomal protein CEP57L1 [Rhinopithecus bieti]

---MP-------------
-------R-RRS----LSPVLE---------------------------------------SQI-----
--MDSELMHSIV----GSYHKPP----
-E-------RV-FVPSFTQNEPS------QNCH--PA-----NLEVTSPKILHSPN-------SQ-------
-------G------------------------NADI------------------------EFEECFIDISIQ

LSSAQSRCTLLEKQLEYTKRMVLNVEREKNMILEQQ------------------------------------
-----------AQLQREKEQDQMKLYAKLEKLDVLEKECFRLTTTQKTAEDKIKHLEEKLKEEEHQRKLFQD
KASELQTGLEISKIIMSSVSNLKHSKQ---KKTSSK-------------------------KTKCIKRGPP
WQICSKFGALPFVAE-------K------------------------RRQHRG-P--HILQKPFNVAETRC
LPR----PSRT-TS-WC-KAIPPDSEKSISISDNLSELLMAMQDELDQMSMEHQE---LLKQMKETESHSVC
DDIECELECLVKKMEIKGEQISKLKKHEESVR--KLQQKVQNSKM-SEASGIQQEDSNPKGSKNIKQS--PR
KCLTDTNLFQK-NSSFHPIR-VHNLQMKLRRDDIMWEQ
>XP_005377908.1/1-564 PREDICTED: centrosomal protein CEP57L1 isoform X1
[Chinchilla lanigera]
---MRLRGTPSG------
--AGGVFHS--------------
---RTFWSRWPGEWIRKAS---S--
-------R-KTSTLRLLSHTQEQHIK--------------------------------TTTIRL-----
---KKAESKFKITLKKCGSFFIMP----
-KPGLGSLGRV-KAAPLTSGKCSF----AAACA--AVCDRNYNPQEPPRRCLSQPA-------NQRFWRQEL
SELGGRSALILALKTLQEKIHRLELERTQAEDNLNILSREAAQYKKALENETNERNLAHQELIKQKKDISIQ
LSSAQSRCTLLEKQLEYTKRMVLNVEREKNMILEQQ------------------------------------
-----------AQLQREKEQDQMRLHAKLEKLDVLEKECFRLTTTQKTAEDKIKHLEEKLKEEEHQRKLFQD
KAAELQTGLEISKILMSSVSNSKRSKE---KKKSSK-------------------------KTKCLKRGPP
LQIYSKFRAPLFEAK-------K------SASAG-CSVNAGR-QNLQQTTHHCG-P--QILPKRAQVIEPRC
LCK----PPRA-IS-QY-KAVPRESEKSISICDSLSELLMAMQDELDQMSMEHAE---LLKQMKETETHSMC
DDVECELERLVKKMEIKGKQISRLKKHQDSVY--KLQQKVQNSKA-SKASGIQQDGN--QELKNMKNS--PR
KCLNEANSSQK-SSNFHPMQ-VHNIRMKLRRDDIMWEQ
>Q8VDS7.1/1-400 RecName: Full=Centrosomal protein CEP57L1; AltName:
Full=Centrosomal protein 57kDa-like protein 1; AltName: Full=Centrosomal
protein of 57 kDa-related protein; Short=Cep57R; AltName: Full=Cep57-related
protein [Mus musculus]AAH21375.1 RIKEN cDNA 2410017P07 gene [Mus musculus]

---MDSELSQSMV----GSYLNPP----
-E-------RM-HLPSFTQNEAF------QNCH--PG-----TP----PKMFNSPN-------NQ-------
-------ALVSALKTLQEKIRRLELERTQAEDNLNLLSREAAQYKKALEEETNERNLAHQELIKQKKDISIQ
LSSAQSRCILLEKQLEYTKRMVLNVEREKTMILEQQ------------------------------------
-----------AQLQREKEQDQMKLHAKLEKLHVLEKECLRLTATRQTAEDKIKCLEEKLKEEEHQRRLFQD
RACE---KTNCLKREPP
QQRDHKFRTPTFER--
-KK----PFRT-TS-QA-RANPQSSGEPVSICDSLSELLMTMEEELDQMNMEHRE---LLRQTMQPGNHSVS
EDIEQELEQLAKKMESKGDQISKLKKHQDSVR--KLQEKIENSRI-NESSGIH---GNPKGSKNLKTS--PR
KCVSETSSFQR-DRGFQPVQ-VHSLQSKLRRDDIKWEQ
>XP_036011351.1/1-481 centrosomal protein CEP57L1 isoform X1 [Mus musculus]

--MS--------------------
--SEIR-EKPS--CSAC
-----LCR-EFGCA--SHRADSDAEAGA------------------------------GLQGSRDA-----
-------------------TPR------------------VEVQAETMDSELSQSMV----GSYLNPP----
-E-------RM-HLPSFTQNEAF------QNCH--PG-----TP----PKMFNSPN-------NQ-------
-------ALVSALKTLQEKIRRLELERTQAEDNLNLLSREAAQYKKALEEETNERNLAHQELIKQKKDISIQ
LSSAQSRCILLEKQLEYTKRMVLNVEREKTMILEQQ------------------------------------
-----------AQLQREKEQDQMKLHAKLEKLHVLEKECLRLTATRQTAEDKIKCLEEKLKEEEHQRRLFQD
RACELQTGFEISKILMSTVSNSKHCKE---KKKQPK-------------------------KTNCLKREPP
QQRDHKFRTPTFER--
-KK----PFRT-TS-QA-RANPQSSGEPVSICDSLSELLMTMEEELDQMNMEHRE---LLRQMMQPGNHSVS
EDIEQELEQLAKKMESKGDQISKLKKHQDSVR--KLQEKIENSRI-NESSGIH---GNPKGSKNLKTS--PR
KCVSETSSFQR-DRGFQPVQ-VHSLQSKLRRDDIKWEQ
>XP_036011358.1/1-438 centrosomal protein CEP57L1 isoform X8 [Mus musculus]

---M-ALV-------
--KICM-----

--TMDSELSQSMV----GSYLNPP----
-E-------RM-HLPSFTQNEAF------QNCH--PG-----TP----PKMFNSPN-------NQ-------
-------ALVSALKTLQEKIRRLELERTQAEDNLNLLSREAAQYKKALEEETNERNLAHQELIKQKKDISIQ
LSSAQSRCILLEKQLEYTKRMVLNVEREKTMILEQQ------------------------------------
-----------AQLQREKEQDQMKLHAKLEKLHVLEKECLRLTATRQTAEDKIKCLEEKLKEEEHQRRLFQD
RACELQTGFEISKILMSTVSNSKHCKE---KKKQPK--------------------------KTNCLKREPP
QQRDHKFRTPTFER--
-KK----PFRT-TS-QA-RANPQSSGEPVSICDSLSELLMTMEEELDQMNMEHRE---LLRQMMQPGNHSVS
EDIEQELEQLAKKMESKGDQISKLKKHQDSVR--KLQEKIENSRI-NESSGIH---GNPKGSKNLKTS--PR
KCVSETSSFQR-DRGFQPVQ-VHSLQSKLRRDDIKWEQ
>XP_036011355.1/1-447 centrosomal protein CEP57L1 isoform X5 [Mus musculus]

---MT-------------------
---NARM-THY-------
--QSLLL-----
-------------------TKQ------------------------TMDSELSQSMV----GSYLNPP----
-E-------RM-HLPSFTQNEAF------QNCH--PG-----TP----PKMFNSPN-------NQ-------
-------ALVSALKTLQEKIRRLELERTQAEDNLNLLSREAAQYKKALEEETNERNLAHQELIKQKKDISIQ
LSSAQSRCILLEKQLEYTKRMVLNVEREKTMILEQQ------------------------------------
-----------AQLQREKEQDQMKLHAKLEKLHVLEKECLRLTATRQTAEDKIKCLEEKLKEEEHQRRLFQD
RACELQTGFEISKILMSTVSNSKHCKE---KKKQPK--------------------------KTNCLKREPP
QQRDHKFRTPTFER--
-KK----PFRT-TS-QA-RANPQSSGEPVSICDSLSELLMTMEEELDQMNMEHRE---LLRQMMQPGNHSVS
EDIEQELEQLAKKMESKGDQISKLKKHQDSVR--KLQEKIENSRI-NESSGIH---GNPKGSKNLKTS--PR
KCVSETSSFQR-DRGFQPVQ-VHSLQSKLRRDDIKWEQ
>XP_036011356.1/1-445 centrosomal protein CEP57L1 isoform X6 [Mus musculus]

---MP-------------------

--GDVYR-----
-------------------TRK------------------AE-QIKTMDSELSQSMV----GSYLNPP----
-E-------RM-HLPSFTQNEAF------QNCH--PG-----TP----PKMFNSPN-------NQ-------
-------ALVSALKTLQEKIRRLELERTQAEDNLNLLSREAAQYKKALEEETNERNLAHQELIKQKKDISIQ
LSSAQSRCILLEKQLEYTKRMVLNVEREKTMILEQQ------------------------------------
-----------AQLQREKEQDQMKLHAKLEKLHVLEKECLRLTATRQTAEDKIKCLEEKLKEEEHQRRLFQD
RACELQTGFEISKILMSTVSNSKHCKE---KKKQPK--------------------------KTNCLKREPP
QQRDHKFRTPTFER--
-KK----PFRT-TS-QA-RANPQSSGEPVSICDSLSELLMTMEEELDQMNMEHRE---LLRQMMQPGNHSVS
EDIEQELEQLAKKMESKGDQISKLKKHQDSVR--KLQEKIENSRI-NESSGIH---GNPKGSKNLKTS--PR
KCVSETSSFQR-DRGFQPVQ-VHSLQSKLRRDDIKWEQ
>XP_021029765.1/1-445 centrosomal protein CEP57L1 isoform X2 [Mus caroli]

---MP-------------------

--GDVYR-----
-------------------TRK------------------TE-QIKTMDSELSQSMV----GSYLNPP----
-E-------RM-YLPSFTQNEAF------QNCH--PG-----TP----PKMFNSPN-------NQ-------
-------ALVSALKTLQEKIRRLELERTQAEDNLNLLSREAAQYKKALEEETNERNLAHQELIKQKKDISIQ
LSSAQSRCILLEKQLEYTKRMVLNVEREKTMILEQQ------------------------------------
-----------AQLQREKEQDQMKLHAKLEKLHVLEKECLRLTATQQTAEDKIKCLEEKLKEEEHQRRLFQD
RACELQTGFEISKILMSTVSNSKHCKK---KKKQPK--------------------------KINCLKREPP
QQRDHKFRRPTFES--
-KK----PFRT-TS-QA-RANPQSSGEPVSICDSLSELLMTMEEELDQMNMEHRE---LLRQMIQPGNHSVS
EDIEQELEQLAKKMESKGDQISKLKKHQDSVR--KLQEKIENSRI-NESSGVH---GNPKGSKNLKTS--PR
KCVSETSSFQR-DRRFQPVQ-VHSLQTKLRRDDIKWEQ
>XP_036011353.1/1-455 centrosomal protein CEP57L1 isoform X3 [Mus musculus]

---MP-------------------
---PRHL-SLPA---NQR
-----FWR-PW---LTGSVHL-----
--TMDSELSQSMV----GSYLNPP----

-E-------RM-HLPSFTQNEAF------QNCH--PG-----TP----PKMFNSPN-------NQ-------
-------ALVSALKTLQEKIRRLELERTQAEDNLNLLSREAAQYKKALEEETNERNLAHQELIKQKKDISIQ
LSSAQSRCILLEKQLEYTKRMVLNVEREKTMILEQQ------------------------------------
-----------AQLQREKEQDQMKLHAKLEKLHVLEKECLRLTATRQTAEDKIKCLEEKLKEEEHQRRLFQD
RACELQTGFEISKILMSTVSNSKHCKE---KKKQPK--------------------------KTNCLKREPP
QQRDHKFRTPTFER--
-KK----PFRT-TS-QA-RANPQSSGEPVSICDSLSELLMTMEEELDQMNMEHRE---LLRQMMQPGNHSVS
EDIEQELEQLAKKMESKGDQISKLKKHQDSVR--KLQEKIENSRI-NESSGIH---GNPKGSKNLKTS--PR
KCVSETSSFQR-DRGFQPVQ-VHSLQSKLRRDDIKWEQ
>XP_021029764.1/1-455 centrosomal protein CEP57L1 isoform X1 [Mus caroli]

---MP-------------------
---PRHL-SLPA---NQR
-----FWR-PW---LTGSVHL-----
-------------------T---------------------------MDSELSQSMV----GSYLNPP----
-E-------RM-YLPSFTQNEAF------QNCH--PG-----TP----PKMFNSPN-------NQ-------
-------ALVSALKTLQEKIRRLELERTQAEDNLNLLSREAAQYKKALEEETNERNLAHQELIKQKKDISIQ
LSSAQSRCILLEKQLEYTKRMVLNVEREKTMILEQQ------------------------------------
-----------AQLQREKEQDQMKLHAKLEKLHVLEKECLRLTATQQTAEDKIKCLEEKLKEEEHQRRLFQD
RACELQTGFEISKILMSTVSNSKHCKK---KKKQPK--------------------------KINCLKREPP
QQRDHKFRRPTFES--
-KK----PFRT-TS-QA-RANPQSSGEPVSICDSLSELLMTMEEELDQMNMEHRE---LLRQMIQPGNHSVS
EDIEQELEQLAKKMESKGDQISKLKKHQDSVR--KLQEKIENSRI-NESSGVH---GNPKGSKNLKTS--PR
KCVSETSSFQR-DRRFQPVQ-VHSLQTKLRRDDIKWEQ
>XP_052020247.1/1-445 centrosomal protein CEP57L1 isoform X4 [Apodemus sylvaticus]

---MPG------

----------------------------------DVYRTRKTEQIKTMDSELSHSMV----GSYLNPP----
-E-------RM-YLPSFTQNEAF------QNHH--LG-----NS----PKMFNSPN-------NQ-------
-------ALVSALKTLQEKIHRLELERTQAEDNLNLLSREAAQYKKALEEETTERDLAHEELIKQKKDISIQ
LSSAQSRCILLEKQLEYTKRMVLNVEREKTMILEQQ------------------------------------
-----------AQLQREKEQDQMKLHAKLEKLHVLEKECLRLTATQQTAEDKIKYLEEKLKEEEHQRRLFQD
KACELQTGFEISKILMSTVSNSKHCKE---KKKLPK--------------------------KTNCLKREPP
QQMDHKFRVPTFEK--
-EK----PFRA-TS-QA-RANAHSSGEPVSICDSLSELLMTMQEELAQMNMEHRE---LLRQMMQTESHSVS
EDIELELEQLVKKMESKGDQISKLKKHQDSVR--KLQEKVENSRI-NESSGLH---GHPKGSKNLKTS--PR
KCVSETSAFQR-DSSFQPVQ-VHSLQAKLRRDDIKWEQ
>XP_052020246.1/1-449 centrosomal protein CEP57L1 isoform X3 [Apodemus sylvaticus]

---MI-------------------
--T-PVPG--DP--
---KLSSC--------
-----------------------FHGY------------------QTMDSELSHSMV----GSYLNPP----
-E-------RM-YLPSFTQNEAF------QNHH--LG-----NS----PKMFNSPN-------NQ-------
-------ALVSALKTLQEKIHRLELERTQAEDNLNLLSREAAQYKKALEEETTERDLAHEELIKQKKDISIQ
LSSAQSRCILLEKQLEYTKRMVLNVEREKTMILEQQ------------------------------------
-----------AQLQREKEQDQMKLHAKLEKLHVLEKECLRLTATQQTAEDKIKYLEEKLKEEEHQRRLFQD
KACELQTGFEISKILMSTVSNSKHCKE---KKKLPK--------------------------KTNCLKREPP
QQMDHKFRVPTFEK--
-EK----PFRA-TS-QA-RANAHSSGEPVSICDSLSELLMTMQEELAQMNMEHRE---LLRQMMQTESHSVS
EDIELELEQLVKKMESKGDQISKLKKHQDSVR--KLQEKVENSRI-NESSGLH---GHPKGSKNLKTS--PR
KCVSETSAFQR-DSSFQPVQ-VHSLQAKLRRDDIKWEQ
>XP_021060543.1/1-429 centrosomal protein CEP57L1 isoform X1 [Mus pahari]

---MDSEFSHSMI----GSYLNPP----
-E-------RM-YLPSFTQNEAF------QNCQ--PG-----NS----PKMFNSPN-------NQ-------
-------ALVSALKTLQEKIRRLELERIQAEDNLNLLSREAAKYKKALEEETSERDLAHQELIKQKKDISIQ
LSSAQSRCILLEKQLEYTKRMVLNVEREKTMILEQQ------------------------------------
-----------AQLQREKEQDQMKLHAKLEKLHVLEKECLRLTATQRTAEDRIKYLEEKLKEEEHQRRLFQD
RACELQTGFEISKILMSTVSNSKHCKE---KKKLPK--------------------------KTNCLKREPP
QQMDHKFKVPTFER--
-EK----PFRT-TS-QA-RANPHSSGEPVSICDSLSELLMTMEEELDQMNMEHRE---LLRQMMQPGNHSVS
EDIEHELEQLVRKMESKGHQISKLKKHQDRVR--KLQEKIENARI-NESSGIH---GNPKGSKNLKTS--PR
KCVSETSSFQR-DRSFQPVQ-VHSLQTKLRRDDIKWEQ
>XP_031204127.1/1-431 centrosomal protein CEP57L1 isoform X4 [Mastomys coucha]XP_031204128.1 centrosomal protein CEP57L1 isoform X4 [Mastomys coucha]

---MTMDSELSHSMV----GSYLKPP----
-E-------RT-YLPSFTQNEAV------QNYH--PG-----NS----PKMFNSPN-------NQ-------
-------ALVSALKTLQEKIRCLELERTQAEDNLNLLSREAAQYKKALEEESNERNLAHEELIKQKKDISIQ
LSSARSRCILLEKQLEYTKRMVLSVEREKTMILEQQ------------------------------------
-----------TQLQREKEQDQMKLHAKLEKLHILEKECLKLTVTQQTAEDKIKYLEEKLKEEEHQRRLFQD
KACELQTGFEISKILMSTVSNSKHCKE---KKKLPK--------------------------KTNCLKRGPP
QPPDHKFRAPAFER--
-AK----PFRT-TA-QT-RANPHSSGEPVSICDSLSELLMTMQEELDQMNMEHRE---LLRQLMQTESQSVS
EDIEQELEQLVKKMESKGDQISLLKRHQDSVR--KLQEKVENSRI-NESSGIH---GNPKGSKNLKNS--PR
KCVSETSAFQR-NRSFQPVQ-VHSLQTKLRRDDIKWEQ
>XP_031204124.1/1-445 centrosomal protein CEP57L1 isoform X2 [Mastomys coucha]

--MPR------------D
-----VYRTRK---
---------------------------------------TEQ--IKTMDSELSHSMV----GSYLKPP----
-E-------RT-YLPSFTQNEAV------QNYH--PG-----NS----PKMFNSPN-------NQ-------
-------ALVSALKTLQEKIRCLELERTQAEDNLNLLSREAAQYKKALEEESNERNLAHEELIKQKKDISIQ
LSSARSRCILLEKQLEYTKRMVLSVEREKTMILEQQ------------------------------------
-----------TQLQREKEQDQMKLHAKLEKLHILEKECLKLTVTQQTAEDKIKYLEEKLKEEEHQRRLFQD
KACELQTGFEISKILMSTVSNSKHCKE---KKKLPK--------------------------KTNCLKRGPP
QPPDHKFRAPAFER--
-AK----PFRT-TA-QT-RANPHSSGEPVSICDSLSELLMTMQEELDQMNMEHRE---LLRQLMQTESQSVS
EDIEQELEQLVKKMESKGDQISLLKRHQDSVR--KLQEKVENSRI-NESSGIH---GNPKGSKNLKNS--PR
KCVSETSAFQR-NRSFQPVQ-VHSLQTKLRRDDIKWEQ
>XP_031204123.1/1-461 centrosomal protein CEP57L1 isoform X1 [Mastomys coucha]

---MR-------------------
--DAGPRAH-ALSAALSVLD
-----VWR-RW---
---------------------------------------LEEGGGKTMDSELSHSMV----GSYLKPP----
-E-------RT-YLPSFTQNEAV------QNYH--PG-----NS----PKMFNSPN-------NQ-------
-------ALVSALKTLQEKIRCLELERTQAEDNLNLLSREAAQYKKALEEESNERNLAHEELIKQKKDISIQ
LSSARSRCILLEKQLEYTKRMVLSVEREKTMILEQQ------------------------------------
-----------TQLQREKEQDQMKLHAKLEKLHILEKECLKLTVTQQTAEDKIKYLEEKLKEEEHQRRLFQD
KACELQTGFEISKILMSTVSNSKHCKE---KKKLPK--------------------------KTNCLKRGPP
QPPDHKFRAPAFER--
-AK----PFRT-TA-QT-RANPHSSGEPVSICDSLSELLMTMQEELDQMNMEHRE---LLRQLMQTESQSVS
EDIEQELEQLVKKMESKGDQISLLKRHQDSVR--KLQEKVENSRI-NESSGIH---GNPKGSKNLKNS--PR
KCVSETSAFQR-NRSFQPVQ-VHSLQTKLRRDDIKWEQ
>XP_034380762.1/1-455 centrosomal protein CEP57L1 isoform X1 [Arvicanthis niloticus]

---MP-------------------
---PRHL-SLPA---NQR

-----FWR-RW---LTGSAHL-----
-------------------T---------------------------MDSELSHSVV----GSYLNPP----
-E-------RM-CPPSFTQNEAF------QNYH--PG-----NS----LKMCNSLN-------NQ-------
-------ALVSALKTLQEKIRRLELERTQAEDNLNLLSREAAQYKKALEMESNERNLAHQELIKQKKDISIQ
LSSAQSRCILLEKQLEYTKRMVLNVEREKTVILEQQ------------------------------------
-----------AQLQREKEQDQMKLHAKLEKLHVLEKECLRLTATQQNAEDKIKYLEEKLKEEEHQRRLFQD
KACELQTGFEISKILMSTVSNSKHCKE---KKKLPK--------------------------KTNCLKREPR
QQMDHKFRAPTFKR--
-ET----PFRV-TA-QA-RANSHSSGEPVSVCDSLSELMMTMQDELDQMNMEHRE---LLRQMIQTESRSVS
EDLEHELDRLVQKMESKEDQISRLKKHQDSVR--KLQEKVENSRI-NESSGIH---GNPKRSKKLKTS--PQ
KCVSETSAFQR-DSSFQPVQ-VHSLQTKLRRDDIKWEQ
>XP_028624841.1/1-479 LOW QUALITY PROTEIN: centrosomal protein CEP57L1 [Grammomys surdaster]

---MP-------------------
---PRHL-SLPA---NQR
-----FWR-PW---LTGSVRL-----
-------------------T---------------------------MDSELSHSVV----GSYLNPP----
-E-------RT-CLPSFTQNEAL------QNYH--PG-----NS----PKMFNSPN-------NQ-------
-------ALVSALKTLQEKIRRLELERTQAEDNLNLLSREAAQYKKALEVETSERDLAHQELIKQKKDISIQ
LSSAQSRCILLEKQLEYTKRMVLNVEREKTMILEQQ------------------------------------
-----------AQLQKEKEQDQMKLHAKLEKLHVLEKECLRLTATQQTAEDKIKYLEEKLKEEEHQRRLFQD
KACELQTGFEISKILMSTVSNSKHCKE---KKKLPK--------------------------KTNCLKRSPP
QQADHKFRAPAFERG-------K-------------------------MVYPCG-L--DDLQKPVLATEAWC
LSK----PFRM-TA-QT-RANSHSSGEPVSICDSLSELLMTMQAELDQMNMEHRE---LLRQMMQTESHSIS
EDIEHELEQLVKKMDSKGDQISKLKKHRDSVR--KLQERVENSRI-NESSGIH---GNPKGSKNLKTS--PR
KCVSETSAFQR-DSGFQPVQ-VHSLQTKLRRDDITWEQ
>NP_001017448.2/1-446 centrosomal protein CEP57L1 [Rattus norvegicus]

---MP-------------------
---RDVY-RTRK---NEQ
-----I--K-----
-------------------T---------------------------MDAELPHSMI----GSYLNPP----
-E-------RM-YLPSFSQTEAS------QNCH--PG-----SS----PKMFNSPN-------NQ-------
-------ALVSALKTLQEKIRRLELERTQAEDNLNLLSREAAQYKKALEEETNERNLAHEELTRQKKDISIQ
LSSAQSRCILLEKQLEYTKRMVLNVEREKNMILEQQ------------------------------------
-----------AQLQREKEQDQMKLHAKLEKLDVLEKECLRLTTTQQTAEEKIKYLEEKLKEEEHQRRLFQD
RACELQTGLEISKILMSTVSSSKLCNE---RKKLPK--------------------------KTNCLKREPP
QHTDCRFRGPASER--E---
-RP----PFRM-TS-SA-RAEPHSSGEPFSICDNLSELLRTMQDELDQMNMEHRE---LLRQIAQTGSHSDS
EELEQELEHLARKMESKEDQISKLQKHQDRVR--KLQEKVENSRI-NESSGIH---GNPKRSKNLKTS--PR
KCLSETSAFQK-DRSFQPVK-VHSLPPRLRRDDVKWEQ
>XP_006256624.1/1-471 centrosomal protein CEP57L1 isoform X1 [Rattus norvegicus]
--MLPTRSVCDRKYNSQ-
---MP-------------------
---PRHS-SLPA---NQR
-----FWR-PW---LTGSVCL-----
-------------------T---------------------------MDAELPHSMI----GSYLNPP----
-E-------RM-YLPSFSQTEAS------QNCH--PG-----SS----PKMFNSPN-------NQ-------
-------ALVSALKTLQEKIRRLELERTQAEDNLNLLSREAAQYKKALEEETNERNLAHEELTRQKKDISIQ
LSSAQSRCILLEKQLEYTKRMVLNVEREKNMILEQQ------------------------------------
-----------AQLQREKEQDQMKLHAKLEKLDVLEKECLRLTTTQQTAEEKIKYLEEKLKEEEHQRRLFQD
RACELQTGLEISKILMSTVSSSKLCNE---RKKLPK--------------------------KTNCLKREPP
QHTDCRFRGPASER--E---
-RP----PFRM-TS-SA-RAEPHSSGEPFSICDNLSELLRTMQDELDQMNMEHRE---LLRQIAQTGSHSDS
EELEQELEHLARKMESKEDQISKLQKHQDRVR--KLQEKVENSRI-NESSGIH---GNPKRSKNLKTS--PR
KCLSETSAFQK-DRSFQPVK-VHSLPPRLRRDDVKWEQ
>XP_032744700.1/1-471 centrosomal protein CEP57L1 isoform X5 [Rattus rattus]
--MLPTRSVCDRKYNSQ-
---MP-------------------

--PRHS-SLPA---NQR
-----FWR-PW--LTGSVCL-----
-----------------T-------------------------MDAELSHSMI----GSYLNPP----
-E-------RM-YLPSFSQTEAS------QNCH--PG-----SS----PKMFSSPN-------NQ-------
-------ALVSALKTLQEKIRRLELERTQAEDSLNLLSREAAQYKKALEEETNERNLAHEELTRQKRDISIQ
LSSAQSRCSLLEKQLEYTKRMVLNVEREKNMILEQQ------------------------------------
-----------AQLQREKEQDQMKLHAKLEKLDVLEKECLRLTTTQQTAEEKIKYLEEKLKEEEHQRRLFQD
RACELQTGLEISKILMSTVSNPKLCKE---RKKLPK-------------------------KPNCLKRELL
QHTDGKFSGPASER---E---
-TP----PFRM-TS-SA-RAKPHSSGEPFSICDSLSELLRTMQDELDQMNMEHRE---LLRQIAQTGSHSDS
EELEQELEHLARKMESKEDQISKLQKHQDRVR--KLQEKVENSRI-NESSGIH---GNPKRSKNLKTS--PR
KCLSETSAFQK-DRSFQPVK-VHSLPPRLRRDDVKWEQ
>XP_051031529.1/1-482 centrosomal protein CEP57L1 [Phodopus roborovskii]
---MLPTPAVCDRNYNPH-
---RP-------------------
--PRRR-PLPA---NQR
-----SRRSSG--LTGSVHL-----
-----------------T-------------------------MDSELSHSVI----GSYLNPP----
-E-------RM-YLPSFIQNEPS------QNGH--PV-----NSEVIPPKMFNSPN-------NQ-------
-------ALVSALKTLQEKIHRLELERTQAEDNLNSLSREAAQYKKALENETNERNLAHQELIKQKKDISFQ
LSSAQSRCTLLEKQLEYTKRMVLNVEREKAMILEQQ------------------------------------
-----------AQLQREKEQDQMKLHAKLEKLDVLEKECFRLTTTQQTAEDKIRYLEEKLKEEEHQRKLFQD
RACELQTGLEISKILMSTVSHSKSCKE---KKKPLK-------------------------KTKCLKRDPP
QRTAPMPRALPLERE-------T--C
LHK----PLRT-TS-QG-KAGPPNSGEPVSMCDSLSELLMAMQEELDQMTVEHSE---LLKHMMQTGSHSVP
DDMEQELEQLVKKMESKGAQISKLKKHQDSVR--KLQEKIQDSRI-KGPSGVLREDGNPKGPKNMKNI--PR
KSLNDTNPFQK-NSSFRPIH-VHNLLGKLRRDDIKWEQ
>XP_007653002.1/1-478 centrosomal protein CEP57L1 isoform X3 [Cricetulus griseus]XP_027258021.1 centrosomal protein CEP57L1 isoform X3 [Cricetulus griseus]
---MLPTPCVCDRNYNPH-
---MP-------------------
--PQLL-SQPA---NQR
-----LWR-PG--LTGNVHLNIYSS
LVH--------------MWT-------------------------MNPELSHNVI----GSYLNPP----
-E-------RM-YFPSFIQNEAS------QNGY--PV-----DSEVIPPKMFNSPN-------NQ-------
-------ALVSALKTLQEKIHRLELERTQAEDNLNSLSREAAQYKKALESETNERDLAHRELIKQKKNISIQ
LSSAQSRCTLLEKQLEYTKRMVLNVEREKAMILEQQ------------------------------------
-----------AQLQREKEQDQMKLHAKLEKLDVLEKECFRLTTTQQTAEDKIKYLEEKLKEEEHHRKLFQD
KACELQTGLEISKILMSTVSHSKLCKE---KKKPLK-------------------------KTKCLKRDPP
QQMDP---
KFR----ALSL-QGGKG-KAGPPNSEEPISICDNLSELLMAMQEELDQMTVEHKE---LLKEMMQPGRY--S
DDIEHELEQLAKKIESKGDQISKLKKHQDSVR--KLQQKVQNSRI-NESSCVLQEHDNPKGPKNIKTS--SR
KGLNETNPLQK-NSSFRPVQ-VHNLQVKLRRDDIKWEQ
>XP_007652991.1/1-489 centrosomal protein CEP57L1 isoform X1 [Cricetulus griseus]XP_027258019.1 centrosomal protein CEP57L1 isoform X1 [Cricetulus griseus]
---MLPTPCVCDRNYNPH-
---MP-------------------
--PQLL-SQPA---NQR
-----LWR-PG--LTGNVHLNIYSS
LVH--------------MWT-------------------------MNPELSHNVI----GSYLNPP----
-E-------RM-YFPSFIQNEAS------QNGY--PV-----DSEVIPPKMFNSPN-------NQ-------
-------ALVSALKTLQEKIHRLELERTQAEDNLNSLSREAAQYKKALESETNERDLAHRELIKQKKNISIQ
LSSAQSRCTLLEKQLEYTKRMVLNVEREKAMILEQQ------------------------------------
-----------AQLQREKEQDQMKLHAKLEKLDVLEKECFRLTTTQQTAEDKIKYLEEKLKEEEHHRKLFQD
KACELQTGLEISKILMSTVSHSKLCKE---KKKPLK-------------------------KTKCLKRDPP
QQMDPKFRALSLQGG-------K--C
LYK----SLGT-TS-QG-KAGPPNSEEPISICDNLSELLMAMQEELDQMTVEHKE---LLKEMMQPGRY--S
DDIEHELEQLAKKIESKGDQISKLKKHQDSVR--KLQQKVQNSRI-NESSCVLQEHDNPKGPKNIKTS--SR
KGLNETNPLQK-NSSFRPVQ-VHNLQVKLRRDDIKWEQ

>XP_007653015.1/1-429 centrosomal protein CEP57L1 isoform X6 [Cricetulus griseus]XP_027258026.1 centrosomal protein CEP57L1 isoform X6 [Cricetulus griseus]
--MLPTPCVCDRNYNPH-
---MP-------------------
---PQLL-SQPA---NQR
-----LWR-PG---LTGNVHLNIYSS
LVH--------------MWT---------------------------MNPELSHNVI----GSYLNPP----
-E-------RM-YFPSFIQNEAS------QNGY--PV-----DSEVIPPKMFNSPN-------NQ-------
---DISIQ
LSSAQSRCTLLEKQLEYTKRMVLNVEREKAMILEQQ------------------------------------
-----------AQLQREKEQDQMKLHAKLEKLDVLEKECFRLTTTQQTAEDKIKYLEEKLKEEEHHRKLFQD
KACELQTGLEISKILMSTVSHSKLCKE---KKKPLK--------------------------KTKCLKRDPP
QQMDPKFRALSLQGG-------K--C
LYK----SLGT-TS-QG-KAGPPNSEEPISICDNLSELLMAMQEELDQMTVEHKE---LLKEMMQPGRY--S
DDIEHELEQLAKKIESKGDQISKLKKHQDSVR--KLQQKVQNSRI-NESSCVLQEHDNPKGPKNIKTS--SR
KGLNETNPLQK-NSSFRPVQ-VHNLQVKLRRDDIKWEQ
>XP_051020245.1/1-474 centrosomal protein CEP57L1 isoform X2 [Acomys russatus]
--MLPTRSVCDRNYNPQ-
---MP-------------------
---PRHFLSLPA---NQR
-----FWR-PG---LTGSICL-----
-------------------A---------------------------MDSELSHSVL----GSYLNPP----
-E-------RR-FAPPFPEKGAS------QNCH--PV-----NS----AKTFNSPN-------NQ-------
-------ALVSALKTLQEKIHRLDLERTQAEDNLNALSREAAEYKRALENETSERNLAHQELSKQKKDISIQ
LSSAQSRCTLLEKQLEYTKRMVLNVEREKAMILEQQ------------------------------------
-----------AQLQWEKEQDQMKLHAKLEKLDVLEKECFRLTATQQTAEDKIKYLEEKLKEEEHQRKLFQD
KACELQTGLEIRKILMSTISNSKHCKE---KKKPPK--------------------------KTKCSKTEPP
LQMDHKFRAPTFAR--EARC
PSK----PLGT-TP-RA-DAEPHNSGEPVSICDNLSELLLAMQDELTQMSVEHKE---LLKRMRQTGSQSVS
EDIEHELDQLVRKMESKGDQIAKLRKHQDIVC--KLQQRVQNSRI-NKSSGIHQED-----SKNMKNS--PR
KCVNEANLFQK-NSSSHPVQ-VHNVQLKLRRDDITWEQ
>XP_021504731.1/1-468 centrosomal protein CEP57L1 isoform X4 [Meriones unguiculatus]
--MLPTRFVCDRNYNSQ-
---MP-------------------
---PRRFLWLPA---NQR
-----FRR-PG---LTGNDHL-----
-------------------T---------------------------MNSDLAQSVV----GSYLNPP----
-K-------SM-YVPSFRQNEAF------QNCH--PV-----NS----SKTFNSPN-------NQ-------
-------ALVSALKTLQEKIHRLELERTQAEDNLNVLSREAAQYKKALENETEERNLAHQELIKQKKDISIQ
LSSAQSRCLLLEKQLEYTKRMVLNVEREKNMILEQQ------------------------------------
-----------TQLQREKEQDQMKLHAKLEKLDVLEKECFRLTRTQQTAEDKIKYLEEKLKEEEHQRKLFQD
KAAEQ--TNCIKREPP
QQTDHTSRAQTFERE-------K-------------------------MVEPSG-L--CNLQKPLLASEARS
LSK----SLRT-AS-LA-TAETRNSGERISICDNLSELLMAMQDELEQMSMEHKE---LLKRMRHSRSHVVS
EDIEQELEQLVKKMEKKEDQISKLIKHQDSVR--KLQRKVQNSRI-KESSGIQKGDSHPKGSKSMKKS--PR
KCWDESNPFLK-NS-FHSTQ-VHSLQATLRREDIKWEQ
>XP_060229488.1/1-497 centrosomal protein CEP57L1 isoform X1 [Meriones unguiculatus]
--MLPTRFVCDRNYNSQ-
---MP-------------------
---PRRFLWLPA---NQR
-----FRR-PG---LTGNDHL-----
-------------------T---------------------------MNSDLAQSVV----GSYLNPP----
-K-------SM-YVPSFRQNEAF------QNCH--PV-----NS----SKTFNSPN-------NQ-------
-------ALVSALKTLQEKIHRLELERTQAEDNLNVLSREAAQYKKALENETEERNLAHQELIKQKKDISIQ
LSSAQSRCLLLEKQLEYTKRMVLNVEREKNMILEQQ------------------------------------
-----------TQLQREKEQDQMKLHAKLEKLDVLEKECFRLTRTQQTAEDKIKYLEEKLKEEEHQRKLFQD
KAAELHTGLEVNKILMSTISNSKHCKK---KKKQRK--------------------------QTNCIKREPP
QQTDHTSRAQTFERE-------K-------------------------MVEPSG-L--CNLQKPLLASEARS

LSK----SLRT-AS-LA-TAETRNSGERISICDNLSELLMAMQDELEQMSMEHKE---LLKRMRHSRSHVVS
EDIEQELEQLVKKMEKKEDQISKLIKHQDSVR--KLQRKVQNSRI-KESSGIQKGDSHPKGSKSMKKS--PR
KCWDESNPFLK-NS-FHSTQ-VHSLQATLRREDIKWEQ
>XP_055472150.1/1-498 centrosomal protein CEP57L1 isoform X1 [Psammomys obesus]
--MLPTRFVCDRNYNSQ-
---MP-------------------
---PRRFLWLPA---NQR
-----FRR-PG---LTGNVHL-----
-------------------T---------------------------MNSDLAQSVV----GSYLNPP----
-E-------SM-YVPSFRQNEAF------QNCH--PV-----NS----SKTFNSPN-------NQ-------
-------ALVSALKTLQEKIHRLELERTQAEDNLNVLSKEAAQYKKALENETEERNLAHQELIKQKKDISIQ
LSSAQSRCLLLEKQLEYTKRMVLNVEREKNMILEQQ------------------------------------
-----------TQLQREKEQDQMKLHAKLEKLDVLEKECFRLTRTQQTAEDKIKYLEEKLKEEEHQRKLFQD
KASELQTGLEVNKMLMSTISNSKHCKK---KKKQRK--------------------------QTNCIKREPP
QQTGHTSRAQTFERE-------K-------------------------MVEPSR-L--CNLQKPLPVSEARS
LSK----PLRT-AS-LA-TAEARNSGEPISICDNLSELLVAMQDELDQMSMEHKE---LLKRMRHARSHVVS
EDIEQELEQLVKKMEKKEDQISKLMKHQDSVR--KLHRKVQNSRI-KESSGIQRGDGHPKGSKSMKKS--PR
KCWDETNPFLK-NSNFRPTQ-VHSLQAMLRREDIKWEQ
>XP_060229489.1/1-471 centrosomal protein CEP57L1 isoform X3 [Meriones unguiculatus]

--MHGDVYRVRKRH
----------------QIKT---------------------------MNSDLAQSVV----GSYLNPP----
-K-------SM-YVPSFRQNEAF------QNCH--PV-----NS----SKTFNSPN-------NQ-------
-------ALVSALKTLQEKIHRLELERTQAEDNLNVLSREAAQYKKALENETEERNLAHQELIKQKKDISIQ
LSSAQSRCLLLEKQLEYTKRMVLNVEREKNMILEQQ------------------------------------
-----------TQLQREKEQDQMKLHAKLEKLDVLEKECFRLTRTQQTAEDKIKYLEEKLKEEEHQRKLFQD
KAAELHTGLEVNKILMSTISNSKHCKK---KKKQRK--------------------------QTNCIKREPP
QQTDHTSRAQTFERE-------K-------------------------MVEPSG-L--CNLQKPLLASEARS
LSK----SLRT-AS-LA-TAETRNSGERISICDNLSELLMAMQDELEQMSMEHKE---LLKRMRHSRSHVVS
EDIEQELEQLVKKMEKKEDQISKLIKHQDSVR--KLQRKVQNSRI-KESSGIQKGDSHPKGSKSMKKS--PR
KCWDESNPFLK-NS-FHSTQ-VHSLQATLRREDIKWEQ
>XP_006982835.1/1-457 centrosomal protein CEP57L1 isoform X1 [Peromyscus maniculatus bairdii]XP_006982836.1 centrosomal protein CEP57L1 isoform X1 [Peromyscus maniculatus bairdii]XP_042117947.1 centrosomal protein CEP57L1 isoform X1 [Peromyscus maniculatus bairdii]

---MDSELSPSVI----GSYLNPP----
-E-------RM-YLPSFTQNEAS------QNCR--PV-----NSEVTSPKMFNSSN-------NQ-------
-------ALVSALKTLQEKIYRLELERTQAEDNLNSLSREAAQYKKALENETNERNLAHQELIKQKKDISIQ
LSSAQSRCVLLEKQLEYTKRMVLNVEREKAMILEQQ------------------------------------
-----------AQLQREKEEDQLKLHAKLAKLDVLEQECFRLTATQQTAEDKIKYLEEKLKEEEHQRKLFQD
KACELQTGLEISKILMSTVSHSQHYKE---KKKPPK--------------------------KTKCLKREPP
QQMDPRFRALFLERE-------K-------------------------MVHPHG-L--YYLQKPVLVPEPQS
LYK----ALRT-SS-QA-KVLPLDSGEPVSTCDNLSELLMAMQEELDQMIVEHRE---LQKQMLQDGSHSVS
DDAEHALEQLVRKMERKGDQISKLKKHQDSVR--KLQQRVQSSRI-NEPS-IHWEAGKPKGPK--KNS--PR
KCLNETNPFQK-NSSIHPVQ-VHNLQVKLRRDDIQWEQ
>XP_028746069.1/1-457 centrosomal protein CEP57L1 isoform X1 [Peromyscus leucopus]XP_028746070.1 centrosomal protein CEP57L1 isoform X1 [Peromyscus leucopus]XP_037056226.1 centrosomal protein CEP57L1 isoform X1 [Peromyscus leucopus]XP_037056227.1 centrosomal protein CEP57L1 isoform X1 [Peromyscus leucopus]XP_037056228.1 centrosomal protein CEP57L1 isoform X1 [Peromyscus leucopus]

--MDSELSQSVI----GSYLNPP----
-E-------RI-YLPSFTQNEAS------QNCH--PV-----NSEVTSPKMFNSPN-------NQ-------
-------ALVSALKTLQEKIYRLELERTQAEDNLNSLSREAAQYKKALENETNERNLAHQELIKQKKDISIQ
LSSAQSRCVLLEKQLEYTKRMVLNVEREKAMILEQQ------------------------------------
-----------AQLQREKEEDQLKLHAKLAKLDVLEQECFRLTTTQQTAEDKIKYLEEKLKEEEHQRKLFQD
KACELQTGLEISKILMSTVSHSQHCKE---KKKPPK-------------------------KTKCLKREPP
QQMDPRFRALFLERE-------K------------------------MVHPHG-L--YYLQKPVLVPEAQS
LYK----ALRT-SS-QA-KVLPLNSGDTVSTCDNLSELLMAMQEELDQMIVEHRE---LQKQMLQAGSHSLS
DDAEHALEQLVRKMESKGDQISKLKKHQDSVR--KLQQRVQSSRT-NEPS-IHWEAGKPKGPK--KNS--PR
KCLNETNPFQK-NSSVHPVQ-VHNLQVKLRRDDIQWEQ
>XP_052607478.1/1-483 centrosomal protein CEP57L1 isoform X1 [Peromyscus californicus insignis]

--MP------------------
---PQHFWSLWA---NQR
-----FWR-PG---LTGNVHP-----
------------------T---------------------------MDSELSQSVI----GSYLNPP----
-E-------RM-YLPSFTQNEVS------QNCH--PV-----NSEVTS-KMFNSPN-------NQ-------
-------ALVSALKTLQEKIYRLELERTQAEDSLNSLSREAAQYKKALENETNERNLAHQELIKQKKDISIQ
LSSAQSRCVLLEKQLEYTKRMVLNVEREKAMILEQQ------------------------------------
-----------AQLQREKEEDQLKLHAKLAKLDVLEKECFRLTTTQQTAEDKIKYLEEKLKEEEHQRKLFQD
KACELQTGLEISKILMSTVSHSQHCKE---KKKPPK-------------------------KTKCLKREPP
QQMDPRFRALFLERE-------K------------------------MMHPHG-L--YYLQKPALVPEPQT
LSK----ALRT-RS-QA-KALPLSSGEPVSICDNLSELLMAMQEELDQMIVEHKE---LRKQMLQAGSHLVS
DDAEHVLEQLVRKMESKGDQISKLKKHQDSVR--KLQQKVQSSRI-NDPS-IHWEAGNPKGPK--KNS--PR
KCLNETNPFQK-NSSVHPVQ-VHNLQVKLRRDDIQWEQ
>XP_059128475.1/1-484 centrosomal protein CEP57L1 isoform X2 [Peromyscus eremicus]

--MP------------------
---PQHFWSLWA---NQR
-----FWR-PG---LTGNVHL-----
------------------T---------------------------MDSELSQSVI----GSYLNPP----
-E-------RM-YLPSFTQNEAS------QNCH--PV-----NSEVTSPKMFNSPN-------NQ-------
-------ALVSALKTLQEKIYRLELERTQAEDSLNSLSREAAQYKKALENETNERNLAHQELIKQKKDISIQ
LSSAQSRCVLLEKQLEYTKRMVLNVEREKAMILEQQ------------------------------------
-----------AQLQREKEEDQLKLHAKLAKLDVLEQECFTLTTAQQTAEDKIKYLEEKLKEEEHQRKLFQD
KACELQTGLEISKILMSTVSHSQHCKE---KKKPPK-------------------------KTKCLKREPP
QQMDPRFRALFLEKE-------K------------------------MTHPHG-L--HYLQKPVLVPEPQS
LYK----ALRT-SS-QA-KARPLSSGEPIFICDDLSELLMAMQEELDQMIVEHKE---LRKQMLQAGSRSVS
DDAEHVLEQLVRKMESKGDQISKLKKHQDSVR--KLQQKVQSSRI-NDPS-IHWEAGNPKGPK--KNS--PR
KCLNETNPFQK-NSSVHPVQ-VHNLQVKLRRDDIQWEQ
>XP_036024477.1/1-498 centrosomal protein CEP57L1 isoform X1 [Onychomys torridus]
--MLPTPSVCDRNYNSQ-
--MP------------------
---PQHFWLLRA---SQR
-----FW---LTGNVHL-----
------------------T---------------------------MDSELSQSVI----GSYLNPP----
-E-------RM-YLPSFTQNEAS------QNCH--PV-----NSEVTSPKMFNSPN-------NQ-------
-------ALVSALKTLQEKIYRLELERTQAEDNLNSLSREAAQYKKALENETNERNLAHQELIKQKKDISIQ
LSSAQSRCVLLEKQLEYTKRMVLNVEREKAMILEQQ------------------------------------
-----------AQLQREKEEDQLKLHAKLAKLDVLEKECFRLTTTQQTAEEKIKYLEEKLKEEEHQRKLFQD
KACELQTGLEVSKILMSTVSHSQHCKE---KKKPPK-------------------------KTKCLKREPP
QQMDPRFRALFLERE-------K------------------------MMHPHG-L--YCLQKPVLAPEPQC
LYK----ALRT-AS-QS-KALPLNSGEPASICDNLSELLMAMQEELDQMIVEHRE---LREQMTEAGSRSVS
DDAEHVLEQLVRKMESKGDQISKLKKHQDSVR--KLQQKVQSSRL-NDPS-IHWEAGSPQGPKNVKHG--PR
KCWNEPNPFQK-NSSSHPVQ-VHNLQVKLRRDDIKWEQ
>XP_040584329.1/1-545 centrosomal protein CEP57L1 isoform X1 [Mesocricetus auratus]

--MLPTASVCDRNYNPH-
---MP-------------------
---PQHL-PLPA---NQR
-----FWR-PG---FTENVHL-----
-------------------T---------------------------MDPELSHSVI----GSYLNPP----
-E-------RK-YLPSFIQNEAS------QNGH--PV-----NSEVTPPKMFNSPN-------NR-------
-------ALVSALKTLQEKIHRLELERTQAEDNLNSLCREAAQYKKALENETNERNLAHQELIKQKKDISIQ
LSSAQSRCTLLEKQLEYTKRMVLNVEREKAMILEQQVGFWFMMLLLPQPLECCEYRREPQVPALDFQHIKDF
AQLSSALK---AQLQREKEQDQMKLHAKLEKLDVLEKECFRLTTTQQTAEDKIKYLEEKLKEEEHQRKLFQD
KACELQTGLEISRILMSTVSHSKLCKE---KKKPLK--------------------------KTKCLKREPP
QQMDPKFRALSLEGG-------K-------------------------MLHPYG-L--HYFQKPVLLPESRC
LYK----SLRT-TS-QD-NAEPLNSGESISICDNLSELLMAMQEELDQMTMQHRE---LLKQMMQTRSHSDS
DDIEHELEQLVKKMESKGDQISKLKKHQDSVR--KLQQKVQNSRI-NASSGFLRELGNPKGSKDTKNS--PR
KCLNETNPFQK-RVSFRPVQ-AHNLQVKLRRDDIKWEQ
>XP_038196717.1/1-434 centrosomal protein CEP57L1 isoform X4 [Arvicola amphibius]
--MLPTPAACDRNYNPQ-
---MP-------------------
---PQHFRSLSA---NQR
-----FRR-PG---LTGNVHL-----
-------------------T---------------------------MDSELSPSLI----GSYLNPP----
-E-------RM-YPPSFIKNETS------QIGH--PV-----NSEVTPPKMINSPN-------NQ-------
-------ALVSALKTLQEKIHRIELERTQAEDNLNSLSREAAQYKKALENETNERNLAHQELIKQKKDISIQ
LSSARSRCILLEKQLEYTKRMVLNVEREKAIILEQ-------------------------------------
----
---QLQTGLEISRLLMSTVSQSQPCKG---KKKPPK--------------------------KTKSLKREPP
QQMDPKCRARSLERE-------K-------------------------TISAYG-L--RSFQRPAPVAEPQC
RYQ----PLRT-AS-WG-KAVPPDSGEPVPVCDSLSDLLMAMQEELDQMAAEHRE---LLR---HTGSHSVS
DDMERQLEQLVKKMESKEGQISKLKRHQDSVR--KLQQKVQNSRI-NESSGIRREDGNPKGPKNVKNS--PR
KCWDEANPFQK-HSTCHPAQ-VHNLQVKLRKDDIKWEQ
>XP_038196713.1/1-499 centrosomal protein CEP57L1 isoform X1 [Arvicola amphibius]
--MLPTPAACDRNYNPQ-
---MP-------------------
---PQHFRSLSA---NQR
-----FRR-PG---LTGNVHL-----
-------------------T---------------------------MDSELSPSLI----GSYLNPP----
-E-------RM-YPPSFIKNETS------QIGH--PV-----NSEVTPPKMINSPN-------NQ-------
-------ALVSALKTLQEKIHRIELERTQAEDNLNSLSREAAQYKKALENETNERNLAHQELIKQKKDISIQ
LSSARSRCILLEKQLEYTKRMVLNVEREKAIILEQQ------------------------------------
-----------AQLQREKEQDQMKLYAKLEKLNVLEKECFRLTATQQTAEDKINYLEEKLKEEEHQRKLFQD
RACELQTGLEISRLLMSTVSQSQPCKG---KKKPPK--------------------------KTKSLKREPP
QQMDPKCRARSLERE-------K-------------------------TISAYG-L--RSFQRPAPVAEPQC
RYQ----PLRT-AS-WG-KAVPPDSGEPVPVCDSLSDLLMAMQEELDQMAAEHRE---LLR---HTGSHSVS
DDMERQLEQLVKKMESKEGQISKLKRHQDSVR--KLQQKVQNSRI-NESSGIRREDGNPKGPKNVKNS--PR
KCWDEANPFQK-HSTCHPAQ-VHNLQVKLRKDDIKWEQ
>XP_057618081.1/1-499 centrosomal protein CEP57L1 isoform X1 [Chionomys nivalis]
--MLPTPAVCDRNYNPQ-
---MP-------------------
---PQHFRSLSA---NQR
-----FRR-PG---LTGNVHL-----
-------------------T---------------------------MDSELSHSII----GSYLNPP----
-E-------RM-CPPLFIKNETS------QIGH--PV-----NSEVIPPKMINSPN-------NQ-------
-------ALVSALKTLQEKIHRIELERTQAEDNLNSLSREAAQYKKALENETNERNLAHEELIKQKKDISIQ
LSSAQSRCILLEKQLEYTKRMVLNVEREKAMILEQQ------------------------------------
-----------AQLQREKEQDQMKLYAKLEKLNVLEKECFRLTATQQAAEDKINYLEEKLKEEEHQRKLFQD
RACELQTGLEISRLLMPTVSQSQHCKR---KKKPPK--------------------------KTKCLKREPP
QQMDPKCRAQSLERV-------K-------------------------MIGAYG-L--RSLQRPAPVTELQC
RYK----PLRT-TS-QV-KAVAPNPGEPVPICDSLSDLLMAMQEELAQMTVEHRE---LMR---QTGSHSVS
DDIEHELEQLVKKMESKEDQISKLKKHQDSVR--KLQQKVQNSRI-NEPSGIHQEDGNPKGLKNVKNS--PR

KCWDETNPFRK-HSTCHPVH-VHNVQVKLRKDDIRWEQ
>XP_048281237.1/1-523 centrosomal protein CEP57L1 isoform X1 [Myodes glareolus]
--MLPTPAGCDRSYNPQ-
---IP-------------------
---PQHFRSLQA---NQR
-----FRR-PG---LTGNVLLVFCWH
VCQKRASAPRAVGGSWPSQT---------------------------MDSELSHSVI----GSYLNPP----
-E-------KM-YPPSFIKNETS------QIGH--PV-----NLEVTTPKMINSPN-------NQ-------
-------ALVSALKTLQEKIHRIELERTQAEDNLNSLSREAAQYKKALENETNERNLAHQELIKQKKDISIQ
LSSAQSRCILLEKQLEYTKRMVLNVEREKAMILEQQ------------------------------------
-----------AQLQREKEQDQMKLYAKLEKLNVLEKECFRLTATQQTAEDKINYLEEKLKEEEHQRKLFQD
RACELQTGLEISRLLMSTVSQSQHSKE---KKKPPK--------------------------KTKCLKREPP
QQMDPKCRAQSLERE-------K-------------------------VMHAYG-L--HCLQRPAPVTEPQC
RYK----PLRT-AS-RG-KAVPSNSGEPIPTCDSLSDLLMAMQEELDQMTAEHRE---LLR---QTGSHSVS
EDMEHELQQLVKKMESKEDQISKLKKHQDSVR--KLQQKVQKSRI-NESSGLHQEDGHPKGPKNMKNS--PR
KCWNETNPFQK-HSTCHPAQ-VHNLQVKLRKDDIMWEQ
>XP_027712488.1/1-464 centrosomal protein CEP57L1 isoform X1 [Vombatus ursinus]XP_027712497.1 centrosomal protein CEP57L1 isoform X1 [Vombatus ursinus]

---MYAEFKHSAI----GSYRKPP----
-D-------KI-TNPSFAYHEAA-----PQDNLPKKLEP------------VSIPN-------SH-------
-------ALLLALKTLQDKIHRLELERTQAEDNLNTLSQEAALYKKALQNENNERDLAHQELIKQRKDISAQ
LSAAQIRCSILEKQLELTKKMVLNAEQEKNFTLEQQ------------------------------------
--------------QWEKGQDQMKLNEKLEKLEVLEKECVRLTTTQKTAEEKIKHLEQKLIEEEHQRKLIED
SAAQLQSGLEINRILMSSALPSQQNK----KKKSAK--------------------------EKKSSKRGL-
QQTHSKFGA--------IG---K------SANES-HFVNASV-QTILNMMKHGNSP---SLQAYPEAPERRA
ISK------RTVRC----RPTSSYSSKSTSIGDGLTDLVMAMQDELDQMSIEHQE---LLKQVQESPRLSVN
EEIEYELECSARKMEVKGEQICKLKKYQDHVN--KLQQKSQNSK--SEAFSIEPEDENHKRTKNVPVMVVTR
KGTNKTKSPQKNCSCQMPQKCFQKIQMTLRKDDIKWEQ
>XP_020863874.1/1-466 centrosomal protein CEP57L1 isoform X1 [Phascolarctos cinereus]XP_020863875.1 centrosomal protein CEP57L1 isoform X1 [Phascolarctos cinereus]

---MYAEFKHSMI----GSYRKPP----
-D-------KI-TNPSVAYREAA-----PQDNLPKKLEP------------LSIPN-------SH-------
-------ALLLALKTLQDKIHRLELERTQAEDNLNTLSQEAALYKKALQNENNERNLAHQELIKQRKDISAQ
LSAAQIRCSVLEKQLELTKKVILNAEQEKNFALEQQ------------------------------------
------------QLQWEKGRDQIKLNEKLEKLEVLEKECVRLTTTQKTAEEKIKHLEQKLIEEEHQRKLIQD
SAAQLQTGLEINRILMSSVLPSQQTK----KKKSAK--------------------------GKKSSKKGL-
QQTHSKFGA--------IG---K------SANES-HFVNASV-QTILNMTKHGNSP---SLQACPEAPERRA
ISK------RTVRC----RPTSSYSSKSTSIGDGLTDLVMAMQDELDQMSIEHQE---LLKRVQESQRLSVN
AEIEYELECLARNMEVKGEQICKLKKYQDHVN--KLQQKSQNSK--SEAFSNEPEDENYKRTKNVPVMVVTR
KGTNKTKPPQENCSCHMPQNRFQKIQMTLRKDDIKWEQ
>XP_043855651.1/1-465 centrosomal protein CEP57L1 isoform X1 [Dromiciops gliroides]XP_043855652.1 centrosomal protein CEP57L1 isoform X1 [Dromiciops gliroides]

---MYAELKHSMI----GSYREPP----
-D-------KI-TDPSFAYCEAA-----TQNHLPKKSQP------------LSTPN-------SH-------
-------ALLLALKTLQDKIHRLELERTQAEDNLNTLSQEAALYKKALQNENNERDLVHQELIKQRKDISAQ
LSAAQTRCSILEKQLELTKKMVLNAEQEKNLTVEQQ------------------------------------

-------------LQWEKGQDQMKLNAKLEKLEVLEKECVRLTSTQKTAEEKIKHLEQKLIEEEHQRKLIQD
RAAQLQTGLEINRILMSSVLPSQQTK----KKKAAK-------------------------GKKCSKRGL-
QQTHSKFGA--------VG---K------SPSES-HFVNASM-QSILNMMKHGNSP---SLQGCPEATERRA
ISK------RTVRC----RPTSSYSSKSTSVGDGLTDLVMAMQDELDQMSIEHQE---LLKQVQGSPRLSVN
EEIEYELECLARKMEMKGEQICKLKKYQDHVN--KLQQKSQNSE--SEEFSIQPENENHKGTKTVPVMVVTR
KGTNKTKAPPKNCSCQMPQKCFQKIRMTLRKDDVKWEQ
>XP_031820307.1/1-466 centrosomal protein CEP57L1 isoform X1 [Sarcophilus harrisii]

--MYAEFKHSMI----GSYRKPP----
-D-------KI-ANPSFAYCGAA-----PQDQLPKKLQP------------LSTPN-------SH-------
-------ALLLALKTLQDKIYQLELERTQAEDNLNTLSQEAALYKKALQNENNERDLAHQELIKQRKDISAQ
LSAAQTRCSILEKQLELAKKMVFNAEQEKIFALEQQ------------------------------------
------------QLQWEKGQDQMKLNAKLEKLEVLEKECVRLTTTQKTAEEKIKHLEQKLTEEEHHRKLIQD
SAAQLQTGLEINRILISSVLPSQQTK----KKKSTK-------------------------GKKSSKRGF-
QQTHSKLGA--------IG---K------SANES-HVVNSNV-KTILNMMKHGNSP---SLQGCPEANERRA
ISK------RTLRC----RPMSSYSSKSTSTGDGLTDLVMAMQDELDQMNIEHQE---LLKQIHGSPTLSAN
EEIEYELECLARKMEMKKEQICKLKKYQDHVS--KLQPKSPNSK--SEAFDIQSEEENHKGTKKVPVVVVTR
KSTNKTKSPQKNCKCQMPEKCFQKIQMTLRKDDVKWEQ
>XP_036624683.1/1-437 centrosomal protein CEP57L1 isoform X1 [Trichosurus vulpecula]XP_036624684.1 centrosomal protein CEP57L1 isoform X1 [Trichosurus vulpecula]

--MCAEFKHSMI----GSYRKPP----
-D-------KI-TNLSFAYCEAA-----PQDNLPKKLQH------------LSTPN-------SH-------
-------ALLLALKTLQDKIHLLELERTQAEDNLNTLSQEATLYKKALQNENNERDLAHQELIKQRKDISAQ
LSAAQTRCSILEKQLELTTKMVLNAEQEKNFALEQQ------------------------------------
------------QLQWEKGQDQMKLNAKLEKLEVLEKECVRLTTTQKTAEEKIKHLEQKLIEEEHQRKLIQD
SAAQLQTGLEINRILMSSVLPSQQTK----KKKSAK-------------------------EKKSSKREL-
-QTHSKFGA--------IG---K---ACPEVPERRA
ISK------RTVRC----RPISSYPNKSTPVGDGLTDLVMAMQDELDQMSIEHQE---LLKQVQESPRLSVN
EEIECELECLARKMEMKGEQICKLKKYQDHVN--KLQQKSQNSK--SEAFSIEPEDENHKGKKNVPVMVVTR
KGRNKTKLPLENCSCQMPQKCFQKIRMTLRKDDVKWEQ
>XP_044530270.1/1-458 centrosomal protein CEP57L1 isoform X1 [Gracilinanus agilis]

--MFADMKHSMI----GSYRKPP----
-D-------KI-TDPSFIYSGEA-----PWKHLSKKL-P------------FSTPN-------TP-------
-------ALLLALKTLQDKIQQLVIERAQAEENLSALSREAEQYKKALQDENNERDLAHQELIKQRKDISTQ
LLAAQTRCSILEKQLALTKKMVLNAEQEKNVALEQQ------------------------------------
------------Q---EKDQNQMKLNAKLEKLEVLEKECFRLTTTQKTAEEKIKYLEEKLIEEEHQRKLVQD
RAAQLQTGLEINRILMSSVLPSQLIK----RKKSTK-------------------------KIKCSKRGL-
QQTHSKFGA--------IG---K------SANVS-HSVNTSV-QTLLNLMKHGSSP---SLQGCPEATERRA
IGK------RTVRS----RPTSSYSS----VGDGLSDLLMAMQDELDQMSTEHEE---LLKQVQESPVLPVN
EDLECELSCLARKMEIKGEQIHKLKKYQDHVN--KLQQKSQNEK--SEASSIQPEDEDQKETKNVPVTVVTK
KGTNKTKPPQKNFSCQMPHKCVQKIQMTLRKDDVKWEQ
>XP_007484469.1/1-459 centrosomal protein CEP57L1 isoform X1 [Monodelphis domestica]XP_056674628.1 centrosomal protein CEP57L1 isoform X1 [Monodelphis domestica]

---M-----
--MYADLKHSVI----GSYRKPP----
-D-------KI-TDPSFIYCGEA-----PWKHPSNKL-P-----------FSTPN-------TP-------
-------ALLLALKTLQDKIQQLVIERAQAEENLSALSQEAEQYKKALQDENNERDLAHQELIKQRKDISTQ
LLAAQTRCSILEKQLALTKKMVLNAEQEKNVALEQQ-----------------------------------
------------Q---EKDQNQMKLNAKLEKLEVLEKECFRLTTTQKTAEEKIKYLEEKLLEEEHQRKLVQD
RAAQLQTGLEINRILMSSVLPSQLIK----RKKSAK-------------------------KIKCSKRGL-
QQTHSKFGA--------IG---K------SASVS-HSANTSV-QTLLNLMKHGSSP---SLQGCPEATERRA
ISK------RTVRC----RPTSSYSS----VGDGLSDLLMAMQDELDQMITEHEE---LLKQVQESPVLLVN
EDLECELGCLARKMEMKGEQIHKLKKYQEHVN--KLQQKSQNEK--SEASSIQPEDEDQKETKNVPVTVVTK
KGTNKTKPPQKNFSCQMPHKCFQKIQMTLRKDDVKWEQ
>XP_035152352.2/1-610 centrosomal protein CEP57L1 isoform X1 [Callithrix jacchus]
MSRAILERRAGPWSWRGGGARGSKPALRPAPSVGISPEGGSRE-----GRRGPHPQPLPLLRPAQTRSRPGP
SA------------------FFPAQAE------------------ATRRGKAIGKSPAL--WQATSR-----
-------------PQVLRARQPTAPCCYACVTANE----------AHASLEALSLSP-------------
-------G-AVSVGGALESVLEKG-----------------------------------K-EKI-----
--MDSELMHSIV----GSYRKPP----
-E-------RV-FVPSFTKNESS------QNCH--PA-----NLEVTSSKIIHSPN-------SQ-------
-------ALILALKTLQEKIHRLELERTQAEDNLNTLSKEAAQYKKALENETNERNLAHQELIKQKKDISVQ
LSSAQSRCTLLEKQLEYTKRMVLNVEREKNMILEQQ-----------------------------------
-----------AQLQREKEQDEMKLYAKLEKLDVLEKECFRLTATQKTAEDKIKYLEERLKEEEHQRKLFQD
KASELQTGLEISKIIMSSVSNLKHSKE---KKKSSK-------------------------KTKCVKRGSP
RQVCSKFGALPFVAE-------K------------------------MRQHRG-P--HILQKSSNETEPIC
LPK----PSRT-TS-WC-KAIPTDSEKSISICDNLSELLMAMQDELDQMSVEHQE---LLKQMKETESHSVF
NDIECELECLVKKMEIKGTQISKLKKHQDSVR--KLQQKVQNSKM-SKASGIQQEGSNPKGSRNIKNS--PR
KCLTDTNLFQK-NSSFHPIP-VHNLQVKLRRDDIMWEQ
>MBZ3879316.1/1-700 Centrosomal protein CEP57L1 [Sciurus carolinensis]
MS---------PFDISFQKIHSAAP-----------PNTKNSEMDFKLSLKDNLFYHMDSRNTFQLKSSPPP
PSQRGPSSSGNPRAPSRAKELAEPQGQRLRARPFVQLHRWDRPRGREQGGEMGSLAPSPSPFSARHK-----
-------------PGAAPAPAPSSPPKQTRPAKAK----------PLGKAPPSSRLPSD---PKS---AER
GRRPLPAT-THAQPRVACVRLQRPSVCAGKRFSFPGVRSSWTGVGEGKVRNRGLLGTWALAAATNWI-----
--MNSELIHSIV----GSYLKPP----
-E-------EV-FAPSFTQDES------SQNCH--TV-----NLEVTSPKMLHSPN-------NQ-------
-------ALILALKTLQEKIHHLELERTQAEDNLNILSREAAQYKKALENETNERNLAHQELIKQKKDISLQ
LSSAQSRCTLLEKQLEYTKRMVLNVEREKNMILEQQ-----------------------------------
-----------AQLQREKEQDQLRLHAKLEKLDVLEKECFRLTTTQKTAEDKIKHLEEKLKEEEHHRKLFQD
KASELQTGLEISKILMSSVSKSKLSKG---KKKSSK-------------------------KTKCFKRGPP
QQIYAKLRELPFEAE-------K------SAA---CPVNASM-HSLLKMMNHCS-Q--HSLQKP-EMTEPRC
LYR----PTRK-TS-QC-KAVSQDSEKSISICDNLSELLMAMQDELDHMSMEHEE---LLKQMKKTKSRSVC
DDIECELEHLVKKMEIKGEQISKLRKHQDNVR--KLQQKVQNSKM-SDTSGIQREDSNLKGSKNIKNS--PR
KCLNETNPFQK--NNFQPVQ-VRNLQMKLRRDDIMWEQ
>ETE69941.1/1-413 Centrosomal protein CEP57L1, partial [Ophiophagus hannah]

--------LVAALKTLQEKIHRLEIERIQAENDLNCLSREAAQYRRALQQESNEKNRVHQELMEEKKDVSVP
LRAAQSRCSLLEKQLDYMRKMVLKAELEKNLVLEQQ-----------------------------------
-----------THLQKEDDQNQMELCAKLEKLEVLEKQCWTLISTQKTAEEKIKHLEQKLLEEQHQRKLIQD
KAAQLQTGLEMNRILLSSLSPEKEVKKKSRKKKVLK-------------------------NNSTVKRT--
-QPSFQGGMLPFVAG-------K------SASSS-HSVVANV-QSVLHMMKYRS-A--CMTSQCPEGTEK--
-----RTSRWTGIC----KPVS-CSTTS-SATETLSDLLLGMQDELGQMSFEHQE---LSKQIEESQNQDIR
EDLERELDCLVKQMEIKGEQISKLKKHQDHV--YKLKQRAQNLK--RNTAYSTSRPNELKGSKETVIS--P-
----SISSTNKTTNSLRLLKSAQKLQSVLKKDDIVWES
>XP_015676799.2/1-457 centrosomal protein CEP57L1 isoform X1 [Protobothrops mucrosquamatus]XP_015676800.1 centrosomal protein CEP57L1 isoform X1 [Protobothrops mucrosquamatus]

--ME----
-------------------S---------------------------VDSESKHSYI----GSFLQPP----
-D-------KI--KSAFADLKPK------------SV------ISC-------PPN-------NQ-------
-------ALVAALKTLQEKICRLEIERIQAENDLNCLSREAAQYRKALQQESNEKNIVHPELMEERKDVSAQ
LRAAQSRCSLLEKQLDYMRKMVLKAELEKKLVLEQQ------------------------------------
-----------THLQKEDDQNQMELCAKLEKLEILEKQCWTLISTQKTAEEKIKHLEQKLLEEQHQRKLIQD
KAAQLQTGVEMNRILLSSLSPEKEVKKKSRKKKVIK--------------------------NNSSMKRT--
-QSSFQGGMLPFVAG-------K------SASSS-HSVVANV-QSVLHMMKYRS-P--CMTSQCPEGTEK--
-----RTSRWPGIC----KPAS-CSTTS-SATESLSDLLLGMQDELGQMSFEHQE---LSKQIEESQNQDIR
EDLERELDCLVKQMEIKGEQISKLKKHQDHV--YKLKQRAQKLK--REPACSASRPNELKGSKETVIP--P-
----SISSTNKTTNSLRLLKSAQKIQSVLKKDDIVWES
>XP_039207096.1/1-458 centrosomal protein CEP57L1 isoform X1 [Crotalus tigris]XP_039207097.1 centrosomal protein CEP57L1 isoform X1 [Crotalus tigris]XP_039207099.1 centrosomal protein CEP57L1 isoform X2 [Crotalus tigris]XP_039207100.1 centrosomal protein CEP57L1 isoform X2 [Crotalus tigris]

---MME----
-------------------S---------------------------VDSESKHSYI----GSFLQPP----
-D-------KM--KSAFADLKPK------------GV------ISC-------PPN-------NQ-------
-------ALVAALKTLQEKICRLEMERIQAENDLNCLSREAAQYRKAFQQESNEKNIVHLELMEERKDVSAQ
LRAAQSRCSLLEKQLDYMRKMVLKAELEKKLVLEQQ------------------------------------
-----------THLQKEDDQNQMELCAKLEKLEVLEKQCWTLISTQKTAEEKIKHLEQKLLEEQHQRKLIQD
KAAQLQTGVEMNRILLSSLPPEKEVKKKSRKKKVVK--------------------------NNSSMKRT--
-QSSFQGGMLPFVAG-------K------SASSS-HSVVANV-QSVLHMMKYRS-P--CMTSQCPEGTEK--
-----RTSRWPGIC----KPAS-CSTTS-SATETLSDLLLGMQDELGQMSFEHQE---LSKQIEESQNQDIR
EDLERELDCLVKQMEIKGEQISKLKKHQDHV--YKLKQRAQKLK--REPACSASRPNELKGSKETVIP--P-
----SISSTIKTTNSLRLLKSAQKIQSVLKKDDIVWES
>XP_007434081.1/1-476 centrosomal protein CEP57L1 isoform X1 [Python bivittatus]

---MALARGE--------------

---GELCPRSSIME----
-------------------S---------------------------VDTESKHSYI----GSFLQPP----
-D-------KI--KSAFADLRPK------------KV------NSC-------PPN-------NQ-------
-------ALVAALKTLQEKIHRLEVERTQAEDDLNCLSREAAQYRKALQHESNEKNVIHQELMEEKKDVSAQ
LRAARSRCSLLEKQLDYMRKMVLKAELEKNLVLEQQ------------------------------------
-----------THLQKEDDQNQMELCAKLEKLEVLEKQCWRLISTQKTAEEKMKQLEQKLQEEQHQRKLIQD
KAAQLQTGLEMNRILLSSLSPEKDVKKKSRKKKVIK--------------------------NNSGMRRTYG
SQPCLQGGMLPFVAG-------K------SASSS-HSVVANV-QSVLHMMKYRS-P--CMTSQCPEGTEK--
-----RTSRWTGVC----KPMI-CSTSS-SATETLSDLLLGMQDELGQMSFEHQE---LSKQIEESQNQDIR
EDLERELDCLVKQMEIKGEQITKLKKHQDHV--YKLKQRAQKLK--RKPTSSASRPNELKGSKETAIP--P-
----STSSTNKTTSSLQLLKSAQKLQSVLKKDDIVWES
>XP_026522435.1/1-472 centrosomal protein CEP57L1 isoform X2 [Notechis scutatus]

---MASVGRE--------------

---VECCPRSYMME----
-------------------S---------------------------VDNESKHSYI----GSFLQPP----
-D-------KL--KS-FADLRPK------------RT------INC-------PPN-------NQ-------
-------ALVAALKTLQEKIHRLEIERIQAENDLNCLSREAAQYRRALEQESNEKNIVHQELMEEKKDVSVP
LRAAQSRCSLLEKQLDYMRKMMLKAELEKKLVLEQQ------------------------------------
-----------THLQKEDDQNQMELCAKLEKLEVLEKQCWTLISTQKTAEEKIKHLEQKLLEEQHQRKLIQD
KAAQLQTGLEMNRILLSSLSPEKEVKKKSRKKKVLK--------------------------NNSTVKRT--
-QPSFQGGMLPFVAG-------K------SASSS-HSVVANV-QSVLHMMKYRS-A--CMTSQCPEGTEK--
-----RTSRWTGIC----KPVS-CSTTS-SATETLSDLLLYMQDELGQMNFEHQE---LSKQIEESQNQDIR

EDLERELDCLVKQMEIKGEQISKLKKHQDHV--YKLKQRAQNLK--RDTAYSTSRPNELKGSKETIIS--P-
----SISSTNKTTHSLRLLKSAQKLQSVLKKDDIVWES
>XP_026582419.1/1-472 centrosomal protein CEP57L1 isoform X2 [Pseudonaja textilis]

---MASVGRE--------------

---AECCPRSYMME----
------------------S---------------------------LDNESKHSYI----GSFLQPP----
-D-------KL--KS-FADLRPK------------ST------INC-------PPN-------NQ-------
-------ALVAALKTLQEKIHRLEIERIQAENDLNCLSREAAQYRRALEQESNEKNIVHQELMEEKKDVSMP
LRAAQSRCSLLEKQLDYMRKMMLKAELEKKLVLEQQ------------------------------------
-----------THLQKKDDQNQMELCAKLEKLEVLEKQCWTLISTQKTAEEKIKHLEQKLLEEQHQRKLIQD
KAAQLQTGLEMNRILLSSLSPEKEVKKKSRKKKVLK-------------------------NNSTVKRT--
-QPSFQGGMLPFVAG-------K------SASSS-HSVVANV-QSVLHMMKYRS-A--CMTSQCPEGPEK--
-----RTSRWTGIC----KPVS-RSTTS-SATETLSDLLLCMQDELGQMNFEHQE---LSKQIEESQNQDIR
EDLERELDCLVKQMEIKGQQISKLKKHQDHV--YKLKQRAQNLK--RDTAYSTSRPNELKGSKETIIS--P-
----SISSTNKTTNSLQLLKSAQKLQSVLKKDDIVWES
>KAG8123959.1/1-496 hypothetical protein E2320_019559 [Naja naja]

---MASVGGEEMTGSI--------
--GRPT-NLTER
HLWLPQGR--RDIECCPRSYMME----
------------------S---------------------------VDNESKHSYI----GSFLQPP----
-D-------KL--KSAFADLRPK------------SV------INC-------PPN-------NQ-------
-------ALVAALKTLQEKIHRLEMERIQAENDLNCLSREAAQYRRALQQESNEKNIVHQELMEEKKDVSVP
LRAAQSRCSLLEKQLDYMRKMMLKAELEKKLVLEQQ------------------------------------
-----------THLQKEDDQNQVELCAKLEKLEVLEKQCWTLISTQKTAEEKIKHLEQKLLEEQHQRKLIQD
KAAQLQTGLEMNRILLSSLSP--EVKKKSRKKKVLK-------------------------NNSSVKRT--
-QPSFQGGMLPFVAG-------K------SASSS-HSVVANV-QSVLHMMKYRS-A--CMASQCPEGTEK--
-----RTSRWTGIC----KPVS-CSTTP-CATETLSDLLLGMQDELGQMSFEHQE---LSKQIEESQNQDIR
EDLERELDCLVKQMEIKGEQISKLKKHQDHV--YKLKQRAQNLK--RETAYSTSRPNELKGSKETVIS--S-
----SISSTNKTTNSLRLLKSAQKLQSVLKKDDIVWES
>XP_026522433.1/1-497 centrosomal protein CEP57L1 isoform X1 [Notechis scutatus]

---MASVGREEMTGSI--------
--GRPT-NLTER
HLWLPQGR--KDIECCPRSYMME----
------------------S---------------------------VDNESKHSYI----GSFLQPP----
-D-------KL--KS-FADLRPK------------RT------INC-------PPN-------NQ-------
-------ALVAALKTLQEKIHRLEIERIQAENDLNCLSREAAQYRRALEQESNEKNIVHQELMEEKKDVSVP
LRAAQSRCSLLEKQLDYMRKMMLKAELEKKLVLEQQ------------------------------------
-----------THLQKEDDQNQMELCAKLEKLEVLEKQCWTLISTQKTAEEKIKHLEQKLLEEQHQRKLIQD
KAAQLQTGLEMNRILLSSLSPEKEVKKKSRKKKVLK-------------------------NNSTVKRT--
-QPSFQGGMLPFVAG-------K------SASSS-HSVVANV-QSVLHMMKYRS-A--CMTSQCPEGTEK--
-----RTSRWTGIC----KPVS-CSTTS-SATETLSDLLLYMQDELGQMNFEHQE---LSKQIEESQNQDIR
EDLERELDCLVKQMEIKGEQISKLKKHQDHV--YKLKQRAQNLK--RDTAYSTSRPNELKGSKETIIS--P-
----SISSTNKTTHSLRLLKSAQKLQSVLKKDDIVWES
>XP_026582418.1/1-497 centrosomal protein CEP57L1 isoform X1 [Pseudonaja textilis]

---MASVGREEMTGSI--------
--GRPT-NLTER
HLWLPQGR--RDTECCPRSYMME----
------------------S---------------------------LDNESKHSYI----GSFLQPP----
-D-------KL--KS-FADLRPK------------ST------INC-------PPN-------NQ-------
-------ALVAALKTLQEKIHRLEIERIQAENDLNCLSREAAQYRRALEQESNEKNIVHQELMEEKKDVSMP
LRAAQSRCSLLEKQLDYMRKMMLKAELEKKLVLEQQ------------------------------------
-----------THLQKKDDQNQMELCAKLEKLEVLEKQCWTLISTQKTAEEKIKHLEQKLLEEQHQRKLIQD
KAAQLQTGLEMNRILLSSLSPEKEVKKKSRKKKVLK-------------------------NNSTVKRT--

-QPSFQGGMLPFVAG-------K------SASSS-HSVVANV-QSVLHMMKYRS-A--CMTSQCPEGPEK--
-----RTSRWTGIC----KPVS-RSTTS-SATETLSDLLLCMQDELGQMNFEHQE---LSKQIEESQNQDIR
EDLERELDCLVKQMEIKGQQISKLKKHQDHV--YKLKQRAQNLK--RDTAYSTSRPNELKGSKETIIS--P-
----SISSTNKTTNSLQLLKSAQKLQSVLKKDDIVWES
>XP_032072750.1/1-473 centrosomal protein CEP57L1 isoform X1 [Thamnophis elegans]

---MASVRGE--------------

---VECCSRSYTME----
-------------------S---------------------------VDSESKHSYI----GSFLQPP----
-E-------RL--KSAFADLRPK------------SV------INF-------PPN-------NQ-------
-------ALVAALKTLQEKIHRLEIERIQAENDLNCLSREAAQYRRALQQESNEKHIVHQELMEEKKDVSVP
LRAAQSRCSLLEKQLDYMRKMVLKAEMEKKLVLEQQ------------------------------------
-----------THLQKEDDQNQMELCAKLEKLEVLEKQCWTLISTQKTAEEKIKHLEQKFLEEQHQRKLIQD
KAAQLQTGLEMNRILLSSLSPEKEVKKKSRKKKVIK--------------------------NNSTVKRT--
-QPSFQGGMLPFVAG-------K------SASSS-HSVVANV-QSVLHMMKYRS-P--CMTSQCPEGTEK--
-----RTSRWTGLS----KPVP-CSTTS-CATEMLSDLLLGMQDELGQMSFEHQE---LSKQIKESQNQDIR
EDLERELDCLVKQMEIKGEQISKLKKHQDHV--YKLKQRAQKLK--RETAYSTSNPNELKGSKETVIA--P-
----SICSTNKTTNSLRLLKSAQKLQSVLKKDDIVWES
>XP_013929138.1/1-473 PREDICTED: centrosomal protein CEP57L1 isoform X2 [Thamnophis sirtalis]

---MASVRGE--------------

---VECCSRSYTME----
-------------------S---------------------------VDSESKHSYI----GSFLQPP----
-E-------RL--KSAFADLRPK------------SV------INF-------PPN-------NQ-------
-------ALVAALKTLQEKIHRLEIERIQAENDLNCLSREAAQYRRALQQESNEKHIVHQELMEEKKDVSVP
LRAAQSRCSLLEKQLDYMRKMVLKAEMEKKLVLEQQ------------------------------------
-----------THLQKEDDQNQMELCAKLEKLEVLEKQCWTLISTQKTAEEKIKHLEQKLLEEQHQRKLIQD
KAAQLQTGLEMNRILLSSLSPEKEVKKKSRKKKVIK--------------------------NNSTVKRT--
-QPSFQGGMLPFVAG-------K------SASSS-HSVVANV-QSVLHMMKYRS-P--CMTSQCPEGTEK--
-----RTSRWTGLS----KPVP-CSTTS-CATEMLSDLLLGMQDELGQMSFEHQE---LSKQIKESQNQDIR
EDLERELDCLVKQMEIKGEQISKLKKHQDHV--YKLKQRAQKLK--RETAYSTSNPNELKGSKETVIA--P-
----SICSTNKTTNSLRLLKSAQKLQSVLKKDDIVWES
>XP_013929137.1/1-480 PREDICTED: centrosomal protein CEP57L1 isoform X1 [Thamnophis sirtalis]

---MEQITGLFLFSFV--------

--LVECCSRSYTME----
-------------------S---------------------------VDSESKHSYI----GSFLQPP----
-E-------RL--KSAFADLRPK------------SV------INF-------PPN-------NQ-------
-------ALVAALKTLQEKIHRLEIERIQAENDLNCLSREAAQYRRALQQESNEKHIVHQELMEEKKDVSVP
LRAAQSRCSLLEKQLDYMRKMVLKAEMEKKLVLEQQ------------------------------------
-----------THLQKEDDQNQMELCAKLEKLEVLEKQCWTLISTQKTAEEKIKHLEQKLLEEQHQRKLIQD
KAAQLQTGLEMNRILLSSLSPEKEVKKKSRKKKVIK--------------------------NNSTVKRT--
-QPSFQGGMLPFVAG-------K------SASSS-HSVVANV-QSVLHMMKYRS-P--CMTSQCPEGTEK--
-----RTSRWTGLS----KPVP-CSTTS-CATEMLSDLLLGMQDELGQMSFEHQE---LSKQIKESQNQDIR
EDLERELDCLVKQMEIKGEQISKLKKHQDHV--YKLKQRAQKLK--RETAYSTSNPNELKGSKETVIA--P-
----SICSTNKTTNSLRLLKSAQKLQSVLKKDDIVWES
>XP_058030175.1/1-473 centrosomal protein CEP57L1 [Ahaetulla prasina]

---MASIRGE--------------

---VECCSRSYTVE----
-------------------S---------------------------VDSESKHSYI----GSFLQPP----
-E-------RL--KSTFADLRPK------------SV------INF-------PPN-------NQ-------
-------ALVAALKTLQEKIHRLEIERIQAENDLNCLSKEAAQYRRALQQESNEKNIVHQELMEEKKDVSVP
LRAAQSRCSLLEKQLDYMRKMMLKAELEKKLVLEQQ------------------------------------

-----------THLRKEDDQNQMELCAKLEKLEVLEKQCWTLISTQKTAEEKIKHLEQKLLEEQHQRKLIQD
KAAQLQTGLEMNRILLSSLSPEKEVKKKSRKKKVIK--------------------------NNSSVKRT--
-EPSFQGGMLPFVAG-------K------SASSS-HSVVANV-QSVLHMMKHRN-P--CMTSQCPEGTER--
-----RTSKWTGIC----KPVS-CSTTS-CTTETLSDLLLGMQDELGQMSFEHQE---LSKQIEESQNQDIR
EDLERELDYLVKQMEIKGEQISQLKKHQDHV--YKLKQRAQKLK--KEPAYSTSRPNELKRSKETVIT--S-
----SICSTNKTTNSLRLLKSAQKLQSVLKKDDIVWES
>XP_034265023.1/1-474 centrosomal protein CEP57L1 isoform X1 [Pantherophis guttatus]

---MASIRRE--------------

---VECCPRSYTME----
------------------S----------------------------VDSESKHSYI----GSFLQPP----
-E-------RL--KSAFADLRPK------------SV------INF-------PPN-------NQ-------
-------ALVAALKTLQEKIHRLEIERIQAENDLNCLSREAAQYRRALQQESNKKNIVHQELMEEKKDVSVP
LRAAQSRCSLLEKQLDYMRKMVLKAELEKKLVLEQQ------------------------------------
-----------THLQKEDDQNQMELCAKLEKLEVLEKQCWTLISTQKTAEEKIKHLEQKLLEEQHQRKLIQD
KAAQLQTGLEMNRILLSSLSPEKEVKKKSRKKKVIK--------------------------NNSSLKRT--
-QPSFQGGMLPFVAG-------K------SASSS-HSVVANV-QTVLHMMKHRNSP--CMTSQCPQGTEK--
-----RTSRWTGIC----KPVS-YSTTS-CATETLSDLLLGMQDELGQMSFEHQE---LSKQIEESQNQDIR
EDLERELDCLVKQMEIKGEQISKLKKHQDHV--SKLKQRAQKLK--KEPAYSTSRPNELKGSKETVIS--P-
----SICSTNKTTNSLRLLKSAQKLQSVLKKDDIVWES
>XP_034965165.1/1-465 centrosomal protein CEP57L1 [Zootoca vivipara]XP_034965166.1 centrosomal protein CEP57L1 [Zootoca vivipara]

--ME----
------------------S----------------------------IDSESKHSYI----GSFIQPP----
-D-------NI--YSAFANIRSK------------EA------AAIAKD--TPAPN-------NQ-------
-------ALVAALKTLQEKIRRLELERSQAEENLNCLSREAAQYKKAFQHDSNEEDITHQGMMQEKKDVSMQ
LMAAQSRCSLLEKQLDYMRKMMINAELEKKLVLEQQ------------------------------------
-----------TQLQKEKDQNQLELCAKLDKLEILEKECWRLTSTQKTAEEKIRHLEQKLLEEQHQRKLIQD
KAAQLQTGLEMNRILMCSLSPREEANKKRRKKKAVK--------------------------KP-GLRKACS
SQPYLPTGTLPFVAG-------K------SASSS-HSVVANV-QSVLHMMKYRS-Q--SAASPRPEGGEK--
-----RT----GMS----RLSS-CSTSS-SATEHLSDLLLIMQDELGQMNFEHQE---LLKQIQETQNQEVR
EDLERELDCLVKQMEAKGEQISKLRKHQENV--CRLKQKAKKLK--QKAANSASRPNGIKGAKETAAA--SR
ASVNSPCPVNKSTRSLQLLKSAQKLQSVLKKDDIVWEP
>XP_053237614.1/1-466 centrosomal protein CEP57L1 isoform X1 [Podarcis raffonei]XP_053237615.1 centrosomal protein CEP57L1 isoform X1 [Podarcis raffonei]

--ME----
------------------S----------------------------LDSESKNSYV----GSFLQPP----
-G-------NI--YSAFATLRSK------------EA------TAMAKD--TPAPN-------NQ-------
-------ALVAALKTLQEKIRRLELERSQAEENLNCLSREAAQYKKAFQHDSNEKDITHQGMMQEKKDVSMQ
LRAAQSRCSLLEKQLDYMRKMMVNAELEKKLVLEQQ------------------------------------
-----------MQLQKEKDQNQLELCAKLDKLEILEKECWRLTSTQKTAEDKIRHLEQKLLEEQHQRKLIQD
KAAQLQTGLEMNRILMCSLSPQKEANKKSRKKKAIK--------------------------KSSGLRKACG
SQSYLPAGTLPFVAG-------K------SASSS-HSVVANV-QSVLHMMKYRS-Q--SAASQRPEGGEK--
-----RT----GMS----RPSS-CSTSS-SATEHLSDLLLIMQDELGQMNFEHQE---LLKQIQETQNQEVR
EDLERELDCLVKQMESKGEQISKLRKHQENV--YRLRQKAKKLK--QKAANSASRPNGLKGAKETSAT--SR
ASVNSACPMNKSTSSLQLLKSAQKLQSVLKKDDIVWEP
>XP_028578961.1/1-466 centrosomal protein CEP57L1 isoform X1 [Podarcis muralis]XP_028578962.1 centrosomal protein CEP57L1 isoform X1 [Podarcis muralis]XP_028578964.1 centrosomal protein CEP57L1 isoform X1 [Podarcis muralis]

--ME----
-------------------S---------------------------LDSESKNSYI----GSFLQPP----
-G-------NI--YSAFANVRSK------------EA------TAMAKD--TPAPN-------NQ-------
-------ALVAALKTLQEKIRRLELERSQAEENLNCLSREAAQYKKAFQHDANEKDITHQGMMEEKKDVSMQ
LRAAQSRCSLLEKQLDYMRKMMVNAELEKKLVLEQQ------------------------------------
-----------MQLQKEKDQNQLELCAKLDKLEILEKECWRLTSTQKTAEDKIRHLEQKLLEEQHQRKLIQD
KAAQLQTGLEMNRILMCSLSPQKEANKKSRKKKAVK-------------------------KPSGLRKACG
SQPYLPAGTLPFVAG-------K------SASSS-HSVVANV-QSVLHMMKYRS-Q--SAASQRPEGGEK--
-----RT----GTS----RPSS-CSTSS-SATEHLSDLLLIMQDELGQMNFEHQE---LLKQIQETQNQEVR
EDLERELDCLVKQMESKGEQLSKLRKHQENV--YRLRQKARKLK--QKAANSASRPNRLKGAKETAAS--SR
ASVNSACPMNKSTSSLQLLKSAQKFQSVLKKDDIVWEP
>XP_032999461.1/1-466 centrosomal protein CEP57L1 [Lacerta agilis]XP_032999462.1 centrosomal protein CEP57L1 [Lacerta agilis]

--ME----
-------------------S---------------------------IDSESKHSYI----GSFLQPP----
-D-------NI--YSAFANIRSK------------EA------AAIQKD--TPAPN-------NQ-------
-------ALVAALKTLQEKIRRLELERSQAEENLNCLSREAAQYKKAFQHDSNEKDITHQGMMQEKKDVSMQ
LSAAQSRCSLLEKQLDYMRKMMVNAELEKKLVLEQQ------------------------------------
-----------MQLQKEKDQNQLELCAKLDKLEILEEQCWRLTSTQKTAEEKIRHLEHKLLEEQHQRKLIQD
KAAQLQTGLEMNRILMCSLSPQKEANKKSRKKKAIK-------------------------KPSGLRKACC
SQPYLPAGTLPFVAG-------K------SASSS-HSVVANV-QSVLHMMKYRS-Q--SAASQRPEGAEK--
-----RT----GMS----RPSS-CSTSS-SATEHLSDLLLIMQDELGQMNFEHQE---LLKQIQETQNQEVR
EDLERELDGLVKQMEAKGEQISKLRKHQENV--YRLRQKAKKLK--QKAANSASRPNGLKGAKETAAA--SR
ASVNSTCPVNKSTSSLQLLKSVQKLQSVLKKDDIVWEP
>XP_061479553.1/1-469 centrosomal protein CEP57L1 isoform X1 [Rhineura floridana]XP_061479554.1 centrosomal protein CEP57L1 isoform X1 [Rhineura floridana]

--ME----
-------------------S---------------------------VDTESKHSYI----GSFLQPP----
-D-------KM--HSAFADFRSK------------EA------TAITKD--TIAPN-------NQ-------
-------ALAAALKTLQEKIHRLELERSQAEDDLNCLSREAAQYKKVLQHESDEKDITRQELMQERKDVSMQ
LRAAQSRCSLLEKQLDYMRKMMVSAELEKKLVLEQQ------------------------------------
-----------TQLEKEKDQNQLELCIKLDKLEILEKECWRLIRTQKTAEEKIKHLEQKLQEEQHQRKLIQD
KAAQLQTGLEMNRILMSSLSPQKDAKKKSRKKKAIK-------------------------KNSDLRKTCG
SQPHLPAGTLPFVAG-------K------SASSS-HSVVANV-QSVLHMMKYRS-P--CAPSQHPERAEK--
-----RTSRWSGMS----GRAS-CST-S-SATENLSDLLLVMQDELGQMSFEHQE---LLKQIQETQNHEVW
EDLERELDCLVKQMEIKGEQISKLRKHQENV--YRLRQRAQKLK--RKAANAASRPDELKGTTETPVP--PR
VSVNSACSVNKSISSLRLLKNAQKLQLVLKKDDIVWES
>XP_054836807.1/1-469 centrosomal protein CEP57L1 isoform X2 [Eublepharis macularius]

--ME----
-------------------S---------------------------VDIESKHSYI----GSYIQPP----
-Q-------KI--PTPFGDLWPK------------KT------AAVATD--TPAPN-------SQ-------
-------ALVSALKSLQEKIHRLELERSQAEDDLNCLSREAAQYKKTLLHESNEKDIANQELMQERKDVSVQ
LSAAQYRCSLLEKQLDYMRKMVTSAEMEKKLVLEKQ------------------------------------
-----------TQLQKEKDQNQVELCAKLDKLEILEKECWRLTGTQKAAEEKIKHLEVKLQEEQHHRKLIQD
KAAQLQTGLEMNRILMSSLSPQKEAKKKSRKKKAVK-------------------------KNTSLWKEYS
SQQYLPTEALPFVAG-------K------STSSS-HSIVANV-QSILHMMKSRG-P--HVTPQCPEGAEK--
-----RVSRWTDVC----RSGSSCST-S-SAAENLSDLLLLMQDELGQMSFEHQE---LLKQIQETKNHEVR
EDLERELDCLVKQMEIKGEQITKLRKHQENV--YKLKQKAQKLK--RKAANV-SKPDDLKVTKEVTVP--PT
SSVYGTCSVNKSTNSLQLLRSAQKIQSVLKKDDILWEP

>XP_054836798.1/1-483 centrosomal protein CEP57L1 isoform X1 [Eublepharis macularius]

--MFMMGGR-----------

---CYDLWNPTT---
----------------S-----------------------VDIESKHSYI----GSYIQPP----
-Q-------KI--PTPFGDLWPK-----------KT------AAVATD--TPAPN-------SQ-------
-------ALVSALKSLQEKIHRLELERSQAEDDLNCLSREAAQYKKTLLHESNEKDIANQELMQERKDVSVQ
LSAAQYRCSLLEKQLDYMRKMVTSAEMEKKLVLEKQ------------------------
----------TQLQKEKDQNQVELCAKLDKLEILEKECWRLTGTQKAAEEKIKHLEVKLQEEQHHRKLIQD
KAAQLQTGLEMNRILMSSLSPQKEAKKKSRKKKAVK------------------------KNTSLWKEYS
SQQYLPTEALPFVAG-------K------STSSS-HSIVANV-QSILHMMKSRG-P--HVTPQCPEGAEK--
-----RVSRWTDVC----RSGSSCST-S-SAAENLSDLLLMQDELGQMSFEHQE---LLKQIQETKNHEVR
EDLERELDCLVKQMEIKGEQITKLKRHQENV--YKLKQKAQKLK--RKAANV-SKPDDLKVTKEVTVP--PT
SSVYGTCSVNKSTNSLQLLRSAQKIQSVLKKDDILWEP
>XP_060093951.1/1-470 centrosomal protein CEP57L1 isoform X2 [Heteronotia binoei]XP_060093960.1 centrosomal protein CEP57L1 isoform X2 [Heteronotia binoei]XP_060093968.1 centrosomal protein CEP57L1 isoform X2 [Heteronotia binoei]

---ME----
----------------S-----------------------LDIESKHSYI----GSFIQPP----
-H-------KI--SIPFADLWPK-----------KT------TAVTAD--TPAPN-------SQ-------
-------ALLSALKSLQEKIHRLELERSQAEDDLNCLSREAAQYKKTLQHESNEKDIVHQKLMQDRKDVSVQ
LSAAQSRCSLLEKQLDYMRKMVTSAELEKKLVVEQQ------------------------
----------SQLQKEKDQNQAEICAKLDKLEILEKECWRLTGTQKAAEEKIKHLEARLQEEQHHRKLIQD
KAAQLQTGLEMNRILMTSLSPQKEAKKKSGKKKAVK------------------------KNSSLKKECS
SQPHLAAGALPFVAG-------K------SASSS-HSVAANV-QSVLHMMKSRG-P--HLIPQGPEGAEK--
-----RISRWTGVC----RSGSFCST-S-SATDNLSDLLLIMQDELGQMSFEHQE---LLKQIQETKNHEVR
EDLERELECLVKQMEIKGEQITKLKRHQESV--YKLKQKAQKLK--RKAANVTSKPDDCKGTKEVTVP--PR
SSVHGTCSVNKSTNSLQLLRSAQKIQSVLKKDDILWEP
>XP_060093943.1/1-479 centrosomal protein CEP57L1 isoform X1 [Heteronotia binoei]

---MTLENS--
LGE-------------HCS-----------------------LDIESKHSYI----GSFIQPP----
-H-------KI--SIPFADLWPK-----------KT------TAVTAD--TPAPN-------SQ-------
-------ALLSALKSLQEKIHRLELERSQAEDDLNCLSREAAQYKKTLQHESNEKDIVHQKLMQDRKDVSVQ
LSAAQSRCSLLEKQLDYMRKMVTSAELEKKLVVEQQ------------------------
----------SQLQKEKDQNQAEICAKLDKLEILEKECWRLTGTQKAAEEKIKHLEARLQEEQHHRKLIQD
KAAQLQTGLEMNRILMTSLSPQKEAKKKSGKKKAVK------------------------KNSSLKKECS
SQPHLAAGALPFVAG-------K------SASSS-HSVAANV-QSVLHMMKSRG-P--HLIPQGPEGAEK--
-----RISRWTGVC----RSGSFCST-S-SATDNLSDLLLIMQDELGQMSFEHQE---LLKQIQETKNHEVR
EDLERELECLVKQMEIKGEQITKLKRHQESV--YKLKQKAQKLK--RKAANVTSKPDDCKGTKEVTVP--PR
SSVHGTCSVNKSTNSLQLLRSAQKIQSVLKKDDILWEP
>XP_056712367.1/1-471 centrosomal protein CEP57L1 [Euleptes europaea]

---ME----
----------------S-----------------------VDTESKHSYI----GSFIQPP----
-H-------KI--PVPFADLWPK-----------TT------AAFAAD--TPAPN-------SQ-------
-------ALVSALKSLQEKIHRLELERSHAEHDLNCLSREAAQYKKTLQCESNEKDTAHQELMQERKDVSVQ
LSAAQSCCSLLEKQLDYMRKMVVSAELEKKLVLEQQ------------------------
----------IQLQKEKDQNQVEICAKLDKLEILEQECWRLTGTQKAAEGKIKHLEAKLQEEQHHRKLIQD
KAAQLQTGLEMNRILMSSLSPQNEAKKKSGKKKAVK------------------------KNHSLRKECS

SQPYLPAGGLPFVAG-------K------SASSS-HSVVANV-QSILHLVKSRG-A--DVIPQGSEGAEK--
-----RTSRWTGVC----RSGSSYSTSS-SATDNLSDLLLVMQDELGQMSFEHQE---LLKQIQETKSQEVR
EDLERELECLVKQMEIKAEQITKLKRHQDSV--YKLKQKARKLK--RKAANFTSKPDDFKGTKEAAVP--PR
GIVHGTCSVNKTTNSLQLLKKAQKIQSVLKQDDILWEP
>XP_048349085.1/1-471 centrosomal protein CEP57L1 isoform X2 [Sphaerodactylus townsendi]XP_048349096.1 centrosomal protein CEP57L1 isoform X2 [Sphaerodactylus townsendi]XP_048349105.1 centrosomal protein CEP57L1 isoform X2 [Sphaerodactylus townsendi]XP_048349113.1 centrosomal protein CEP57L1 isoform X2 [Sphaerodactylus townsendi]

--ME----
-------------------S---------------------------VDTESNYSYI----GSFVQPP----
-H-------KI--SIPFAELWPK------------KT------TAVAAD--TPAPN-------SQ-------
-------ALVSALKSLQEKIHRLELERSQAEADLNCLSREAALYKKTLQHESNEEETAHQELMQERKDVSVQ
LSAAQSRCSLLEKQLDYMRKMMIDAELEKKLVLEQQ------------------------------------
-----------THLQKEKDQNQIEICAKLDKLEILEQECRRLTGTQKGAEEKIKRLEAKLQEEQHHRKLIQD
KAAQLQTGLEMNRILMHSLSPQKEEKKKSQKKKAVK-------------------------KNPSLRKECS
SQPYLPAGALPFVAG-------K------SASSS-HSVAANV-QSVLHMMKSRG-R--YVIPQDQEAAEK--
-----NPSRWTGVC----RSGSAYSASS-SATDNLTELLLLMQDELGQMSFEHQD---LLKQIQETKSYEVR
EDLERELECLVKQMEIKGEQITKLKRHQDSI--YKLKQKAQKLK--RKPANVTSKPDYCRETQEATDP--PR
SSVYGTCSVNKTTNSLQLLKSAQKIQSVLKKDDILWEP
>KAJ6660260.1/1-470 hypothetical protein lerEdw1_017960 [Lerista edwardsae]

--ME----
-------------------S---------------------------LDMESKNSYV----GSFLQPP----
-D-------KI--PSALADFWSK------------KA------TAATID--TSGPN-------NQ-------
-------ALVSALKTLQEKIHRLELERSQAEDDLNCLSREAAQYKKALQHESKKEDVNHLELMQERKDVSMQ
LGAAQSRCSLLEKQLDYMRKMVVSAELEKRLVLEKQ------------------------------------
-----------TQLQKEKDQSQVELCAKLDKLEILEKECWRLTDTQKAAEEKIKHLEAKLIEEQHQRKLIQD
KAAQLQTGLEMNRILVSSLSPQKDAKKKSRKKKVIK-------------------------KTSVSRKVCD
TQPSLQPGTLPFVAG-------K------SASSS-HSVVANV-QSVLHMMKYRS-P--QVTLHHSEAAEQ--
-----RTSRWTDVC----RPGS-CSTSS-SISENLSDILLALQDELGQMSFEHQE---LLKQIQETQNPDAQ
EDLERELDCLVKQMEVKGEQISKLKKHQENV--YKLRQKAQKLK--RKAANAASRPAKFEEAKETTVL--PR
GSVHGTCSVNKSTNSLQLLKTAQRLQLVLKKDDIVWEP
>XP_020656986.1/1-470 centrosomal protein CEP57L1 [Pogona vitticeps]

--ME----
-------------------S---------------------------VDTDSKHSYI----GSFLQPP----
-D-------NI--CSAFADLRSK------------KR------PAIPTD--APVPN-------NK-------
-------ALVAALKTLQEKIHRLELERSQAEDNLNSLSSEAAQYKKALQHESNAKDATHQELLQQRKDVSMQ
LETAQSRCSLLEKQLDYMRKMVVSAELEKKLVLEQQ------------------------------------
-----------TQLQKEKEQNHVELCVKLDKLEILEKECERLTITQKTAEEKIKCLEHKLQEEQHQRKLIQD
KAAQLQTGLEMNRILLSSLSPPKDTKKKSKKKKAGK-------------------------KNPGLIKTYD
SQPCLQTGKLPFVAG-------M------SASSS-HSVVANV-QSVLHMMKNRG-L--CATAQCPEGADK--
-----RISRRTGAC----RPVS-SSTSS-SATENLSDLLLIMQNELGQMNLEHQE---LLKQIEETENQDVQ
EDLERELDCLVKQMEIKGEQISKLRKHQEHV--YKLTQKAQKLK--RKVAKTTSRSEELKGIKEPTIS--PK
SSVHSICSMSRSKNSLELLKSAQKLQSVLKKDDIVWES
>XP_060609127.1/1-475 centrosomal protein CEP57L1 isoform X1 [Anolis sagrei ordinatus]XP_060609128.1 centrosomal protein CEP57L1 isoform X1 [Anolis sagrei ordinatus]

--MEVSNT
TSA--------------DQS--------------------------FDSASKHSYI----GSFLQPP----

-V-------NI--RS---GFELK------------KK------T-IPTD--IPAPN-------NQ-------
-------ALVTALRTLQEKIHRLELERSQAEDDLNALSREAAQYKRALQHESNEKDKTHGEMMQERKGVSMQ
LGAAQTRCSLLEKQLEYMRRMVVSAELEKRQVLEQQ------------------------------------
-----------IQLQKEKDQDQVELCAKLDKLEILEKECWRLTSTQKTAEEKIKYLEQKLQEEQHQRKLIQD
KAAQLQTGLEMNRILLSSLSPQKDTKKKSRKKKGVK--------------------------NNTDLTKTHG
SHPSQQTGVLPFVAG-------K------SASSS-HSVVANV-QSVLHMMKYRS-P--CVTSHYPEQSEK--
-----RPSRRAALG---RPSS-CNTSS-SATDSLSDLLLVMQDELGQMSFEHQE---LLKQIQETQEQDMR
EDLERELDCLVKQMEIKGEQISKLKKHQEHV--FKLRRSAQKLK--RKVANAASNSEELTGTKETAVV--SR
LGARSSCPVNRSTSSLQLLKCAQKLQSVLKKDDIVWEP
>XP_008119127.1/1-495 PREDICTED: centrosomal protein CEP57L1 isoform X1 [Anolis carolinensis]

---MGVPPSLG---NV--------
--QRSI-NIG--
---LL---QGEWGTGSSVME----
-------------------S---------------------------FDSASKHSYI----GSFLQPP----
-V-------NI--RS---GLELK------------KK------T-IPKD--IPAPN-------NQ-------
-------ALVTALRTLQEKIHRLELERSQAEDDLNALSREAAQYKRAMQHESNEKDKTHGKVMQERKDVSIQ
LGAAQTRCSLLEKQLDYMRRMVVSAELEKKQVLEQQ------------------------------------
-----------IQLQKEKDQDQVELCAKLDKLEILEKECWRLTSTQKTAEEKIKHLEQKLQEEQHQRKLIQD
KAAQLQTGLEMNRILLSSISPQKDTKKKCKKKKVVK--------------------------NNPDSPKTHG
SQPSPQTGVLPFVAG-------K------SASSS-HSVVANV-QSVLHMMKYRS-P--CVTSHYSEQAEK--
-----RVSRRAVLG---RPSS-CTTSS-SATDSLSDLLLVMQDELGQMSFEHQE---LLKQIQETQDQDMR
EDLERELDCLVKHMEIKGEQISKLKKHQEHV--FKMRRSAQKLK--RKVANAASNSDELKGTKKTAVT--LR
LGARSSCSVNRNTSSLQLLKCAQKLQSVLKKDDIVWEP
>XP_016853400.1/1-490 PREDICTED: centrosomal protein CEP57L1 isoform X3 [Anolis carolinensis]

---MAF------------------
--FI-ESG--
---LLGHT---NTGEWGTGSSVME----
-------------------S---------------------------FDSASKHSYI----GSFLQPP----
-V-------NI--RS---GLELK------------KK------T-IPKD--IPAPN-------NQ-------
-------ALVTALRTLQEKIHRLELERSQAEDDLNALSREAAQYKRAMQHESNEKDKTHGKVMQERKDVSIQ
LGAAQTRCSLLEKQLDYMRRMVVSAELEKKQVLEQQ------------------------------------
-----------IQLQKEKDQDQVELCAKLDKLEILEKECWRLTSTQKTAEEKIKHLEQKLQEEQHQRKLIQD
KAAQLQTGLEMNRILLSSISPQKDTKKKCKKKKVVK--------------------------NNPDSPKTHG
SQPSPQTGVLPFVAG-------K------SASSS-HSVVANV-QSVLHMMKYRS-P--CVTSHYSEQAEK--
-----RVSRRAVLG---RPSS-CTTSS-SATDSLSDLLLVMQDELGQMSFEHQE---LLKQIQETQDQDMR
EDLERELDCLVKHMEIKGEQISKLKKHQEHV--FKMRRSAQKLK--RKVANAASNSDELKGTKKTAVT--LR
LGARSSCSVNRNTSSLQLLKCAQKLQSVLKKDDIVWEP
>XP_008119133.1/1-490 PREDICTED: centrosomal protein CEP57L1 isoform X2 [Anolis carolinensis]XP_008119141.1 PREDICTED: centrosomal protein CEP57L1 isoform X2 [Anolis carolinensis]XP_008119146.1 PREDICTED: centrosomal protein CEP57L1 isoform X2 [Anolis carolinensis]

---MADLPRYRFDCN---------

-------R---HYGEWGTGSSVME----
-------------------S---------------------------FDSASKHSYI----GSFLQPP----
-V-------NI--RS---GLELK------------KK------T-IPKD--IPAPN-------NQ-------
-------ALVTALRTLQEKIHRLELERSQAEDDLNALSREAAQYKRAMQHESNEKDKTHGKVMQERKDVSIQ
LGAAQTRCSLLEKQLDYMRRMVVSAELEKKQVLEQQ------------------------------------
-----------IQLQKEKDQDQVELCAKLDKLEILEKECWRLTSTQKTAEEKIKHLEQKLQEEQHQRKLIQD
KAAQLQTGLEMNRILLSSISPQKDTKKKCKKKKVVK--------------------------NNPDSPKTHG
SQPSPQTGVLPFVAG-------K------SASSS-HSVVANV-QSVLHMMKYRS-P--CVTSHYSEQAEK--
-----RVSRRAVLG---RPSS-CTTSS-SATDSLSDLLLVMQDELGQMSFEHQE---LLKQIQETQDQDMR
EDLERELDCLVKHMEIKGEQISKLKKHQEHV--FKMRRSAQKLK--RKVANAASNSDELKGTKKTAVT--LR
LGARSSCSVNRNTSSLQLLKCAQKLQSVLKKDDIVWEP

>XP_042301699.1/1-467 centrosomal protein CEP57L1 isoform X1 [Sceloporus undulatus]XP_042301700.1 centrosomal protein CEP57L1 isoform X1 [Sceloporus undulatus]

--ME----
-------------------S---------------------------FDNESKHSYI----GSFLLPP----
-G-------KI--GS---DLGPK------------KK------TAIPAD--TPAPN-------NQ-------
-------ALVAALRTLQEKIHRLELERSQAEDDLNALSKEAAQYKRTLQHESNEKDISHEEMTQERKDVSMQ
LGAAQTRCSLLEKQLDYMRRMVISAQLEKKQVLEQQ------------------------------------
-----------IQLQKEKDQDQVELCAKLEKLEILEKECWRLTSTQKTAEEKIKCLEQKLQEEQHQRKLIQD
KAAQLQTGLEMNRILLSSFSPQKDMKKKSKKKKVVK--------------------------KNPDLKKTHG
SPPPQSTGMLPFVAG-------K------SASSS-HSVVANV-QSVLHMMKYRS-P--CVTSLYPEGTEN--
-----RTSRRAALS----RSVS-CTTSS-STTDSLSDLLLIMQDELGQMSFEHQE---LLKQIQETQDQDIR
EDLERELDCLVKQMEVKGEQISKLKKHQEHV--YKMRQRAQKLK--RKVASAAPNLDELKGTKETAVT--PR
HGVRSTCCANRSTSSLQLLKCAQKLQSVLKKDDIVWEQ
>EMP38037.1/1-500 Centrosomal protein CEP57L1 [Chelonia mydas]
---MLLSAKG-------PRP
PLTM---AAPGREPADTQTFLTFQKA--------
--SCTV-SALIG
KCKLLW---GEIEEGQ--
-------------------T---------------------------MGSESKQSFI----GSFLQPP----
-D-------KM-FVPTFGQSKSK------------KA------TATTGDM-LPAPN-------NQ-------
-------ALMSALKTLQEKIRRLELERSQAEDNLSCLSIEAAQYKKTLQHETNEKDIAHEELIQQKKDVSVQ
LSAAQSHCSLLEKQLDYMRKMVFNAELEKNMVLQQQ------------------------------------
-----------TQLQKEKDQNQMELHAKLEKLEVLEKECFRLSTTQRTAEDKIKQLEEKLREEEHHRKLTQD
KAAQLQTGLEINRILMSSVSPQNERKKKNRKKKTA-------------------------------------
----------------------K------SASSS-HSVSANV-QSVLHMMKHRS-P--CILSRCPEAAEH--
-----RISRRTIAC----KSVSSCSTSS-SVTENLSDLLLAIQDELGQMSFEHQE---LLKQIQETQNCEVR
EDLERELDCLVKQMEIKGEQISKLKKHQASVH--KLKQKAQKLK--RQSAHVLPKSDDLKGTREIPVT--SS
GRASKSCPVQKSKSSLQLLKNVQKLQSTLKKDDIMWEQ
>XP_039388685.1/1-472 centrosomal protein CEP57L1 isoform X2 [Mauremys reevesii]

--ME--
-------------------T---------------------------MGSESKQSFI----GSFLQPP----
-D-------RM-SVPTFGQSKSK------------KV------TTTTGDM-LPAPN-------NQ-------
-------ALMSALKTLQEKIRRLELERSQAEDNLSCLSIEAAQYKKTLQHETNVKDIAHEELIQQKKDVSVQ
LSAAQSRCSLLEKQLDYMRKIVFNAELEKNMVLEQQ------------------------------------
-----------IPLQKEKDQNHMELHAKLEKLEVLEKECFRLSTTQRTAEDKIKQLEEKLCEEEHQRKLIQD
KAAQLQTGLEINRILTSSVSSQNEPTKKSRKKKTAK--------------------------KKSALKKVHP
PQFCLKAGMLPFVAG-------K------SASSS-HSVSANV-QSVLHMMKHRS-P--CVLSRCVEADEH--
-----RISRRTIAC----KSASSCSTSS-SVTENLSDLLLAIQDELGQMSFEHQE---LLKQIQETQNCEVR
EDLERELDCLVKQMEIKGEQISKLKKHQASVH--KLKQKTQKLK--RQSA-VLPKSDDLKGTREIPVT--PS
RSASKNCPVQKSKSSLQLLKNVQKLQSTLKKDDIMWEQ
>XP_044867557.1/1-476 centrosomal protein CEP57L1 isoform X2 [Mauremys mutica]

---MV-----------

--GKLV--
-------------------T---------------------------MGSESKQSFI----GSFLQPP----
-D-------RM-FVPTFGQSKSK------------KV------TTTTGDM-LPAPN-------NQ-------
-------ALMSALKTLQEKIRRLELERSHAEDNLSCLSIEAAQYKKTLQHETNAKDIAHEELMQQKKDVSVQ
LSAAQSRCSLLEKQLDYMRKIVFNAELEKNMVLEQQ------------------------------------
-----------IPLQKEKDQNHMELHAKLEKLEVLEKECFRLSTTQRTAEDKIKQLEEKLREEEHQRKLIQD
KAAQLQTGLEINRILTSSVSSQNEPTKKSRKKKTAK--------------------------KKSALKKVHP
PQFCLKAGMLPFVAG-------K------SASSS-HSVSANV-QSVLHMMKHRS-P--CVLSRCVEADEH--
-----RISRRTIAC----KSASSCSTSS-SVTENLSDLLLAIQDELGQMSFEHQE---LLKQIQETQNCEVR

EDLERELDCLVKQMEIKGEQISKLKKHQASVH--KLKQKTQKLK--RQSA-VLPKSDDLKGTREIPVT--PS
RSASKNCPVQKSKSSLQLLKNVQKLQSTLKKDDIMWEQ
>XP_030411938.1/1-470 centrosomal protein CEP57L1 [Gopherus evgoodei]XP_030411939.1 centrosomal protein CEP57L1 [Gopherus evgoodei]

--MGSESKQSFI----GSFLQPP----
-D-------RM-FVPTFGQSKSK------------KV------TATTGDM-LPASN-------SQ-------
-------ALMSALKTLQEKIRRLELERSQAEDNLSCLSIEAAQYKKTLQLETNVKDIAHEELIQQKKDVSVQ
LSAAQSRCSLLEKQLDYMRKIVFNAELEKNMVLEQQ-----------------------------------
-----------TPLQKEKDQNHMELHAKLEKLEILEKECFRLSNTQRTAEDKIKQLEEKLREEEHQRKLIQD
KAAQLQTGLEINRMLTSSVSSQNEPTKKSRKKKIAK------------------------KKSALKKVHP
PQFCLKAGMLPFVAG-------K------SASSS-HSVSANV-QSVLHIMKHRS-P--CVLSRCVEADEH--
-----RISRRTTAC----KSASSCSTSS-SVTDNLSDLLLAIQDELGQMSFEHQE---LLKQIQETQNCEVR
EDLERELDCLVKQMEIKGEQISKLKKHQASVH--KLKQKAQKLK--RQSAHVLLKSDDLKGTREIPVT--PS
RSASKNCPVQKSKSSLQLLKNVQKLQSTLKKDDITWEQ
>XP_043398695.1/1-485 centrosomal protein CEP57L1 isoform X1 [Chelonia mydas]

--MADNTLFLLF-----------

--GEEK--
-------------------T---------------------------MGSESKQSFI----GSFLQPP----
-D-------KM-FVPTFGQSKSK------------KA------TATTGDM-LPAPN-------NQ-------
-------ALMSALKTLQEKIRRLELERSQAEDNLSCLSIEAAQYKKTLQHETNEKDIAHEELIQQKKDVSVQ
LSAAQSRCSLLEKQLDYMRKMVFNAELEKNMVLQQQ-----------------------------------
-----------TQLQKEKDQNQMELHAKLEKLEVLEKECFRLSTTQRTAEDKIKQLEEKLREEEHHRKLIQD
KAAQLQTGLEINRILMSSVSPQNERKKKNRKKKTAK------------------------KKSALKKVHP
PQLCLKAGMLPFVAG-------K------SASSS-HSVSANV-QSVLHMMKHRS-P--CILSRCPEAAEH--
-----RISRRTIAC----KSVSSCSTSS-SVTENLSDLLLAIQDELGQMSFEHQE---LLKQIQETQNCEVR
EDLERELDCLVKQMEIKGEQISKLKKHQASVH--KLKQKAQKLK--RQSAHVLPKSDDLKGTREIPVT--SS
GRASKSCPVQKSKSSLQLLKNVQKLQSTLKKDDIMWEQ
>XP_044867553.1/1-484 centrosomal protein CEP57L1 isoform X1 [Mauremys mutica]XP_044867554.1 centrosomal protein CEP57L1 isoform X1 [Mauremys mutica]XP_044867555.1 centrosomal protein CEP57L1 isoform X1 [Mauremys mutica]XP_044867556.1 centrosomal protein CEP57L1 isoform X1 [Mauremys mutica]KAH1173696.1 hypothetical protein KIL84_017535 [Mauremys mutica]

--MADNTLFLQL-----------

--REEK--
-------------------T---------------------------MGSESKQSFI----GSFLQPP----
-D-------RM-FVPTFGQSKSK------------KV------TTTTGDM-LPAPN-------NQ-------
-------ALMSALKTLQEKIRRLELERSHAEDNLSCLSIEAAQYKKTLQHETNAKDIAHEELMQQKKDVSVQ
LSAAQSRCSLLEKQLDYMRKIVFNAELEKNMVLEQQ-----------------------------------
-----------IPLQKEKDQNHMELHAKLEKLEVLEKECFRLSTTQRTAEDKIKQLEEKLREEEHQRKLIQD
KAAQLQTGLEINRILTSSVSSQNEPTKKSRKKKTAK------------------------KKSALKKVHP
PQFCLKAGMLPFVAG-------K------SASSS-HSVSANV-QSVLHMMKHRS-P--CVLSRCVEADEH--
-----RISRRTIAC----KSASSCSTSS-SVTENLSDLLLAIQDELGQMSFEHQE---LLKQIQETQNCEVR
EDLERELDCLVKQMEIKGEQISKLKKHQASVH--KLKQKTQKLK--RQSA-VLPKSDDLKGTREIPVT--PS
RSASKNCPVQKSKSSLQLLKNVQKLQSTLKKDDIMWEQ
>XP_039388683.1/1-484 centrosomal protein CEP57L1 isoform X1 [Mauremys reevesii]XP_039388684.1 centrosomal protein CEP57L1 isoform X1 [Mauremys reevesii]

--MADNTLFLQL-----------

--REEK--
-------------------T---------------------------MGSESKQSFI----GSFLQPP----
-D-------RM-SVPTFGQSKSK------------KV------TTTTGDM-LPAPN-------NQ-------

-------ALMSALKTLQEKIRRLELERSQAEDNLSCLSIEAAQYKKTLQHETNVKDIAHEELIQQKKDVSVQ
LSAAQSRCSLLEKQLDYMRKIVFNAELEKNMVLEQQ------------------------------------
-----------IPLQKEKDQNHMELHAKLEKLEVLEKECFRLSTTQRTAEDKIKQLEEKLCEEEHQRKLIQD
KAAQLQTGLEINRILTSSVSSQNEPTKKSRKKKTAK-------------------------KKSALKKVHP
PQFCLKAGMLPFVAG-------K------SASSS-HSVSANV-QSVLHMMKHRS-P--CVLSRCVEADEH--
-----RISRRTIAC----KSASSCSTSS-SVTENLSDLLLAIQDELGQMSFEHQE---LLKQIQETQNCEVR
EDLERELDCLVKQMEIKGEQISKLKKHQASVH--KLKQKTQKLK--RQSA-VLPKSDDLKGTREIPVT--PS
RSASKNCPVQKSKSSLQLLKNVQKLQSTLKKDDIMWEQ
>XP_050807359.1/1-485 centrosomal protein CEP57L1 isoform X1 [Gopherus flavomarginatus]

---MADNTLFLQL-----------

--PEEK--
------------------T---------------------------MGSESKQSFI----GSFLQPP----
-D-------RM-FVPTFGQSKSK------------KV------TATTGDM-LPASN-------NQ-------
-------ALMSALKTLQEKIRRLELERSQAEDNLSCLSIEAAQYKKTLQLETNVKDIAHEELIQQKKDVSVQ
LSAAQSRCSLLEKQLDYMRKIVFNAELEKNMVLEQQ------------------------------------
-----------TPLQKEKDQNHMELHAKLEKLEVLEKECFRLSTTQRTAEDKIKQLEEKLREEEHQRKLIQD
KAAQLQTGLEINRMLASSVSSQNEPTKKSRKKKIAK-------------------------KKSALKKVHP
PQFCLKAGMLPFVAG-------K------SASSS-HSVSANV-QSVLHMMKHRS-P--CVLSRCVEADEH--
-----RISRRTTAC----KSASSCSTSS-SVTDNLSDLLLAIQDELGQMSFEHQE---LLKQIQETQNCEVR
EDLERELDCLVKQMEIKGEQISKLKKHQASVH--KLKQKAQKLK--RQSAHVLPKSDDLKGTREIPVT--PS
RSASKNCPVQKSKSSLQLLKNVQKLQSTLKKDDITWEQ
>TFK14359.1/1-486 intraflagellar transport protein 81-like protein [Platysternon megacephalum]

---MADNTFFLRL-----------

--QEEK--
------------------T---------------------------MGSESKQSFI----GSFLQPP----
-D-------RM-FVPTFGQSKSK------------KV-----IIATTGDM-LPAPN-------NQ-------
-------ALMSALKTLQEKIRHLELERSQAEDNLSCLSIEAAQYKKTLQHETNAKDIAHEELIQQKKDVSVQ
LSAAQSHCSLLEKQLDYMRKMVFNAELEKNMVLEQQ------------------------------------
-----------TRLQKEKDQNHMELHAKLEKLEVLEKECFKLSTTQRTAEDKIKQLEEKLREEEHQRKLIQD
KAAQLQTGLEINRILTSSVSSQNEPKKKSRKKKTTK-------------------------KKSALKKVHP
PQFCLKAGMLPFVAG-------K------SASSS-HSVSANV-QSVLHMMKHRS-P--CVLSQCPEAAEH--
-----RISRRSIAC----KSVSSCSTSS-SVTENLSDLLLAIQDELGQMSFEHQE---LLKQIQETQNCEVR
EDLERELDCLIKQMEIKGEQISKLKKHQASVH--KLKQKTQKLK--RESAHVLPKSDDLKGTREIPVT--PS
GSASKNCPVQKSKSSLQLLKNVQKLQSTLKKDDIMWEQ
>XP_032620508.1/1-490 centrosomal protein CEP57L1 [Chelonoidis abingdonii]

--MPATVAAASLARARR-----------

--RQEK--
------------------T---------------------------MGSESKQSFI----GSFLQPP----
-D-------RM-FVPTFGQSKSK------------KV------TATTDDM-LPASN-------SQ-------
-------ALMSALKTLQEKICRLELERSQAEDNLSYLSVEAAQYKKTLQHETNVKDIEHEELIQQKKDVSVQ
LSAAQSRCSLLEKQLDYMRKIVFNAELEKNMVLEQQ------------------------------------
-----------TPLQKEKDQNHMQLHAKLEKLEILEKECFRLSTTQRTAEDKIKQLEEKLREEEHQRKLIQD
KAAQLQTGLEINRILTSSVSSQNEPTKKSRKKKTAK-------------------------KKSALKKVHP
PQFCLKAGMLPFVAG-------K------SASSS-HSVSANV-QSVLHMMKHRS-P--CVLSWCVEADEH--
-----RISRRTIAC----KSASSCSTSS-SVTDNLSDLLLVIQDELGQMSFEHQE---LLKQIQETQNCEVR
EDLERELDCLVKQMEIKGEQISKLKKHQASVH--KLKQKTQKLK--RQSAHVLPKSDDLKGTREIPVT--PS
RSASKNCPVQKSKSSLQLLKNVQKLQSTLKKDDIMWEQ
>XP_053879927.1/1-470 centrosomal protein CEP57L1 isoform X1 [Malaclemys terrapin pileata]

--MGSESKQSFI----GSFLQPP----
-D-------RM-FVPTFGQSKSK-----------KG------TATTGDM-LPAPN-------NQ-------
-------ALMSALKTLQEKIRRLELERSQAEDNLSCLSIEAAQYKKTLQHETNAKDLAHEELIQQKKDVSVQ
LSAAQSQCSLLEKQLDYMRKMVFNAELEKNMVLEQQ------------------------------------
-----------TQLQKEKDQKHMELHAKLEKLEVLEKECFRLLTTQRTAEDKIKILEEKLREEEHQRKLIQD
KAAQLQTGLEINRILTSSVSSQNEPKKKSRKKKTAK-------------------------KKSALKKVHP
PQFCLKAGMLPFVAG-------K------SASSS-HSVSANV-QSVLHMMKHRS-P--CISSQCPEAAEH--
-----RISRRTIAC----KSVSSRSTSS-SVTENLSDLLLAIQDELGQMSFEHQE---LLKQIQETQNCEVR
EDLERELDCLVKQMEIKGEQISKLKKHQASVH--KLKQKTQKLK--RQSAHVLPKSDDLKGTREIPVP--PS
GSASKNCPVQKSKSSLQLLKNVQKLQSTLKKDDIMWEQ
>XP_034621795.1/1-478 centrosomal protein CEP57L1 isoform X1 [Trachemys scripta elegans]

---MET-----------

---SLME--
------------------T---------------------------MGSESKQSFI----GSFLQPP----
-D-------RM-FVPTFGQSKSK-----------KG------TATTGDM-LPAPN-------NQ-------
-------ALMSALKTLQEKIRRLELERSQAEDNLSCLSIEAAQYKKTLQHETNAKDLAHEELIQQKKDVSVQ
LSAAQSQCSLLEKQLDYMRKMVFNAELEKNMVLEQQ------------------------------------
-----------TQLQKEKDQKHMELHAKLEKLEVLEKECFRLLTTQRTAEDKIKILEEKLREEEHQRKLIQD
KAAQLQTGLEINRILTSSVSSQNEPKKKSRKKKTAK-------------------------KKSALKKVHP
PQFCLKAGMLPFVAG-------K------SASSS-HSVSANV-QSVLHMMKHRS-P--CILSRCPEAAEH--
-----RISRRTIAC----KSVSSRSTSS-SVTENLSDLLLAIQDELGQMSFEHQE---LLKQIQETQNCEVR
EDLERELDCLVKQMEIKGEQISKLKKHQASVH--KLKQKTQKLK--RQSAHVLPKSDDLKGTREIPVP--PS
GSASKNCPVQKSKSSLQLLKNVQKLQSTLKKDDIMWEQ
>XP_023958262.1/1-478 centrosomal protein CEP57L1 isoform X1 [Chrysemys picta bellii]

---MEI-----------

---SLME--
------------------T---------------------------MGSESKQSFI----GSFLQPP----
-D-------RM-FVPTFGQSKSK-----------KG------TATAGDM-LPAPN-------NQ-------
-------ALMSALKTLQEKIRRLELERSQAEDNLSCLSIEAAQYKKTLQHETNAKDLAHEELIQQKKDVSVQ
LSAAQSHCSLLEKQLDYMRKMVFNAELEKNMVLEQQ------------------------------------
-----------TQLQKEKDQKHMELHAKLEKLEVLEKECFRLLTTQRTAEDKIKILEEKLREEEHQRKLIQD
KAAQLQTGLEINRILTSSVSSQNEPKKKSRKKKTAK-------------------------KKSALKKVHP
PQFCLKAGMLPFVAG-------K------SASSS-HSVSANV-QSVLHMMKHRS-P--CILSQCPEAAEH--
-----RISRRTIAC----KSVSSRSTSS-SVTENLSDLLLAIQDELGQMSFEHQE---LLKQIQETQNCEVR
EDLERELDCLVKQMEIKGEQISKLKKHQASVH--KLKQKTQKLK--RQSAHVLPKSDDLKGTREIPVP--PS
GSASKNCPVQKSKSSLQLLKNVQKLQSTLKKDDIMWEQ
>XP_024050060.1/1-470 centrosomal protein CEP57L1 [Terrapene carolina triunguis]

--MGSESKQSFI----GSFLQPP----
-D-------RM-FVPTFGQSKSK-----------KV------TATTGDM-LPAPN-------NQ-------
-------ALMSALKTLQEKIRRLELERSQAEDNLSCLSIEAAQYKKTLQHETNAKDLAHEELIQQKKDVSVQ
LSAAQSHCSLLEKQLDYMRKMVFNAELEKNMVLEQQ------------------------------------
-----------TQLQKEKDQNHMELHAKLEKLEVLEKECFRLLTTQRTAEDKIKQLEDKLREEEHQRKLIQD
KAAQLQIGLEINRILTSSVSSQNEPKKKSRKKKTAK-------------------------KKSALKKVHP
PQFCLKAGMLPFVAG-------K------SASSS-HSVSANV-QSVLHMMKHRS-P--CILSRCPEAAEH--
-----RISRRTIAC----KSISSRSTSS-SVTENLSDLLLAIQDELGQMSFEHQE---LLKQIQETQNCEVR
EDLERELDCLVKQMEIKGEQISKLKKHQASVH--KLKQKTQKLK--RQSAHVLPKSDDLKGTREIPVT--PS
GSASKNCPVQKSKSSLQLLKNVQKLQSTLKKDDIMWEQ
>XP_038253489.1/1-469 centrosomal protein CEP57L1 isoform X1 [Dermochelys coriacea]XP_038253492.1 centrosomal protein CEP57L1 isoform X1 [Dermochelys coriacea]XP_038253493.1 centrosomal protein CEP57L1 isoform X1 [Dermochelys

coriacea]XP_038253494.1 centrosomal protein CEP57L1 isoform X1 [Dermochelys coriacea]XP_038253495.1 centrosomal protein CEP57L1 isoform X1 [Dermochelys coriacea]XP_043366721.1 centrosomal protein CEP57L1 isoform X1 [Dermochelys coriacea]

---MGSESKQSFI----GSFLQPP----
-D-------KM-FVPTFGQSKSK------------KA------TATTGDM-LPAPN-------NQ-------
-------ALMSALKTLQEKIRHLELERSQAEDNLSFLSIEAAQYKKTLQHETNEKDLAHEELIQQKKDVSVQ
LSAAQSRCSLLEKQLDYMRKMVFNAELEKNMVLEQQ------------------------------------
-----------TQLQKEKDRNQMELHAKLEKLEVLEKECFRFSTTQRTAEDKIKQLEEKLCEEEHQRKLIED
KAAQLQTGLEINRILMSSVSPQNEPKKKNQKKKTAK--------------------------KKSALKKVHP
PQFCLNAGMLPFVAG-------K------SASSS-HSVSANV-QSVLHMMKHRS-P--CVLSRCPEAAEH--
-----RISRRTIAC----KSVSSCST-S-SVTENLSDLLLAIQDELGQMSFEHQE---LSKQIQETQNCEVR
EDLERELDCLVKQMEIKGEQISKLKKHQASVH--KLKQKAQKLK--RQSAHVLPKSDDLKETREIPVT--SS
GRASKSCPVQKSKSSLQLLKNVQKLQSTLKKDDIMWEQ
>XP_048700801.1/1-470 centrosomal protein CEP57L1 isoform X1 [Caretta caretta]XP_048700802.1 centrosomal protein CEP57L1 isoform X1 [Caretta caretta]

---MGSESKQSFI----GSFLQPP----
-D-------KM-FVPTFGQSKSK------------KA------TATIGDM-LPAPN-------NQ-------
-------ALMSALKTLQEKIRHLELERSQAEDNLSCLSIEAAQYKKTLQHETNEKDIAHEELIQQKKDVSVQ
LSAAQSRCSLLEKQLDYMRKMIFNAELEKNMVLEQQ------------------------------------
-----------TLLQKEKDQNQMELHAKLEKLEVLEKECFRLSTTQRTAEDKIKQLEEKLCEEEHQRKLIQD
KAAQLQTGLEINRILMSSVSPQNEPKKKNRKKKTAK--------------------------KKSALKKVHP
PQFCLKAGMLPFVAG-------K------SASSS-HSVSANV-QSVLHMMKHRS-P--CVLSRCPEAAEH--
-----RISRRTIAC----KSVSSCSTSS-SVTENLSDLLLAIQDELGQMSFEHQE---LLKQIQETQNCEVR
EDLERELDCLVKQMEIKGEQISKLKKHQASVH--KLKQKAQKLK--RQSAHVLPKSDDLKGTREIPVT--SS
GRASKSCPVQKSKSSLQLLKNVQKLQSTLKKDDIVWEQ
>XP_025040509.1/1-477 centrosomal protein CEP57L1 isoform X3 [Pelodiscus sinensis]

---MHLAAKK----------------

-------------------T---------------------------MDSESKQSFI----GSFLQPP----
-D-------RM-FAPTFGQSTLK------------KV------TATTNDV-LPAPN-------NQ-------
-------ALLSALQTLQEKIRRLELERSQAEDNLCCLSIEAAQYKKALQRETNEKDIAHGEMIQQKKDVSVQ
LSAAQSRCSLLEKQLDYMRKMVFNAELEKNMVLEQQ------------------------------------
-----------TLLQKEKDQNQMELHAKLEKLEALEKECFRLISTQRTAEDKIKQLEEKLREEEHQRKLIQD
KAAQLQTGLEINRILMSSESLQNEPKKRNRKKKTAK--------------------------KKSALKKVHP
PHFCLKAGMLPFVAG-------K------SASSS-HSVSANV-QSILHLMKHRS-P--CVLSRCPEPAEH--
-----RISRRTIAC----KSVSSCST-S-SVTENLSDLLLAIQDELGQMSFEHQE---LLKQIQETQNCEVR
EDLERELDCLVKQMEIKGEQISKLKKHQASVH--KLKQKVQKLK--KQSAHVLPKSDDLNGTREVSVT--PS
KSASKTCPVQKSKSSLHLLKNAQKLQSTLKKDDILWEQ
>XP_025040508.1/1-482 centrosomal protein CEP57L1 isoform X2 [Pelodiscus sinensis]

---MAARAG---------------

--SRQARG--
-------------------T---------------------------MDSESKQSFI----GSFLQPP----
-D-------RM-FAPTFGQSTLK------------KV------TATTNDV-LPAPN-------NQ-------
-------ALLSALQTLQEKIRRLELERSQAEDNLCCLSIEAAQYKKALQRETNEKDIAHGEMIQQKKDVSVQ
LSAAQSRCSLLEKQLDYMRKMVFNAELEKNMVLEQQ------------------------------------
-----------TLLQKEKDQNQMELHAKLEKLEALEKECFRLISTQRTAEDKIKQLEEKLREEEHQRKLIQD

KAAQLQTGLEINRILMSSESLQNEPKKRNRKKKTAK-------------------------KKSALKKVHP
PHFCLKAGMLPFVAG-------K------SASSS-HSVSANV-QSILHLMKHRS-P--CVLSRCPEPAEH--
-----RISRRTIAC----KSVSSCST-S-SVTENLSDLLLAIQDELGQMSFEHQE---LLKQIQETQNCEVR
EDLERELDCLVKQMEIKGEQISKLKKHQASVH--KLKQKVQKLK--KQSAHVLPKSDDLNGTREVSVT--PS
KSASKTCPVQKSKSSLHLLKNAQKLQSTLKKDDILWEQ
>XP_019375663.1/1-471 PREDICTED: centrosomal protein CEP57L1 isoform X1 [Gavialis gangeticus]XP_019375664.1 PREDICTED: centrosomal protein CEP57L1 isoform X1 [Gavialis gangeticus]

--MDSESKHSFI----GSFLQPP----
-D-------KS-KYSTFAHRKSK-----------KL------TTAVGHL-PSGPD-------NQ-------
------VALMAALKTLQEKIHRLELERSQAEENLSSLSVEAAQYKKALHHESCEKDIAHQELMQQKKDVSVQ
LSAAQSHCSLLEKQLDYMRKMVFSAEQDKKMVLQQQ------------------------------------
-----------TQLQKEKNQNQMELHAKLEKLEVLEKECFKLTTTQKTAEVKIKQLEEKLREEEHQRKLIQD
KAAQLQTGFEINRILMSSVSSQNEPKRKNRKKKTVK-------------------------KNPSLKKVTP
PQFYVKTGVLPFVAG-------K------SASSS-HSVSANV-QSVLHMMKHRS-P--CISSQHPEKAER--
-----RVSRRTIAS----KSVSSCSTSS-SVTENLSDLLLAIQDELGQMSFEHQE---LLKQIQETENCKVR
EDLERELDCLVKRMEIKGDQISKLKKHQASVQ--KLREKAQKLK--REAAYVKLKSDDMEGIKEIPVT--PK
ENGSISCPGQKSKRCLQLLKNMQKLQSTLKKDDIMWEQ
>XP_019393417.1/1-471 PREDICTED: centrosomal protein CEP57L1 isoform X1 [Crocodylus porosus]XP_019393418.1 PREDICTED: centrosomal protein CEP57L1 isoform X1 [Crocodylus porosus]

--MDSESKHSFI----GSFLQPP----
-D-------KS-KYSTFAHRKSK-----------KL------TTAVGDL-PSVPD-------NQ-------
------VALMAALKTLQEKIHRLELERSQAEENLSSLSVEAAQYKKALHHESCEKDIAHQELMQQKKDVSVQ
LSAAQSRCSLLEKQLDYMRKMVFSAEQDKKVVLQQQ------------------------------------
-----------TQLQKEKNQNQMELHAKLEKLEILEKECFKLTTTQKTAEVKIKQLEEKLCEEEHQRKLIQD
KAAQLQTGLEINRILMSSVSSQNEPKRKNRKKKTVK-------------------------KNPSLKKVTP
PQFYVKTGVLPFVAG-------K------SASSS-HSVSANV-QSVLHMMKHRS-P--CVSSQHPEKAER--
-----RVSRRTIAS----KSVSSCSTSS-SVTENLSDLLLAIQDELGQMSFEHQE---LLKQIQETENCKVR
EDLERELDCLVKRMEIKGDQISKLKKHQASVQ--KLREKAQKLK--REAAHVKLKSDDMEGIKEIPVT--PK
ENGSISCPGQKSKRSLQLLKNMQKLQSTLKKDDIMWEQ
>XP_006029978.1/1-471 centrosomal protein CEP57L1 isoform X1 [Alligator sinensis]XP_006029979.1 centrosomal protein CEP57L1 isoform X1 [Alligator sinensis]XP_025067195.1 centrosomal protein CEP57L1 isoform X1 [Alligator sinensis]

--MDSESKHSFI----GSFLQPP----
-D-------KS-KYSTFAHRKSK-----------KL------TTAMGDL-PSGPD-------NQ-------
------VALMAALKTLQEKIHRLELERSQAEENLSSLSVEAAQYKKALHHESCERDITHQELMQQKKDVSMQ
LSAAQSRCSLLEKQLDYMRKMVFSAEQEKKMVLQQQ------------------------------------
-----------TQLQKEKNQNQMELHAKLEKLEVLEKECFKLTTTQKTAEDKIKQLEEKLREEEHQRKLIQD
KAAQLQTGLEINRILMSSVSSQNEPKRKNRRKKTVK-------------------------KNPSLKKVTP
PQFYVKTGVLPFVAG-------K------SASSS-HSVSANV-QSVLHMMKYRS-P--CVSSQYPEKAEC--
-----RVSRRTIAS----KSVSSCSTSS-SVTENLSDLLLAIQDELGQMSFEHQE---LVKQIQETENCEVR
EDLERELDCLVKRMEIKGDQISKLKKHQASVQ--KLREKAQKLK--REAARVKLKSDDMEGIKEIPVT--PK
ENDSISCPGQKSKSSLQLLKNMQKLQSTLKKDDIMWEQ
>KYO24703.1/1-501 centrosomal protein CEP57L1 isoform B [Alligator mississippiensis]

--MAPAAG------P--------

--SVNY-NSQEA
TRKMRQ---EQRRAEQ--
-------------------T---------------------------MDSESKHSFI----GSFLQPP----
-D-------KS-KYSTFAHRKSK------------KL------TTAMGDL-PSGPD-------NQ-------
------VALMAALKTLQEKIHRLELERSQAEENLSSLSVEAAQYKKTLHHESCEKDITHQELMQQKKDVSMQ
LSAAQSRCSLLEKQLDYMRKMVFSAEQEKKMVLQQQ------------------------------------
-----------TQLQKEKNQNQMELHAKLEKLEVLEKECFKLTTTQKTAEDKIKQLEEKLHEEEHQRKLIQD
KAAQLQTGLEINRILMSSVSSQNEPKRKNRRKKTVK--------------------------KNPSLKKVTP
PQFYVKTGVLPFVAG-------K------SASSS-HSVSANV-QSVLHMMKYRS-P--CVSSQYPEKAEC--
-----RVSRRTIAS----KSVSSCSTSS-SVTENLSDLLLAIQDELGQMSFEHQE---LLKQIQETENCEVR
EDLERELDCLVKQMEIKGDQISKLKKHQASVQ--KLREKAQKLK--REAARVKLKSDDMEGIKEIPVT--PK
ENYSISCPGQKSKSSLQLLKNMQKLQSTLKKDDIMWEQ
>NXS46254.1/1-431 CE57L protein [Balaeniceps rex]

---MDSESKNSFI----GSFLQPP----
-D-------KM--PEAFAYTESE------------KL------AAVGGDR-PSTPN-------NQ-------
-------AMVAALKTLQEKIRRLELEKSQAEDNLCSLSIVAAQYKKALEHESYRKDTAHQELMQKRKDVSVQ
LNAAQSRCSLLEKQLDYMREMVSSAELEKKMVLEQQ------------------------------------
-----------AQLKKEEDQNRLELHAKLEKLEMLEKECLKLTATQKIAEDKIKHLEEKLCKEEHQRKLIQD
KTAQLQVGLEINRILMSSVTSQNEPKKENGKKKKPK-------------------K------------KMHP
SQLHVKAGELPFVAG-------K------------------V-------------------------TTS--
-----GISGRSALS----KSVSSRSTSP-TG-EGLSDLLLAIQDELGQMSFEHQE---LLKQMQETQDSKVR
EDLEQELDCLVKQMEIKGEQISKLKKHQATVQ--KLKRKTQKLK--QGAAHVKLNCGNQKEAKEIAVT--VR
ESMSKSCPGQKSRSSLQLLKNVQKLQSTLKKTDTMWEQ
>NXW06856.1/1-433 CE57L protein [Fregetta grallaria]

---NSFI----GSFLQPP----
-D-------KM-LPAAFAYIESK------------KL------AAVGGDR-PSIPT-------NQ-------
-------AVVAALKTLQEKIRRLELEKAQAEDNLCSLSVAAAQYKKALEHESCKKDTAHQELMQQRKDVSAQ
LSAAQSRCSLLEKQLDYMRKMVSSAELEKKMVLEQQ------------------------------------
-----------AQLQKEEDQNRLALHAKLEKLEMLEKECLKLTATQRIAEDKIKHLEEKICKEEHQRKLIQD
KTAQLQRGFEINRILMSSVTSQNEPKKENRKKKKTK-------------------M------RNPTTKKMHL
SQLHVKAGELPFVAG-------K------------------V-------------------------TTS--
-----GISGRSELP----KSVSSCSTSP-TATRSLSDLLLAIQDELGQMSFEHQE---LLKQIQETQDSKVR
EDLGQELNCLVKQMEIKGEQISKLKKHQATVQ--KLKRKTQKLK--QGAAHIKLKCGEQKEAKEIAVT--GR
ESMSKSCPGQKSKSSLQLLKTVQKLQSTLKKDDIMWEQ
>KAF1620095.1/1-438 Centrosomal protein CEP57L1, partial [Eudyptes robustus]

---MDSESKNSFI----GSFLQPP----
-D-------KM-LPAAFAYIESK------------KL------AAVGGDK-PSVPN-------NQ-------
-------AVVAALKTLQEKIRRLELEKSQAEDNLCSLSIAAAQYRKALEHESYKKDTAHQELMQQRKDISVQ
LNAAQSRCSLLEKQLDYMRKMVSSAELEKEMVLE-Q------------------------------------
-----------AQLQKEEDQNRLELHAKLEKLEILEKECLKLTATQRIAEDKIKHLEEKLCKEEHQRKLIQD
KTAQLQTGFEINRILMSSVTSQNEPKKENEQKKKTK-------------------K------RNPTMKKTHL
SQLHVKAGELPFVAG-------K------V-------------------------------------TTS--
-----GISGRSALS----KSVSSCSTSP-TATTSLSDLLLAIQDELGQMSFEHEE---LLKQIQETQDSKVR
EDLEGELDCLVKQMEIKGEQISELKKHQATVQ--KLKIKTQKLK--QGTAHVKLKCGDQKEEKKIAVT--VR
ESVSKSCPGQKSRSSLQLLKNVQKLQSTLKKDDIMWEQ
>KAF1563393.1/1-438 Centrosomal protein CEP57L1, partial [Eudyptes
pachyrhynchus]

---MDSESKNSFI----GSFLQPP----
-D-------KM-LPAAFAYIESK------------KL------AAVGGDK-PSVPN-------NQ-------
-------AVVAALKTLQEKIRRLELEKSQAEDNLCSLSIAAAQYRKALEHESYKKDTAHQELMQQRKDISVQ
LNAAQSRCSLLEKQLDYMRKMVSSAELEKEMVLE-Q------------------------------------
-----------AQLQKEEDQNRLELHAKLEKLEILEKECLKLTATQRIAEDKIKHLEEKLCKEEHQRKLIQD
KTAQLQTGFEINRILMSSVTSQNEPKKENEQKKKTK-------------------K------RNPTMKKTHL
SQLHVKAGELPFVAG-------K------V-------------------------------------TTS--
-----GISGRSALS----KSVSSCSTSP-TATRSLSDLLLAIQDELDQMSFEHEE---LLKQIQETQDSKVR
EDLEGELDCLVKQMEIKGEQISELKKHQATVQ--KLKIKTQKLK--QGTAHVKLKCGDQKEEKKIAVT--VR
ESVSKSCPGQKSRSSLQLLKNVQKLQSTLKKDDIMWEQ
>KAF1558477.1/1-438 Centrosomal protein CEP57L1, partial [Eudyptes schlegeli]KAF1615545.1 Centrosomal protein CEP57L1, partial [Eudyptes chrysolophus]

---MDSESKNSFI----GSFLQPP----
-D-------KM-LPAAFAYIESK------------KL------AAVGGDK-PSIPN-------NQ-------
-------AVVAALKTLQEKIRRLELEKSQAEDNLCSLSIAAAQYRKALEHESYKKDTAHQELMQQRKDISVQ
LNAAQSRCSLLEKQLDYMRKMVSSAELEKEMVLE-Q------------------------------------
-----------AQLQKEEDQNRLELHAKLEKLEILEKECLKLTATQRIAEDKIKHLEEKLCKEEHQRKLIQD
KTAQLQTGFEINRILMSSVTSQNEPKKENEQKKKTK-------------------K------RNPTMKKTHL
SQLHVKAGELPFVAG-------K------V-------------------------------------TTS--
-----GISGRSALS----KSVSSCSTSP-TATRSLSDLLLAIQDELGQMSFEHEE---LLKQIQETQDSKVR
EDLEGELDCLVKQMEIKGEQISELKKHQATVQ--KLKIKTQKLK--QGTAHVKLKCGDQKEEKKIAVT--VR
ESVSKSCPGQKSRSSLQLLKNVQKLQSTLKKDDIMWEQ
>KAF1635255.1/1-438 Centrosomal protein CEP57L1, partial [Eudyptes filholi]

---MDSESKNSFI----GSFLQPP----
-D-------KM-LPAAFAYIESK------------KL------AAVGGDK-PSVPN-------NQ-------
-------AVVAALKTLQEKIRRLELEKSQAEDNLCSLSIAAAQYRKALEHESYKKDTAHQELMQQRKDTSVQ
LNAAQSRCSLLEKQLDYMRKMVSSAELEKEMVLE-Q------------------------------------
-----------AQLQKEEDQNRLELHAKLEKLEILEKECLKLTATQRIAEDKIKHLEEKLCKEEHQRKLIQD
KTAQLQTGFEINRILMSSVTSQNEPKKENEQKKKTK-------------------K------RNPTMKKTHL
SQLHVKAGELPFVAG-------K------V-------------------------------------TTS--
-----GISGRSALS----KSVSSCSTSP-TATRSLSDLLLAIQDELGQMSFEHEE---LLKQIQETQDSKVR
EDLEGELDCLVKQMEIKGEQISELKKHQATVQ--KLKIKTQKLK--QGTAHVKLKCGDQKEEKKIAVT--VR
ESVSKSCPGQKSRSSLQLLKNVQKLQSTLKKDDIMWEQ
>KAF1644373.1/1-438 Centrosomal protein CEP57L1, partial [Eudyptes chrysocome]

---MDSESKNSFI----GSFLQPP----
-D-------KM-LPAAFAYIESK------------KL------AAVGGDK-PSIPN-------NQ-------
-------AVVAALKTLQEKIRRLELEKSQAEDNLCSLSIAAAQYRKALEHESYKKDTAHQELMQQRKDTSVQ
LNAAQSRCSLLEKQLDYMRKMVSSAELEKEMVLE-Q------------------------------------
-----------AQLQKEEDQNRLELHAKLEKLEILEKECLKLTATQRIAEDKIKHLEEKLCKEEHQRKLIQD
KTAQLQTGFEINRILMSSVTSQNEPKKENEQKKKTK-------------------K------RNPTMKKTHL
SQLHVKAGELPFVAG-------K------V-------------------------------------TTS--
-----GISGRSALS----KSVSSCSTSP-TATRSLSDLLLAIQDELGQMSFEHEE---LLKQIQETQDSKVR
EDLEGELDCLVKQMEIKGEQISELKKHQATVQ--KLKIKTQKLK--QGTAHVKLKCGDQKEEKKSAVT--VR
ESVSKSCPGQKSRSSLQLLKNVQKLQSTLKKDDIMWEQ
>KAF1515909.1/1-438 Centrosomal protein CEP57L1, partial [Eudyptes sclateri]

```
------------------------------------------------------------------------
------------------------------------------------------------------------
------------------------------------------------------------------------
-----------------------------------------------MDSESKNSFI----GSFLQPP----
-D-------KM-PPAAFAYIESK------------KL------AAVGGDK-PSIPN-------NQ-------
-------AVVAALKTLQEKIRRLELEKSQAEDNLCSLSIAAAQYRKALEHESYKKDTAHQELMQQRKDTSVQ
LNAAQSRCSLLEKQLDYMRKMVSSAELEKEMVLE-Q------------------------------------
-----------AQLQKEEDQNRLELHAKLEKLEILEKECLKLTATQRIAEDKIKHLEEKLCKEEHQRKLIQD
KTAQLQTGFEINRILMSSVTSQNEPKKENEQKKKTK-------------------K------RNPTMKKTHL
SQLHVKAGELPFVAG-------K------V-------------------------------------TTS--
-----GISGRSALS----KSVSSCSTSP-TATRSLSDLLLAIQDELGQMSFEHEE---LLKQIQETQDSKVR
EDLEGELDCLVKQMEIKGEQISELKKHQATVQ--KLKIKTQKLK--QGTAHVKLKCGDQKEEKKIAVT--VR
ESVSKSCPGQKSRSSLQLLKNVQKLQSTLKKDDIMWEQ
>KAF1588294.1/1-438 Centrosomal protein CEP57L1, partial [Eudyptes moseleyi]
------------------------------------------------------------------------
------------------------------------------------------------------------
------------------------------------------------------------------------
------------------------------------------------------------------------
-----------------------------------------------MDSESKNSFI----GSFLQPP----
-D-------KM-LPAAFAYIESK------------KL------AAVGGDK-PSVPN-------NQ-------
-------AVVAALKTLQEKIRRLELEKSQAEDNLCSLSIAAAQYRKALEHESYKKDTAHQELMQQRKDISVQ
LNAAQSRCSLLEKQLGYMRKMVSSAELEKEMVLE-Q------------------------------------
-----------AQLQKEEDQNRLELHAKLEKLEILEKECLKLTATQRIAEDKIKHLEEKLCKEEHQRKLIQD
KTAQLQTGFEINRILMSSVTSQNEPKKENEQKKKTK-------------------K------RNPTMKKTHL
SQLHVKAGELPFVAG-------K------V-------------------------------------TTS--
-----GISGRSALS----KSVSSCSTSP-TATRSLSDLLLAIQDELGQMSFEHEE---LLKQIQETQDSKVR
EDLEGELDCLVKEMEIKGEQISELKKHQATVQ--KLKIKTQKLK--QGTAHVKLKCGDQKEEKKIAVT--VR
ESVSKSCPGQKSRSSLQLLKNVQKLQSTLKKDDIMWEQ
>KAF1467850.1/1-438 Centrosomal protein CEP57L1, partial [Megadyptes antipodes
antipodes]
------------------------------------------------------------------------
------------------------------------------------------------------------
------------------------------------------------------------------------
------------------------------------------------------------------------
-----------------------------------------------MDSESKNSFI----GSFLQPP----
-D-------KM-LPAAFAYIESK------------KL------AAVGGDK-PSIPN-------NQ-------
-------AVVAALKTLQEKIRRLELEKSQAEDNLCSLSIAAAQYRKALEHESYKKDTAHQELMQQRKDISVQ
LNAAQSRCSLLEKQLDYMRKMVSSAELEKEMVLE-Q------------------------------------
-----------AQLQKEEDQNRLELHAKLEKLEILEKECLKLTATQRIAEDKIKHLEEKLCKEEHQRKLIQD
KTAQLQTGFEINRILMSSVTSQNEPKKENEQKKKTK-------------------K------RNPTMKKTHL
SQLHVKAGELPFVAG-------K------V-------------------------------------TTS--
-----GISGCSALS----KSVSSCSTSP-TATRSLSDLLLAIQDELGQMSFEHEE---LLKQIQETQDSKVR
EDLEGELDCLVKQMDIKGEQISKLKKHQATVQ--KLKIKTQKLK--QGTAHVKLKCGDQKEEKKIAVT--VR
ESVSKSCPGQKSRSSLELLKNVQKLQSTLKKDDIMWEQ
>KAF1664889.1/1-438 Centrosomal protein CEP57L1, partial [Aptenodytes
patagonicus]
------------------------------------------------------------------------
------------------------------------------------------------------------
------------------------------------------------------------------------
------------------------------------------------------------------------
-----------------------------------------------MDSESKNSFI----GSFLQPP----
-D-------KM-LPAAFAYIKSK------------KL------AAVGGDK-PSIPN-------NQ-------
-------AVVAALKTLQEKIRRLELEKSQAEDNLCSLSIAAAQYKKALEHESYKKDTAHQELMQQRKDVSVQ
LNAAQSRCSLLEKQLDYMRKMVSSAELEKEMVLEQQ------------------------------------
-----------AQLQKEEDQNRLELHAKLEKLEILEKECLKLAATQRIAEDKIKHLEEKLCKEEHQRKLIQD
KTAQLQTGFEINRILMSSVTSQNEPKKENGQKKKTK-------------------K------RNPTMKKTHL
SQLHVKAGELPFVAG-------K------V-------------------------------------TTS--
-----GISGRSALS----KSVSSCSTSP-TATRSLSDLLLAIQDELDQMSFEHEE---LLKQIQETQDSKVR
EDLEGELDCLVKQMEIKGEQISKLKKHQATVQ--KLKIKTQKLK--QGAAHVKLKCGDQK-EKKIAVT--VR
ESMSKSCPGQKSRSSLQLLKNVQKLQSTLKKDDIMWEQ
```

>KAF1483898.1/1-439 Centrosomal protein CEP57L1, partial [Pygoscelis
antarcticus]

---MDSESKNSFI----GSFLQPP----
-D-------KM-LPAAFAYIESK------------KL------AAVGGDK-PSIPN-------NQ-------
-------AVVAALKTLQEKIRRLELEKSQAEDNLCSLSIAAAQYKKALEHESYKKDTAHQELMQQRKDVSVQ
LNAAQSRCSLLEKQLDYMRKMVSSAELEKEMVLEQQ------------------------------------
-----------AQLQKEEDQNRLELHAKLEKLEILEKECLKLAATQRIAEDKIKHLEEKLCKEEHQRKLIQD
KTAQLQTGFEINRILMSSVTSQNEPKKENRQKKKTK-------------------K------RNPTMKKTHL
SQLHVKAGELPFVAG-------K------V-------------------------------------TTS--
-----GISGRSALS----KSVSSCSTSP-TATRSLSDLLLAIQDELGQMSFEHEE---LLKQIQETQDSKVR
EDLEGELDCLVKQMEIKGEQISKLKKHQATVQ--KLKIKTQKLN--QGAAHVKLKCGDQKEEKKIAVT--VR
ESMSKSCPGQKSGSSLQLLKNVQKLQSTLKKDDIMWEQ
>KAK1212658.1/1-439 CE57L protein, partial [Pygoscelis papua]

---MDSESKNSFI----GSFLQPP----
-D-------KM-LPAAFAYIESK------------KL------AAVGGDK-PSIPN-------NQ-------
-------AVVAALKTLQEKIRRLELEKSQAEDNLCSLSIAATQYKKALEHESYKKDTAHQELMQQRKDVSVQ
LNAAQSRCSLLEKQLDYMRKMVSSAELEKEMVLEQQ------------------------------------
-----------AQLQKEEDQNRLELHAKLEKLEILEKECLKLAATQRIAEDKIKHLEEKLCKEEHQRKLIQD
KTAQLQTGFEINRILMSSVTSQNEPKKENGQKKKTK-------------------K------RNPTMKKTHL
SQLHVKAGELPFVAG-------K------V-------------------------------------TTS--
-----GISGRSALS----KSVSSCSTSP-TATRSLSDLLLAIQDELGQMSFEHEE---LLKQIQETQDSKVR
EDQEGELDCLVKQMEIKGEQISKLKKHQATVQ--KLKIKTQKLN--QGAAHVKLKCGDQKEEKKIAVT--VR
ESMSKSCPGQKSRSSLQLLKNVQKLQSTLKKDDIMWEQ
>KAF1672427.1/1-439 Centrosomal protein CEP57L1, partial [Pygoscelis
papua]KAK0681245.1 CE57L protein, partial [Pygoscelis papua]KAK1190269.1 CE57L
protein, partial [Pygoscelis papua]

---MDSESKNSFI----GSFLQPP----
-D-------KM-LPAAFAYIESK------------KL------AAVGGDK-PSIPN-------NQ-------
-------AVVAALKTLQEKIRRLELEKSQAEDNLCSLSIAATQYKKALEHESYKKDTAHQELMQQRKDVSVQ
LNAAQSRCSLLEKQLDYMRKMVSSAELEKEMVLEQQ------------------------------------
-----------AQLQKEEDQNRLELHAKLEKLEILEKECLKLAATQRIAEDKIKHLEEKLCKEEHQRKLIQD
KTAQLQTGFEINRILMSSVTSQNEPKKENGQKKKTK-------------------K------RNPTMKKTHL
SQLHVKAGELPFVAG-------K------V-------------------------------------TTS--
-----GISGRSALS----KSVSSCSTSP-TATRSLSDLLLAIQDELGQMSFEHEE---LLKQIQETQDSKVR
EDLEGELDCLVKQMEIKGEQISKLKKHQATVQ--KLKIKTQKLN--QGAAHVKLKCGDQKEEKKIAVT--VR
ESMSKSCPGQKSRSSLQLLKNVQKLQSTLKKDDIMWEQ
>KAF1396544.1/1-439 Centrosomal protein CEP57L1, partial [Spheniscus
humboldti]KAF1430864.1 Centrosomal protein CEP57L1, partial [Spheniscus
magellanicus]

---MDSESKNSFI----GSFLQPP----
-D-------KM-LPAAFAYIESK------------KL------AAVGGDK-PSIPN-------NQ-------
-------AVVAALKTLQEKIRRLELEKSQAEDNLCSLSIAAAQYRKALEHESYKKDTAHQELMQQRKDVSVQ
LNVAQSRCSLLEKQLDYMRKMVSSAELEKEMVLEQQ------------------------------------
-----------VQLQKEEDQNWLELHAKLEKLEILEKECLKLTATQRIAEDKIKHLEEKLCKEEHQRKLIQD
KTAQLQTGFEINRILMSSVTSQNEPKKENGQKKKTK-------------------K------RNPTMKKTHL

SQLHVKAGELPFVAG-------K------V------------------------------------TTS--
-----GISGRSTLS----KSVSSCSTSP-TATRSLSDLLLAIQDELGQMSFEHEE---LLKQIQETQDSKVR
EDLEGELDCLVKQMEIKGEQISKLKKHQATVQ--KLKIKTQKLK--QGAAHVKLKCGDQKEEKKIAVT--VR
ESVSKSCPGQKSRSSLQLLKNVQKLQSTLKKDDIMWEQ
>KAF1463819.1/1-439 Centrosomal protein CEP57L1, partial [Spheniscus demersus]

---MDSESKNSFI----GSFLQPP----
-D-------KM-LPAAFAYIESK------------KL------AAVGGDK-PSIPN-------NQ-------
-------AVVAALKTLQEKIRRLELEKSQAEDNLCSLSIAAAQYRKALEHESYKKDTAHQELMQQRKDVSVQ
LNAAQSRCSLLEKQLDYMRKMVSSAELEKEMVLEQQ------------------------------------
-----------VQLQKEEDQNWLELHAKLEKLEILEKECLKLTATQRIAEDKIKHLEEKLCKEEHQRKLIQD
KTAQLQTGFEINRILMSSVTSQNEPKKENGQKKKTK-------------------K------RNPTMKKTHL
SQLHVKAGELPFVAG-------K------V------------------------------------TTS--
-----GISGRSTLS----KSVSSCSTSP-TATRSLSDLLLAIQDELGQMSFEHEE---LLKQIQETQDSKVR
EDLEGELDCLVKQMEIKGEQISKLKKHQATVQ--KLKIKTQKLK--QGAAHVKLKCGDQKEEKKIAVT--VR
ESVSKSCPGQKSRSSLQLLKNVQKLQSTLKKDDIMWEQ
>KAF1435873.1/1-439 Centrosomal protein CEP57L1, partial [Spheniscus mendiculus]

---MDSESKNSFI----GSFLQPP----
-D-------KM-LPAAFAYIESK------------KL------AAVGGDK-PSIPN-------NQ-------
-------AVVAALKTLQEKIRRLELEKSQAEDNLCSLSIAAAQYRKALEHESYKKDTAHQELMQQRKDVSVQ
LNAAQSRCSLLEKQLDYMRKMVSSAELEKEMVLEQQ------------------------------------
-----------VQLQKEEDQNWLELHAKLEKLEILEKECLKLTATQRIAEDKIKHLEEKLCKEEHQRKLIQD
KTAQLQTGFEINRILMSSVTSQNEPKKENGQKKKTK-------------------K------RNPTMKKTHV
SQLHVKAGELPFVAG-------K------V------------------------------------TTS--
-----GISGRSTLS----KSVSSCSTSP-TATRSLSDLLLAIQDELGQMSFEHEE---LLKQIQETQDSKVR
EDLEGELDCLVKQMEIKGEQISKLKKHQATVQ--KLKIKTQKLK--QGAAHVKLKCGDQKEEKKIAVT--VR
ESVSKSCPGQKSRSSLQLLKNVQKLQSTLKKDDIMWEQ
>XP_009892172.1/1-451 PREDICTED: centrosomal protein CEP57L1 [Charadrius vociferus]

---MDSESKNSFI----GSFLHPP----
-D-------KL-LPAAFAYIESK------------KL------AAVGGDR-PSVPN-------NQ-------
-------AVVAALKTLQEKIRRLELEKSQAEDNLCSLSIAAAQCKKALEHESYKRDTAHQELMQQRKDVSVQ
LNAAQSRCSLLEKQLDYMRKMVSSAELEKKMVLEQQ------------------------------------
-----------AQLQKEENQNQLELHAKLEKLEMLEKECLKLTATQRTAEDKIKCLEEKLCKEEHQRKLIQE
KTAQLQMGLEINRILMSLVTSQKEPEKENRKKKKTR-------------------K------RNPTMKKTHL
SQLHVKAGELPFVAG-------K----------------------------HRN-P--RISPRSQGGATS--
-----GISGHSALS----KSVSSCSTSP-TANSSLSDLLLAIQDELGQMSFKHQE---VLKQIQETQDSKVR
EDLKRELDCLVKQMEIKGEQISKLKKHQATVQ--KLKRKTQKLK--QEAAHVKLKCGDQKEAKEIAVT--VR
KSMSKSCPGQKSRSSLQLLKSVQKLQSTLKKDDIMWEQ
>NXY72152.1/1-439 CE57L protein [Glareola pratincola]

---MDSESKNSFI----GSFLQPP----
-D-------KM-LPAAFAYIASK------------KL------AAVDSDR-PSIPN-------NQ-------
-------AVVAALKTLQEKIRHLELEKSQAEDNLRSLSVAAAQYRKALEHESYEKDAVHRELMQQKKDINVQ
LNAAQSRCSLLEKQLDYMRKMVSSAELEKKMVLEQQ------------------------------------
-----------AQLQKEEGQNQLELHAKLEKLEMLEKECFKLTATQRIAEDKIKHLEEKLCKDKHQRKLIQG

KTAQLQTGFETNRILMSLVTSQSEPKKENGKKKKTK-------------------K------RNPKMKKIHL
SQLHVKAGELPFVAG-------K------V-------TSSGI------------------------------
-------SGHSALS----KPVSSCSTSP-TATRSLSDLLLDIQDELGQMSFKHQE---LLKKMREIEDSKVL
EDLKRELDCLVKQMEIKGEQISKMKKHQATVQ--KLKRKTQKLK--QGTARVEVKCGDKKEAKEIAVT--VR
KNFSRSCPGQKSRSSLQLLKNVQKLQSTLKKDDIMWEQ
>NWT40437.1/1-439 CE57L protein [Chroicocephalus maculipennis]

---MDSQSKNSFV----GSFLQPP----
-D-------KM-LPAAFAYVESK------------KL------AAVGGDR-PSIPN-------NQ-------
-------ALVAALKTLQEKIHHLELEKSQVEDNLRSLSIAAAQYRKALKHESYKRDTVHQELMQQRKDISVQ
INAAQSRCSLLEKQLDYMRKMVSSAELEKKIILEQQ------------------------------------
-----------AQFQKEEDQKRLELHAKLEKLEMLEEVCFKLTATQRIAEDKIKHLEEKLCKEEHQRKLIQD
KTAQLQTAFEINRILMSLVTSQNEPKKENGKKKKTK-------------------K------RNPTMKKTHL
SQLHVKAGELPFVAG-------K------V-------TSSGI------------------------------
-------SAHSALS----KPVSSCSTSP-TATRNLSDLLLAIQDELGQMSFKHQE---LLKKIQEIEDSRVC
EGLEGELDCLVKQMEIKGEQISKLKKHQATVQ--KLKRKTQKLK--QGAAHVELKCGDKKEAKEIAVT--VR
KSFSKSCPGQKSRSSLQLLKYVQKLQSTLKKDGIMWEQ
>NWU52984.1/1-439 CE57L protein [Dromas ardeola]

---MDSESKNSFI----GSFLQPP----
-D-------KM-LPAAFAYIESK------------KL------AAAGGDR-PSIPN-------NQ-------
-------AVVAALKTLQEKIRHLELEKSQAEDNLCSLSVAAAQYRKALEHESYEKDTVHRELMQQRKDISVR
LNAAQSRCSLLEKQLDYMRKMVSSAELEKKMVLEQQ------------------------------------
-----------AQLQKEEDQNRLELHAKLEKLEMLEEECFKLTATQRIAEDKIKHLEEKLCKEEHQRKLIQD
KTAQLQTGFEINRILMSLVTSQNEPKKENGKKNRTK-------------------K------RNPTMNKMHL
SQLHVKAGELPFVAG-------K------V-------TSSGI------------------------------
-------SGHSALS----KPVSSCSTSP-TATRSFSDLLLAVQDELGQMSFKHQE---LLKKVQEIEDSKVC
EDLERQLDCLVKQMEIKGEQISKLKKHQATVQ--KLKRKTQKLK--EGAAHVELKRGDKKEAKEIAVT--VR
KSFSKSCPGEKSRSSLQLLKNVQKLQSTLKKDGIMWEQ
>NXV45773.1/1-440 CE57L protein [Uria aalge]

---MDSESKDSFI----GSFLQPP----
-D-------KM-LPAAFAYIESK------------KL------AAVGSDR-PSIPN-------NQ-------
-------AVVAALRTLQEKIHHLELEKSQAEDNLRSLSIAAAQYRKALEHESYERDTVHRELMQQRKDISVQ
LNAAQSRCSLLEKQLDYMRKMVSSAELEKKMVLEQQ------------------------------------
-----------AQLPKEEDQNRLELHAKLEKLEMLEKECFKLTATQRIAEDKIKHLEEKLCKEEHQRKLIQD
KTAQLQTGFEMNRILMSLVTSQNEPKKEKGKKKKTK-------------------K------RNPTMKKMHL
SQLHVKAGELPFVAG-------K------V------RVSSGI------------------------------
-------SGHSALS----KPVSSCSTSP-TATRSLSDLLLAIQDELGQMSFKHQE---LLKKIQEIEDSRVH
EDLEGELDYLVKQMEIKGEQISKLKKHQATVQ--KLKRKTQKTK--QGAAHVKLNCGDKKEAKEIAVT--VR
KSFSKSCPGQKSRSSLQLLKNVQKLQSTLKKDGIVWEQ
>NWX72974.1/1-445 CE57L protein [Alca torda]

---MDSESKDSFI----GSFLQPP----
-D-------KM-LPAAFAYIESK------------KL------AAVGSDR-PSIPN-------NQ-------
-------AVVAALRTLQEKIHHLELEKSQAEDNLRSLSIAAAQYRKALEHESYERDTVHRELMQQRKDISVQ
LNAAQSRCSLLEKQLDYMRKMVSSAELEKKMVLEQQ------------------------------------
-----------AQLQKEEDQNRLELHAKLEKLEMLEKECFKLTATQRIAEDKIKHLEEKLCKEEHQRKLIQD
KTAQLQTGFEINRILMSLVTSQNEPKKEKGKKKKTK-------------------K------RNPTMKKMHL

SQLHVKAGELPFVAG-------K------SVSSS-HSVSANV-----------------------------
-------QRHSALS----KPVSSCSTSP-TATRSLSDLLLAIQDELGQMSFKHQE---LLKKIQEIEDSRVH
EDLEVELDCLVKQMEIKGEQISKLKKHQATVQ--KLKRKTQKTK--QGAAHVKLNCGDKKEAKEIAVT--VR
KSFSKSCPGQKSRSSLQLLKNVQKLQSTLKKDGIMLEQ
>NXN60021.1/1-445 CE57L protein [Rynchops niger]

---MDSESKNSFI----GSFLQPP----
-D-------KM-LPAAFAYTESK------------KL------AAVGGDT-PSLPN-------NQ-------
-------AVVAALKTLQEKIHHLELEKSQAEDNLRSLSIAAAQYRKALEHESYERGTVHQELMQQRKDISVQ
LNAAQSRCSLLEKQLDYMRRMVSVAELEKKMVLEQQ------------------------------------
-----------AQFQKQEDQNRLELHAKLEKLEMIEKECFKLTATQRIAEDKIKHLEEKLCKEEHQHKLIQD
KTAQLQTGFEINRILMSLVTSQNEPKKENGKKKKTK-------------------K------RNPTMKKTHL
SQLHVKAGELPFVAG-------K------SVSSS-HSVSANV------------------------------
-------QRRGALS----KSVSSCSTSP-TATRSLSDLLLAIQDELGQMSFKHQE---LLKKIQEIEDSRVR
EDLEGELDCLVKQMEIKGEQISKLKKHQATVQ--KLKRKTQKLK--QGAAHVELKCGDKNEAKEIAVT--VR
KSFSKSCPGQKSRSSLQLLKNVQKLQSTLKKDGIVWEQ
>NXL48732.1/1-437 CE57L protein [Podilymbus podiceps]

---MDSESKNSFI----GSFLQLP----
-D-------KM-LPAAFAHGESK------------KL------AAVGGDR-PSIPN-------NQ-------
-------AVVAALKNLQEKIRRLELEKSQAEDNLCNLSMAAARFKKALERESYKKDVAHQELMQQRKDVSVQ
LNAAQSRCSLLEKQLDYMRKMVSSAELEKKMVLEQQ------------------------------------
-----------AQLQKEEDQNWLELHTKLEKLEVLEKECLKLTATQRIAEDKIKHLEDKLCKEEHQRKLIQD
KTAQLQTGFEINRILMSSVTSQNEPKKENERKKKTK-------------------K------RNPTRKKMHL
SQLHVKAGELPFVAG-------K-------------VRPNVLAK----------------------------
---------VVALS----KSVSSCSTSP-ATTRSLSDLLLAIQDELGQMSFEHQE---LLKHIQETQDSRVC
EALEQELDCLVKQMKIKGEQISKLKKHQATVQ--KLK--TQKLK--QGTAHVKLKCGDQKEAKEIKIS--VR
ENMSKSCPGQKSRSCLQLLKNVQKLQSTLKKDDIMWEQ
>KFZ54356.1/1-443 Centrosomal protein cep57l1 [Podiceps cristatus]

---MDSESKNSFI----GSFLQLP----
-D-------KM-LPAAFAHRESK------------KL------AAVGGDR-PSIPN-------NQ-------
-------AVVAALKTLQEKIRRLELEKSQAEDNLCNLSMAAARFKKALEHESYKKDVAHQELMQQRKDVSVQ
LNAAQSRCSLLEKQLDYMRKMVSSAELEKKMVLEQQ------------------------------------
-----------AQLQKEEDQNWLELHAKLEKLEVLEKECLKLTATQRIAEDKIKHLEDKLCKEEHQRKIIQD
KMAQLQTGFEINRILMSSVTSQNEPKKENERNKKTK-------------------K------RNPTMKKMHL
SQLHEKAGKLPFVAG-------K------SVSSS-HSVSANV-QR---------------------------
---------RSALS----KSVSSCSTLP-ATTRSLLDLLLAIQDELGQMSFEHQE---LLKHIQETQDSRVC
EALEQELDCLVKQMKIKGEQISKLKKHQATVQ--KLK--TQKLK--QGTAHVQLKCGDQKEAKEIKVT--VR
ENMSKSCPGQRSRSRSCLQLLKNVQKLQSTLKKDGIMWEQ
>KFV99501.1/1-441 Centrosomal protein CEP57L1, partial [Fulmarus glacialis]

---MDSESKNSFI----GSFLPPP----
-D-------KM-LPAAFAYIES-------------KL------AAVDVDR-PSIPN-------NQ-------
-------AVVAALKTLQEKIRRLELEKAQAEDNLCSLSIAAAQYKKALEHESYKKDTAHQELMQQKKDVSVQ
LNAAQSRCSLLEKQLDYMRKMVSSAELEKKMVLEQQ------------------------------------
-----------AQLQKEEDQNRLALHAKLEKLEMLEKVCLKLTATQRIAEDKIKHLEEKLCKEEHQRKLIQD
KTAQLQTGFEINRILMSSVTSQNEPKKENRKKKKTK-------------------K------WNSTMKKM--
-QLHVKAGELPFVAG-------K------SVSSS-HSVSANV-QR---------------------------

---------RSALS----KSVSSCSTSP-TATRSLSDLLLALQDELGQMSFEHQE---LLKQIQETQDSKVR
EDLEQELDCLIKQMEIKGEQISKLKKHQATVQ--KLKRKTQKLK--QGAAHVKLKCGDQKEAKKIAVT--VR
ESMSKSCPGQQSRSSLQLLKNVQKLQSTLKKDDIMWEQ
>XP_009586474.1/1-447 PREDICTED: centrosomal protein CEP57L1 [Fulmarus glacialis]

---MDSESKNSFI----GSFLPPP----
-D-------KM-LPAAFAYIES-------------KL------AAVDVDR-PSIPN-------NQ-------
-------AVVAALKTLQEKIRRLELEKAQAEDNLCSLSIAAAQYKKALEHESYKKDTAHQELMQQKKDVSVQ
LNAAQSRCSLLEKQLDYMRKMVSSAELEKKMVLEQQ------------------------------------
-----------AQLQKEEDQNRLALHAKLEKLEMLEKVCLKLTATQRIAEDKIKHLEEKLCKEEHQRKLIQD
KTAQLQTGFEINRILMSSVTSQNEPKKENRKKKKTK-------------------K------WNSTMKKM--
-QLHVKAGELPFVAG-------KHRNPRISSRSQ-GGATSGI-SG---------------------------
---------RSALS----KSVSSCSTSP-TATRSLSDLLLALQDELGQMSFEHQE---LLKQIQETQDSKVR
EDLEQELDCLIKQMEIKGEQISKLKKHQATVQ--KLKRKTQKLK--QGAAHVKLKCGDQKEAKKIAVT--VR
ESMSKSCPGQQSRSSLQLLKNVQKLQSTLKKDDIMWEQ
>NXH79175.1/1-444 CE57L protein [Oceanodroma tethys]

---MDSESKNSFI----GSFLQPP----
-D-------KM-LPGAFAYIESK------------KL------AAVGGDR-PSIPN-------NQ-------
-------AVVTALKTLQEKIRRLELEKAQAEDNLCSLSIAAAQYKKALEHESYKKDTAHQELMQQRKDVSVK
LNAAQSRCSLLEKQLDYMRKMVSSAELEKKMGLE-Q------------------------------------
-----------AQLQKEEDQNQLALHAILEKLEMLEKECLKLTATQRTAEDKIKHLEEKLCKEAHQRKLIQD
KTAQLQTGFEINRILMSSVTSQNEPKKENGKKKKTK-------------------K------RNPTMKKMHL
SQLHVKAGELPFVAG-------K------SVGSS-HSVSANV-QR---------------------------
---------RSALS----KSVSSCSTSP-TATRSLSDLLLAIQDELGQMSFDHQE---LLKQIQETQDSKVC
EDLEQKLDCLVKKMEIKGEQISKLKKHQATVQ--KLKRKTQKLK--QGAAHVKIKCGGQKEAKEIAVT--VR
ESMSKPCPGQKSRSSLQLLKNVQKLQSTLKKDDIMWEQ
>KFP50146.1/1-445 Centrosomal protein CEP57L1 [Cathartes aura]

---MDSESKNSFI----GSFLQPP----
-D-------KM-LPAAFAYIESK------------TL------AAVGGDR-PSIPN-------NQ-------
-------AVVAALKTLQEKIRRLELEKSQAEDNLCSLSIAAAQYKKALEHESYKKDTAHQELMQQRKDVSVQ
LNAAQSRCSLLEKQLDYMRKMVASAELEKKVVLEQQ------------------------------------
-----------AQVQKEEDQNRLELHAKLEKLEMLEKECLKLTATQRIAEDKIKHLEEKLCKEEHQRKLIQD
KTAQLQTGFEINRILMSSVTSQKEPKKENGKKKKTK-------------------K------RNPAMKQMHI
SQLRVKAGELPFVAG-------K------SVSSS-HSVSANV-QR---------------------------
---------RSALS----KSVSSCSTSP-TATRSLSDLLLAIQDELGQMSFVHQE---LLKQMQETQDSKVC
EYLEQELDCLVKQMETKGEQISKLKKHQATVQ--KLKRKTQKLK--QGAAHVKLKCDDQKKAKETAVT--VR
ESMSKLCPGQKSRSSLQLLQNVRKLQSTLKKDDIMWEQ
>KFQ95634.1/1-445 Centrosomal protein CEP57L1, partial [Nipponia nippon]

---MDSESKNSFI----GSFLQPP----
-D-------KL-LPAAFACIESK------------KL------AAVGCDR-SSIPN-------NQ-------
-------AVVAALKTLKEKIRRLELEKSQAEDNLCSLSIAAAQYKKALEHESYKKDTAHQELMQQRKDVSVQ
LNAAQSRCSLLEKQLDYMRKMVSSAELEKKMVLEQQ------------------------------------
-----------AQLKKEEVQNRLELHAKLEKLEMLEKECLKLTATQRIAEDKIKHLEEKLCKEEHQCKLMQD
KTAQLQTGFEINRILMSSVTSQNEHKKENGKKKKTK-------------------K------RNPAMKKMHL
PQLHVKAGELPFVAG-------K------SVGSS-HSVSANV-QR---------------------------

---------CSALS----KSVSSCSTSP-TATRSLSDLLLLIQDELGQMSFEHQE---LLKQIQETQDSKVH
EDLERELDCLVKRMEIKGEQISKLKKHQATVQ--KLKRKTQKLK--QGAAHIKLKCGDQKEAKEIAVT--VR
ECTSKSCPGQKSRSSLQLLKNVQKLQSTLKKDDIMWEQ
>NWH53765.1/1-445 CE57L protein [Fregata magnificens]

---MDSESKNSFI----GSFLQPP----
-D-------KM-LPAAFAYIESK------------KL------AAAGGDR-PSIPN-------NR-------
-------AVVAALKTLQEKIRRLELEKSQAEDNLCSLSIAAAQYKKALEHESYKKDTAHQELMQQRKDISVQ
LNAAQSRCSLLEKQLDYMRKMVSSAELEKKMVLEQQ------------------------------------
-----------AQLQKEEGQNWLELHAKLEKLEMLEKECLKLTVTQRIAEDKIKHLEEKLCKEEHQRKLIQD
RTAQLQTGFEINRILMSSVTSKNEPKKENGKKKKTK-------------------K------RNPTMKKMHL
SQLQVKAGELPFVAG-------K------SVGSS-HSVSANV-QR---------------------------
---------CSALS----KSVSSGSTSP-AATRSLSDLLLAIQDELGQMSFEHQE---LLKQIQDTQESKVR
EDLERELDCLVKQMEIKGEQITKLKKHQAAVQ--KLKRKTQKLK--QGAAYVKLKWGDQKEAKEIAVT--VR
ENMSKSCPGQKSRSSLQLLKNVQKLQSALKKDDIMWEQ
>KFQ54165.1/1-445 Centrosomal protein cep57l1, partial [Pelecanus crispus]

---MDSESKNSFI----GSFLQPP----
-D-------KM-LPAAFAYIESK------------KL------AAVCRDR-PSIPN-------NQ-------
-------AVVAALKTLQEKIRRLELEKSQAEDNLCSLSIAAAQYKKALEHESYKKGTAHQELMQQRKDISVQ
LNAAQSRCSLLEKQLDYMREMVSSAELEKKMVLEQQ------------------------------------
-----------AQLKKEEDQNRLELHAKLEKLEMLEKECLKLTATQRIAEDKIKHLEEKLCKEEHQRKLTQD
KTAQLQRGFEINRILMSSATSPNEPKKENGKKKKPK-------------------K------RNPTMKKMHL
SQLHVKAGELPFVAG-------K------SVGSS-HSVSANV-QR---------------------------
---------RSALS----KSVSSCSTSP-AASRSLSDLLLAIQDELGQMSFEHQE---LLKQIQETQDSKVR
EDLERELDCLVKQMEIKGEQISKLKKHQATVQ--KLKRKTQKLK--QGAAHVKLKCGDQKEAKEFAVT--VR
ENMSKSCPGQKSRSSLQLLKNVRKLQSTLKKDDIMWEQ
>KFQ89500.1/1-445 Centrosomal protein CEP57L1 [Phoenicopterus ruber ruber]

---MDSESKNSFI----GSFLQPP----
-D-------KM-LPAAFSYRESK------------KL------AAVGGDR-PSIPN-------YQ-------
-------AVVAALKTLQEKIRRLELEKSQAEDNLCSLSIAAAQYKKALEHESDKKDIAHQELMQQRKDVSVQ
LNAAQSRCSLLEKQLDYMRKMVSSAELEKKMILEQQ------------------------------------
-----------AQLQKEEDQNWLELHAKLEKLEVLEKECLKLTATQRIAEDKIKHLEDKLCKEEHQRKLIQD
KTTQLQTGFEINRILLSSVTSQNEPKKENEKKKKTK-------------------K------RNPTIKKMHL
SQLHVKAGELPFVAG-------K------SVSSS-HSVSANV-QR---------------------------
---------RSALS----KPVSSCSTSP-TATRSLSDLLLAIQDELGQMSFDHQE---LLKQIQETQDSRVH
QDLERKLDCLVKQMEIKGEQISKLKKHQATVQ--KLKRKTQKLK--QGTAHVKLKCGDQKKAKEIAVT--VK
ESMSKSCPGQKSRSCLQLLKNVQKLQSTLKKNDIMWEQ
>NXO47308.1/1-442 CE57L protein [Aramus guarauna]

---MDSASKNSFI----GSFLQPP----
-D-------KM-LPAAFAYIESK------------KL------AAVGGDG-PSIPN-------DQ-------
-------AVVAALKTLQEKIRRLELEKSQAEDNLCSLSIAAAQYKKALEHESYKKDTAHQELMQQRKDVSVQ
LNAAQSRCSLLEKQLDYMKKMVSSAELEKKMVLEQQ------------------------------------
-----------AQLQKEEDQNWLELRAKLEKLEMLEKECLKLTATQRIAEDKIKHLEEKLCKEEHQRKLMQD
KTVQLQTGFEINRILMSSVKSQNEPKKENGKKKKPK-------------------K------RNPAMKKMHL
SQLHVKAGELPFVAG-------K------SVSSS-HSVSANV-Q----------------------------
---------SVALS----KSVSSGPTSP-TATRSLSDLLLAIQDELGQMSFEHQE---LLKQIQETQDARVC

```
EHLEWELDCLVKQMEIKGEQISKLKKHQASVQ--KLKRKTQKLK--QGAAHVKLKRGDQKETKETA----VR
ESISRSCPGQKSRSSLQLLKNVRKLQSTLKKDDIMWEQ
>NXI90958.1/1-443 CE57L protein [Psophia crepitans]
------------------------------------------------------------------------
------------------------------------------------------------------------
------------------------------------------------------------------------
------------------------------------------------------------------------
-----------------------------------------------MDSESKNSFI----GSFLQPP----
-D-------KM-LPAAFAYIESK------------KL------AAIGGDR-PSIPN-------NQ-------
-------AVVAALKTLQEKIRRLELEKSQAEDNLCSLSIAAAQYKKALEHESYEKDTAHQELMQQRKDVSVQ
LNAAQSRCSLLEKQLDYMRKMVSSAELEKKMVLEQQ------------------------------------
-----------AQLQKEKDQNWLELCAKLEKLEMLEKECLKLTATQRIAEDKIKHLEEKLCKEEHQRKLIQD
KTVQLQTVFEINRILMSSVTSQNEPEKENGKKKKTK-------------------K------RNPMMKKMHL
SQFHIKAGELPFVAG-------T------SVSSS-HSVSANV-QR---------------------------
---------GSVLS----QSISSGSTSL-TATRSLSDILLAIQDELGQMTFEHQE---LLKQIEETQDTKVC
EHLERELDCLVKRMDMKAEQISKLKKHQASVQ--KLKRKTQKLK--QMTGHIKLKCGDQKETKETA----GR
ESMSKCSLGQKSRSSLQLLKNVQKLQSTLKKDDIMWEQ
>NXS61249.1/1-439 CE57L protein [Brachypteracias leptosomus]
------------------------------------------------------------------------
------------------------------------------------------------------------
------------------------------------------------------------------------
------------------------------------------------------------------------
-----------------------------------------------MDSESKNSFI----GRLLQPP----
-D-------KM-LPAAFAHKESK------------KL------AAVGSDR-PSIPN-------NQ-------
-------AVVAALKTLQEKIHCLELEKSQAEDNLCSLSIAAAQYKKALEHESYKKDIAHQELMQQRKDISVQ
LNAAQSRCSLLEKQLDYMRKMVSNAELENKMVLEQQ------------------------------------
-----------AQLQKEEDQNWLKLHAKLEKLEMLEKECLKLTATQRIAEDKIKHLEEKLCKEEHQRKLIQD
KTAQLQTGFEINRILMSSVTSQNEPKKENGKRKKTK-------------------K------RNPTMKKMHL
SPLLVKAGELPFVAG-------K------VTTSK-TS-----------------------------------
--------GHGAFS----KSLSFCSTSP-SATGSLSDLLLAVQDELGQMSFEHQE---LLKQIQETQDSRVR
EDLEQELDCLVKQMDIKGEQISKLKKHRATMQ--KLKRKIQELK--QGTAHVKLKCDSQKEAKEITLT--LR
ESMSKSCPGQKSRSSLQLLKNVQKLQSTLKKDDIMWEQ
>NXW56482.1/1-443 CE57L protein [Eurystomus gularis]
------------------------------------------------------------------------
------------------------------------------------------------------------
------------------------------------------------------------------------
------------------------------------------------------------------------
-----------------------------------------------MDSESKNSFI----GSFLQPP----
-D-------KK-LPAAFAHKESK------------KL------AAVGGHR-PSIPN-------NQ-------
-------AVVTALKTLQEKIHCLELEKSQAEDNLCNLSIAAAQYKKALEHESYKKDIAHQELMQQRKDVSVQ
LNAAQSRCSLLEKQLDYMRKMVSSAELENKMVLEQQ------------------------------------
-----------AQLQKEEDRNWLELHAKLEKLEMLEKECLKLSATQRIAEDKIKHLEEKLCKEEHQRKLIQD
KTAQLQTGFEINRILMSSVTSQNGPKKENGKKKKTK-------------------K------RNPTMKKMHL
SQLHVKAGELPFVAG-------K------SVSSS-HSVSANV------------------------------
-------QRHSAFS----KSLSSCSTLP-TATGSLLHLLLAIQDELGQMSFEHQE---LLKQIQETQDSKVR
EDLEQELDCLVKQMDIKGEQISKLKKHQASVQ--KL--KTQKLK--QGAAHVKLKCDGQKEAKEIAVT--VR
ESVSKTCPGQKSRSSLQLLKNVQKLQSTLKKDDIMWEK
>NWY50920.1/1-436 CE57L protein [Chionis minor]
------------------------------------------------------------------------
------------------------------------------------------------------------
------------------------------------------------------------------------
------------------------------------------------------------------------
-----------------------------------------------MEYESKNSFI----GSFFQPP----
-D-------KI-LPAAFAYTESK------------NL------AAV-CDR-PSIPS-------NQ-------
-------AVMAALKTLQEKIHRLELEKSQAEDNLCSLSIAAAQYKKALEHESYKKDKAHQELMQQRKDVSVQ
LNAAQSRCSLLEKQLDYMRKMVSSAELEKKMVLEQQ------------------------------------
-----------AQLQKEEDQNWLELHAKLEKLEMLEKECLKLSATQRTAEDKIRHLEEKLRKEEHQRKLIQD
KTAQLQVGFEINRMLMSSVTSQNEPKKENGKKKKTK-------------------K------RNPTMKKMHL
LQLHVKAGELPFVAG-------K--------------VSESK------------------------------
--------WHSALS----KSVSSCYASP-IDTRNLPDLLLAMQDELGQMSFEHQE---LLKQIQEIQDSKVR
EGLERELDCLVKQMEIKGEQICKLKKHQATVQ--KLKRKTQILK--QEAAHVKQKCGDQKEAKEIAVT--IR
```

KSVSKSCAGQKSRNSLQLLKNVQKLQSTLKKDGIMWEQ
>NXT51243.1/1-437 CE57L protein [Pluvianellus socialis]

---MDSESKNSFI----GSFFQPP----
-D-------KM-LPAALAYTESK------------KL------AAVGGDR-PSIPS-------NQ-------
-------AVMAALKTLQEKIHRLELEKSQAEDNLCSLSIAAAQYKKALEHESYKKDKAHQELMQQRKDVSVQ
LNAAQSRCSLLEKQLDYMRKMVSSAELEKKMVLEQQ------------------------------------
-----------AQLQKEEDQNWLELHAKLEKLEMLEKECLKLSATQRTAEDKIKHLEEKLRKEEHQRKLIQD
KTAQLQVGFEINRMLMSSVTSQNEPKKENGKKKKTK-------------------K------RNPTMKKTHL
LELQVKAGELPFVAG-------K--------------VSESK------------------------------
--------WHSALS----KSVSSCYASP-TDTRNLLDLLLAVQDELGQMSFEHQE---LLKQIQETQDSKVR
EGLERELDCLVKQMEIKGEQICKLKKRQATVQ--KLKRKTQKLK--QEAAHGKQKCGDQKEAKEIAVT--IR
KSVSKSCAGQKSRNSLQLLKNVQKLQSTLKKDGIMWEQ
>XP_009461636.1/1-467 PREDICTED: centrosomal protein CEP57L1 [Nipponia nippon]

---MDSESKNSFI----GSFLQPP----
-D-------KL-LPAAFACIESK------------KL------AAVGCDR-SSIPN-------NQ-------
-------AVVAALKTLKEKIRRLELEKSQAEDNLCSLSIAAAQYKKALEHESYKKDTAHQELMQQRKDVSVQ
LNAAQSRCSLLEKQLDYMRKMVSSAELEKKMVLEQQ------------------------------------
---------------KEEVQNRLELHAKLEKLEMLEKECLKLTATQRIAEDKIKHLEEKLCKEEHQCKLMQD
KTAQLQTGFEINRILMSSVTSQNEHKKENGKKKKTK-------------------K------RNPAMKKMHL
PQLHVKAGELPFVAG-------K------SVGSS-HSVSANV-QSVLHIMKHRN-P--HISSRSQGGATS--
-----GISGCSALS----KSVSSCSTSP-TATRSLSDLLLLIQDELGQMSFEHQE---LLKQIQETQDSKVH
EDLERELDCLVKRMEIKGEQISKLKKHQATVQ--KLKRKTQKLK--QGAAHIKLKCGDQKEAKEIAVT--VR
ECTSKSCPGQKSRSSLQLLKNVQKLQSTLKKDDIMWEQ
>XP_009324697.1/1-467 PREDICTED: centrosomal protein CEP57L1 [Pygoscelis adeliae]

---MDSESKNSFI----GSFLQPP----
-D-------KM-LPAAFAYIESK------------KL------AAVGGDK-PSIPN-------NQ-------
-------AVVAALKTLQEKIRRLELEKSQAEDNLCSLSIAAAQYKKALEHESYKKDTAHQELMQQRKDVSVQ
LNAAQSRCSLLEKQLDYMRKMVSSAELEKEMVLEQ-------------------------------------
--------------QKEEDQNRLELHAKLEKLEILEKECLKLAATQRITEDKIKHLEEKLCKEEHQRKLIQD
KTAQLQTGFEINRILMSSVTSQNEPKKENGQKKKTK-------------------K------RNPTMKKTHL
SQLHVKAGELPFVAG-------K------SVGSS-HSVSANV-QSVLHIMKHRN-P--CISSRSQGGATS--
-----GISGRSALS----KSVSSCSTSP-TATRSLSDLLLAIQDELGQMSFEHEE---LLKQIQETQDSKVR
EDLEGELDCLVKQMEIKGEQISKLKKHQATVQ--KLKIKTQKLN--QGAAHVKLKCGDQKEEKKIAVT--VR
ESMSKSCPGQKSRSSLQLLKNVQKLQSTLKKDDIMWEQ
>XP_019330150.1/1-470 PREDICTED: centrosomal protein CEP57L1 [Aptenodytes forsteri]

---MDSESKNSFI----GSFLQPP----
-D-------KM-LPAAFAYIKSK------------KL------AAVGGDK-PSIPN-------NQ-------
-------AVVAALKTLQEKICRLELEKSQAEDNLCSLSIAAAQYKQALEHESYKKDTAHQELMQQRKDVSVQ
LNAAQSRCSLLEKQLDYMRKMVSSAELEKEMVLEQQ------------------------------------
-----------AQLQKEEDQNRLELHAKLEKLEILEKECLKLAATQRIAEDKIKHLEEKLCKEEHQRKLIQD
KTAQLQTGFEINRILMSSVTSQNEPKKENGQKKKTK-------------------K------RNPTMKKTHL
SQLHVKAGELPFVAG-------K------SVGSS-HSVSANV-QSVLHIMKHRN-P--CISSRSQGGATS--
-----GISGRSALS----KSVSSCSTSP-TATRSLSDLLLAIQDELDQMSFEHEE---LLKQIQETQDSKVR

EDLEGELDCLVKQMEIKGEQISKLKKHQATVQ--KLKIKTQKLK--QGAAHVKLKCGDQK-EKKIAVT--VR
ESMSKSCPGQKSRSSLQLLKNVQKLQSTLKKDDIMWEQ
>XP_050750837.1/1-471 centrosomal protein CEP57L1 [Gymnogyps californianus]

---MDSESKNSFI----GSFLQPP----
-D-------KM-LPAAFAYIESK------------TL------AAVGGDR-PSIPN-------NQ-------
-------AVVAALKTLQEKIRRLELEKSQAEDNLCSLSIAAAQYKKALEHESCKKDTAHQELMQQRKDVSVQ
LNAAQSRCSLLEKQLDYMRKMVASAELEKKVVLEQQ------------------------------------
----------AQVQKEEDQNRLELHAKLEKLEMLEKECLKLTATQRIAEDKIKHLEEKLCKEEHQRKLIQD
KTAQLQTGFEINRILMSSVTSQKEPKKENGKKKKTK-------------------K------RNPAMKKVHI
SQLRVKAGELPFVAG-------K------SVSSS-HSVSANV-QSVLHIMKHRN-P--RISSQSQGGATS--
-----GISGRSALS----KSVSSCSTSP-TATRSLSDLLLAIQDELGQMSFVHQE---LLKQMQETQDSKVR
EYLEQELDCLVKQMETKGEQISKLKKHQATVQ--KLKRKTQKLK--QGAAHVKLKCDDQKKAKEAAVS--VQ
ESMSKSCPGQKSRSSLQLLQNVRKLQSTLKKDDIMWEQ
>XP_059687604.1/1-471 centrosomal protein CEP57L1 [Gavia stellata]

---MDSESKNSFI----GSFLQPP----
-D-------KM-LPASFAYIESK------------KL------AAVGGDR-PSIPN-------NQ-------
-------AVVAALKTLQEKIRRLELEKSQAEDNLCSLSIAAAQYKKALEHESYKNDTAHQELMQQRKDVTVQ
LNAAQSRCSLLEKQLDYMRKMVSSVELEKKMVLEQQ------------------------------------
----------AQLQKEEDQNRLELHAKLEKLEMLEKECLKLTATQRIAEDKIKHLEEKLREEEHQRKLIQD
KTAQLQAGFEINRILMSSVTSQNKPKKENGKKKKTK-------------------K------RNPTMKKMHL
SPLHVKAGELPFVAG-------K------SVGSS-HSVSANV-QSVLHIMKHRN-P--RVSSRSQGGATS--
-----GIAGHSAFS----KSVSSCSTSP-TATRSLSDLLLTVQDELGQMSFEHQE---LLKQIQDTQDSKVR
EDLEQVLDCLVKQMEIKGEQISKLKNHQATVQ--KLKRKTQKLK--QETAHDKLKCGDKKKAKEIAVT--VR
ESMSKSCPGQKSRSSLQLLKNVQKLQSSLKKDDIMWEK
>KAK4824334.1/1-474 hypothetical protein QYF61_013669, partial [Mycteria americana]

--LFLQ--
-------------------A---------------------------MDSESKNSFI----GSFLQPP----
-D-------KM-LPAAFAYIESK------------KL------AAV-GDR-PSIPN-------NQ-------
-------AVMAALKTLQEKIRRLELEKSQAEDNLCSLSIAAAQYKKALEHESYKKDTAHQELMQQRKDISVQ
LNAAQSRCSLLEKQLDYMRKMVSSAELEKKMFLE-Q------------------------------------
----------AQLQKEEDQNRLELHAKLEKLEMLEKECLKLTATQRIAEDKIKHLEEKFCKEEHQRKLIQD
KTAQLQTGFEINRILMSSVTSQNEPKKENGKNKKTK-------------------K------RNPTMKKMHL
SQLRVRAGELPFVAG-------K------SVGSS-HSVSANL-QSVLHIMKHRN-P--RTSSRRQGGATS--
-----GISGRSALS----KSVSSCSTSP-TATRSLSDLLLAIQDELGQMSFEHQE---LLKQIQETQDSKVR
EDLERERDCLVKQMEIKGEQISKLKKHQASVQ--KLKRRTQKLK--QGAAHVRLKCGDQKEAKEIAVT--VR
KSMSKPCPGQKSRSSLQLLKNVQKLQSTLKKDDIMWAQ
>PKU46040.1/1-475 centrosomal protein cep57l1 [Limosa lapponica baueri]

--MGKL--
-------------------A---------------------------MDSESKNSFI----GSFLQPP----
-D-------KM-RPAAFAYIESK------------KL------AAVGGDR-PSIPN-------NQ-------
-------AVVAALKTLQEKIRHLELEKSQAEDNLCSLSIAAAQYKKALEHESYKKDTVHQELMQQRKDISVQ
LNAAQSRCSLLEKQLDYMRKMVFSAELEKKRVLEQQ------------------------------------
----------AQLQKEEDQNRLELHAKLEKLEMLEKECLKLTATQRIAEDKIKHLEEKLCKEEHQRKLIQE
KTAQLQTGFEINRILMSSVTSQNEPKKENRKKKKTK-------------------G-------NPTMKKMHL
SQLHVKAGELPFVAG-------K------SVSCS-HSVSANV-QSVLHIMKHRN-P--RISPRSQGRATS--
-----GISGHSAVS----KSVSSCSTSP-TATRSLSDLLLAIQDELGQMSFKHRE---LLKKIQETQDSKVH

EDLEQELDCLVKQMEIKGEQISKLKKHQAAVQ--KLKRKIQKLK--QGAAHVALKCGDQKKAKEIPVT--VR
KSLSKSCPGQKSRSSLQLLKNVQKLQSTLKKDDIMWEQ
>XP_032861600.1/1-471 centrosomal protein CEP57L1 [Tyto alba]XP_032861601.1 centrosomal protein CEP57L1 [Tyto alba]XP_032861602.1 centrosomal protein CEP57L1 [Tyto alba]XP_032861603.1 centrosomal protein CEP57L1 [Tyto alba]XP_032861605.1 centrosomal protein CEP57L1 [Tyto alba]

--MDSESKNSFI----GSFLQPP----
-D-------KM-LPAAFAYIESK-----------KL------TAVGGDR-LSTPN-------NQ-------
-------AVVAALKTLQEKIRRLELEKSLAEDNLCSLSIAASQYKKALEHVSYKKDIAHQELMQQRKDISMQ
LNAAQSRCSLLEKQLDYMREMVSSAELEKKMVLEQQ-----------------------------------
-----------AQLQKEEDQNRLQLHAKLENLKMLEKECLKLTATRRIAEDKIKHLEENLRKEEHQHKLIQD
KTAQLQTGFQINRILMSSVTSQNEHKKQNRKKKKTK-------------------K------RNPTMKKTHL
SQLHVKAGELPFVAG-------K------SVSSS-HSVSANI-QSVLYIMKHRN-P--RISSRSQGGATS--
-----GISGHSALS----KYVSSCSASP-TTTRSLSDLLLAIQDELDQMSFEHQE---LLKQIQETQDSKVR
EDLERELKSLVKQIEIKGEQISKLKKHQSTVQ--KLKRKTQKMK--QGAAHVKLKCGDQMEAKEIALT--VM
ESMSKSCPGQKSRSSLQLLKNVQKLQSTLKKDDIMWEQ
>XP_028941020.1/1-471 LOW QUALITY PROTEIN: centrosomal protein CEP57L1 [Antrostomus carolinensis]

--MDFESKNSFI----GSFLQPP----
-D-------KM-LPAAFAYIESK-----------KL------AAVGGDR-ASIPN-------NQ-------
-------AVMTALKMLQEKIRRLELEKSQAEDNLCSLSIEAARYKKALDHESHKKDTVHQELMQQRKDVSVQ
LNAAQSRCSLLEKQLDYMRKMVSSAELEKKMVLKQQ-----------------------------------
-----------AQFQKEEDQNQLELHAKLEKLEMLEXFCLKLTATQRTAEYKIKQLQEKLCKEELQRKLIQD
KTAQFQTGFEISRILMSSVTSQNEPKKENGKKKKTR-------------------K------RNPPVKKTHL
RQLNVKAGELPFVAG-------K------SVSSS-HSVSANV-QSVLHMMKHRN-P--HTLSRSQGGATS--
-----GISGRSARS----KSVSSCSTSP-TGTRNLSDLLLSIQDELGQMSFEHQE---LLKQIQETQDSRVR
EDLEWELDCLVKQMEIKGEQISKLKKHQATVQ--KLKRKTQKLK--QGEAHVRLKCGDQKEAKEITVA--VR
ESMSKSCPGQKSRSSLQLLKNVRKLQSTLKKDDIMWEQ
>NWU60935.1/1-439 CE57L protein [Pterocles burchelli]

--MDSESKNSFI----GSFRQPP----
-D-------KM-LHAAFASIESK-----------KL------AAVGVDR-PSIPD-------NQ-------
-------AVVAALKTLQEKIRHLELEKSQAEDNLCSLSITAAQYKKTLERESHKKDVARQELMQRRKDVSLQ
LSAAQSRCSLLKKQLDYMRKMVSSAELENKMVLEQQ-----------------------------------
-----------AQLQKEEDQNCLELHAKLEKLEMLEKECLKLTAAQKIAEDKIKHLEEKLRKAEHQRKLIQD
KTAQLQTGFEISRILMPSITSLNEPKKEN-NNKKTK-------------------K------RNPAMKKVHV
SQLHVKAGELPFVAG-------K-------------VSATS------------------------------
-----GSSGHSALS----KSVSCCSTSP-TATRSLSDLLLARQDELGQMSFEHQE---LLKQIQETQDSKVR
EDLGQELDCLVKQMEIKGEQVSKLKNHQAAVQ--KLKRKTQKLK--QGAAPVKLTRGEQMEAKDIAVT--VR
ESMSKSCPGQKGRSSLQLLKNVQKLQSALKNDYVMWEQ
>NXV77401.1/1-438 CE57L protein [Atlantisia rogersi]

--MDSESKSSFI----GSFLQPP----
-D-------KM-LPAAFDYIESK-----------KL------AAVGGDR-PSIPN-------NQ-------
-------AVVAALKTLQEKIRRLELEKSQAEDNLCSLSIAAAQYKKALEHESDKKDTARQELMQQRKDVSVQ
LNAAQSRCSLLEKQLDYMRKMVSSAELERKMVLEQQ-----------------------------------
-----------AQLQKEEDQNWLELHAKLEKLEMLEKECVKLTATQRIAEDKIKHLEEKLCKEEYRRKLIQD

KTAQLQTGFEINRILMSSVTSQNEPKRENGKDKKTK-------------------K------RNPTVKKVQL
SQLHVKAGELPFVAG-------K------VSATK-HSCQIDI-YR--------------------------
---------RSALP----KSSFS------TSMRSHSDLLLAIQDELGRMSFEYQE---LLKQIEETQDAKVC
EDLEQELDCLVKQMEIKAVQISKLKKHQASVE--KLKSKTQKLK--QGAVRAKLKCSDQKKAKETA----IR
ESMPKCSPEQKSRSSLQLLKTVQKLQSSLKKNDIVWEQ
>XP_032041083.1/1-467 centrosomal protein CEP57L1 [Aythya fuligula]

---MDSESKDSFI----GSFLQPP----
-D-------KM-IPASFAYIESK------------KL------AAVGVDG-PSISN-------NQ-------
-------AVVAALRTLQEKIHRLELEKSQAEDNLCSLSIAAAQYKKALEHESYKKDRAHQELMQQRKDISVQ
LNAAQSRCSLLEKQLDYMRKMVSSAELEKKIVLEQ-----------------------------------
--------------QKEKDQNQMELHAKLEKLEVLEKECLKLTATQRIAEDKIKHLKEKLSEEEHQHKLLQD
KAAQLQKGFEISRILMSSVSSENECKKKNRKKNKHK-------------------K------KNPTMKKTHP
SQFHVKAGVLPFVAG-------K------SVSSS-HSVSANV-QSVLHIMKHRC-P--RITSQRQDGATS--
-----GISRQTALS----KPVSSCSTSS-TVTGSLSDLLLAIQDERSQMSFEHQE---LLKQIQETQDSKVR
EDLEQKLSCFVKQMNIKEEQISKLKKHQTSVQ--KLKRKTQKLK--QEAAHVKLKYGNQKEAKEIAVT--VR
QSMSKSHAGQKSSSSLQLLKNVQKLQSTLKKDDIMWEQ
>XP_027310529.2/1-472 centrosomal protein CEP57L1 isoform X1 [Anas platyrhynchos]XP_038031749.1 centrosomal protein CEP57L1 isoform X1 [Anas platyrhynchos]XP_038031750.1 centrosomal protein CEP57L1 isoform X1 [Anas platyrhynchos]XP_038031751.1 centrosomal protein CEP57L1 isoform X1 [Anas platyrhynchos]

---MDSESKDSFI----GSFLQPP----
-D-------KM-IPASFAYIESK------------KL------AAVGVDE-PSISN-------NQ-------
-------AVVAALRTLQEKIHRLELEKSQAEDNLCSLSIAAAQYKKALEHESYKKDTAHQELMQQRKDISVQ
LNAAQSRCSLLEKQLDYMRKMVSSAELEKKMVLEQQ----------------------------------
-----------TQLQKENDQNQVELHAKLEKLEVLEKECLKLTATQRIAEDKIKHLEEKLFEEEHQHKLLQA
KAAQLQKGLEISRILMSSVSSENECKKKNRKKNKPK-------------------KQ-----KNPTVKKMHP
SQFHVKAGVLPFVAG-------K------SVSSS-HSVSANV-QSVLHIMKHRC-P--RITSQRQDGATS--
-----GISGQTALS----KPVPSCSASS-TVTGSLSDLLLAMQDEMSQMSFEHQE---LLKQIQETQDSKVR
EDLEQKLSCLVKQMNIKEEQISKLKKHQTSVQ--KLKRKTQKLK--QEAAHVKQKYGNQKEAKEIAVT--VR
QSMSKSHAGQKSSSSLQLLKNVQKLQSTLKKDDIMWEQ
>XP_035179083.1/1-470 centrosomal protein CEP57L1 isoform X1 [Oxyura jamaicensis]XP_035179084.1 centrosomal protein CEP57L1 isoform X1 [Oxyura jamaicensis]XP_035179085.1 centrosomal protein CEP57L1 isoform X1 [Oxyura jamaicensis]XP_035179086.1 centrosomal protein CEP57L1 isoform X1 [Oxyura jamaicensis]XP_035179087.1 centrosomal protein CEP57L1 isoform X1 [Oxyura jamaicensis]

---MDSESKDSFI----GSFLQPP----
-D-------KM-IPASFAYTESK------------KL------AAVGVDG-PSISN-------NQ-------
-------AVVAALRTLQEKIHCLELEKSQAEDNLCSLSIAAAQYKKALEHESYKKDITHQELMQQRKDLSVQ
LNAAQSRCSLLEKQLDYMRKMVSSVELEKKMVLEQQ----------------------------------
-----------TQLQK--DQNQVELHAKLEKLEILEKECLKLSATQRIAEDKIKHLEEKLSKEEHQHKLLQD
KATQLQKEFEISRILMSSLSSENECKKKNRKKNKPK-------------------KQ-----KNATMKKTCP
SQFHVKAGVLPFVAG-------K------SVSSS-HSVSANV-QSVLHIMKHRC-P--RITSRRQDGATS--
-----GISGQTALS----KPVLSCSTSS-TVTGSLSDLLLAIQDEMSQMSFEHQE---LLKQIQETQDSKVH
EDLEQKLSCLVKQMNIKEEQISKLKKHQTSVQ--KLKRKTQKLK--QEAAHVKLKYGNQKEAKEITVT--VR
PSMSKSRAGQKSSRSLELLKNVQKLQLTLKEDDIMWEQ
>XP_035420718.1/1-472 LOW QUALITY PROTEIN: centrosomal protein CEP57L1 [Cygnus atratus]

---MDSESKDSFI----GSFLQPP----
-D-------KM-IPASFAYTESK------------QL------AALGVDM-PSISN-------NQ-------
-------AVVAALRTLQEKIHRLELEKSQAEDNLCSLSIAAAQYKNALEHESYKKDTAHQELMQQRKVISVQ
LNATQSRCSLLEKQLDYMRKMVSSAELEKKMVLEQQ------------------------------------
-----------TQLQKEKDQNQVELHAKLEKLEVLEKECLKLTATQKIAEDKIKHLEEKLSKEEHQHKLIQD
KATQLQKGFEISRISMSSVSSENERKKKKRKKNKPK-------------------KQ-----KNPTMKKTHP
SQFHVKAGVLPFVAG-------K------SVSSS-HSVSANV-QSVLHIMKHRC-P--RITSQRQDGATP--
-----GISGQTALS----KPVSSCSTSS-TVTGNLSDLLLAIQDEMRQRSFEHQE---LLKQIQETQDSKVQ
EDLEQKLSCLVKQMNIKEEQISKLKKHQTSVQ--KLKRKTQKLK--QEAAHVKLKSGNQKEAKEITVT--VR
QSMSKSRAGQKSSSSLQLLKNVQKLQLALKKDDIMWEQ
>XP_040407568.1/1-474 centrosomal protein CEP57L1 isoform X5 [Cygnus olor]

---M--
-------------------A---------------------------MDSESKDSFI----GSFLQPP----
-D-------KM-IPASFAYTESK------------QL------AALGVDM-PSISN-------NQ-------
-------AVVAALRTLQEKIHRLELEKSQAEDNLCSLSIAAAQYKKALEHESYKKDTAHQELMQQRKVISVQ
LNATQSRCSLLEKQLDYMRKMVSSAELEKKMVLEQQ------------------------------------
-----------TQLQKEKDQNQVELHAKLEKLEVLEKVCLKLTATQKIAEDKIKHLEEKLSKEEHQHKLIQD
KATQLQKGFEISRISMSSVSSENERKKKKRKKNKPK-------------------KQ-----KNPTVKKTHP
SQFHVKAGVLPFVAG-------K------SVSSS-HSVSANV-QSVLHIMKHRC-P--RITSQRQDGATS--
-----GISGQTALS----KPVSSCSTSS-TVTGSLSDLLLAIQDEMSQMSFEHQE---LLKQIQETQDSKVR
EDLEQKLSCLVKQMNIKEEQISKLKKHQTSVQ--KLKRKTQKLK--QEAAHVKLKSGNQKEAKEITVT--VR
QSMSKSRAGQKSSSSLQLLKNVQKLQLALKKDDIMWEQ
>XP_040407566.1/1-485 centrosomal protein CEP57L1 isoform X3 [Cygnus olor]

-MYLQF---RVYLEEA--
-------------------A---------------------------MDSESKDSFI----GSFLQPP----
-D-------KM-IPASFAYTESK------------QL------AALGVDM-PSISN-------NQ-------
-------AVVAALRTLQEKIHRLELEKSQAEDNLCSLSIAAAQYKKALEHESYKKDTAHQELMQQRKVISVQ
LNATQSRCSLLEKQLDYMRKMVSSAELEKKMVLEQQ------------------------------------
-----------TQLQKEKDQNQVELHAKLEKLEVLEKVCLKLTATQKIAEDKIKHLEEKLSKEEHQHKLIQD
KATQLQKGFEISRISMSSVSSENERKKKKRKKNKPK-------------------KQ-----KNPTVKKTHP
SQFHVKAGVLPFVAG-------K------SVSSS-HSVSANV-QSVLHIMKHRC-P--RITSQRQDGATS--
-----GISGQTALS----KPVSSCSTSS-TVTGSLSDLLLAIQDEMSQMSFEHQE---LLKQIQETQDSKVR
EDLEQKLSCLVKQMNIKEEQISKLKKHQTSVQ--KLKRKTQKLK--QEAAHVKLKSGNQKEAKEITVT--VR
QSMSKSRAGQKSSSSLQLLKNVQKLQLALKKDDIMWEQ
>XP_040407564.1/1-506 centrosomal protein CEP57L1 isoform X1 [Cygnus olor]

--MSTPALARWERA--------
--SEHH-CCSHQ
HRLGQF---QLK-KQR--
-------------------A---------------------------MDSESKDSFI----GSFLQPP----
-D-------KM-IPASFAYTESK------------QL------AALGVDM-PSISN-------NQ-------
-------AVVAALRTLQEKIHRLELEKSQAEDNLCSLSIAAAQYKKALEHESYKKDTAHQELMQQRKVISVQ
LNATQSRCSLLEKQLDYMRKMVSSAELEKKMVLEQQ------------------------------------
-----------TQLQKEKDQNQVELHAKLEKLEVLEKVCLKLTATQKIAEDKIKHLEEKLSKEEHQHKLIQD
KATQLQKGFEISRISMSSVSSENERKKKKRKKNKPK-------------------KQ-----KNPTVKKTHP
SQFHVKAGVLPFVAG-------K------SVSSS-HSVSANV-QSVLHIMKHRC-P--RITSQRQDGATS--
-----GISGQTALS----KPVSSCSTSS-TVTGSLSDLLLAIQDEMSQMSFEHQE---LLKQIQETQDSKVR
EDLEQKLSCLVKQMNIKEEQISKLKKHQTSVQ--KLKRKTQKLK--QEAAHVKLKSGNQKEAKEITVT--VR
QSMSKSRAGQKSSSSLQLLKNVQKLQLALKKDDIMWEQ
>NWZ24584.1/1-439 CE57L protein [Asarcornis scutulata]

---MDSESKDSFI----GSFLQPP----
-D-------KM-IPASFAYIESK------------KL------AAVGVDG-PSISN-------NQ-------
-------AVVAALRTLQEKIHRLELEKSQAEDNLCSLSIAAAQYKKALEHESYKKDRTHQELMQQRKDISVQ
LNAAQSRCSLLEKQLDYMRKMVSSAELEKKMVLEQQ------------------------------------
-----------TQLQKEKDQNQGELHAKLEKLEVLEKECLKLTATQRIAEDKIKHLKEKLSEEEHQHKLLQD
KAAQLQEGFEISRILMSSVSSENECKMKNRKKNKHK-------------------K------KNPTMKRTHP
SQFHVKAGMLPFVVG-------K------V-------------------------------------PTS--
-----GISRQTALS----KPVSSCSTSS-TVTGSLSDLLLAIQDERSQMSFEHQE---LLKQIQETQDSEVR
EDLEQKLSCLVKQMNIKEEQISKLRKHQTSVQ--KLKRKTQLK--QEAAHVKLKYGNQKEAKEIAVT--LR
QSMSKSHAGQKSSSSLQLLKNVQKLQSTLKKDDIKWEQ
>EOB03555.1/1-445 Uncharacterized protein C6orf182, partial [Anas platyrhynchos]

---MDSESKDSFI----GSFLQPP----
-D-------KM-IPASFAYIESK------------KL------AAVGVDE-PSISN-------NQ-------
-------AVVAALRTLQEKIHRLELEKSQAEDNLCSLSIAAAQYKKALEHESYKKDTAHQELMQQRKDISVQ
LNAAQSRCSLLEKQLDYMRKMVSSAELEKKMVLEQQ------------------------------------
-----------TQLQKENDQNQVELHAKLEKLEVLEKECLKLTATQIIAEDKIKHLEEKLFEEEHQHKLLQA
KAAQLQKGLDISRILMSSVSSENECKKKNRKKNKPK-------------------K------KNPTVKKMHP
SQFHVKAGVLPFVAG-------K------SVSSS-HSVSANV-QR---------------------------
---------QTALS----KPVPSCSASS-TVTGSLSDLLLAMQDEMSQMSFEHQE---LLKQIQETQDSKVR
EDLEQKLSCLVKQMNIKEEQISKLKKHQTSVQ--KLKRKTQLK--QEAAHVKQKYGNQKEAKEIAVT--VR
QSMSKSHAGQKSSSSLQLLKNVQKLQSTLKKDDIMWEQ
>NXI74358.1/1-445 CE57L protein [Anseranas semipalmata]

---MDSESKDGFI----GSFLQPR----
-D-------KM-IPAPFAYTESK------------NL------AAVGGDG-PSIPN-------NQ-------
-------AVVAALRTLQEKIHRLELERSQAEDNLCSLSIAAAQYKKALEHDSYKKDTAHQELMQQRKDISVQ
LNAAQSRCSLLEKQLDYMRKMVSSAELENKMFLEQQ------------------------------------
-----------TQLQNEKDQNQVELHAKLEKLEVLEKECLKLTATQRIAEDKIKHLEEKLCKEEHQHKLIQD
KAAQLQTGFEINRILMSSVSSENEPKKKNRKKTKNK-------------------K------KNPAMKKTHL
SQFYVKAGVLPFVAG-------K------SVSSS-HSVSANV-QR---------------------------
---------QAALS----KSVSSCSTSS-TVTRSLSDLLLAIQDELSQMSFEHQE---LLKQIQETQDSKVR
EDLEQKLSCLAKQMDVKGEQISKLKKHQTSVQ--KLKRKTQLK--QEAAHVKLKYGNQKEAKEITVT--VR
EGMSKSCAGQKSRSSLQLLKNVQKLQSTLKKDDIMWEQ
>NXG31275.1/1-438 CE57L protein [Dromaius novaehollandiae]

---MGSESKHSFI----GSFLQPP----
-D-------KI-LPAAFAYRESK------------KL------TAVGGDRLSPVPS-------NQ-------
-------AVVAALKTLQEKIHRLELERSQAEDNLCSLSAEAALYKKALEQESYRKDISHQELMHQRKDISVQ
LNAAQSRCSLLEKQLDYMKEMVSSAELEKKMVLEQQ------------------------------------
-----------NQLQKEENQNQVELHAKLEKLEVLEKQWLKLTATQRIAEDKIKHLEEKLCKEEHQRKLIQD
QAAQLQTGFEINRILLSLVSSQNESKKKNRKKKKNK-------------------K------KNPTVKKRCL
PQFHVKAGVLPFVAG-------K------VS----------------------------VTKR---------
---------QTIAS----KSVSSYSTSS-TVTRNLSDLLLAIEGELSQMSFEHQE---LLKQIQETHNSEVR
EDLEWELDCLVKKMEIKGEQISKLKKHQASVQ--KIKQKTQLK--QEAAHVKLKCGDQKETKEIAVA--IR
ESRSKACPGQKSKSSLQLLKNVQKLQSTLKRDDIMWEQ
>NXE51093.1/1-438 CE57L protein [Casuarius casuarius]

---MESESKHSFI----GSFLQPP----
-D-------KI-LPAAFAYRESK------------KL------TAVGGDRLSPVPN-------NQ-------
-------AVVAALKTLQEKIHRLELERSQAEDNLCSLSAEAARYKAALEQESYRKDISHQELMQQRKDISVQ
LNAAQSRCSLLEKQLDYMKEMVSSAELEKKMVLEQQ------------------------------------
-----------NQLQKEKNQNQVELHAKLEKLEVLEKQCLKLTATQRIAEDKIKHLEEKLCKEEHQRKLIQD
KAAQLQTGFEINRIFLSLVSSQNESKKKNRKKKKNK-------------------K------KNPTVKKRCL
PQFHVKAGVLPFVAG-------K------VS----------------------------VTKR---------
---------QTIAS----KSVSSYSTSS-TVTRNLSDLLLAIEDELGRMSFEHQE---LLKQIQETHNSEVR
EDLEQELDCLVKKMEIKGEQISKLKNHQPSVQ--KIKQKTQKLK--PEAAHVKLKCGDQKETKEIPVA--IR
ESRSKACPGQKSKSSLQLLKNVQKLQSTLKRDDIMWEQ
>XP_025975679.1/1-477 centrosomal protein CEP57L1 [Dromaius novaehollandiae]

--MGKL--
-------------------A---------------------------MGSESKHSFI----GSFLQPP----
-D-------KI-LPAAFAYRESK------------KL------TAVGGDRLSPVPS-------NQ-------
-------AVVAALKTLQEKIHRLELERSQAEDNLCSLSAEAALYKKALEQESYRKDISHQELMHQRKDISVQ
LNAAQSRCSLLEKQLDYMKEMVSSAELEKKMVLEQQ------------------------------------
-----------NQLQKEENQNQVELHAKLEKLEVLEKQWLKLTATQRIAEDKIKHLEEKLCKEEHQRKLIQD
QAAQLQTGFEJNRILLSLVSSQNESKKKNRKKKKNK-------------------K------KNPTVKKRCL
PQFHVKAGVLPFVAG-------K------SASSS-HSVSANV-QSVLHIMKHRN-P--RVSSRGQEGTAS--
-----GISGQTIAS----KSVSSYSTSS-TVTRNLSDLLLAIEGELSQMSFEHQE---LLKQIQETHNSEVR
EDLEWELDCLVKKMEIKGEQISKLKKHQASVQ--KIKQKTQKLK--QEAAHVKLKCGDQKETKEIAVA--IR
ESRSKACPGQKSKSSLQLLKNVQKLQSTLKRDDIMWEQ
>XP_009675240.1/1-471 PREDICTED: centrosomal protein CEP57L1 [Struthio camelus australis]

---MDSESKHSYI----GSFLQPP----
-D-------KI-LPAAFAHSESK------------KL------TAVDGER-PPILN-------NQ-------
-------AVVAALKTLQEKIRRLELERSQAEDNLCSLSAEAAQYKKALEHESYKKDISHQELMQQRKGVSMQ
LNAAQSRCSLLEKQLDYMKKMVSSAELEKKMILEQQ------------------------------------
-----------TQLQREKNQNRVELHAKLEKLEVLEKQCLKLTATQRIAEDKIKHLEEKLCEEEHQRKLIQE
KAAQLQTGFEINRILLSLASSQNELKKKNRKKKKTK-------------------K------KNPTVKRMYL
PQFNVKAGVLPFVAG-------K------SASSS-HSVSANV-QSVLHIMKHRN-P--PFSSRGQEGAAS--
-----GISGQTVAS----KSVSSFSTIS-TATRNLSDLLLAVEDELGQMSFEHQE---LLRQIQGTHNSEVR
EDLERELDCLVKKMEIKGEQISKLKKHQANVQ--KIKQKTQKLK--QEAAHVKLKCGDQKETKEIPVA--IR
ESRSKACPGQKSKSSLQLLKNVQKLQWTLKRDDIMWEQ
>NXD08238.1/1-455 CE57L protein [Nothocercus nigrocapillus]

---MDFESKYRFI----GSSLQPP----
-D-------KI-LPATFASKEPK------------KL------TAVADDGLSPLPN-------NQ-------
-------AVVSALKTLQEKIRRLELERSQAEDNLCSLSAEAARYKKALEQESYKKDISHQELMQQRKDVSVQ
LNAAQSRCSLLEKQLDYMKKMVASAEMEKKMVLEQQ------------------------------------
-----------TQLQKEKNQNRVELHAKLEKLEVLEKQCLRLTATQRIAEDKIKHLEEKLCKEEHQRKLIQD
KAAQLQTGFEINRILLSLVSSQNERKKKIRKKKTKKHIGISCLFLF-----IL-WN------KNPAVKKICL
LPFHVRAGVLPFVAG-------K---GAAT--
-----GISRQTTAS----KSVSSCSMSS-TVTRNLSDILLAMQDELGQMSFEHQE---LLKQIQETHNSEVL
EDLEQQLDCLVKKMEIKGEQICKMKKHQASIRKNKIKQKTQKLK--QEAAHIKLKCDDQKETKESQVA--IR
ESKSKACPGQKSKSSLQLLKNAQKLQSTLKKDDIMWER
>XP_033619328.1/1-326 centrosomal protein CEP57L1 isoform X7 [Fukomys damarensis]

--MR--------------------

---------------------------------PKK------------------------------------
-----------AQLQREKEQDQMKLHAKLEKLDVLEKECFRLTTTQKTAEDKIKHLEEKLQEEEHQRKLFQD
KAAELQTGLEISKILMSSVSNSKHSKE---KKKSSK--------------------------KSKCLKKGPP
LQIYSKFRAPLFEAK-------K------SASAG-CSVNAGR-QSLQHKTYHCS-P--HGFPKGTQVIEPRC
LCK----PPRT-IS-QY-KAVPRESEKSISICDNLSELLMAMQDELDQMSMEHEE---LLKQMKETESHSIC
DNIEYELERLVKKMEIKEKQISKLKTHQDNVH--KLQQKVQNSKT-SKASGIQQDSN--QSLKSMKNS--PR
KCLNKANSSQK-SSNFHPIQ-VHNIQVKLRRDDITWEQ
>XP_015352762.2/1-337 centrosomal protein CEP57L1 [Marmota marmota marmota]

--------------------MVLNVEREKNMILEQQ------------------------------------
-----------AQLQREKEQDQLKLHAKLEKLDVLEKECFRLTTTQKTAEDKIKHLEEKLTEEEHQRKLFQD
KASELQTGLEISKILMSSVSKSKLSKE---KKKSSK--------------------------KTKSLKRGPT
QQIYSKLRELPFEAE-------K------SAA---SSANGSM-YSLLKMMRHCS-Q--CSLQKP-EVTEPRC
LYR----PTRK-TS-QY-KAVPQDSEKSISICDNLSELLMAMQDELDQMSMEHEE---LLKQMKETESHSVC
DDIECELEHLVKKMEIKGEQISKLRKHQDNVY--KLQQKVQNSKMLSNTSVIQQEDSNLKGSKNIKNS--PR
KCLNETNPFQK-NNNFHPVQ-VHNLQMKLRRDDIMWEQ
>OWK01436.1/1-359 CEP57L1 [Cervus elaphus hippelaphus]

--MQ
LSSAQSRCILLEKQLEYTKRMVLNVEREKNMILEQQ------------------------------------
-----------AQLQREKEQDHMRLHAKLEKLDVLEKECFKLTTTQKTAEDKIKHLEEKLKEEEHQRKLFQD
KASQLQTGLEINRILMSSVTNPRHFKE---KKKSSK--------------------------KTKCLRRGPS
QQICSKFGTLPIVAE-------K------SASAS-HSANAST-QN-LQTVPHYG-P--HILQKPAEVTELRC
LYK----PSRT-TS-QC-KAVPSDSEKSISICNNLSELLMAMQDELDQMTMEYQD---LLNQMKETESQSVC
EDIECELERLVKKMEIKGEQISKLMQHQDSVR--KLQQKVQNSKM-SETSALQQEDGNCKGTKNIKNS--SR
KCLLTNS-LQK-NSNFRPIR-VHNLQMKLRRDDIMWEQ
>XP_047618335.1/1-369 centrosomal protein CEP57L1 isoform X5 [Phacochoerus africanus]

---MDSVSLY------------------

--ISMQ
LSSAQSRCTLLEKQLEYTKRMVLNVEREKDMILEQQ------------------------------------
-----------AQLQREKEQDHMKLQAKLEKLDVLEKECFKLTTTQKTAEDKIKHLEEKLKEEEHQRKLFQD
KASQLQTGLAINRILMASVSNPKCSKE---KKKSSK--------------------------KTKCLKRGPP
QQIYSKFGSLPVVAE-------K------SASAS-HSVNAST-QNLLQTMQHYG-P--HILQKPAKVIEPRY
LYK----PSRT-TS-QC-KAVPSDSEKSISICDNLSELLMAMQDELDQMSIEYQE---LLSQMKETESQSVC
EDIECELEHLVKKMEIKGERISKLMKHQDSVR--KLQQKVQSSKM-SEASAIQQQDSNLKGSKNIKNS--PR
KCLLTNS-LQK-NSSFHPIR-VHNLQMKLRRDDILWEQ

>XP_007447338.1/1-321 PREDICTED: centrosomal protein CEP57L1 [Lipotes vexillifer]

---MDSELMHSIV----GSYLKPP----
-E-------RV-LVPSITQSDES-----SQTHY--SV-----NLEVTSPKMLHSPN-------SQ-------
-------ALILALKTLQEKIHRLELERTQAEDNLNILSREAAQYKKALEDERKERNLAHEELIKQKKDISMQ
LSSAQSHCTLLEKQLEYTKRMVLNVEREKNMILEQQ------------------------------------
-----------AQLQREKEQDHMKLQAKLEKLDVLEKECFKLTTTQKTAEGKIKHLEEKLKEEEHQRKLFQD
KASQLQTGLEISRILMSSVSNPKRSKE---KKKSSK------------------------------------
----

------------------------------VR--KLQQKVQNLKM-SEASAIQKEDSNCKGSKNIKNS--PR
KYLLTNS-PQK-NSNFRPIR-VHNLQMKLRRDDIMWEQ
>XP_052020244.1/1-584 centrosomal protein CEP57L1 isoform X1 [Apodemus sylvaticus]

---MLLLLLCPRPPPPSQRAPRDP
AVIEPRAAPLKHRAPSTEPAPSP-SPVCGDRPRGREQGGETGSSPPAPPLSPPGTAGSRPC-PMPA--SPPR
-----HTR-PAKTAIGSSPALGQSTSGPR---VQCA--------------------PPIKAGCCACA-----
-------------------TPSRAHALSAALSVLDVWRRWLEGEGKTMDSELSHSMV----GSYLNPP----
-E-------RM-YLPSFTQNEAF------QNHH--LG-----NS----PKMFNSPN-------NQ-------
-------ALVSALKTLQEKIHRLELERTQAEDNLNLLSREAAQYKKALEEETTERDLAHEELIKQKKDISIQ
LSSAQSRCILLEKQLEYTKRMVLNVEREKTMILEQQ------------------------------------
-----------AQLQREKEQDQMKLHAKLEKLHVLEKECLRLTATQQTAEDKIKYLEEKLKEEEHQRRLFQD
KACELQTGFEISKILMSTVSNSKHCKE---KKKLPK--------------------------KTNCLKREPP
QQMDHKFRVPTFEK--
-EK----PFRA-TS-QA-RANAHSSGEPVSICDSLSELLMTMQEELAQMNMEHRE---LLRQMMQTESHSVS
EDIELELEQLVKKMESKGDQISKLKKHQDSVR--KLQEKVENSRI-NESSGLH---GHPKGSKNLKTS--PR
KCVSETSAFQR-DSSFQPVQ-VHSLQAKLRRDDIKWEQ
